# Ligand-to-Copper Charge-Transfer-Enabled C–H Sulfoximination of Arenes

**DOI:** 10.1021/acs.orglett.3c00256

**Published:** 2023-02-03

**Authors:** Wanqi Su, Peng Xu, Roland Petzold, Jiyao Yan, Tobias Ritter

**Affiliations:** † Max-Planck-Institut für Kohlenforschung, Kaiser-Wilhelm-Platz 1, 45470 Mülheim an der Ruhr, Germany; ‡ Institute of Organic Chemistry, RWTH Aachen University, Landoltweg 1, 52074 Aachen, Germany

## Abstract

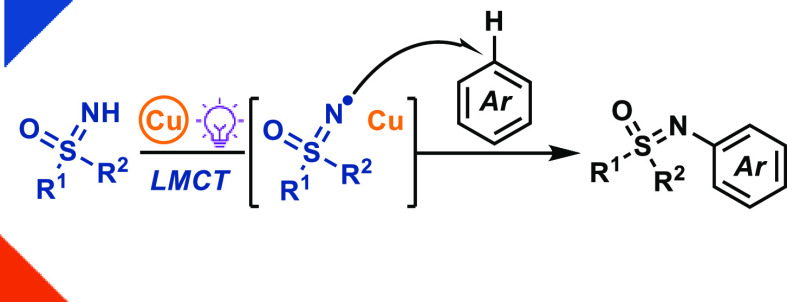

Herein, we report a photoinduced sulfoximine-to-copper charge-transfer-enabled generation of sulfoximinyl radicals directly from *NH*-sulfoximines for C–H sulfoximination of arenes via radical addition. Through copper-LMCT, N-arylation of *NH*-sulfoximines was achieved for the first time using arenes of different electronic structures as the aryl donors.

Nitrogen-centered sulfoximinyl radicals can be accessed from preactivated sulfoximines, such as N-halogenated sulfoximines^1^ and hypervalent iodine(III) reagents^2^ via homo- or mesolytic cleavage of weak nitrogen-heteroatom bonds. However, direct generation of sulfoximinyl radicals from *NH*-sulfoximines via either single electron transfer (SET) or hydrogen atom transfer (HAT) is challenging, due to the high oxidation potential (*E*_ox_ = +1.92 to +2.00 V vs SCE)^3^ and the high bond dissociation energy (BDE, BDE_N–H_ = 104–106 kcal/mol, by DFT calculation, Figure 1A, left). Apart from the challenges in their generation, synthetic applications of N-centered sulfoximinyl radicals are complicated by their propensity to engage in hydrogen atom abstraction, in preference to other desired radical addition processes.^1^ For example, even if sulfoximinyl radicals are generated, their addition to arenes has not been reported (Figure 1A, right). Herein, we report the direct generation of N-centered sulfoximinyl radicals from *NH*-sulfoximines via sulfoximine-to-copper charge transfer (LMCT). The approach differs conceptually from previous methods because the putative copper-sulfoximinyl radical complex generated by LMCT exhibits distinct reactivity in the sense that arene addition is observed, while the deleterious HAT can be suppressed (Figure 1B).

**Figure 1:**
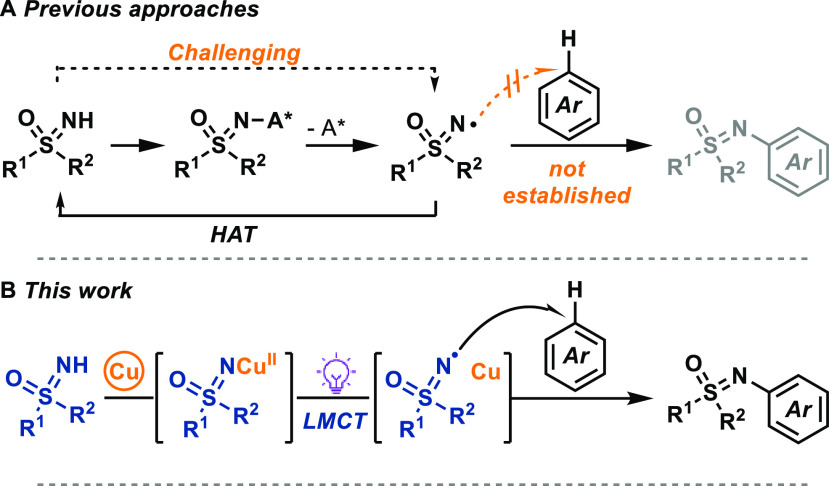
(A) Previous approaches to access sulfoximinyl radicals and challenges in their synthetic applications. (B) Aromatic C–H sulfoximination enabled by copper-LMCT.

Photoinduced ligand-to-copper charge transfer is a useful tool in organic synthesis to generate radicals^4^ from chlorides,^5^ azides,^6^ alkyl ligands,^7^ and carboxylates.^8^ Copper-mediated and copper-catalyzed benzoate-to-copper charge transfer have been introduced to produce aryl radicals directly from benzoic acids, and have enabled several previously inaccessible aromatic decarboxylative functionalization reactions (Figure 2A).^8a−8d,8g^ Herein, we show that the concept is general beyond copper benzoates, to now also achieve C–H functionalization of arenes. The LMCT approach enables a synthesis of *N*-aryl sulfoximines directly from *NH*-sulfoximines and arenes, which is currently unachieved. The fundamental advance of the work is the first example of arene C–H functionalization through copper-mediated LMCT for C–heteroatom bond formation. However, the requirement of excess arene and a relatively small functional group tolerance are the major drawbacks of the method.

**Figure 2:**
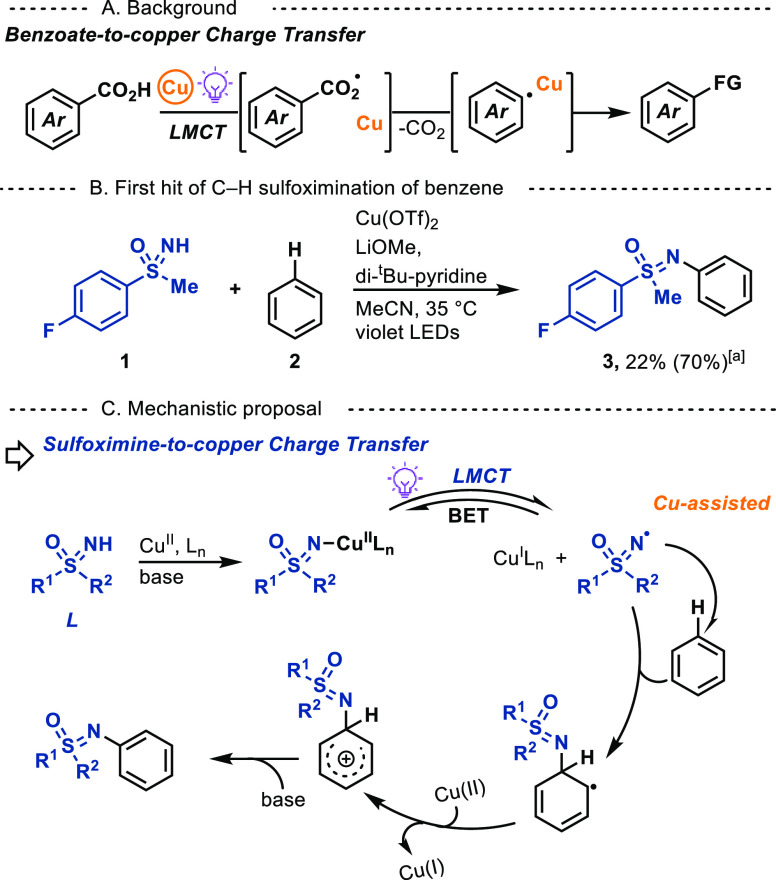
(A) Benzoate-to-copper charge transfer. (B) First hit of C–H-sulfoximination of benzene. Reaction conditions: Cu(OTf)_2_ (2.5 equiv), LiOMe (1.0 equiv), 2,6-di-*tert*-butylpyridine (DTBP, 2.0 equiv), benzene/MeCN (v/v = 1:1, *c* = 25 mM), 15 h violet LEDs irradiation, 35 °C. ^[a]^Cu(OTf)_2_ (2.0 equiv), LiOMe (1.0 equiv), DTBP (2.0 equiv), benzene (100 equiv), MeCN (*c* = 25 mM). (C) Mechanistic proposal of C–H sulfoximination enabled by sulfoximine-to-copper charge transfer.

Sulfoximines are important pharmacophores and their physicochemical and *in vitro* pharmacokinetic parameters can be fine-tuned by N*-*arylation.^9^ To date, *N-*aryl sulfoximines are mainly prepared by transition-metal-catalyzed or -mediated C–N cross-coupling reactions.^10^*NH*-sulfoximines as nucleophiles, are coupled with aryl (pseudo)halides,^11^ aryl boronic acids,^12^ aryl siloxanes,^13^ heteroarenes and polyfluoroarenes,^14^ and others.^15^ Sulfoximination of electron-rich arenes is achieved by oxidizing the arenes to the radical cations, which serve as the electrophiles.^3^ But direct sulfoximination of electron-neutral or deficient arenes has not been established. Conventionally, sulfoximinyl radicals generated from preactivated sulfoximines typically engage in HAT reactions,^16^ or addition reactions to highly reactive radicophiles, such as styrenes, that can outcompete HAT.^17^ No N*-*arylation has been reported via addition reactions of sulfoximinyl radicals to a diverse set of arenes.

While investigating the decarboxylative sulfoximination of benzoic acids, we observed that when benzoate was omitted from the reaction mixture, *N*-aryl sulfoximines were still formed in the presence of benzene, albeit in only 22% yield (Figure 2B).^8g^ Upon further study, we concluded that in the absence of benzoates, sulfoximine-to-copper charge transfer could occur to generate a copper-based sulfoximinyl radical-like species that can add to benzene. An increase of the electrophilicity of the N*-*centered radicals by Lewis acidic copper(II) coordination^18^ could increase the rate of radical addition as opposed to HAT. In the absence of copper salts, sulfoximinyl radical generated from the N*-*bromo sulfoximine **4** under literature-reported reaction conditions with benzene did not result in desired N*-*arylation but only in HAT (Table 1, Supporting Information page S18 for more detail). After radical addition, single electron oxidation by copper(II) would generate a Wheland intermediate, which, upon deprotonation, could afford the *N*-aryl sulfoximine product (Figure 2C). Copper is required for the charge transfer, as well as for modulating the subsequent reactivity for addition chemistry versus HAT.

**Table 1:**
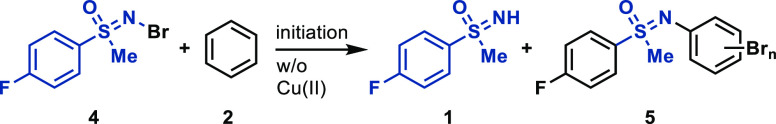
Control Experiments with N–Br Sulfoximine as Radical Precusors^a^

entry	initiation	Cu(II)	Cu(I)	yield of **1**/**5** (%)^e^
1	AIBN, 80 °C^b^	/	/	84/0
2	AIBN, 80 °C^b^	2.0 equiv	/	0/0
3	AIBN, 80 °C^b^	/	2.0 equiv	0/0
4	blue LEDs^c^	/	/	94/0
5	blue LEDs^c^	2.0 equiv	/	0/6
6	blue LEDs^c^	/	2.0 equiv	0/0
7	blue LEDs^c^	/, 2.0 equiv NFTPT	/	78/0
8	violet LEDs^d^	2.0 equiv	/	0/13

a Cu(II): Cu(OTf)_2_, Cu(I): Cu(MeCN)_4_BF_4_, NFTPT: 1-fluoro-2,4,6-trimethylpyridinium tetrafluoroborate.

b **4** (1.0 equiv), 2,2′-azobis(2-methylpropionitrile) (AIBN, 5 mol %), benzene (10 equiv), DCM/MeCN (v/v = 1/1, c = 0.1 M,), 80 °C, 5 h.^1^

c **4** (1.0 equiv), benzene (10 equiv), DCM/MeCN (v/v = 1/1, *c* = 25 mM), blue LEDs (450 nm) irradiation, 35 °C, 15 h.^17b^

d **4** (1.0 equiv), LiOMe (1.0 equiv), 2,6-di-*tert*-butylpyridine (2.0 equiv), benzene (10 equiv), MeCN (*c* = 25 mM), violet LEDs (390 nm) irradiation, 35 °C, 15 h.

e ^19^F NMR yield with 2-fluorotoluene(1.0 equiv) as an internal standard.

We observed a significant absorbance at 370–470 nm in the UV–vis absorption spectra of the mixture of **1** and Cu(OTf)_2_, which we assigned to the LMCT band of sulfoximine-ligated copper(II) species^19^ (Figure 3A). The LMCT band overlaps with the violet LED emission spectrum, consistent with excitation of the corresponding copper(II) species under the reaction conditions (Supporting Information Figure S1). Reduction of Cu(II) to Cu(I) as the C–H sulfoximination reaction progresses is supported by the continuous decrease of the Cu(II)-based *d–d* transition band (550 nm–900 nm)^20^ in the UV–vis spectrum of the reaction mixture upon irradiation (Figure 3B).^8a^ Generation of a Cu(I) species is confirmed by subsequent addition of 2,2′-biquinoline to the irradiated reaction mixture, which results in the purple [Cu^I^(biq)_2_]^+^ complex (λ_max_ = 546 nm, Supporting Information Figure S6).^21^ Formation of the sulfoximinyl radical intermediate is supported by isolation of the cyclization product **8**, when benzene is replaced by *N*,*N*-diphenylmethacrylamide (**7**)^22^ (Figure 3C). Deprotonated *NH*-sulfoximine (*E*_ox_ = 1.86 V vs SCE, Supporting Information Figure S3) is unlikely to be oxidized by Cu(OTf)_2_ (*E*_1/2_ = 0.80 V vs SCE)^8a^ via an intermolecular SET process. The above observations are consistent with formation of a photoinduced LMCT excited state of the Cu(II) sulfoximine complexes, followed by a homolytic Cu–N bond cleavage to generate sulfoximinyl radicals, associated with copper.^4^ Copper-assisted radical addition to arene is supported by observing N-arylated product upon irradiating a mixture of benzene, Cu(OTf)_2_, bases, and sulfoximinyl-containing I(III) reagents, which are known to produce sulfoximinyl radicals^16b^ (Figure 3D). No kinetic isotope effect was observed for the C–H sulfoximination of benzene versus perdeuterated benzene (KIE = 1.0) in a competition experiment shown in Figure 3E. The result is consistent with fast deprotonation of the Wheland intermediate.

**Figure 3:**
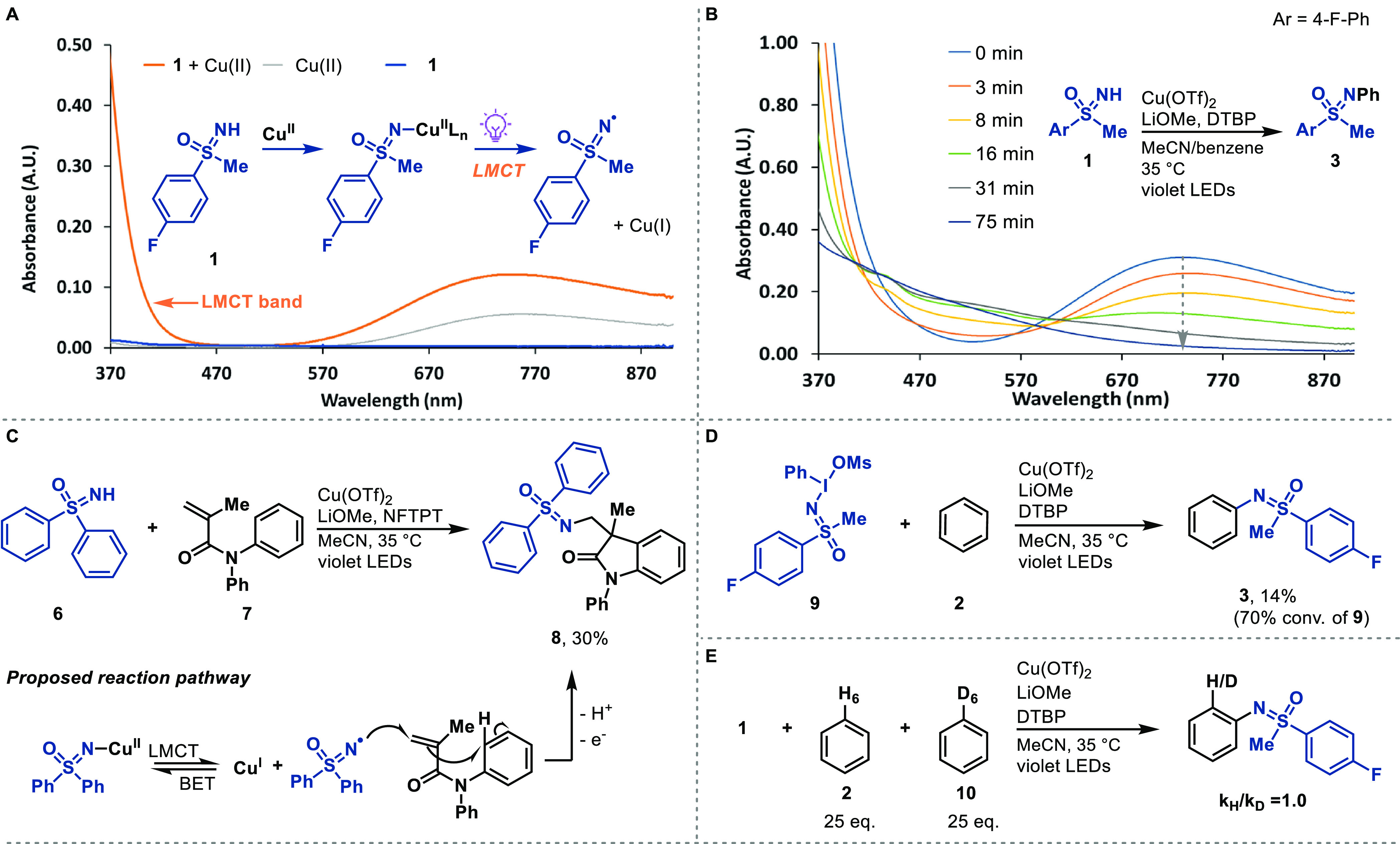
Mechanistic investigations. DTBP: 2,6-di-*tert*-butylpyridine. NFTPT: 1-fluoro-2,4,6-trimethylpyridinium tetrafluoroborate. (A) UV–vis absorption spectra of reaction components. (B) UV–vis absorption spectra upon irradiation of the reaction mixture of C–H sulfoximination of benzene. (C) Sulfoximinyl radical capture experiment. (D) Sulfoximinyl-containing I(III) reagent as the radical precursor. (E) Determination of kinetic isotope effect.

Optimization of the initial reaction conditions with benzene resulted in a more general C–H sulfoximination reaction of other arenes. The reaction was performed with violet LED irradiation of a mixture of *NH*-sulfoximine, LiOMe, 2,6-di-*tert*-butylpyridine (DTBP), Cu(OTf)_2_ and arenes in MeCN, with or without additional oxidant 1-fluoro-2,4,6-trimethylpyridinium tetrafluoroborate (NFTPT) (Figure 4). LiOMe is the optimal inorganic base for *NH*-sulfoximine deprotonation for more efficient coordination to copper.^8g^ Addition of 2.6-di-*tert*-butylpyridine (DTBP) as the ligand is crucial for the desired addition reactivity, presumably due to stabilization of the sulfoximine-copper complex to prevent unproductive back electron transfer.^8f^ Sulfoximination of electron-neutral and -rich arenes works well with Cu(II) added as the terminal oxidant. For electron-deficient arenes, to which the radical addition is slower, HAT of the sulfoximinyl radicals would dominate, which consumes Cu(II) unproductively and leads to a low yield. In such cases, the addition of 1-fluoro-2,4,6-trimethylpyridinium tetrafluoroborate (NFTPT) could increase the yield by reoxidizing the Cu(I) to Cu(II) (Supporting Information Tables S1–S3 for more detail).

**Figure 4:**
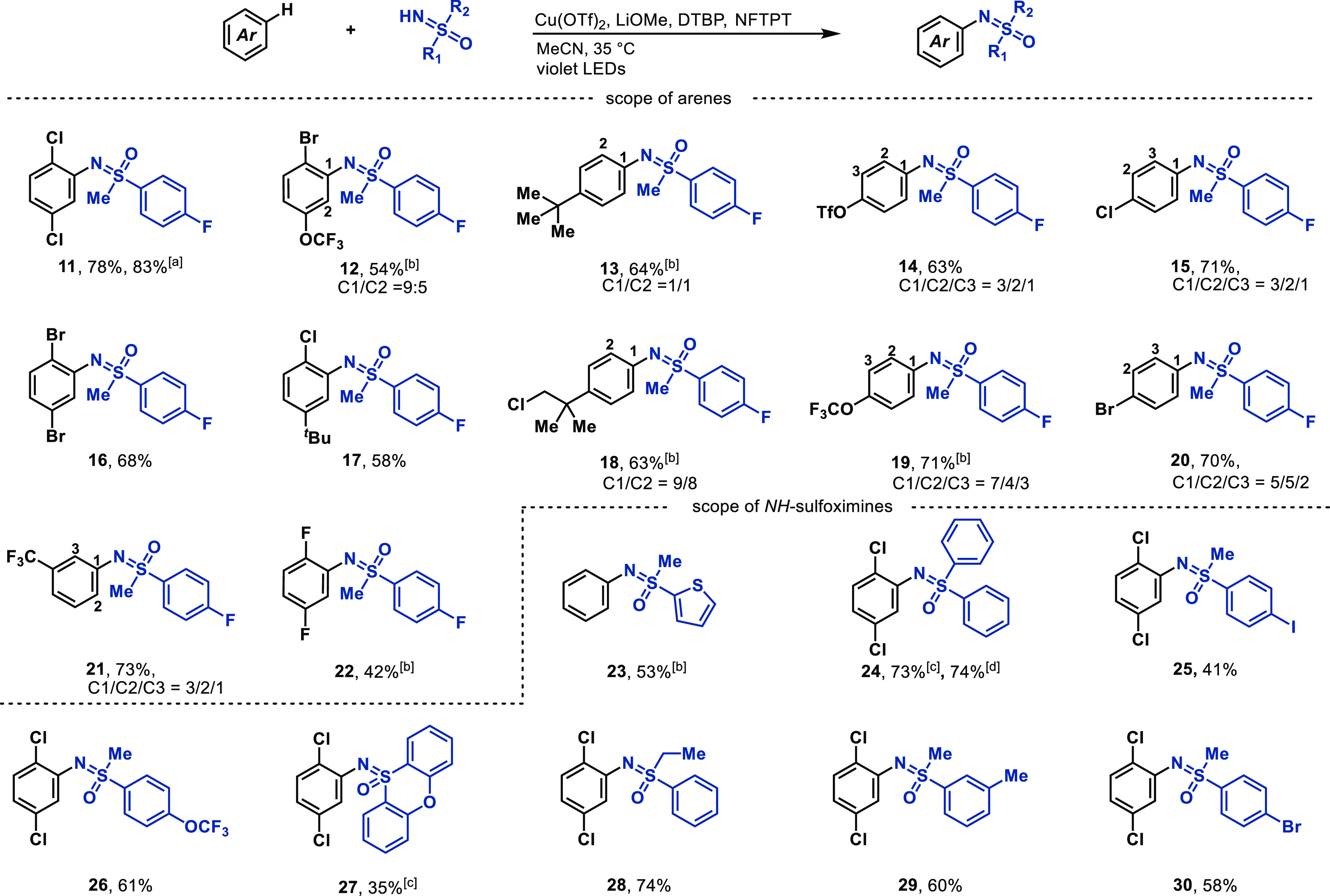
Substrate scope. Standard conditions for C–H sulfoximiantion: sulfoximine (0.20 mmol, 1.0 equiv), arene (50 or 100 equiv), Cu(OTf)_2_ (2.0 equiv), LiOMe (1.0 equiv), 2,6-di-*tert*-butylpyridine (DTBP, 2.0 equiv), 1-fluoro-2,4,6-trimethylpyridinium tetrafluoroborate, (NFTPT, 0.75 equiv), MeCN (*c* = 25 mM), 15 h violet LEDs irradiation, 35 °C. ^[a]^Using recovered arene, ^19^F NMR yield ^[b]^Without NFTPT. ^[c]^Wthout DTBP. ^[d]^Without DTBP, and 1.0 mmol scale.

Under optimized conditions, simple arenes with different electronic structures (**3**, **13**, **21**) all performed well in the C–H sulfoximination (Figure 4). Electron-deficient arenes are typically challenging for radical addition of electrophilic radicals due to the lack of appropriate polarity match,^1^ yet they react well in the Cu-LMCT approach (e.g., **11**, **16**, **21**). In agreement with the proposed radical addition mechanism, and the formation of Wheland intermediates, selectivity was observed for less sterically hindered, electron-rich positions on arenes (**13**, **19**, **21**). In most cases, the resulting constitutional isomers can be separated by column chromatography. Functional groups like (pseudo)halides (**11**, **14**, **16**), which are problematic in low-valent transition metal catalysis, are tolerated. N-aryl sulfoximines with highly functionalized aryl substituents are often not readily accessible from aryl boronic acids or -bromides because they are rarely commercially available. In such cases, C–H functionalization of disubstituted arenes (e.g., **11**, **12**, **16**, **17**, **22**) via the LMCT approach can quickly access such highly substituted compounds. The arene must be added in excess to avoid lower yields. After the reaction, the remaining arene can be recovered in high purity, and can be reused. The C–H sulfoximination cannot tolerate arenes containing weak C–H bonds due to fast HAT. Yet, *NH*-sulfoximines containing benzylic C–H bonds are tolerated (**29**). The scope of *NH*-sulfoximines includes aryl alkyl sulfoximines and more challenging diaryl sulfoximines (**24**, **27**). Electron-rich (**26**) and neutral sulfoximines (**28**, **29**) give higher yields than the electron-deficient ones, possibly due to more effective charge transfer process to copper(II).

Sulfoximine-to-copper charge transfer gives access to sulfoximinyl radicals directly from *NH*-sulfoximines and enables a useful protocol for C–H sulfoximination of arenes. Extension of the LMCT concept to other copper-philic substrates beyond the initially investigated carboxylates establishes the utility of the concept for C–H functionalization reactions to engage in previously elusive transformations.

## Data Availability

The data underlying this study are available in the published article and its Supporting Information.

## Supporting Information Available

Experimental procedures and spectral data (PDF)
