# Supplementary material for: Ligand-to-Copper Charge-Transfer-Enabled C–H Sulfoximination of Arenes
Source: Org Lett. 2023 Feb 3;25(6):1025–9. doi: 10.1021/acs.orglett.3c00256 (PMC9942232; doi:10.1021/acs.orglett.3c00256)
Supplement: Supplementary file 1 — ol3c00256_si_001.pdf [file ol3c00256_si_001.pdf]

SUPPORTING INFORMATION

Ligand-to-Copper Charge Transfer-Enabled C–H Sulfoximination of Arenes

Wanqi Su,[1,2] Peng Xu,[1] Roland Petzold,[1] Jiyao Yan,[1,2] Tobias Ritter[1]*

[1]Max-Planck-Institut für Kohlenforschung, Kaiser-Wilhelm Platz 1, D-45470 Mülheim an der Ruhr, Germany

[2]Institute of Organic Chemistry, RWTH Aachen University, Landoltweg 1, 52074 Aachen, Germany

*E-mail: ritter@kofo.mpg.de

TABLE OF CONTENTS

TABLE OF CONTENTS 1

MATERIALS AND METHODS 8

EXPERIMENTAL DATA 9

General procedure of preparing *NH*-sulfoximines[2] 9

General procedure for aromatic C–H sulfoximination 9

Reaction conditions optimization for aromatic C–H sulfoximination 10

Table S1 Inorganic base optimization 10

Table S2 Pyridine base optimization 10

Table S3 Additional oxidant optimization 11

Table S4 Control experiments 12

Synthesis of *NH*-sulfoximines[2] 13

((4-Fluorophenyl)imino)(methyl)-λ^6-sulfanone (**1**) 13

Iminodiphenyl-λ^6-sulfanone (**6**) 13

Imino(methyl)(phenyl)-λ^6-sulfanone (**S1**) 14

Ethyl(imino)(phenyl)-λ^6-sulfanone (**S4**) 14

Imino(methyl)(m-tolyl)-λ^6-sulfanone (**S5**) 15

Imino(methyl)(4-(trifluoromethoxy)phenyl)-λ^6-sulfanone (**S6**) 15

Imino(methyl)(thiophen-2-yl)-λ^6-sulfanone (**S7**) 16

(4-Bromophenyl)(imino)(methyl)-λ^6-sulfanone (**S8**) 17

10-Imino-10H-10 λ^4-phenoxathiine 10-oxide (**S9**) 17

Imino(4-iodophenyl)(methyl)-λ^6-sulfanone (**S10**) 18

Mechanistic Studies: Copper-LMCT enabled C–H sulfoximination 18

Control experiments using N-bromo sulfoximine[3] as radical precusor 18

Emission spectrum of Kessil PR160L(390nm) LEDs[6] 19

Cyclic voltammograms 20

UV–vis absorption spectrum of reaction components in C–H sulfoximination 21

Photolysis of the mixture of **1**, benzene, DTBP, LiOMe and $Cu(OTf)_2$ 22

Synthesis of *N,N*-diphenylmethacrylamide (**7**)[8] 23

Radical cyclisation experiment: proof of sulfoximinyl radical intermediate 24

C–H sulfoximination of sulfoximinyl-containing I(III) reagent[9] 25

Kinetic isotope effect (KIE) determination by competition experiment 26

Copper-LMCT enabled C–H sulfoximination: arene scope 28

(4-Fluorophenyl)(methyl)(phenylimino)-λ^6-sulfanone (**3**) 28

((2,5-Dichlorophenyl)imino)(4-fluorophenyl)(methyl)-λ^6-sulfanone (**11**) 28
Synthesis of ((2,5-dichlorophenyl)imino)(4-fluorophenyl)(methyl)-λ^6-sulfanone (**11**) using recovered arene 29
(Bromo-(trifluoromethoxy)phenyl)imino)(4-fluorophenyl)(methyl)-λ^6-sulfanone (**12**) 30
((Tert-butyl)phenyl)imino)(4-fluorophenyl)(methyl)-λ^6-sulfanone (**13**) 31
(((4-Fluorophenyl)(methyl)(oxo)-λ^6-sulfaneylidene)amino)phenyl trifluoromethanesulfonate (**14**) 32
((Chlorophenyl)imino)(4-fluorophenyl)(methyl)-λ^6-sulfanone (**15**) 34
((2,5-Dibromophenyl)imino)(4-fluorophenyl)(methyl)-λ^6-sulfanone (**16**) 36
((5-(Tert-butyl)-2-chlorophenyl)imino)(4-fluorophenyl)(methyl)-λ^6-sulfanone (**17**) 37
(((1-Chloro-2-methylpropan-2-yl)phenyl)imino)(4-fluorophenyl)(methyl)-λ^6-sulfanone (**18**) 37
(4-Fluorophenyl)(methyl)((trifluoromethoxy)phenyl)imino)-λ^6-sulfanone (**19**) 38
((Bromophenyl)imino)(4-fluorophenyl)(methyl)-λ^6-sulfanone (**20**) 40
(4-Fluorophenyl)(methyl)((trifluoromethyl)phenyl)imino)-λ^6-sulfanone (**21**) 41
((2,5-Difluorophenyl)imino)(4-fluorophenyl)(methyl)-λ^6-sulfanone (**22**) 43

Copper-LMCT enabled C–H sulfoximination: *NH*-sulfoximine scope 44
Methyl(phenylimino)(thiophen-2-yl)-λ^6-sulfanone (**23**) 44
((2,5-Dichlorophenyl)imino)diphenyl-λ^6-sulfanone (**24**) 44
1 mmol scale synthesis of ((2,5-dichlorophenyl)imino)diphenyl-λ^6-sulfanone (**24**) 45
Recovery of the unreacted arene 1,4-dichlorobenzene 46
((2,5-Dichlorophenyl)imino)(4-iodophenyl)(methyl)-λ^6-sulfanone (**25**) 48
((2,5-Dichlorophenyl)imino)(methyl)(4-(trifluoromethoxy)phenyl)-λ^6-sulfanone (**26**) 48
10-((2,5-Dichlorophenyl)imino)-10*H*-10-λ^4--phenoxathiine 10-oxide (**27**) 49
((2,5-Dichlorophenyl)imino)(ethyl)(phenyl)-λ^6-sulfanone (**28**) 50
((2,5-Dichlorophenyl)imino)(methyl)(m-tolyl)-λ^6-sulfanone (**29**) 51
(4-Bromophenyl)((2,5-dichlorophenyl)imino)(methyl)-λ^6-sulfanone (**30**) 52

DFT calculation 53
Method 53
Bond disscociation energy of different *NH*-sulfoximines 53
Cartesian coordinates of the optimized species 53

SPECTROSCOPIC DATA 57
^{1}H NMR of ((4-fluorophenyl)imino)(methyl)-λ^6-sulfanone (**1**) 57
^{13}C NMR of ((4-fluorophenyl)imino)(methyl)-λ^6-sulfanone (**1**) 58
^{19}F NMR of ((4-fluorophenyl)imino)(methyl)-λ^6-sulfanone (**1**) 59
^{1}H NMR of iminodiphenyl-λ^6-sulfanone (**6**) 60
^{13}C NMR of iminodiphenyl-λ^6-sulfanone (**6**) 61

^{1}H NMR of imino(methyl)(phenyl)-λ6-sulfanone (**S1**)....62

^{13}C NMR of imino(methyl)(phenyl)-λ^{6}-sulfanone (**S1**)....63

^{1}H NMR of ethyl(imino)(phenyl)-λ^{6}-sulfanone (**S4**)....64

^{13}C NMR of ethyl(imino)(phenyl)-λ^{6}-sulfanone (**S4**)....65

^{1}H NMR of imino(methyl)(m-tolyl)-λ6-sulfanone (**S5**)....66

^{13}C NMR of imino(methyl)(m-tolyl)-λ^{6}-sulfanone (**S5**)....67

^{1}H NMR of imino(methyl)(4-(trifluoromethoxy)phenyl)-λ^{6}-sulfanone (**S6**)....68

^{13}C NMR of imino(methyl)(4-(trifluoromethoxy)phenyl)-λ^{6}-sulfanone (**S6**)....69

^{19}F NMR of imino(methyl)(4-(trifluoromethoxy)phenyl)-λ^{6}-sulfanone (**S6**)....70

^{1}H NMR of imino(methyl)(thiophen-2-yl)-λ^{6}-sulfanone (**S7**)....71

^{13}C NMR of imino(methyl)(thiophen-2-yl)-λ^{6}-sulfanone (**S7**)....72

^{1}H NMR of (4-bromophenyl)(imino)(methyl)-λ^{6}-sulfanone (**S8**)....73

^{13}C NMR of (4-bromophenyl)(imino)(methyl)-λ^{6}-sulfanone (**S8**)....74

^{1}H NMR of 10-imino-10H-10 λ4-phenoxathiine 10-oxide (**S9**)....75

^{13}C NMR of 10-imino-10H-10 λ^{4}-phenoxathiine 10-oxide (**S9**)....76

^{1}H NMR of imino(4-iodophenyl)(methyl)-λ^{6}-sulfanone (**S10**)....77

^{13}C NMR of imino(4-iodophenyl)(methyl)-λ^{6}-sulfanone (**S10**)....78

^{1}H NMR of N,N-diphenylmethacrylamide (**7**)....79

^{13}C NMR of *N,N*-diphenylmethacrylamide (**7**)....80

^{1}H NMR of 3-methyl-3-(((oxodiphenyl-λ^{6}-sulfaneylidene)amino)methyl)-1-phenylindolin-2-one (**8**)....81

^{13}C NMR of 3-methyl-3-(((oxodiphenyl-λ^{6}-sulfaneylidene)amino)methyl)-1-phenylindolin-2-one (**8**)....82

^{1}H NMR of sulfoximinyl containing I(III) reagent **9**....83

^{19}F NMR of sulfoximinyl containing I(III) reagent **9**....84

^{1}H NMR of (4-fluorophenyl)(methyl)(phenylimino)-λ^{6}-sulfanone (**3**)....85

^{13}C NMR of (4-fluorophenyl)(methyl)(phenylimino)-λ^{6}-sulfanone (**3**)....86

^{19}F NMR of (4-fluorophenyl)(methyl)(phenylimino)-λ^{6}-sulfanone (**3**)....87

^{1}H NMR of ((2,5-dichlorophenyl)imino)(4-fluorophenyl)(methyl)-λ^{6}-sulfanone (**11**)....88

^{13}C NMR of ((2,5-dichlorophenyl)imino)(4-fluorophenyl)(methyl)-λ^{6}-sulfanone (**11**)....89

^{19}F NMR of ((2,5-dichlorophenyl)imino)(4-fluorophenyl)(methyl)-λ^{6}-sulfanone (**11**)....90

^{1}H NMR of ((2-bromo-5-(trifluoromethoxy)phenyl)imino)(4-fluorophenyl)(methyl)-λ^{6}-sulfanone (**12-C1**) ... 91

^{13}C NMR of ((2-bromo-5-(trifluoromethoxy)phenyl)imino)(4-fluorophenyl)(methyl)-λ^{6}-sulfanone (**12-C1**) .. 92

^{19}F NMR of ((2-bromo-5-(trifluoromethoxy)phenyl)imino)(4-fluorophenyl)(methyl)-λ^{6}-sulfanone (**12-C1**) .. 93

^{1}H NMR of ((5-bromo-2-(trifluoromethoxy)phenyl)imino)(4-fluorophenyl)(methyl)-λ^{6}-sulfanone (**12-C2**) ... 94

^{13}C NMR of ((5-bromo-2-(trifluoromethoxy)phenyl)imino)(4-fluorophenyl)(methyl)-λ^{6}-sulfanone (**12-C2**) .. 95

^{19}F NMR of ((5-bromo-2-(trifluoromethoxy)phenyl)imino)(4-fluorophenyl)(methyl)-λ^{6}-sulfanone (**12-C2**) .. 96

^{1}H NMR of ((tert-butyl)phenyl)imino)(4-fluorophenyl)(methyl)-λ^{6}-sulfanone (**13**) ... 97

^{13}C NMR of ((tert-butyl)phenyl)imino)(4-fluorophenyl)(methyl)-λ^{6}-sulfanone (**13**) ... 98

^{19}F NMR of ((tert-butyl)phenyl)imino)(4-fluorophenyl)(methyl)-λ^{6}-sulfanone (**13**) ... 99

^{1}H NMR of 4-(((4-fluorophenyl)(methyl)(oxo)-λ^{6}-sulfaneylidene)amino)phenyl trifluoromethanesulfonate (**14-C1**) ... 100

^{13}C NMR of 4-(((4-fluorophenyl)(methyl)(oxo)-λ^{6}-sulfaneylidene)amino)phenyl trifluoromethanesulfonate (**14-C1**) ... 101

^{19}F NMR of 4-(((4-fluorophenyl)(methyl)(oxo)-λ^{6}-sulfaneylidene)amino)phenyl trifluoromethanesulfonate (**14-C1**) ... 102

^{1}H NMR of 3-(((4-fluorophenyl)(methyl)(oxo)-λ^{6}-sulfaneylidene)amino)phenyl trifluoromethanesulfonate (**14-C2**) ... 103

^{13}C NMR of 3-(((4-fluorophenyl)(methyl)(oxo)-λ^{6}-sulfaneylidene)amino)phenyl trifluoromethanesulfonate (**14-C2**) ... 104

^{19}F NMR of 3-(((4-fluorophenyl)(methyl)(oxo)-λ^{6}-sulfaneylidene)amino)phenyl trifluoromethanesulfonate (**14-C2**) ... 105

^{1}H NMR of 2-(((4-fluorophenyl)(methyl)(oxo)-λ^{6}-sulfaneylidene)amino)phenyl trifluoromethanesulfonate (**14-C3**) ... 106

^{13}C NMR of 2-(((4-fluorophenyl)(methyl)(oxo)-λ^{6}-sulfaneylidene)amino)phenyl trifluoromethanesulfonate (**14-C3**) ... 107

^{19}F NMR of 2-(((4-fluorophenyl)(methyl)(oxo)-λ^{6}-sulfaneylidene)amino)phenyl trifluoromethanesulfonate (**14-C3**) ... 108

^{1}H NMR of ((4-chlorophenyl)imino)(4-fluorophenyl)(methyl)-λ^{6}-sulfanone (**15-C1**) ... 109

^{13}C NMR of ((4-chlorophenyl)imino)(4-fluorophenyl)(methyl)-λ^{6}-sulfanone (**15-C1**) ... 110

^{19}F NMR of ((4-chlorophenyl)imino)(4-fluorophenyl)(methyl)-λ^{6}-sulfanone (**15-C1**) ... 111

^{1}H NMR of ((2-chlorophenyl)imino)(4-fluorophenyl)(methyl)-λ^{6}-sulfanone (**15-C2**) ... 112

^{13}C NMR of ((2-chlorophenyl)imino)(4-fluorophenyl)(methyl)-λ^6-sulfanone (**15-C2**) 113

^{19}F NMR of ((2-chlorophenyl)imino)(4-fluorophenyl)(methyl)-λ^6-sulfanone (**15-C2**) 114

^{1}H NMR of ((3-chlorophenyl)imino)(4-fluorophenyl)(methyl)-λ^6-sulfanone (**15-C3**) 115

^{13}C NMR of ((3-chlorophenyl)imino)(4-fluorophenyl)(methyl)-λ^6-sulfanone (**15-C3**) 116

^{19}F NMR of ((3-chlorophenyl)imino)(4-fluorophenyl)(methyl)-λ^6-sulfanone (**15-C3**) 117

^{1}H NMR of ((2,5-dibromophenyl)imino)(4-fluorophenyl)(methyl)-λ^6-sulfanone (**16**) 118

^{13}C NMR of ((2,5-dibromophenyl)imino)(4-fluorophenyl)(methyl)-λ^6-sulfanone (**16**) 119

^{19}F NMR of ((2,5-dibromophenyl)imino)(4-fluorophenyl)(methyl)-λ^6-sulfanone (**16**) 120

^{1}H NMR of ((5-(tert-butyl)-2-chlorophenyl)imino)(4-fluorophenyl)(methyl)-λ^6-sulfanone (**17**) 121

^{13}C NMR of ((5-(tert-butyl)-2-chlorophenyl)imino)(4-fluorophenyl)(methyl)-λ^6-sulfanone (**17**) 122

^{19}F NMR of ((5-(tert-butyl)-2-chlorophenyl)imino)(4-fluorophenyl)(methyl)-λ^6-sulfanone (**17**) 123

^{1}H NMR of (((1-chloro-2-methylpropan-2-yl)phenyl)imino)(4-fluorophenyl)(methyl)-λ^6-sulfanone (**18**) 124

^{13}C NMR of (((1-chloro-2-methylpropan-2-yl)phenyl)imino)(4-fluorophenyl)(methyl)-λ^6-sulfanone (**18**) ... 125

^{19}F NMR of (((1-chloro-2-methylpropan-2-yl)phenyl)imino)(4-fluorophenyl)(methyl)-λ^6-sulfanone (**18**) ... 126

^{1}H NMR of (4-fluorophenyl)(methyl)((4-(trifluoromethoxy)phenyl)imino)-λ^6-sulfanone (**19-C1**) 127

^{13}C NMR of (4-fluorophenyl)(methyl)((4-(trifluoromethoxy)phenyl)imino)-λ^6-sulfanone (**19-C1**) 128

^{19}F NMR of (4-fluorophenyl)(methyl)((4-(trifluoromethoxy)phenyl)imino)-λ^6-sulfanone (**19-C1**) 129

^{1}H NMR of (4-fluorophenyl)(methyl)((3-(trifluoromethoxy)phenyl)imino)-λ^6-sulfanone (**19-C2**) 130

^{13}C NMR of (4-fluorophenyl)(methyl)((3-(trifluoromethoxy)phenyl)imino)-λ^6-sulfanone (**19-C2**) 131

^{19}F NMR of (4-fluorophenyl)(methyl)((3-(trifluoromethoxy)phenyl)imino)-λ^6-sulfanone (**19-C2**) 132

^{1}H NMR of (4-fluorophenyl)(methyl)((2-(trifluoromethoxy)phenyl)imino)-λ^6-sulfanone (**19-C3**) 133

^{13}C NMR of (4-fluorophenyl)(methyl)((2-(trifluoromethoxy)phenyl)imino)-λ^6-sulfanone (**19-C3**) 134

^{19}F NMR of (4-fluorophenyl)(methyl)((2-(trifluoromethoxy)phenyl)imino)-λ^6-sulfanone (**19-C3**) 135

^{1}H NMR of ((4-bromophenyl)imino)(4-fluorophenyl)(methyl)-λ^6-sulfanone (**20-C1**) 136

^{13}C NMR of ((4-bromophenyl)imino)(4-fluorophenyl)(methyl)-λ^6-sulfanone (**20-C1**) 137

^{19}F NMR of ((4-bromophenyl)imino)(4-fluorophenyl)(methyl)-λ^6-sulfanone (**20-C1**) 138

^{1}H NMR of ((2-bromophenyl)imino)(4-fluorophenyl)(methyl)-λ^6-sulfanone (**20-C2**) 139

^{13}C NMR of ((2-bromophenyl)imino)(4-fluorophenyl)(methyl)-λ^6-sulfanone (**20-C2**) 140

^{19}F NMR of ((2-bromophenyl)imino)(4-fluorophenyl)(methyl)-λ^6-sulfanone (**20-C2**) 141

^{1}H NMR of ((3-bromophenyl)imino)(4-fluorophenyl)(methyl)-λ^{6}-sulfanone (**20-C3**) 142
^{13}C NMR of ((3-bromophenyl)imino)(4-fluorophenyl)(methyl)-λ^{6}-sulfanone (**20-C3**) 143
^{19}F NMR of ((3-bromophenyl)imino)(4-fluorophenyl)(methyl)-λ^{6}-sulfanone (**20-C3**) 144
^{1}H NMR of (4-fluorophenyl)(methyl)((3-(trifluoromethyl)phenyl)imino)-λ^{6}-sulfanone (**21-C1**) 145
^{13}C NMR of (4-fluorophenyl)(methyl)((3-(trifluoromethyl)phenyl)imino)-λ^{6}-sulfanone (**21-C1**) 146
^{19}F NMR of (4-fluorophenyl)(methyl)((3-(trifluoromethyl)phenyl)imino)-λ^{6}-sulfanone (**21-C1**) 147
^{1}H NMR of (4-fluorophenyl)(methyl)((4-(trifluoromethyl)phenyl)imino)-λ^{6}-sulfanone (**21-C2**) 148
^{13}C NMR of (4-fluorophenyl)(methyl)((4-(trifluoromethyl)phenyl)imino)-λ^{6}-sulfanone (**21-C2**) 149
^{19}F NMR of (4-fluorophenyl)(methyl)((4-(trifluoromethyl)phenyl)imino)-λ^{6}-sulfanone (**21-C2**) 150
^{1}H NMR of (4-fluorophenyl)(methyl)((2-(trifluoromethyl)phenyl)imino)-λ^{6}-sulfanone (**21-C3**) 151
^{13}C NMR of (4-fluorophenyl)(methyl)((2-(trifluoromethyl)phenyl)imino)-λ^{6}-sulfanone (**21-C3**) 152
^{19}F NMR of (4-fluorophenyl)(methyl)((2-(trifluoromethyl)phenyl)imino)-λ^{6}-sulfanone (**21-C3**) 153
^{1}H NMR of ((2,5-difluorophenyl)imino)(4-fluorophenyl)(methyl)-λ^{6}-sulfanone (**22**) 154
^{13}C NMR of ((2,5-difluorophenyl)imino)(4-fluorophenyl)(methyl)-λ^{6}-sulfanone (**22**) 155
^{19}F NMR of ((2,5-difluorophenyl)imino)(4-fluorophenyl)(methyl)-λ^{6}-sulfanone (**22**) 156
^{1}H NMR of methyl(phenylimino)(thiophen-2-yl)-λ^{6}-sulfanone (**23**) 157
^{13}C NMR of methyl(phenylimino)(thiophen-2-yl)-λ^{6}-sulfanone (**23**) 158
^{1}H NMR of ((2,5-dichlorophenyl)imino)diphenyl-λ^{6}-sulfanone (**24**) 159
^{13}C NMR of ((2,5-dichlorophenyl)imino)diphenyl-λ^{6}-sulfanone (**24**) 160
^{1}H NMR of ((2,5-dichlorophenyl)imino)(4-iodophenyl)(methyl)-λ^{6}-sulfanone (**25**) 161
^{13}C NMR of ((2,5-dichlorophenyl)imino)(4-iodophenyl)(methyl)-λ^{6}-sulfanone (**25**) 162
^{1}H NMR of ((2,5-dichlorophenyl)imino)(methyl)(4-(trifluoromethoxy)phenyl)-λ^{6}-sulfanone (**26**) 163
^{13}C NMR of ((2,5-dichlorophenyl)imino)(methyl)(4-(trifluoromethoxy)phenyl)-λ^{6}-sulfanone (**26**) 164
^{19}F NMR of ((2,5-dichlorophenyl)imino)(methyl)(4-(trifluoromethoxy)phenyl)-λ^{6}-sulfanone (**26**) 165
^{1}H NMR of 10-((2,5-dichlorophenyl)imino)-10*H*-10-λ^{4}--phenoxathiine 10-oxide (**27**) 166
^{13}C NMR of 10-((2,5-dichlorophenyl)imino)-10*H*-10-λ^{4}--phenoxathiine 10-oxide (**27**) 167
^{1}H NMR of ((2,5-dichlorophenyl)imino)(ethyl)(phenyl)-λ^{6}-sulfanone (**28**) 168
^{13}C NMR of ((2,5-dichlorophenyl)imino)(ethyl)(phenyl)-λ^{6}-sulfanone (**28**) 169
^{1}H NMR of ((2,5-dichlorophenyl)imino)(methyl)(m-tolyl)-λ^{6}-sulfanone (**29**) 170

^{13}C NMR of ((2,5-dichlorophenyl)imino)(methyl)(m-tolyl)-λ^{6}-sulfanone (**29**) 171

^{1}H NMR of (4-Bromophenyl)((2,5-dichlorophenyl)imino)(methyl)-λ^{6}-sulfanone (**30**) 172

^{13}C NMR of (4-Bromophenyl)((2,5-dichlorophenyl)imino)(methyl)-λ^{6}-sulfanone (**30**) 173

REFERENCES 174

MATERIALS AND METHODS

All air- and moisture-insensitive reactions were carried out under an ambient atmosphere and monitored by thin-layer chromatography (TLC). All air- and moisture-sensitive manipulations were performed using standard *Schlenk* and glove-box techniques under an atmosphere of nitrogen. Concentration under reduced pressure was performed by rotary evaporation at 25–40 °C at an appropriate pressure, unless otherwise stated. Purified compounds were further dried under high vacuum (0.008–0.5 Torr). Yields refer to purified and spectroscopically pure compounds, unless otherwise stated.

Solvents

Anhydrous acetonitrile, DCM, THF, and toluene were obtained from Phoenix Solvent Drying Systems. All deuterated solvents were purchased from Euriso-Top.

Chromatography

Thin layer chromatography (TLC) was performed using EMD TLC silica gel 60 F254 plates pre-coated with 250 μm thickness silica gel and visualized by fluorescence quenching under UV light or phosphomolybdic acid stain. Preparative TLC was performed using pre-coated TLC plates SIL G-100 UV_{254} (Layer: 1.00 mm silica gel 60 with fluorescent indicator UV_{254}).

Spectroscopy and Instruments

NMR spectra were recorded on a Bruker Ascend™ 500 spectrometer operating at 500 MHz, 471 MHz, and 126 MHz, for ^{1}H, ^{19}F, and ^{13}C acquisitions, respectively; or a Bruker AV600 spectrometer operating at 600 MHz, 565 MHz, 92 MHz, and 151 MHz, for ^{1}H, ^{19}F, D and ^{13}C acquisitions, respectively. Chemical shifts are reported in ppm with the solvent residual peak as the internal standard. For ^{1}H NMR: $CDCl_3$, δ 7.26; CD_2Cl_2, δ 5.32. For ^{13}C NMR: $CDCl_3$, δ 77.16; CD_2Cl_2, δ 53.84.[1] Data is reported as follows: s = singlet, d = doublet, t = triplet, q = quartet, qd = quartet of doublets, m = multiplet; coupling constants in Hz; integration.

Starting materials

All substrates and materials were used as received from commercial suppliers, unless otherwise stated. $Cu(OTf)_2$ purchased from TCI was dried in a 150 °C oven for 2 hours and stored in a glovebox. $Cu(MeCN)_4BF_4$, 1-fluoro-2,4,6-trimethylpyridinium tetrafluoroborate were purchased from TCI and stored in a glovebox. *NH*-sulfoximines were purchased from commercial suppliers or prepared according to the literatures[2]. *N*-bromo sulfoximine,[3] *N,N*-diphenylmethacrylamide,[8] and sulfoximinyl-containg I(III) reagent[9] were synthesized according to the literatures.

EXPERIMENTAL DATA

General procedure of preparing *NH*-sulfoximines[2]

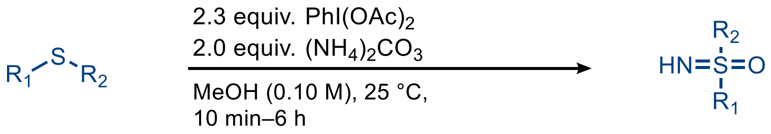

Under an ambient atmosphere, to a stirred solution of sulfide (1.00 mmol) in MeOH (10.0 mL, c = 0.10 M) was added $(NH_4)_2CO_3$ (192 mg, 2.00 mmol, 2.00 equiv.). Subsequently, $PhI(OAc)_2$ (741 mg, 2.30 mmol, 2.30 equiv.) was added, then the reaction mixture was stirred at 25 °C for 10 min to 6 h and monitored by TLC. The solvent was removed under reduced pressure, and the product was purified by flash column chromatography on silica gel eluting with DCM/MeOH to give the desired *NH*-sulfoximine.

General procedure for aromatic C–H sulfoximination

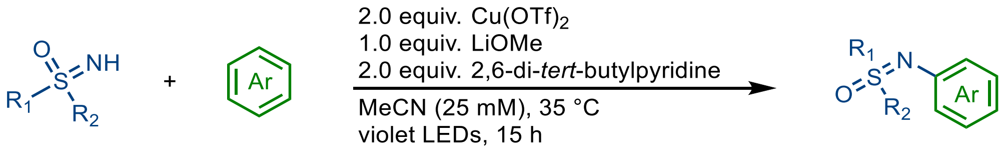

Under an ambient atmosphere, a 10 mL borosilicate vial equipped with a magnetic stir bar was charged with *NH*-sulfoximine (0.200 mmol, 1.00 equiv.). Under nitrogen atmosphere, $Cu(OTf)_2$ (144 mg, 0.400 mmol, 2.00 equiv.), LiOMe (0.200 mmol, 1.00 equiv.), and anhydrous MeCN (8.0 mL, c = 25 mM) were added into the vial, resulting in a blue suspension. The reaction mixture was stirred without irradiation for 30 min at 25 °C. Subsequently, arene (10.0 mmol, 50.0 equiv. or 20.0 mmol, 100 equiv.), and 2,6-di-*tert*-butylpyridine (90.0 µL, 76.5 mg, 0.400 mmol, 2.00 equiv.) were added into the vial, resulting in a green suspension. The vial was sealed with a Teflon cap. The sealed vial was placed 5 cm away from two violet LEDs (Kessil PR160L-390 nm LEDs). The reaction mixture was stirred at a speed of 1000 rpm and irradiated for 15 h while maintaining the temperature at approximately 35 °C through cooling with a fan. After irradiation, volatiles were removed from the reaction mixture under reduced pressure. DCM (15 mL) was added to the residue, and the precipitate was removed by filtration through a glass frit. The filtrate was concentrated under reduced pressure, and the residue was purified by flash column chromatography on silica gel. When necessary, further purification was accomplished by either preparative thin layer chromatography or additional flash column chromatography on silica gel.

NOTE: UV light is harmful to human health, and the operator should wear UV light safety glasses when setting up the reaction. For the duration of the reaction, the irradiation setup was covered by aluminum foil to shield the UV light. The reaction is O_2 sensitive. Schlenk technique was used to avoid air. For simplicity, we have opted to execute the transformation for most compounds by using a glovebox. Control experiments showed that yields were within error of measurement whether the reaction was carried out using a glovebox or Schlenk technique.

Reaction conditions optimization for aromatic C–H sulfoximination

Table S1 Inorganic base optimization

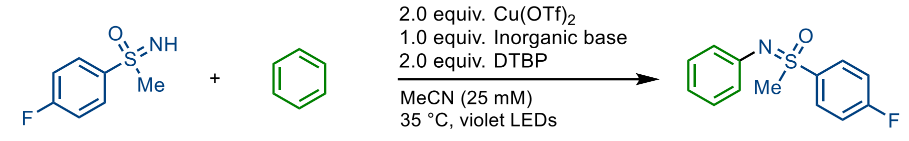

base source	Yield (**3**, %)[a]	base source	Yield (**3**, %)[a]
Na_2CO_3	42	Li_3PO_4	41
Na_3PO_4	56	**LiOH**	69
K_2CO_3	41	**LiO^{t}Bu**	**66**
K_3PO_4	52	**LiOMe**	**70**
Cs_2CO_3	47	1.5 equiv. LiOMe	61
Li_2CO_3	40	2.0 equiv. LiOMe	56

DTBP: 2,6-di-*tert*-butylpyridine. [a] ^{19}F NMR yield with 2-fluorotoluene(1.0 equiv.) as an internal standard.

Table S2 Pyridine base optimization

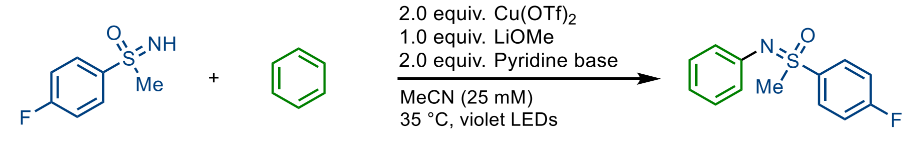

base source	Yield (**3**, %)[a]	base source	Yield (**3**, %)[a]
2,6-di-tert-butylpyridine (DTBP)	**70**	2-fluoro-6-trifluoromethylpyridine	0
2,6-difluoropyridine	0	4-chloro-2,6-dimethylpyridine	23

2,6-lutidine	53	pyridine	15
2,4,6-trimethylpyridine	**74**	2,6-di-tert-butyl-4-methylpyridine	15

DTBP: 2,6-di-*tert*-butylpyridine. [a] ^{19}F NMR yield with 2-fluorotoluene (1.0 equiv.) as an internal standard.

Table S3 Additional oxidant optimization

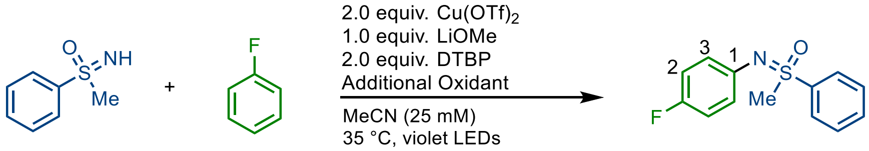

S1 0.05 mmol, 1.00 equiv. **S2** 100 equiv. **S3** (**C1**+**C2**+**C3**)

Oxidant	Yield (**S3**, %)[a]	Oxidant (0.75 equiv.)	Yield (**S3**, %)[a]
No additional oxidant	47	1-fluoropyridinium triflate	39
0.25 equiv. 1-fluoro-2,4,6-trimethylpyridinium tetrafluoroborate (NFTPT)	60	2,6-dichloro-1-fluorpyridinium tetrafluoroborate	32
0.50 equiv. NFTPT	68	N-fluorobenzenesulfonimide(NFSI)	16
**0.75 equiv. NFTPT**	**74**	Selectfluor	19
1.0 equiv. NFTPT	60	/	/
1.5 equiv. NFTPT	48	/	/

Note: When arenes were electron-deficient, relatively low yields were observed, which presumably resulted from the slower rate of radical addition compared to the rate of HAT. Sulfoximinyl radicals were quenched by HAT whereas copper (II) was consumed unproductively. Addition of extra oxidants to reoxidize Cu(I) to Cu(II) increased the yield in some cases.

DTBP: 2,6-di-*tert*-butylpyridine. [a] ^{19}F NMR yield with 2-fluorotoluene(1.0 equiv.) as an internal standard.

Table S4 Control experiments

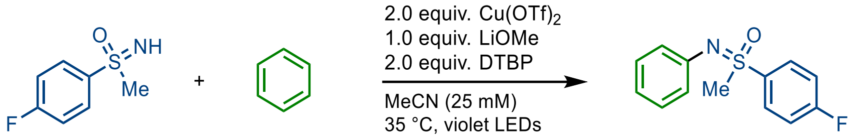

1 0.05 mmol, 1.00 equiv. **2** 100 equiv. **3**

derivation	Yield (**3**, %)[a]
none	70
100 °C heating instead of irradination	0
blue LEDs instead of violet LEDs	8
no $Cu(OTf)_2$	0, no conversion of **1**
no LiOMe and no DTBP	10
no LiOMe but with DTBP (2.0 equiv.)	44
no DTBP but with LiOMe (1.0 equiv.)	0
no DTBP but with LiOMe (3.0 equiv.)	16
2.0 equiv. TEMPO as additive	10
air atmosphere instead of nitrogen atmosphere	38
$Cu(OTf)_2$ (0.3 equiv.) and 1-fluoro-2,4,6-trimethylpyridinium tetrafluoroborate (2.0 equiv.) instead of $Cu(OTf)_2$ (2.0 equiv.)	12
$Cu(OTf)_2$ (0.2 equiv.) and 1-fluoro-2,4,6-trimethylpyridinium tetrafluoroborate (3.0 equiv.) instead of $Cu(OTf)_2$ (2.0 equiv.)	16
$Cu(OTf)_2$ (0.2 equiv.) and $K_2S_2O_8$ (3.0 equiv.) instead of $Cu(OTf)_2$ (2.0 equiv.)	0, 70% conversion of **1**
$Cu(OTf)_2$ (0.2 equiv.) and N-Fluorobenzenesulfonimide (NFSI, 3.0 equiv.) instead of $Cu(OTf)_2$ (2.0 equiv.)	6%
$Cu(OTf)_2$ (0.2 equiv.) and Di-tert-butylperoxide (3.0 equiv.) instead of $Cu(OTf)_2$ (2.0 equiv.)	25%

Benzene (5.0 equiv. instead of 100 equiv.)	25
Benzene (50.0 equiv. instead of 100 equiv.)	40

DTBP: 2,6-di-*tert*-butylpyridine. [a] ^{19}F NMR yield with 2-fluorotoluene(1.0 equiv.) as an internal standard.

Synthesis of *NH*-sulfoximines[2]

((4-Fluorophenyl)imino)(methyl)-λ^6-sulfanone (1)

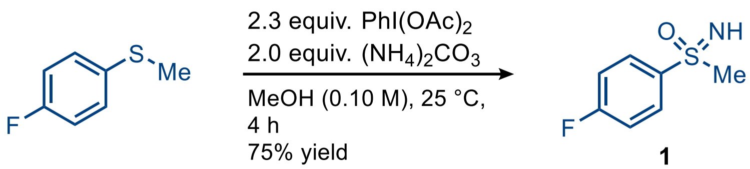

1

Under an ambient atmosphere, to a stirred solution of (4-fluorophenyl)(methyl)sulfide (142 mg, 1.00 mmol, 1.00 equiv) in MeOH (10.0 mL, *c* = 0.10 M) was added $(NH_4)_2CO_3$ (192 mg, 2.00 mmol, 2.00 equiv.). Subsequently, $PhI(OAc)_2$ (741 mg, 2.30 mmol, 2.30 equiv.) was added, then the reaction mixture was stirred at 25 °C for 4 h. The solvent was removed under reduced pressure, and the product was purified by flash column chromatography on silica gel eluting with MeOH/DCM (1/50 v/v) to yield ((4-fluorophenyl)imino)(methyl)-λ^6-sulfanone (**1**) (130 mg, 751 µmol, 75%) as a colorless solid.

R*f* = 0.22 (MeOH/DCM = 1:50 v/v).

NMR Spectroscopy:

^{1}H NMR (500 MHz, CD_2Cl_2, 298 K, δ): 8.01 (dd, *J* = 8.9, 5.2 Hz, 2H), 7.23 (t, *J* = 8.7 Hz, 2H), 3.06 (s, 3H), 2.71 (s, 1H) ppm.

^{13}C NMR (126 MHz, CD_2Cl_2, 298 K, δ): 165.8, 140.3, 130.9, 116.6, 46.8 ppm.

^{19}F NMR (471 MHz, CD_2Cl_2, 298 K, δ): –106.6 (m) ppm.

HRMS-EI (m/z) calculated for $C_7H_8NOSF^+$ $[M]^+$, 173.0305; found, 173.0305; deviation: –0.03 ppm.

Iminodiphenyl-λ^6-sulfanone (6)

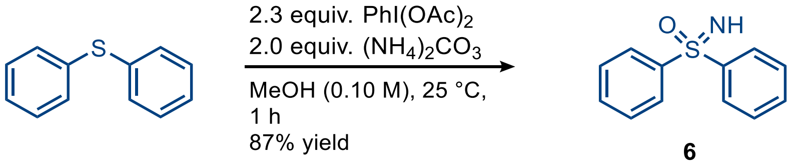

6

Under an ambient atmosphere, to a stirred solution of diphenylsulfide (186 mg, 1.00 mmol, 1.00 equiv) in MeOH (10.0 mL, *c* = 0.10 M) was added $(NH_4)_2CO_3$ (192 mg, 2.00 mmol, 2.00 equiv.). Subsequently, $PhI(OAc)_2$ (741 mg, 2.30 mmol, 2.30 equiv.) was added, then the reaction mixture was stirred at 25 °C for 1 h. The solvent was

removed under reduced pressure, and the product was purified by flash column chromatography on silica gel eluting with MeOH/DCM (1/50 v/v) to yield iminodiphenyl-λ^6-sulfanone (**6**) (190 mg, 874 μmol, 87%) as a colorless solid.

R*f* = 0.51 (MeOH/DCM = 1:50 v/v).

NMR Spectroscopy:

^{1}H NMR (500 MHz, $CDCl_3$, 298 K, δ): 8.04 (d, J = 6.9 Hz, 4H), 7.52 (t, J = 7.2 Hz, 2H), 7.47 (t, J = 7.3 Hz, 4H), 3.06 (s, 1H) ppm.

^{13}C NMR (126 MHz, $CDCl_3$, 298 K, δ): 143.6, 132.7, 129.3, 128.1. ppm.

HRMS-ESI (m/z) calculated for $C_{12}H_{11}NOSNa^+$ $[M+Na]^+$, 240.0454; found, 240.0454; deviation: –0.2 ppm.

Imino(methyl)(phenyl)-λ^6-sulfanone (S1)

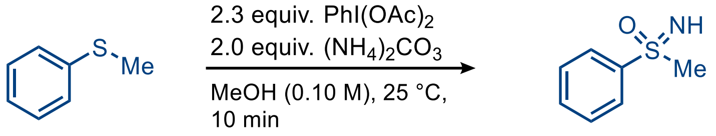

S1

Under an ambient atmosphere, to a stirred solution of (methyl)(phenyl)sulfide (124 mg, 1.00 mmol, 1.00 equiv) in MeOH (10.0 mL, c = 0.10 M) was added $(NH_4)_2CO_3$ (192 mg, 2.00 mmol, 2.00 equiv.). Subsequently, $PhI(OAc)_2$ (741 mg, 2.30 mmol, 2.30 equiv.) was added, then the reaction mixture was stirred at 25 °C for 10 min. The solvent was removed under reduced pressure, and the product was purified by flash column chromatography on silica gel eluting with MeOH/DCM (1/50 v/v) to yield imino(methyl)(phenyl)-λ^6-sulfanone (**S1**) (140 mg, 902 μmol, 90%) as a colorless oil.

R*f* = 0.23 (MeOH/DCM = 1:50 v/v).

NMR Spectroscopy:

^{1}H NMR (500 MHz, $CDCl_3$, 298 K, δ): 8.02 (d, J = 6.9 Hz, 2H), 7.62 (t, J = 7.4 Hz, 1H), 7.56 (t, J = 7.5 Hz, 2H), 3.11 (s, 3H), 2.78 (s, 1H) ppm.

^{13}C NMR (126 MHz, $CDCl_3$, 298 K, δ): 143.6, 133.1, 129.3, 127.7, 46.2 ppm.

HRMS-ESI (m/z) calculated for $C_7H_9NOS^+$ $[M]^+$, 155.0405; found, 155.0402; deviation: 2.2 ppm.

Ethyl(imino)(phenyl)-λ^6-sulfanone (S4)

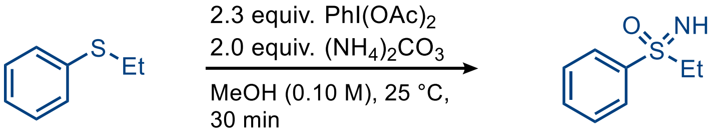

S4

Under an ambient atmosphere, to a stirred solution of (ethyl)(phenyl)sulfide (138 mg, 1.00 mmol, 1.00 equiv) in MeOH (10.0 mL, c = 0.10 M) was added $(NH_4)_2CO_3$ (192 mg, 2.00 mmol, 2.00 equiv.). Subsequently,

$PhI(OAc)_2$ (741 mg, 2.30 mmol, 2.30 equiv.) was added, then the reaction mixture was stirred at 25 °C for 30 min. The solvent was removed under reduced pressure, and the product was purified by flash column chromatography on silica gel eluting with MeOH/DCM (1/50 v/v) to yield ethyl(imino)(phenyl)-λ^6-sulfanone (**S4**) (169 mg, 886 µmol, 89%) as a colorless oil.

R*f* = 0.29 (MeOH/DCM = 1:50 v/v).

NMR Spectroscopy:

^{1}H NMR (500 MHz, $CDCl_3$, 298 K, δ): 7.97 (d, J = 8.1 Hz, 2H), 7.62 (t, J = 7.4 Hz, 1H), 7.55 (t, J = 7.7 Hz, 2H), 3.17 (q, J = 7.4 Hz, 2H), 2.68 (s, 1H), 1.26 (t, J = 7.4 Hz, 3H) ppm.

^{13}C NMR (126 MHz, $CDCl_3$, 298 K, δ): 141.6, 133.2, 129.3, 128.7, 52.0, 8.0 ppm.

HRMS-CI (m/z) calculated for $C_8H_{12}NOS^+$ $[M+H]^+$, 170.0634; found, 170.0632; deviation: 1.0 ppm.

Imino(methyl)(m-tolyl)-λ^6-sulfanone (S5)

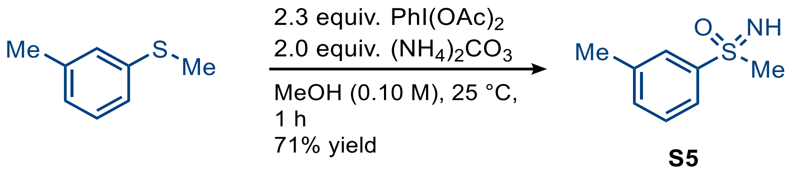

S5

Under an ambient atmosphere, to a stirred solution of methyl(m-tolyl)sulfide (138 mg, 1.00 mmol, 1.00 equiv) in MeOH (10.0 mL, c = 0.10 M) was added $(NH_4)_2CO_3$ (192 mg, 2.00 mmol, 2.00 equiv.). Subsequently, $PhI(OAc)_2$ (741 mg, 2.30 mmol, 2.30 equiv.) was added, then the reaction mixture was stirred at 25 °C for 1 h. The solvent was removed under reduced pressure, and the product was purified by flash column chromatography on silica gel eluting with MeOH/DCM (1/50 v/v) to yield imino(methyl)(m-tolyl)-λ^6-sulfanone (**S5**) (120 mg, 709 µmol, 71%) as a colorless oil.

R*f* = 0.24 (MeOH/DCM = 1:50 v/v).

NMR Spectroscopy:

^{1}H NMR (500 MHz, CD_2Cl_2, 298 K, δ): 7.81 (s, 1H), 7.77 (ddd, J = 5.6, 3.5, 2.2 Hz, 1H), 7.43 (dd, J = 5.4, 1.0 Hz, 2H), 3.04 (s, 3H), 2.90 (s, 1H), 2.44 (s, 3H) ppm.

^{13}C NMR (126 MHz, CD_2Cl_2, 298 K, δ): 143.7, 139.7, 133.8, 129.1, 128.1, 124.8, 46.3, 21.2 ppm.

HRMS-EI (m/z) calculated for $C_8H_{11}NOS^+$ $[M]^+$, 169.0556; found, 169.0557; deviation: –0.4 ppm.

Imino(methyl)(4-(trifluoromethoxy)phenyl)-λ^6-sulfanone (S6)

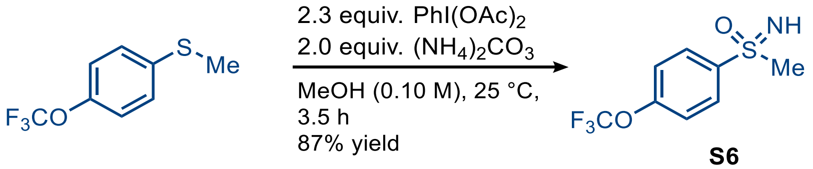

S6

Under an ambient atmosphere, to a stirred solution of methyl(4-(trifluoromethoxy)phenyl)sulfide (208 mg, 1.00 mmol, 1.00 equiv) in MeOH (10.0 mL, *c* = 0.10 M) was added $(NH_4)_2CO_3$ (192 mg, 2.00 mmol, 2.00 equiv.). Subsequently, $PhI(OAc)_2$ (741 mg, 2.30 mmol, 2.30 equiv.) was added, then the reaction mixture was stirred at 25 °C for 3.5 h. The solvent was removed under reduced pressure, and the product was purified by flash column chromatography on silica gel eluting with MeOH/DCM (1/50 v/v) to yield imino(methyl)(4-(trifluoromethoxy)phenyl)-λ^6-sulfanone (**S6**) (209 mg, 874 μmol, 87%) as a colorless solid.

R*f* = 0.39 (MeOH/DCM = 1:50 v/v).

NMR Spectroscopy:

^{1}**H NMR** (500 MHz, CD_2Cl_2, 298 K, δ): 8.06 (d, *J* = 8.9 Hz, 2H), 7.39 (d, *J* = 8.9 Hz, 2H), 3.07 (s, 3H), 2.71 (s, 1H) ppm.

^{13}**C NMR** (126 MHz, CD_2Cl_2, 298 K, δ): 153.2, 143.1, 130.9, 121.9, 120.1, 47.1 ppm.

^{19}**F NMR** (471 MHz, CD_2Cl_2, 298 K, δ): –58.1 (m) ppm.

HRMS-CI (m/z) calculated for $C_8H_9NO_2SF_3^+$ $[M+H]^+$, 240.0301; found, 240.0299; deviation: 0.5 ppm.

Imino(methyl)(thiophen-2-yl)-λ^6-sulfanone (S7)

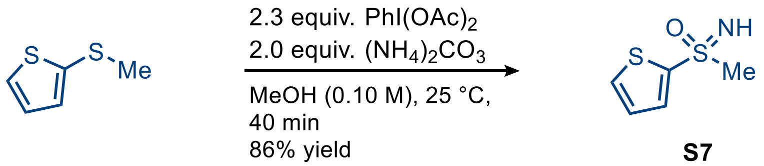

S7

Under an ambient atmosphere, to a stirred solution of 2-(methylthio)thiophene (130 mg, 1.00 mmol, 1.00 equiv) in MeOH (10.0 mL, *c* = 0.10 M) was added $(NH_4)_2CO_3$ (192 mg, 2.00 mmol, 2.00 equiv.). Subsequently, $PhI(OAc)_2$ (741 mg, 2.30 mmol, 2.30 equiv.) was added, then the reaction mixture was stirred at 25 °C for 40 min. The solvent was removed under reduced pressure, and the product was purified by flash column chromatography on silica gel eluting with MeOH/DCM (1/50 v/v) to yield imino(methyl)(thiophen-2-yl)-λ^6-sulfanone (**S7**) (138 mg, 856 μmol, 86%) as a slightly yellowish solid.

R*f* = 0.21 (MeOH/DCM = 1:50 v/v).

NMR Spectroscopy:

^{1}**H NMR** (500 MHz, $CDCl_3$, 298 K, δ): 7.66 (m, 2H), 7.12 (t, *J* = 4.99 Hz, 3.72, 1H), 3.24 (s, 3H), 3.08 (s, 1H) ppm.

^{13}**C NMR** (126 MHz, $CDCl_3$, 298 K, δ): 145.8, 133.4, 133.1, 127.8, 47.5. ppm.

HRMS-ESI (m/z) calculated for $C_5H_7NOS_2Na^+$ $[M+Na]^+$, 183.9861; found, 183.9863; deviation: –1.0 ppm.

(4-Bromophenyl)(imino)(methyl)-λ^6-sulfanone (S8)

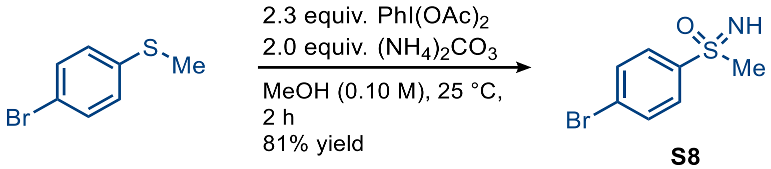

S8

Under an ambient atmosphere, to a stirred solution of (4-bromophenyl)(methyl)sulfide (203 mg, 1.00 mmol, 1.00 equiv) in MeOH (10.0 mL, *c* = 0.10 M) was added $(NH_4)_2CO_3$ (192 mg, 2.00 mmol, 2.00 equiv.). Subsequently, $PhI(OAc)_2$ (741 mg, 2.30 mmol, 2.30 equiv.) was added, then the reaction mixture was stirred at 25 °C for 2 h. The solvent was removed under reduced pressure, and the product was purified by flash column chromatography on silica gel eluting with MeOH/DCM (1/50 v/v) to yield (4-bromophenyl)(imino)(methyl)-λ^6-sulfanone (**S8**) (190 mg, 813 µmol, 81%) as a colorless solid.

Rf = 0.17 (MeOH/DCM = 1:50 v/v).

NMR Spectroscopy:

^{1}**H NMR** (500 MHz, CD_2Cl_2, 298 K, δ): 7.86 (d, *J* = 8.6 Hz, 2H), 7.70 (d, *J* = 8.6 Hz, 2H), 3.05 (s, 3H), 2.72 (s, 1H).

^{13}**C NMR** (126 MHz, CD_2Cl_2, 298 K, δ): 143.6, 133.0, 130.0, 128.5, 46.8 ppm.

HRMS-EI (m/z) calculated for $C_7H_8NOSBr^+$ $[M]^+$, 232.9505; found, 232.9502; deviation: 1.3 ppm.

10-Imino-10H-10 λ^4-phenoxathiine 10-oxide (S9)

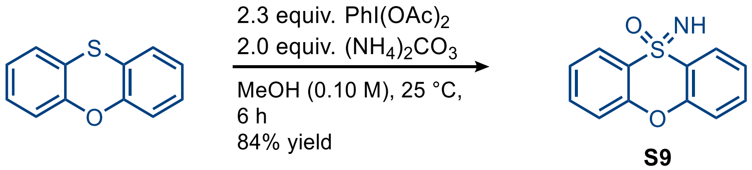

S9

Under an ambient atmosphere, to a stirred solution of phenoxathiine (200 mg, 1.00 mmol, 1.00 equiv) in MeOH (10.0 mL, *c* = 0.10 M) was added $(NH_4)_2CO_3$ (192 mg, 2.00 mmol, 2.00 equiv.). Subsequently, $PhI(OAc)_2$ (741 mg, 2.30 mmol, 2.30 equiv.) was added, then the reaction mixture was stirred at 25 °C for 6 h. The solvent was removed under reduced pressure, and the product was purified by flash column chromatography on silica gel eluting with MeOH/DCM (1/50 v/v) to yield 10-imino-10H-10 λ^4-phenoxathiine 10-oxide (**S9**) (195 mg, 843 µmol, 84%) as a colorless solid.

Rf = 0.48 (MeOH/DCM = 1:50 v/v).

NMR Spectroscopy:

^{1}**H NMR** (500 MHz, CD_2Cl_2, 298 K, δ): 8.06 (dd, *J* = 7.8, 1.7 Hz, 2H), 7.62 (dd, 2H), 7.38 (dt, 4H), 3.28 (s, 1H) ppm.

^{13}**C NMR** (126 MHz, CD_2Cl_2, 298 K, δ): 151.1, 133.5, 127.9, 124.8, 123.2, 118.7 ppm.

HRMS-EI (m/z) calculated for C12H9NO2S+ [M]+, 231.0349; found, 231.0347; deviation: 0.5 ppm.

Imino(4-iodophenyl)(methyl)-λ6-sulfanone (S10)

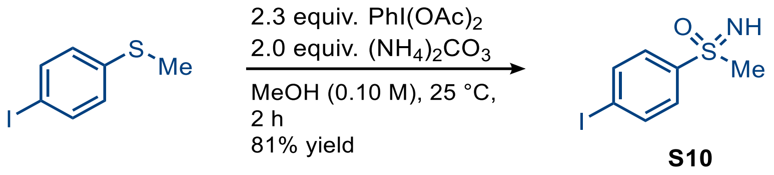

Under an ambient atmosphere, to a stirred solution of (4-iodophenyl)(methyl)sulfide (250 mg, 1.00 mmol, 1.00 equiv) in MeOH (10.0 mL, *c* = 0.10 M) was added $(NH_4)_2CO_3$ (192 mg, 2.00 mmol, 2.00 equiv.). Subsequently, $PhI(OAc)_2$ (741 mg, 2.30 mmol, 2.30 equiv.) was added, then the reaction mixture was stirred at 25 °C for 2 h. The solvent was removed under reduced pressure, and the product was purified by flash column chromatography on silica gel eluting with MeOH/DCM (1/50 v/v) to yield imino(4-iodophenyl)(methyl)-λ6-sulfanone (**S10**) (230 mg, 818 μmol, 82%) as a colorless solid.

R*f* = 0.13 (MeOH/DCM = 1:50 v/v).

NMR Spectroscopy:

^{1}**H NMR** (500 MHz, CD_2Cl_2, 298 K, δ): 7.92 (d, J = 8.58 Hz, 2H), 7.71 (d, J = 8.54 Hz, 2H), 3.05 (s, 3H), 2.44 (s, 1H).

^{13}**C NMR** (126 MHz, CD_2Cl_2, 298 K, δ): 143.8, 138.6, 129.4, 100.5, 46.3 ppm.

HRMS-EI (m/z) calculated for $C_7H_8NOSI^+$ $[M]^+$, 280.9366; found, 280.9367; deviation: –0.5 ppm.

Mechanistic Studies: Copper-LMCT enabled C–H sulfoximination

Control experiments using N-bromo sulfoximine[3] as radical precusor

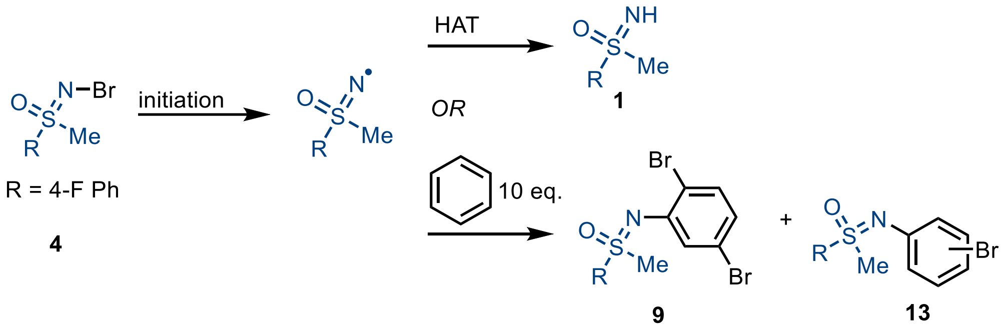

Entry	Initiation	Cu(II)	Cu(I)	d**1**	d**9+13**
a1	AIBN, 80 °C	/	/	84%	/
a2	AIBN, 80 °C	2 eq	/	0	0
a3	AIBN, 80 °C	/	2 eq	0	0
b4	Blue LEDs	/	/	94%	0
b5	Blue LEDs	2 eq	/	0	6%

[b]6	Blue LEDs	/	2 eq	0	0
[b]7	Blue LEDs	/, 2 eq NFTPT	/	78%	0
[c]8	Violet LEDs	2 eq	/	0	13%

N-Bromo sulfoximine **4** was prepared according to the literature.[3] Cu(II): $Cu(OTf)_2$, Cu(I): $Cu(MeCN)_4BF_4$, NFTPT: 1-fluoro-2,4,6-trimethylpyridinium tetrafluoroborate.

[a]Thermal initiation conditions:[4] *N*-Bromo sulfoximine **4** (25 mg, 0.10 mmol, 1.0 equiv.), 2,2′-azobis(2-methylpropionitril) (AIBN, 1 mg, 5 μmol, 5 mol%), benezene (90 μL, 78 mg, 10 equiv.), DCM/MeCN (0.1 M, 1/1, v,v) , 80 °C, 5 h.

[b]Light-induced initiation with blue LEDs conditions:[5] *N*-Bromo sulfoximine **4** (13 mg, 50 μmol, 1.0 equiv.), benezene (45 μL, 39 mg, 1.0 mmol, 10 equiv.), DCM/MeCN (25 mM, 1/1, v,v), blue LEDs (450 nm) irradiation, 35 °C, 15 h.

[c]Light-induced initiation with violet LEDs conditions: *N*-Bromo sulfoximine **4** (13 mg, 50 μmol, 1.0 equiv.), LiOMe (7.5 mg, 0.20 mmol, 1.0 equiv.), 2,6-di-tert-butylpyridine (76.5 mg, 0.400 mmol, 2.00 equiv.), benezene (45 μL, 39 mg, 1.0 mmol, 10 equiv.), MeCN (25 mM), violet LEDs (390 nm) irradiation, 35 °C, 15 h.

[d] ^{19}F NMR yield with 2-fluorotoluene(1.0 equiv.) as an internal standard.

HRMS-EI (m/z) calculated for $C_{13}H_{10}NOSFBr_2^+$ $[M]^+$, 404.8828; found, 404.8836; deviation: –1.8 ppm.

HRMS-EI (m/z) calculated for $C_{13}H_{11}NOSFBr^+$ $[M]^+$, 326.9723; found, 326.9730; deviation: –2.0 ppm.

HRMS-EI (m/z) calculated for $C_{13}H_{11}NOSFBr^+$ $[M]^+$, 326.9723; found, 326.9729; deviation: –1.8 ppm.

Emission spectrum of Kessil PR160L(390nm) LEDs[6]

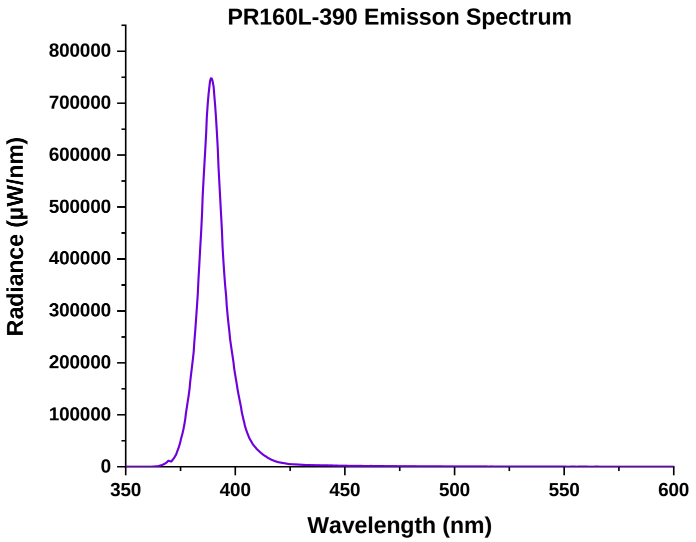

Figure S1. Emission spectrum of the violet LEDs used in the reaction.

Cyclic voltammograms

Cyclic voltammograms were recorded using an Autolab PGSTAT204 potentiostat and a Pt working electrode, a Ag/AgCl reference electrode and a Pt sheet auxiliary electrode.

The voltammogram of (4-chlorophenyl)(imino)(methyl)-λ^6-sulfanone was recorded at 25 °C in 0.1 M tetrabutylammonium hexafluorophosphate in MeCN (3 mL) containing (4-chlorophenyl)(imino)(methyl)-λ^6-sulfanone (5.7 mg, 0.030 mmol). The scan rate was 100 mV s^{-1}. E_{ox} = 2.25 V vs. Ag/AgCl in MeCN was observed. Thus, for (4-chlorophenyl)(imino)(methyl)-λ^6-sulfanone, E_{ox} = 2.21 V vs. SCE in MeCN was obtained.

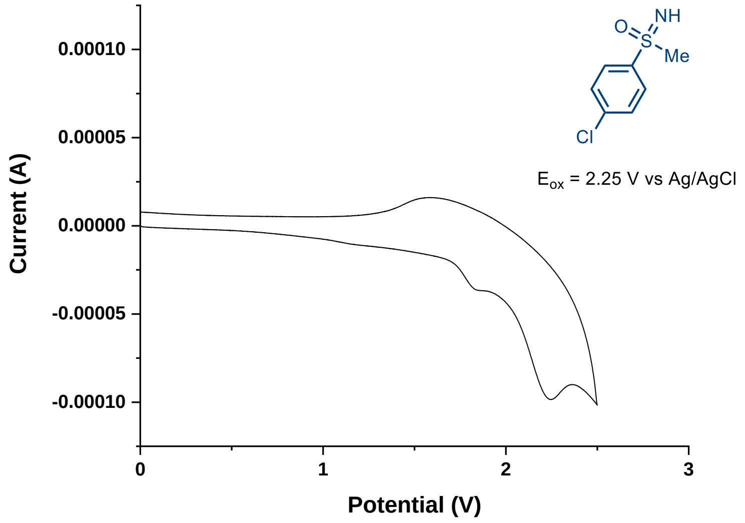

Figure S2. Voltammogram of (4-chlorophenyl)(imino)(methyl)-λ^6-sulfanone in MeCN

The voltammogram of deprotonated (4-chlorophenyl)(imino)(methyl)-λ^6-sulfanone was recorded at 25 °C in 0.1 M tetrabutylammonium hexafluorophosphate in MeCN (3 mL) containing both NH- and lithiated sulfoximine. The scan rate was 100 mV s^{-1}. Two oxidation peaks were observed. E_{ox} = 2.20 V vs. Ag/AgCl in MeCN was assigned to the NH-sulfoximine by comparing with the voltammogram of the pure compound. E_{ox} = 1.90 V vs. Ag/AgCl in MeCN was assigned to the deprotonated sulfoximine.Thus, for lithiated (4-chlorophenyl)(imino)(methyl)-λ^6-sulfanone, E_{ox} = 1.86 V vs. SCE in MeCN was obtained.

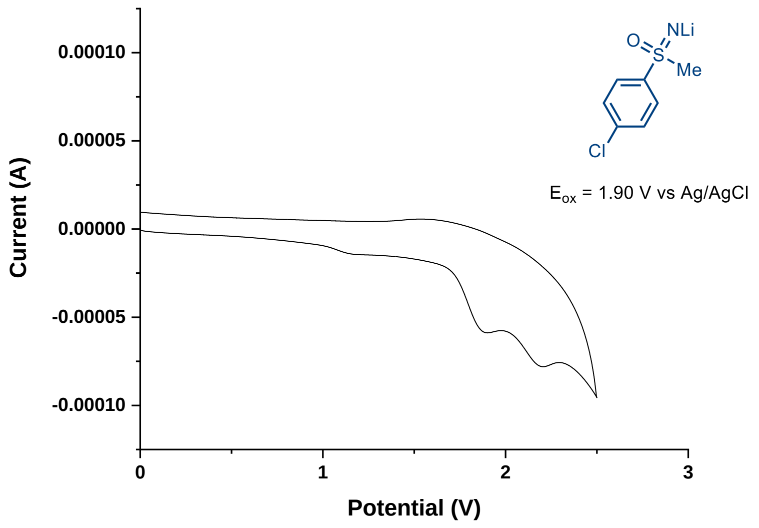

Figure S3. Voltammogram of lithiated (4-chlorophenyl)(imino)(methyl)-λ^6-sulfanone in MeCN

Note: The sample was prepared by the following procedure.

(4-chlorophenyl)(imino)(methyl)-λ^6-sulfanone (94.8 mg, 0.500 mmol, 1.0 equiv) and LiH (4.5mg, 0.55 mmol, 1.0 equiv.) were added in 10 mL MeCN, and the reaction mixture is stirred at 25 °C for 30 mins, which resulted in a white suspension. 3.0 mL of the reaction mixture was taken out using a 5.0 mL syring and filtered through a 0.22 μm syringe filter. To the filtrate, tetrabutylammonium hexafluorophosphate (116 mg, 0.300 mmol, 0.10 M) were added. Then the cyclic voltammograms were recorded.

UV–vis absorption spectrum of reaction components in C–H sulfoximination

Samples were prepared in a glovebox and then taken out of the glovebox. UV–vis spectra of benzene (150 mM), the mixture of benzene (150 mM) + (4-fluorophenyl)(imino)(methyl)-λ^6-sulfanone (**1**, 3.0 mM), the mixture of benzene (150 mM) + $Cu(OTf)_2$ (6.0 mM), the mixture of **1** (3.0 mM) + $Cu(OTf)_2$ (6.0 mM), the mixture of benzene (150 mM) + **1** (3.0 mM) + LiOMe (3.0 mM) + 2,6-di-tert-butylpyridine (DTBP, 6.0 mM), the mixture of benzene (150 mM) + **1** (3.0 mM) + LiOMe (3.0 mM) + 2,6-di-tert-butylpyridine (DTBP, 6.0 mM) + $Cu(OTf)_2$ (6.0 mM) were recorded respectively, using MeCN as the solvent.

Note: Not all the LiOMe was dissolved in MeCN. All LiOMe-containing samples were filtered through a 0.22 μm syringe filter before measurement, so the actual concentration of LiOMe in these samples is less than 3.0 mM.

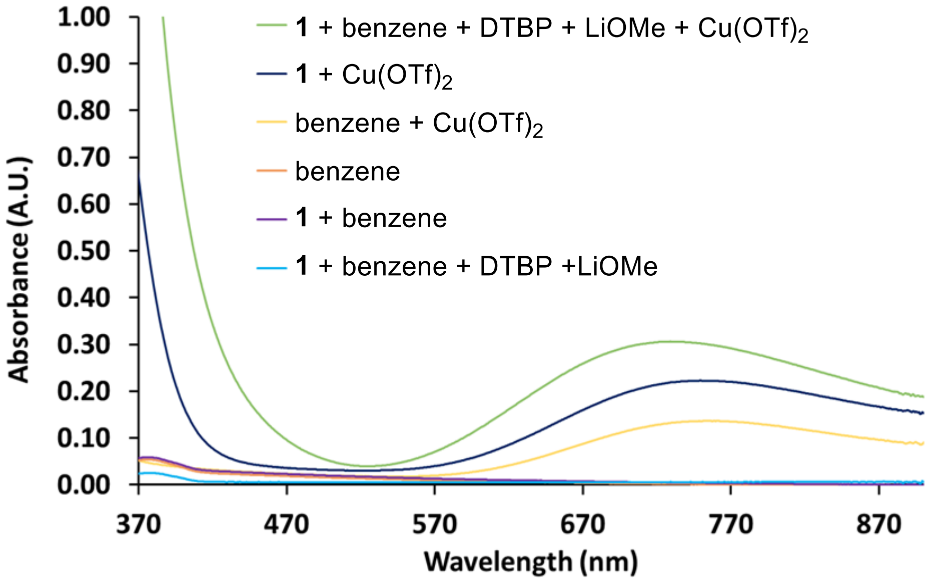

Figure S4. UV–vis spectra analysis of the reaction components in C–H sulfoximination

Photolysis of the mixture of 1, benzene, DTBP, LiOMe and $Cu(OTf)_2$

In a nitrogen-filled glovebox, a mixture of **1** (3.0 mM) + benzene (150 mM) + DTBP (6.0 mM) + LiOMe (3.0 mM) + $Cu(OTf)_2$ (6.0 mM) in 3.0 mL MeCN was transferred to a screw-top quartz cuvette (Hellma fluorescence quartz cuvette, 10 × 10 mm, 3.5 mL). The quartz cuvette was sealed and taken out of the glovebox. The absorption spectra were recorded on a Shimadzu UV–vis Spectrophotometer UV-2600 after the cuvette was irradiated by two Kessil PR160L-390 nm LEDs (5 cm away from two Kessil PR160L-390 nm LEDs, and the temperature was maintained at approximately 35 °C through cooling with a fan) at various times (0 min, 3 min, 8 min, 16 min, 31 min, 75 min).

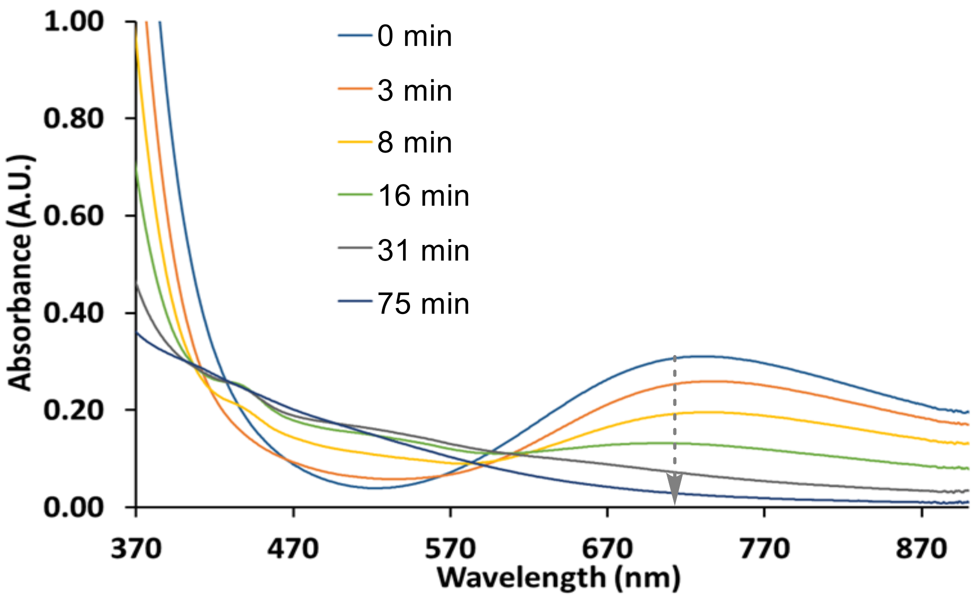

Figure S5. UV–vis spectra of the photolyzed mixture of **1**, benzene, DTBP, LiOMe and $Cu(OTf)_2$ in CH_3CN.

After photolysis, upon addition of 2,2′-biquinoline (1.8 mg) to the reaction mixture, the colourless solution turned to dark purple colour, which indicates the formation of purple $[Cu^I(biq)_2]^+$ complex.[7] 0.1 mL of the above purple reaction mixture was taken out and further diluted to 3.0 mL with MeCN. The absorption spectrum was recorded on a UV–vis spectrophotometer, and a significant absorbance (λ_{max} = 546 nm) was observed.

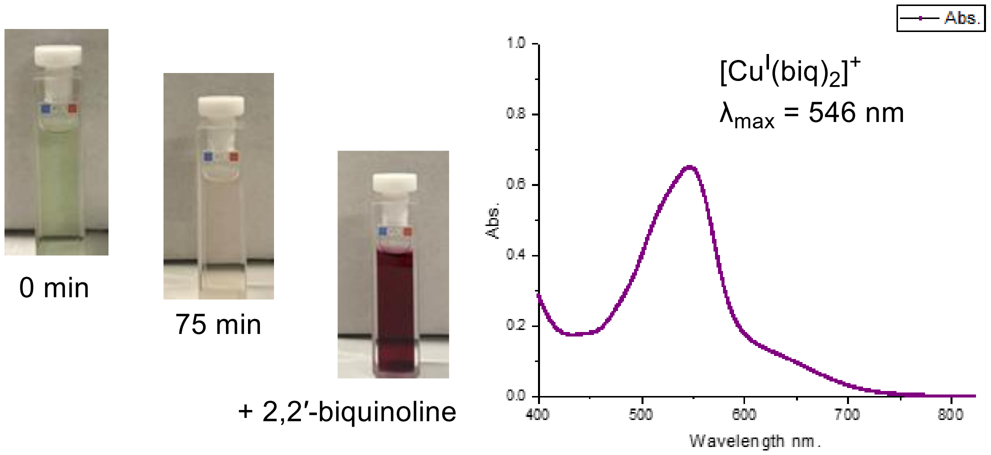

Figure S6. Formation of $[Cu^I(biq)_2]^+$; UV–vis spectrum of the resuting mixture after addition of 2,2′-biquinoline

Synthesis of *N,N*-diphenylmethacrylamide (7)[8]

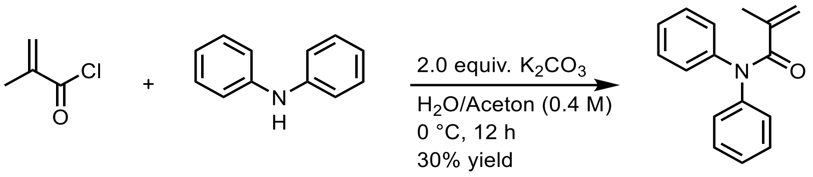

7

Methacryloyl chloride (2.0 g,1.9 mL, 20 mmol, 2.0 equiv) was added to a stirred suspension of potassium carbonate (2.76 g, 20 mmol, 2 equiv) in water (5 mL) and acetone (20 mL) at 0 °C under atmosphere of nitrogen, and then diphenylamine (0.93 g, 10 mmol, 1 equiv) was added in small portions into the mixture. The suspension was stirred at 0 °C for 12h. After filtration, the mixture was concentrated under reduced pressure and extracted with ethyl acetate. The organic layer was dried over Na_2SO_4, and concentrated under reduced pressure. The residue was purified by flash chromatography with EtOAc/Hexanes (1:3) to give the corresponding product **7** as a beige solid. Recrystalization from pentane/DCM affords pure *N,N*-diphenylmethacrylamide (**7**) (0.35 g, 1.5 mmol, 30%) as colorless needle-shaped crystals.

R*f* =0.3 (EA/pentane, 1/10, v/v).

NMR Spectroscopy:

^{1}**H NMR** (500 MHz, $CDCl_3$, 298 K, δ): 7.33 (dd, *J* = 8.4, 7.3 Hz, 4H), 7.24 – 7.20 (m, 2H), 7.17 (dd, *J* = 8.4, 1.2 Hz, 4H), 5.23 (t, *J* = 1.1 Hz, 1H), 5.17 (t, *J* = 1.4 Hz, 1H), 1.84 (t, *J* = 1.3 Hz, 3H). ppm.

^{13}C NMR (126 MHz, $CDCl_3$, 298 K, δ): 172.0, 143.6, 141.3, 129.2, 127.3, 126.6, 121.1, 20.1. ppm.

HRMS-EI (m/z) calculated for $C_{16}H_{15}NO^+$ $[M]^+$, 237.1148; found, 237.1150; deviation: –0.7 ppm.

Radical cyclisation experiment: proof of sulfoximinyl radical intermediate

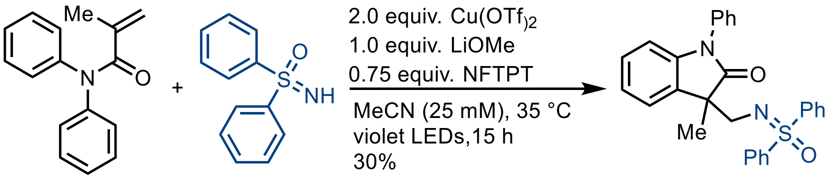

10 eq. **8**

Under an ambient atmosphere, a 8 mL borosilicate vial equipped with a magnetic stir bar was charged with diphenyl sulfoximine (21.7 mg, 0.100 mol, 1.00 equiv.). The vial was transferred into an anhydrous, N_2-filled glovebox. Under nitrogen atmosphere, $Cu(OTf)_2$ (72.3 mg, 0.200 mmol, 2.00 equiv.), LiOMe (3.8 mg, 0.10 mmol, 1.0 equiv.), and anhydrous MeCN (4.0 mL, *c* = 25 mM) were added into the vial, resulting in a blue suspension. The reaction mixture was stirred without irradiation for 30 min at 25 °C. Subsequently, 1-fluoro-2,4,6-trimethylpyridinium tetrafluoroborate (NFTPT) (17 mg, 75 µmol, 0.75 equiv.), *N,N*-diphenylmethacrylamide (237 mg, 1.00 mmol, 10.0 equiv.) were added into the vial. The vial was sealed with a Teflon cap and taken out of the glovebox. The sealed vial was placed 5 cm away from two violet LEDs (Kessil PR160L-390 nm LEDs). The reaction mixture was stirred at a speed of 1000 rpm and irradiated for 15 h while maintaining the temperature at approximately 35 °C through cooling with a fan. After irradiation, volatiles were removed from the reaction mixture under reduced pressure. The residue was purified by flash column chromatography on silica gel (EA/pentane = 1/8, v/v) to afford 3-methyl-3-(((oxodiphenyl-λ^6-sulfaneylidene)amino)methyl)-1-phenylindolin-2-one (**8**) (13.5 mg, 30.0 µmol, 30%, mixture of two enantiomers)

R*f* = 0.2 (EA/pentane = 1/4, v/v).

NMR Spectroscopy:

^{1}H NMR (600 MHz, $CDCl_3$, 298 K, δ): 7.84 (dd, *J* = 8.5, 1.3 Hz, 2H), 7.73 – 7.70 (m, 2H), 7.54 – 7.50 (m, 2H), 7.49 – 7.44 (m, 4H), 7.42 – 7.38 (m, 3H), 7.37 – 7.33 (m, 2H), 7.32 (ddd, *J* = 7.4, 1.3, 0.6 Hz, 1H), 7.21 (td, *J* = 7.7, 1.3 Hz, 1H), 7.10 (td, *J* = 7.5, 1.0 Hz, 1H), 6.86 (ddd, *J* = 7.8, 1.0, 0.5 Hz, 1H), 3.50 (d, *J* = 11.5 Hz, 1H), 3.40 (d, *J* = 11.5 Hz, 1H), 1.50 (s, 3H). ppm.

^{13}C NMR (151 MHz, $CDCl_3$, 298 K, δ): 179.8, 144.1, 140.9, 140.5, 135.2, 134.0, 132.4, 132.3, 129.6, 129.2, 129.01, 128.98, 128.7, 127.9, 127.7, 126.8, 123.7, 122.8, 109.0, 51.3, 50.5, 20.5. ppm.

HRMS-ESI (m/z) calculated for $C_{28}H_{24}N_2O_2S^+$ $[M+H]^+$, 453.1631; found, 453.1628; deviation: 0.7 ppm.

C–H sulfoximination of sulfoximinyl-containing I(III) reagent[9]

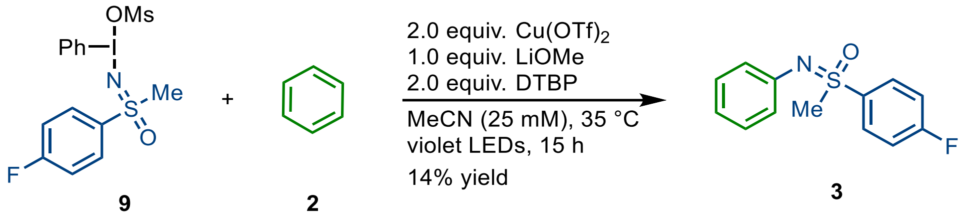

Sulfoximinyl-containing I(III) reagent **9** was prepared according to the literature.[9] In a nitrogen-filled glovebox, a 10 mL borosilicate vial equipped with a magnetic stir bar was charged with (4-fluorophenyl)(imino)(methyl)-λ6-sulfanone derived I(III) reagent (94.2 mg, 0.200 mmol, 1.00 equiv.), LiOMe (7.5 mg, 0.20 mmol, 1.0 equiv.), and $Cu(OTf)_2$ (145 mg, 0.400 mmol, 2.00 equiv.). 2,6-Di-tert-butylpyridine (76.5 mg, 0.400 mmol, 2.00 equiv.), benzene (781 mg, 890 μL, 10.0 mmol, 50.0 equiv.), and anhydrous MeCN (8.0 mL, c = 25 mM) were then added into the vial. The vial was sealed with a Teflon cap and placed 5 cm away from two violet LEDs (Kessil PR160L-390 nm LEDs). The reaction mixture was irradiated for 15 h while maintaining the temperature at approximately 35 °C through cooling with a fan. After irradiation, the reaction mixture was evaporated under reduced pressure to remove all volatiles. EtOAc (15 mL) was added to the residue, and the resulting suspension was filtered through a silica pad, eluting with EtOAc (15 mL). The filtrate was concentrated under reduced pressure, then 1 mL $CDCl_3$ was added. 2-Fluorotoluene (22.0 mg, 22.0 μL, 1.0 equiv.) was added as the internal standard. An ^{19}F NMR spectrum was measured to determine the yield of (4-fluorophenyl)(methyl)(phenylimino)-λ6-sulfanone (**3**) (14%).

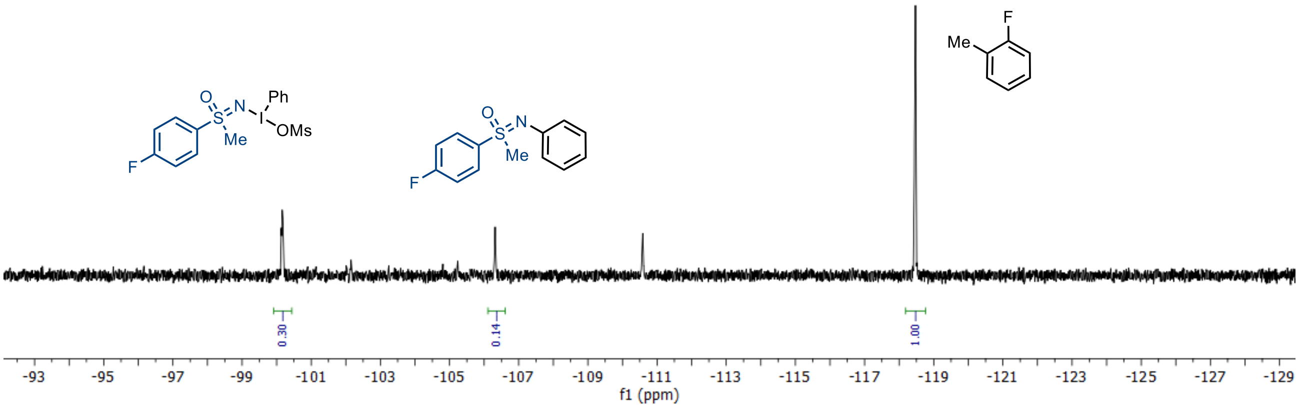

^{19}F NMR of the reaction mixture

HRMS-APPIpos (m/z) calculated for $C_{13}H_{13}NOSF^+$ $[M+H]^+$, 250.0696; found, 250.0696; deviation: 0.2 ppm.

Kinetic isotope effect (KIE) determination by competition experiment

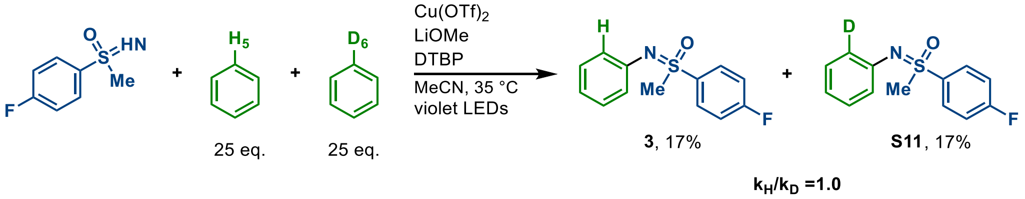

In a nitrogen-filled glovebox, a 10 mL borosilicate vial equipped with a magnetic stir bar was charged with (4-fluorophenyl)(imino)(methyl)-λ^6-sulfanone (34.6 mg, 0.200 mmol, 1.00 equiv.), LiOMe (7.5 mg, 0.20 mmol, 1.0 equiv.), and $Cu(OTf)_2$ (145 mg, 0.400 mmol, 2.00 equiv.). 2,6-Di-tert-butylpyridine (76.5 mg, 0.400 mmol, 2.00 equiv.), benzene (390 mg, 5.00 mmol, 25.0 equiv.), benzene-d_6 (421 mg, 5.00 mmol, 25.0 equiv.), and anhydrous MeCN (8.0 mL, c = 25 mM) were then added into the vial. The vial was sealed with a Teflon cap and placed 5 cm away from two violet LEDs (Kessil PR160L-390 nm LEDs). The reaction mixture was irradiated for 15 h while maintaining the temperature at approximately 35 °C through cooling with a fan. After irradiation, the reaction mixture was evaporated under reduced pressure to remove all volatiles. EtOAc (15 mL) was added to the residue, and the resulting suspension was filtered through a silica pad, eluting with EtOAc (15 mL). The filtrate was concentrated under reduced pressure, then 1 mL $CDCl_3$ was added.Dibromomethane (34.7 mg, 14.0 μL, 0.200 mmol, 1.00 equiv.) was added as the internal standard. ^1H NMR was measured to determin the ratio of **3**/**S11**.

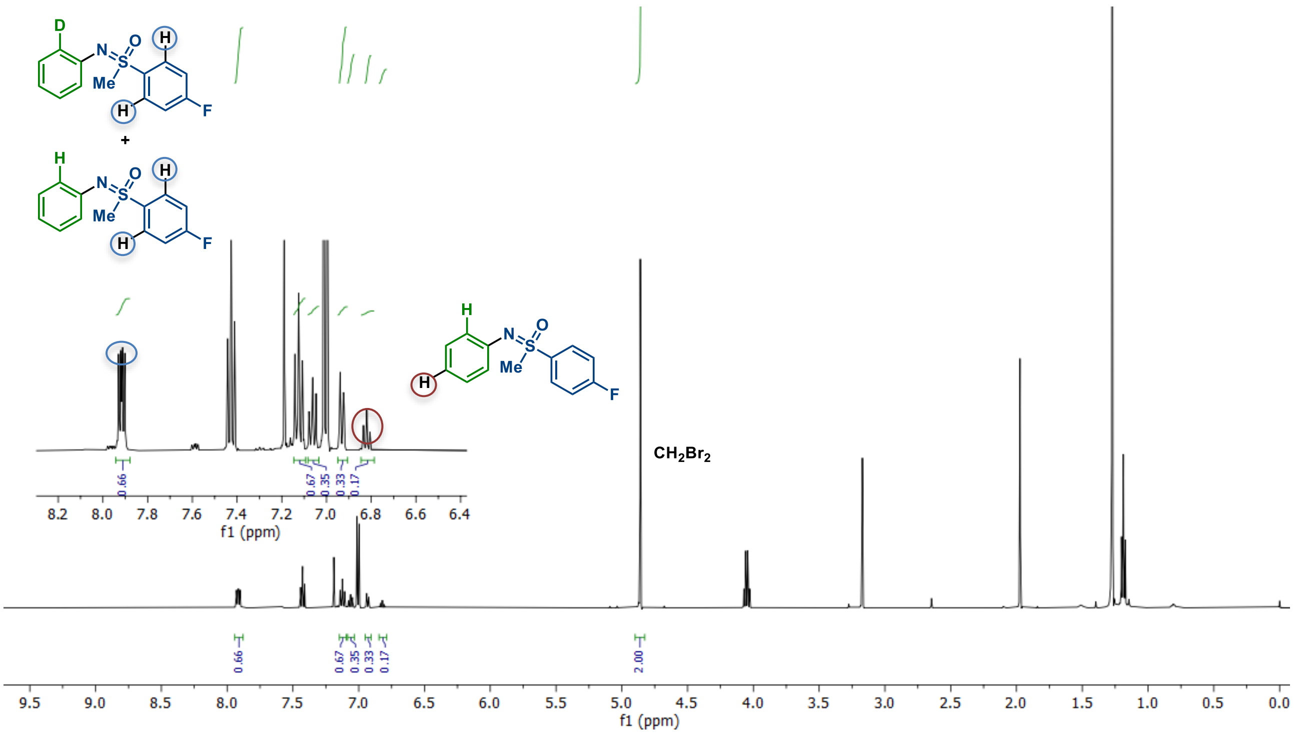

^1H NMR of the reaction mixture

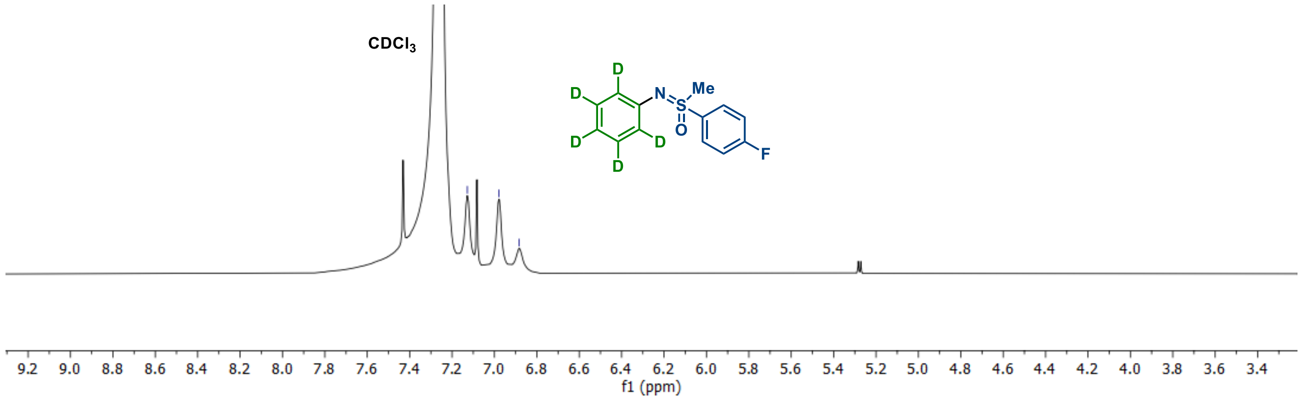

^{2}H NMR of the reaction mixture

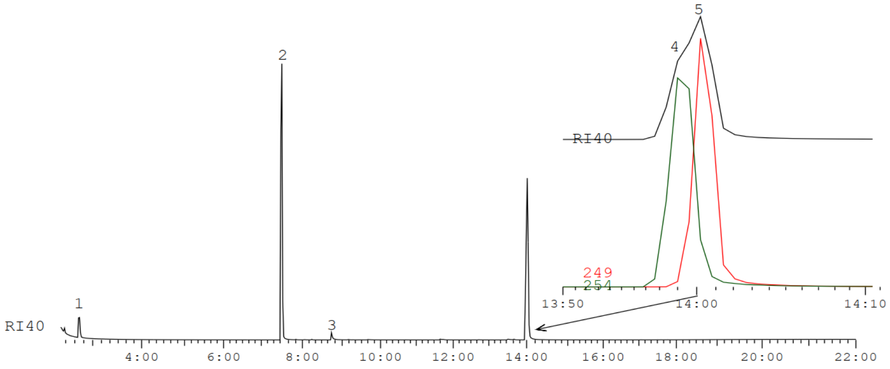

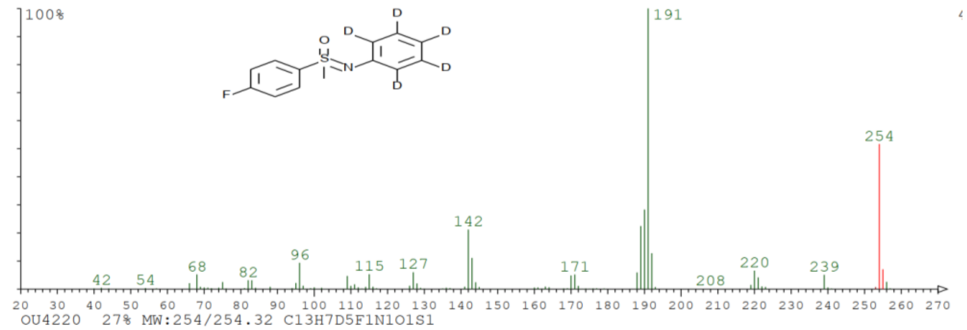

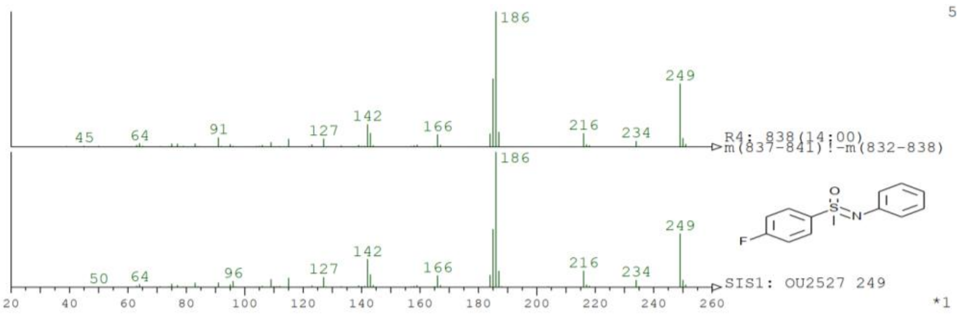

GC-MS of the reaction mixture

HRMS-EI (m/z) calculated for $C_{13}H_{12}NOSF^{+}$ [M]$^{+}$, 249.0618; found, 249.0620; deviation: –0.7 ppm.

HRMS-EI (m/z) calculated for $C_{13}H_{7}NOSFD_{5}^{+}$ [M]$^{+}$, 254.0932; found, 254.0933; deviation: –0.8 ppm.

Copper-LMCT enabled C–H sulfoximination: arene scope

(4-Fluorophenyl)(methyl)(phenylimino)-λ^6-sulfanone (3)

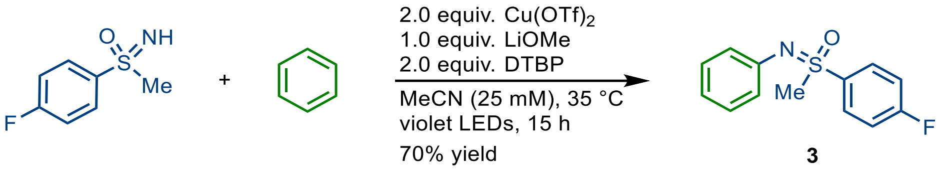

In a nitrogen-filled glovebox, a 10 mL borosilicate vial equipped with a magnetic stir bar was charged with and (4-fluorophenyl)(imino)(methyl)-λ^6-sulfanone (34.6 mg, 0.200 mmol, 1.00 equiv.), LiOMe (7.5 mg, 0.20 mmol, 1.0 equiv.), $Cu(OTf)_2$ (145 mg, 0.400 mmol, 2.00 equiv.), 2,6-di-tert-butylpyridine (76.5 mg, 0.400 mmol, 2.00 equiv.) and benzene (1.56 g, 1.78 mL, 20.0 mmol, 100 equiv.), anhydrous MeCN (8.0 mL, c = 25 mM) was then added into the vial. The vial was sealed with a Teflon cap and placed 5 cm away from two violet LEDs (Kessil PR160L-390 nm LEDs). The reaction mixture was irradiated for 15 h while maintaining the temperature at approximately 35 °C through cooling with a fan. After irradiation, the reaction mixture was evaporated under reduced pressure to remove all volatiles. The residue was purified by chromatography on silica gel (EA/DCM = 1/50, v/v) to yield (4-fluorophenyl)(methyl)(phenylimino)-λ^6-sulfanone (**3**) (34.9 mg, 140 μmol, 70%) as a colorless oil.

R*f* = 0.40 (DCM).

NMR Spectroscopy:

> **^{1}H NMR** (500 MHz, $CDCl_3$, 298 K, δ): 8.02–7.95 (m, 2H), 7.23–7.17 (m, 2H), 7.16–7.11 (m, 2H), 7.02–6.97 (m, 2H), 6.89 (tt, J = 7.2, 1.2 Hz, 1H), 3.24 (s, 3H) ppm.
>
> **^{13}C NMR** (126 MHz, $CDCl_3$, 298 K, δ): 165.7 (d, J = 255.7 Hz), 144.7, 135.4, 131.6 (d, J = 9.6 Hz), 129.2, 123.5, 122.1, 117.0 (d, J = 22.5 Hz), 46.3 ppm.
>
> **^{19}F NMR** (471 MHz, $CDCl_3$, 298 K, δ): –104.6 (m) ppm.

HRMS-ESI (m/z) calculated for $C_{13}H_{12}NOSF^+$ [M]$^+$, 249.0618; found, 249.0619; deviation: –0.2 ppm.

((2,5-Dichlorophenyl)imino)(4-fluorophenyl)(methyl)-λ^6-sulfanone (11)

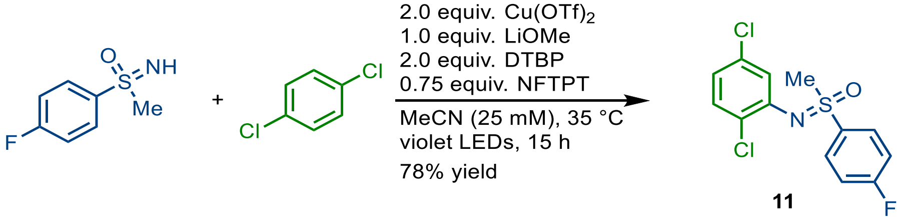

Under an ambient atmosphere, a 10 mL borosilicate vial equipped with a magnetic stir bar was charged with (4-fluorophenyl)(imino)(methyl)-λ^6-sulfanone (34.6 mg, 0.200 mmol, 1.00 equiv.). The vial was transferred into an anhydrous, N_2-filled glovebox. Under nitrogen atmosphere, $Cu(OTf)_2$ (144 mg, 0.400 mmol, 2.00 equiv.), LiOMe (7.5 mg, 0.20 mmol, 1.0 equiv.), and anhydrous MeCN (8.0 mL, c = 25 mM) were added into the vial,

resulting in a blue suspension. The reaction mixture was stirred without irradiation for 30 min at 25 °C. Subsequently, 1-fluoro-2,4,6-trimethylpyridinium tetrafluoroborate (NFTPT) (34.0 mg, 0.15 mmol, 0.75 equiv.), 1,4-dichlorobenzene (1.47 g, 10.0 mmol, 50.0 equiv.), and 2,6-di-*tert*-butylpyridine (90.0 μL, 76.5 mg, 0.400 mmol, 2.00 equiv.) were added into the vial, resulting in a green suspension. The vial was sealed with a Teflon cap and taken out of the glovebox. The sealed vial was placed 5 cm away from two violet LEDs (Kessil PR160L-390 nm LEDs). The reaction mixture was stirred at a speed of 1000 rpm and irradiated for 15 h while maintaining the temperature at approximately 35 °C through cooling with a fan. After irradiation, volatiles were removed from the reaction mixture under reduced pressure. DCM (15 mL) was added to the residue, and the precipitate was removed by filtration through a glass frit. The filtrate was concentrated under reduced pressure, and the residue was purified by flash column chromatography on silica gel (EA/pentane = 1/5, v/v) to yield ((2,5-dichlorophenyl)imino)(4-fluorophenyl)(methyl)-λ^6-sulfanone (**11**) (49.5 mg, 156 μmol, 78%) as a colorless solid.

R*f* = 0.30 (EA/pentane = 1/5, v/v).

NMR Spectroscopy:

^{1}H NMR (500 MHz, $CDCl_3$, 298 K, δ): 8.04 (dd, *J* = 8.9, 5.0 Hz, 2H), 7.25 – 7.22 (m, 2H), 7.20 (dd, *J* = 8.0, 2.7 Hz, 2H), 6.82 (dd, *J* = 8.5, 2.4 Hz, 1H), 3.27 (s, 3H). ppm.

^{13}C NMR (126 MHz, $CDCl_3$, 298 K, δ): 166.0 (d, *J* = 256.4 Hz), 143.1, 134.6 (d, *J* = 3.1 Hz), 132.6, 131.5 (d, *J* = 9.6 Hz), 130.6, 127.1, 123.6, 123.0, 117.1 (d, *J* = 22.6 Hz), 46.1. ppm.

^{19}F NMR (471 MHz, $CDCl_3$, 298 K, δ): –103.6 (m) ppm.

HRMS-EI (m/z) calculated for $C_{13}H_{10}NOSFCl_2^+$ $[M]^+$, 316.9818; found, 316.9844; deviation: –1.7 ppm.

Synthesis of ((2,5-dichlorophenyl)imino)(4-fluorophenyl)(methyl)-λ^6-sulfanone (11) using recovered arene

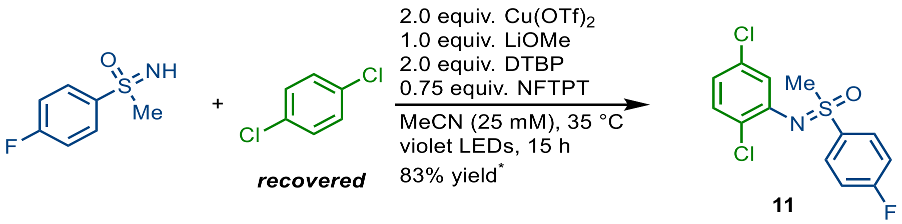

(Recovery of the arene, see Page S36) Under an ambient atmosphere, a 10 mL borosilicate vial equipped with a magnetic stir bar was charged with (4-fluorophenyl)(imino)(methyl)-λ^6-sulfanone (17.3 mg, 0.100 mmol, 1.00 equiv.). The vial was transferred into an anhydrous, N_2-filled glovebox. Under nitrogen atmosphere, $Cu(OTf)_2$ (0.200 mmol, 72.0 mg, 1.00 equiv.), LiOMe (3.7 mg, 0.10 mmol, 1.0 equiv.), and anhydrous MeCN (4.0 mL, *c* = 25 mM) were added into the vial, resulting in a blue suspension. The reaction mixture was stirred without irradiation for 30 min at 25 °C. Subsequently, 1-fluoro-2,4,6-trimethylpyridinium tetrafluoroborate (NFTPT) (17.0 mg, 0.075 mmol, 0.75 equiv.), recovered 1,4-dichlorobenzene (735 mg, 5.00 mmol, 50.0 equiv.), and 2,6-di-*tert*-butylpyridine (45.0 μL, 38.3 mg, 0.200 mmol, 2.00 equiv.) were added into the vial, resulting in a green suspension. The vial was sealed with a Teflon cap and taken out of the glovebox. The sealed vial was placed

5 cm away from two violet LEDs (Kessil PR160L-390 nm LEDs). The reaction mixture was stirred at a speed of 1000 rpm and irradiated for 15 h while maintaining the temperature at approximately 35 °C through cooling with a fan. After irradiation, the reaction mixture was evaporated under reduced pressure to remove all volatiles. EtOAc (15 mL) was added to the residue, and the resulting suspension was filtered through a silica pad, eluting with EtOAc (15 mL). The filtrate was concentrated under reduced pressure, then 1 mL $CDCl_3$ was added. 2-Fluorotoluene (11.0 mg, 11.0 μL, 1.0 equiv.) was added as the internal standard. An ^{19}F NMR was measured to determine the yield of ((2,5-dichlorophenyl)imino)(4-fluorophenyl)(methyl)-λ^6-sulfanone (**11**) (83%).

^{19}F NMR of the reaction mixture

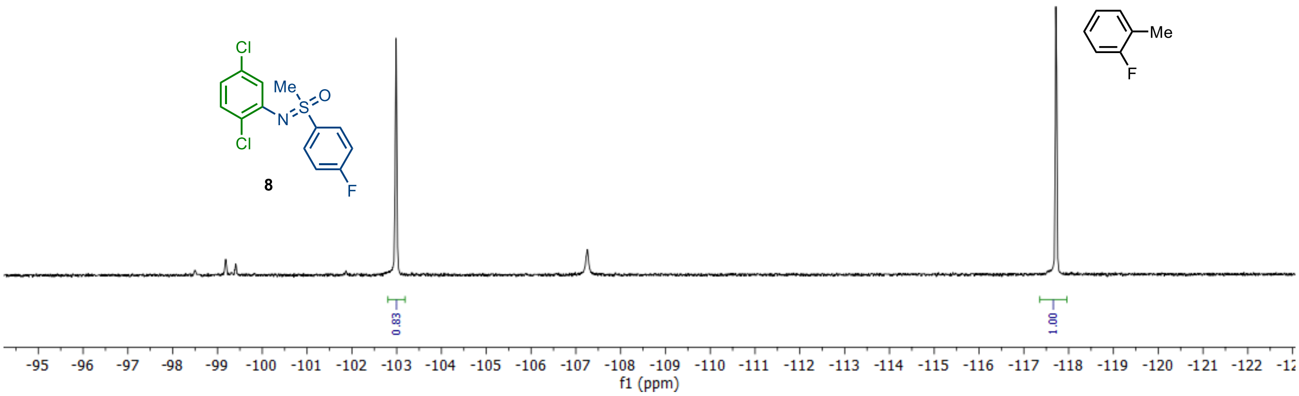

(Bromo-(trifluoromethoxy)phenyl)imino)(4-fluorophenyl)(methyl)-λ^6-sulfanone (12)

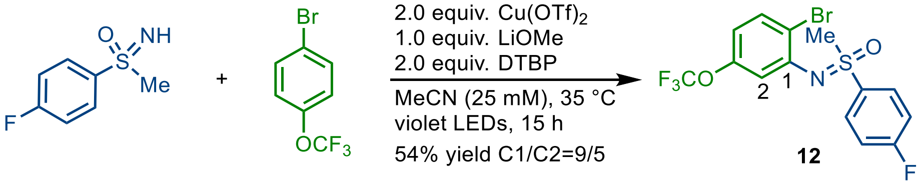

Under an ambient atmosphere, a 10 mL borosilicate vial equipped with a magnetic stir bar was charged with (4-fluorophenyl)(imino)(methyl)-λ^6-sulfanone (34.6 mg, 0.200 mmol, 1.00 equiv.). The vial was transferred into an anhydrous, N_2-filled glovebox. Under nitrogen atmosphere, $Cu(OTf)_2$ (144 mg, 0.400 mmol, 2.00 equiv.), LiOMe (7.5 mg, 0.20 mmol, 1.0 equiv.), and anhydrous MeCN (8.0 mL, c = 25 mM) were added into the vial, resulting in a blue suspension. The reaction mixture was stirred without irradiation for 30 min at 25 °C. Subsequently, 4-(trifluormethoxy)-bromobenzene (2.41 g, 1.48 mL, 10.0 mmol, 50.0 equiv.), and 2,6-di-*tert*-butylpyridine (90.0 μL, 76.5 mg, 0.400 mmol, 2.00 equiv.) were added into the vial, resulting in a green suspension. The vial was sealed with a Teflon cap and taken out of the glovebox. The sealed vial was placed 5 cm away from two violet LEDs (Kessil PR160L-390 nm LEDs). The reaction mixture was stirred at a speed of 1000 rpm and irradiated for 15 h while maintaining the temperature at approximately 35 °C through cooling with a fan. After irradiation, volatiles were removed from the reaction mixture under reduced pressure. DCM (15 mL) was added to the residue, and the precipitate was removed by filtration through a glass frit. The filtrate was concentrated under reduced pressure, and the residue was purified by flash column chromatography on silica gel (EA/pentane = 1/5, v/v) to yield the mixture of ((2-bromo-5-(trifluoromethoxy)phenyl)imino)(4-

fluorophenyl)(methyl)-λ6-sulfanone (**12-C1**) and ((5-bromo-2-(trifluoromethoxy)phenyl)imino)(4-fluorophenyl)(methyl)-λ6-sulfanone (**12-C2**) (45.3 mg, 109 μmol, 54%) as a colorless oil. The residue of mixture containing **12-C1** and **12-C2** was purified by another flash column chromatography on silica gel (EA/pentane = 1/6, v/v) to yield **12-C1** (25.1 mg) and **12-C2** (19.0 mg) seperately as colorless solid for characterizations. The ratio of **12-C1** and **12-C2** was determined by ^{19}F NMR.

((2-bromo-5-(trifluoromethoxy)phenyl)imino)(4-fluorophenyl)(methyl)-λ6-sulfanone (**12-C1**)

R*f* = 0.25 (EA/pentane = 1/6, v/v).

NMR Spectroscopy:

^{1}H NMR (500 MHz, $CDCl_3$, 298 K, δ): 8.05 (dd, *J* = 8.9, 5.0 Hz, 2H), 7.49 (d, *J* = 8.7 Hz, 1H), 7.23 (dd, *J* = 8.8, 8.2 Hz, 2H), 7.05 (d, *J* = 2.8 Hz, 1H), 6.70 – 6.61 (m, 1H), 3,29 (s, 3H) ppm.

^{13}C NMR (126 MHz, $CDCl_3$, 298 K, δ): 166.0 (d, *J* = 256.8 Hz), 148.6, 144.8, 134.4 (d, *J* = 3.1 Hz), 133.5, 131.6 (d, *J* = 9.5 Hz), 120.4 (q, *J* = 257.6 Hz), 117.2 (d, *J* = 22.7 Hz), 116.1, 115.5, 46.0. ppm.

^{19}F NMR (471 MHz, $CDCl_3$, 298 K, δ): –58.1 (s), –103.5 (m).ppm.

HRMS-ESI (m/z) calculated for $C_{14}H_{10}NO_2SF_4Br^+$ [M]$^+$, 410.9546; found, 410.9556; deviation: –2.6 ppm.

((5-bromo-2-(trifluoromethoxy)phenyl)imino)(4-fluorophenyl)(methyl)-λ6-sulfanone (**12-C2**)

R*f* = 0.30 (EA/pentane = 1/6, v/v).

NMR Spectroscopy:

^{1}H NMR (600 MHz, $CDCl_3$, 298 K, δ): 8.00 (dd, *J* = 8.9, 5.0 Hz, 2H), 7.32 (dd, *J* = 2.2, 0.4 Hz, 1H), 7.26 – 7.22 (m, 2H), 7.05 – 7.03 (m, 1H), 7.01 (dd, *J* = 8.6, 2.2 Hz, 1H), 3.26 (s, 3H).ppm.

^{13}C NMR (151 MHz, $CDCl_3$, 298 K, δ): 166.0 (d, *J* = 256.7 Hz), 141.9 (q, *J* = 1.7 Hz), 139.7, 134.7 (d, *J* = 3.1 Hz), 131.3 (d, *J* = 9.6 Hz), 126.8, 125.3, 123.8, 120.8 (q, *J* = 257.2 Hz), 120.4, 117.2 (d, *J* = 22.8 Hz), 46.1. ppm.

^{19}F NMR (565 MHz, $CDCl_3$, 298 K, δ):–57.4 (s), –107.4 (m) ppm.

HRMS-EI (m/z) calculated for $C_{14}H_{10}NO_2SF_4Br^+$ [M]$^+$, 410.9546; found, 410.9549; deviation: –0.8 ppm.

((Tert-butyl)phenyl)imino)(4-fluorophenyl)(methyl)-λ6-sulfanone (13)

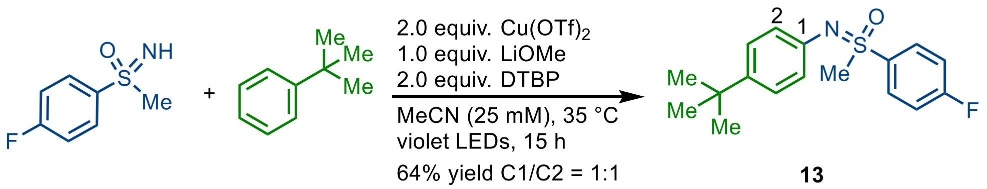

13

Under an ambient atmosphere, a 10 mL borosilicate vial equipped with a magnetic stir bar was charged with (4-fluorophenyl)(imino)(methyl)-λ6-sulfanone (34.6 mg, 0.200 mmol, 1.00 equiv.). The vial was transferred into an anhydrous, N_2-filled glovebox. Under nitrogen atmosphere, $Cu(OTf)_2$ (144 mg, 0.400 mmol, 2.00 equiv.),

LiOMe (7.5 mg, 0.20 mmol, 1.0 equiv.), and anhydrous MeCN (8.0 mL, *c* = 25 mM) were added into the vial, resulting in a blue suspension. The reaction mixture was stirred without irradiation for 30 min at 25 °C. Subsequently, tert-butylbenzene (1.72 g, 2.0 mL, 12.8 mmol, 64.0 equiv.), and 2,6-di-*tert*-butylpyridine (90.0 μL, 76.5 mg, 0.400 mmol, 2.00 equiv.) were added into the vial, resulting in a green suspension. The vial was sealed with a Teflon cap and taken out of the glovebox. The sealed vial was placed 5 cm away from two violet LEDs (Kessil PR160L-390 nm LEDs). The reaction mixture was stirred at a speed of 1000 rpm and irradiated for 15 h while maintaining the temperature at approximately 35 °C through cooling with a fan. After irradiation, volatiles were removed from the reaction mixture under reduced pressure. DCM (15 mL) was added to the residue, and the precipitate was removed by filtration through a glass frit. The filtrate was concentrated under reduced pressure, and the residue was purified by flash column chromatography on silica gel (EA/DCM = 1/100, v/v) to yield the mixture of (4-(tert-butyl)phenyl)imino)(4-fluorophenyl)(methyl)-λ^6-sulfanone (**13-C1**) and (3-(tert-butyl)phenyl)imino)(4-fluorophenyl)(methyl)-λ^6-sulfanone (**13-C2**) (39.1 mg, 128 μmol, 64%) as a colorless oil. The ratio of **13-C1** and **13-C2** was determined by ^{1}H NMR.

The following data were obtained from the mixture of two constitutional isomers.

R*f* = 0.15 (EA/DCM = 1/100).

NMR Spectroscopy:

^{1}H NMR (600 MHz, $CDCl_3$, 298 K, δ): 8.00 (ddd, *J* = 8.9, 5.0, 2.4 Hz, 4H), 7.20 – 7.15 (m, 2H), 7.19 (td, *J* = 8.6, 2.7 Hz, 4H), 7.06 (t, *J* = 7.7 Hz, 1H), 7.00 (s, 1H), 6.92 – 6.87 (m, 1H), 6.90 – 6.84 (m, 2H), 6.80 – 6.72 (m, 1H), 3.24 (s, 3H), 3.21 (s, 3H), 1.24 (s, 9H), 1.23 (s, 9H).ppm.

^{13}C NMR (151 MHz, $CDCl_3$, 298 K, δ): 165.64 (d, *J* = 255.4 Hz), 165.63 (d, *J* = 255.5 Hz), 152.1, 144.9, 144.3, 141.8, 136.2, 135.9, 131.5 (d, *J* = 9.4 Hz), 128.4, 125.8, 123.1, 121.2, 120.3, 119.2, 116.91 (d, *J* = 22.6 Hz), 116.88 (d, *J* = 22.6 Hz), 46.2, 46.2, 34.7, 34.1, 31.6, 31.3. ppm.

^{19}F NMR (565 MHz, $CDCl_3$, 298 K, δ): –104.8 (m) ppm.

HRMS-EI (m/z) calculated for $C_{17}H_{20}NOSF^+$ [M]$^+$, 305.1244; found, 305.1249; deviation: –1.8 ppm. Peak 1

HRMS-EI (m/z) calculated for $C_{17}H_{20}NOSF^+$ [M]$^+$, 305.1244; found, 305.1246; deviation: –0.7 ppm. Peak 2

(((4-Fluorophenyl)(methyl)(oxo)-λ^6-sulfaneylidene)amino)phenyl trifluoromethanesulfonate (14)

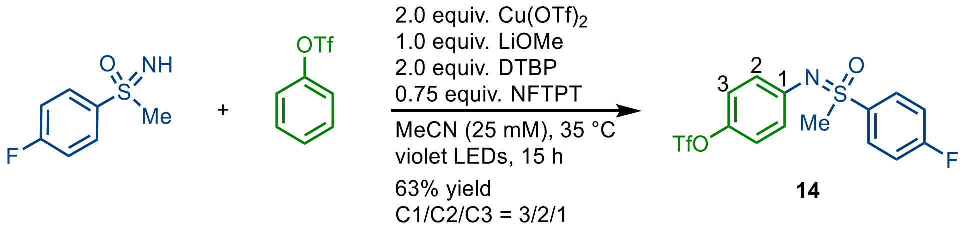

14

Under an ambient atmosphere, a 10 mL borosilicate vial equipped with a magnetic stir bar was charged with (4-fluorophenyl)(imino)(methyl)-λ^6-sulfanone (34.6 mg, 0.200 mmol, 1.00 equiv.). The vial was transferred into an anhydrous, N_2-filled glovebox. Under nitrogen atmosphere, $Cu(OTf)_2$ (144 mg, 0.400 mmol, 2.00 equiv.),

LiOMe (7.5 mg, 0.20 mmol, 1.0 equiv.), and anhydrous MeCN (8.0 mL, c = 25 mM) were added into the vial, resulting in a blue suspension. The reaction mixture was stirred without irradiation for 30 min at 25 °C. Subsequently, 1-fluoro-2,4,6-trimethylpyridinium tetrafluoroborate (NFTPT) (34.0 mg, 0.15 mmol, 0.75 equiv.), phenyltriflate (2.26g, 1.62 mL, 10.0 mmol, 50.0 equiv.) and 2,6-di-*tert*-butylpyridine (90.0 µL, 76.5 mg, 0.400 mmol, 2.00 equiv.) were added into the vial, resulting in a green suspension. The vial was sealed with a Teflon cap and taken out of the glovebox. The sealed vial was placed 5 cm away from two violet LEDs (Kessil PR160L-390 nm LEDs). The reaction mixture was stirred at a speed of 1000 rpm and irradiated for 15 h while maintaining the temperature at approximately 35 °C through cooling with a fan. After irradiation, volatiles were removed from the reaction mixture under reduced pressure. DCM (15 mL) was added to the residue, and the precipitate was removed by filtration through a glass frit. The filtrate was concentrated under reduced pressure, and the residue was purified by flash column chromatography on silica gel (EA/pentane 1/1) to yield the mixture of 4-(((4-fluorophenyl)(methyl)(oxo)-λ^6-sulfaneylidene)amino)phenyl trifluoromethanesulfonate (**14-C1**), 3-(((4-fluorophenyl)(methyl)(oxo)-λ^6-sulfaneylidene)amino)phenyl trifluoromethanesulfonate (**14-C2**), and 2-(((4-fluorophenyl)(methyl)(oxo)-λ^6-sulfaneylidene)amino)phenyl trifluoromethanesulfonate (**14-C3**) (50.0 mg, 126 µmol, 63%) as a colorless oil. The residue of mixture containing **14-C1**, **14-C2,** and **14-C3** was purified by another flash column chromatography on silica gel (EA/pentane = 1/4 to 2/3, v/v) to yield **14-C1** (23.0 mg), **14-C2** (13.6 mg) and **14-C3** (6.5 mg) seperately as colorless solid for characterizations. The ratio of **14-C1**, **14-C2, and 14-C3** was determined by ^{19}F NMR.

4-(((4-fluorophenyl)(methyl)(oxo)-λ^6-sulfaneylidene)amino)phenyl trifluoromethanesulfonate (**14-C1**)

R*f* = 0.2 (EA/pentane = 2/3, v/v).

NMR Spectroscopy:

^{1}H NMR (500 MHz, $CDCl_3$, 298 K, δ): 7.96 (dd, J = 8.9, 5.0 Hz, 2H), 7.23 (dd, J = 8.9, 8.2 Hz, 2H), 7.06 – 6.95 (m, 4H), 3.25 (s, 3H). ppm.

^{13}C NMR (126 MHz, $CDCl_3$, 298 K, δ): 165.9 (d, J = 256.4 Hz), 145.3, 144.3, 134.8 (d, J = 3.0 Hz), 131.5 (d, J = 9.5 Hz), 124.3, 122.0, 118.8 (q, J = 320.9 Hz), 117.2 (d, J = 22.7 Hz), 46.5. ppm.

^{19}F NMR (471 MHz, $CDCl_3$, 298 K, δ): –72.9 (s), –103.6 (m) ppm.

HRMS-EI (m/z) calculated for $C_{14}H_{11}NO_4S_2F_4^+$ $[M+H]^+$, 397.0060; found, 397.0063; deviation: –0.8 ppm.

3-(((4-fluorophenyl)(methyl)(oxo)-λ^6-sulfaneylidene)amino)phenyl trifluoromethanesulfonate (**14-C2**)

R*f* = 0.25 (EA/pentane = 2/3, v/v).

NMR Spectroscopy:

^{1}H NMR (500 MHz, $CDCl_3$, 298 K, δ): 7.95 (dd, J = 8.9, 5.0 Hz, 2H), 7.22 (dd, J = 8.9, 8.1 Hz, 2H), 7.16 (t, J = 8.2 Hz, 1H), 6.98 (ddd, J = 8.2, 2.0, 0.9 Hz, 1H), 6.89 (t, J = 2.3 Hz, 1H), 6.78 (ddd, J = 8.4, 2.5, 0.9 Hz, 1H), 3.27 (s, 3H). ppm.

^{13}C NMR (126 MHz, $CDCl_3$, 298 K, δ): 166.0 (d, J = 256.8 Hz), 150.1, 147.2, 134.5 (d, J = 3.4 Hz), 131.5

(d, J = 9.5 Hz), 130.3, 123.0, 118.8 (q, J = 320.8 Hz), 117.2 (d, J = 22.7 Hz), 115.2 (d, J = 219.0 Hz), 46.4. ppm.

^{19}F NMR (471 MHz, $CDCl_3$, 298 K, δ): –72.9 (s), –103.6 (s). ppm.

HRMS-EI (m/z) calculated for $C_{14}H_{11}NO_4S_2F_4^+$ $[M+H]^+$, 397.0060; found, 397.0068; deviation: –2.1 ppm.

2-(((4-fluorophenyl)(methyl)(oxo)-λ^6-sulfaneylidene)amino)phenyl trifluoromethanesulfonate (**14-C3**)

R*f* = 0.7 (EA/pentane = 2/3, v/v).

NMR Spectroscopy:

^{1}H NMR (500 MHz, $CDCl_3$, 298 K, δ): 8.10 (dd, J = 8.9, 5.0 Hz, 2H), 7.24 (dd, J = 8.9, 8.2 Hz, 2H), 7.13 (dd, J = 8.2, 1.4 Hz, 1H), 7.11 (dd, J = 8.1, 1.7 Hz, 1H), 7.06 (td, J = 7.7, 1.5 Hz, 1H), 6.90 (ddd, J = 8.1, 7.3, 1.7 Hz, 1H), 3.30 (s, 3H). ppm.

^{13}C NMR (151 MHz, $CDCl_3$, 298 K, δ): 166.0 (d, J = 256.5 Hz), 143.8, 138.7, 134.5 (d, J = 3.1 Hz), 131.5 (d, J = 9.6 Hz), 128.8, 123.1, 122.2, 121.8, 118.9 (d, J = 320.0 Hz), 117.2 (d, J = 22.7 Hz), 46.1.ppm.

^{19}F NMR (471 MHz, $CDCl_3$, 298 K, δ): –74.6 (s), –103.6 (s) ppm.

HRMS-EI (m/z) calculated for $C_{14}H_{11}NO_4S_2F_4^+$ $[M+H]^+$, 397.0060; found, 397.0065; deviation: –1.2 ppm.

((Chlorophenyl)imino)(4-fluorophenyl)(methyl)-λ^6-sulfanone (15)

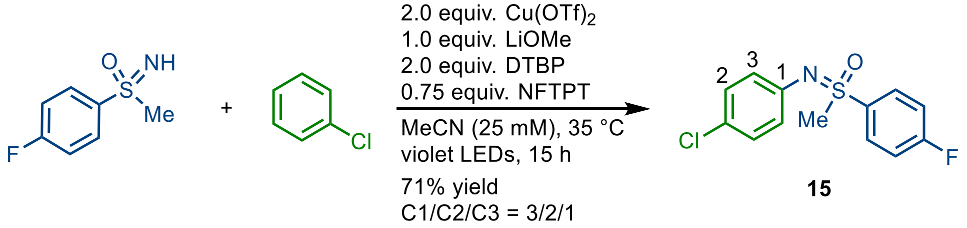

15

Under an ambient atmosphere, a 10 mL borosilicate vial equipped with a magnetic stir bar was charged with (4-fluorophenyl)(imino)(methyl)-λ^6-sulfanone (34.6 mg, 0.200 mmol, 1.00 equiv.). The vial was transferred into an anhydrous, N_2-filled glovebox. Under nitrogen atmosphere, $Cu(OTf)_2$ (144 mg, 0.400 mmol, 2.00 equiv.), LiOMe (7.5 mg, 0.20 mmol, 1.0 equiv.), and anhydrous MeCN (8.0 mL, c = 25 mM) were added into the vial, resulting in a blue suspension. The reaction mixture was stirred without irradiation for 30 min at 25 °C. Subsequently, 1-fluoro-2,4,6-trimethylpyridinium tetrafluoroborate (NFTPT) (34.0 mg, 0.15 mmol, 0.75 equiv.), chlorobenzene (2.25 g, 2.00 mL, 20.0 mmol, 100 equiv.), and 2,6-di-*tert*-butylpyridine (90.0 μL, 76.5 mg, 0.400 mmol, 2.00 equiv.) were added into the vial, resulting in a green suspension. The vial was sealed with a Teflon cap and taken out of the glovebox. The sealed vial was placed 5 cm away from two violet LEDs (Kessil PR160L-390 nm LEDs). The reaction mixture was stirred at a speed of 1000 rpm and irradiated for 15 h while maintaining the temperature at approximately 35 °C through cooling with a fan. After irradiation, volatiles were removed from the reaction mixture under reduced pressure. DCM (15 mL) was added to the residue, and the precipitate was removed by filtration through a glass frit. The filtrate was concentrated under reduced pressure, and the residue was purified by flash column chromatography on silica gel silica gel (EA/pentane = 1/2, v/v) to yield the

mixture of ((4-chlorophenyl)imino)(4-fluorophenyl)(methyl)-λ^6-sulfanone (**15-C1**), ((2-chlorophenyl)imino)(4-fluorophenyl)(methyl)-λ^6-sulfanone (**15-C2**), and ((3-chlorophenyl)imino)(4-fluorophenyl)(methyl)-λ^6-sulfanone (**15-C3**) (40.0 mg, 141 µmol, 71%) as a slightly yellow oil. The residue of mixture containing **15-C1**, **15-C2**, and **15-C3** was purified by another flash column chromatography on silica gel (EA/pentane = 1/4 to 1/2, v/v) to yield **15-C1** (15.7 mg), **15-C2** (12.4 mg) and **15-C3** (7.0 mg) seperately as colorless solid for characterizations. The ratio of **15-C1**, **15-C2, and 15-C3** was determined by ^{19}F NMR.

((4-chlorophenyl)imino)(4-fluorophenyl)(methyl)-λ^6-sulfanone (**15-C1**)

R*f* = 0.20 (EA/pentane = 1/2, v/v).

NMR Spectroscopy:

^{1}H NMR (500 MHz, $CDCl_3$, 298 K, δ): 7.95 (dd, *J* = 8.9, 5.0 Hz, 2H), 7.20 (dd, *J* = 8.9, 8.2 Hz, 2H), 7.07 (d, *J* = 8.8 Hz, 2H), 6.92 (d, *J* = 8.8 Hz, 2H), 3.24 (s, 3H). ppm.

^{13}C NMR (151 MHz, $CDCl_3$, 298 K, δ): 165.8 (d, *J* = 256.2 Hz), 143.5, 134.9 (d, *J* = 3.0 Hz), 131.6 (d, *J* = 9.5 Hz), 129.2, 127.2, 124.5, 117.1 (d, *J* = 22.5 Hz), 46.3. ppm.

^{19}F NMR (471 MHz, $CDCl_3$, 298 K, δ): –104.1 (m) ppm.

HRMS-EI (m/z) calculated for $C_{13}H_{11}NOSFCl^+$ $[M]^+$, 283.0228; found, 283.0239; deviation: –1.9 ppm.

((2-chlorophenyl)imino)(4-fluorophenyl)(methyl)-λ^6-sulfanone (**15-C2**)

R*f* = 0.75 (EA/pentane = 1/2, v/v).

NMR Spectroscopy:

^{1}H NMR (500 MHz, $CDCl_3$, 298 K, δ): 8.07 (dd, *J* = 8.9, 5.0 Hz, 2H), 7.32 (dd, *J* = 7.9, 1.5 Hz, 2H), 7.25 – 7.20 (m, 1H), 7.20 – 7.18 (m, 1H), 7.00 (td, *J* = 7.7, 1.6 Hz, 1H), 6.85 (td, *J* = 7.7, 1.5 Hz, 1H), 3.26 (s, 3H). ppm.

^{13}C NMR (126 MHz, $CDCl_3$, 298 K, δ): 165.9 (d, *J* = 256.2 Hz), 142.0, 135.1 (d, *J* = 3.1 Hz), 131.6 (d, *J* = 9.8 Hz), 130.1, 128.7, 127.4, 124.2, 123.2, 117.0 (d, *J* = 22.5 Hz), 45.9. ppm.

^{19}F NMR (471 MHz, $CDCl_3$, 298 K, δ): –104.1 (m) ppm.

HRMS-EI (m/z) calculated for $C_{13}H_{11}NOSFCl^+$ $[M]^+$, 283.0228; found, 283.0234; deviation: –2.1 ppm.

((3-chlorophenyl)imino)(4-fluorophenyl)(methyl)-λ^6-sulfanone (**15-C3**)

R*f* = 0.25 (EA/pentane = 1/2, v/v).

NMR Spectroscopy:

^{1}H NMR (500 MHz, $CDCl_3$, 298 K, δ): 7.97 (dd, *J* = 8.9, 5.0 Hz, 2H), 7.22 (dd, *J* = 8.9, 8.2 Hz, 2H), 7.07 – 6.99 (m, 2H), 6.89 – 6.84 (m, 2H), 3.25 (s, 3H).ppm.

^{13}C NMR (151 MHz, $CDCl_3$, 298 K, δ): 165.8 (d, *J* = 256.3 Hz), 146.3, 134.9 (d, *J* = 3.1 Hz), 131.5 (d, *J* = 9.4 Hz), 130.0, 123.5, 122.1, 121.4, 117.1 (d, *J* = 22.7 Hz), 46.4. ppm.

^{19}F NMR (471 MHz, $CDCl_3$, 298 K, δ): –103.9 (m) ppm.

HRMS-EI (m/z) calculated for $C_{13}H_{11}NOSFCl^+$ $[M]^+$, 283.0228; found, 283.0233; deviation: –1.6 ppm.

((2,5-Dibromophenyl)imino)(4-fluorophenyl)(methyl)-λ^6-sulfanone (16)

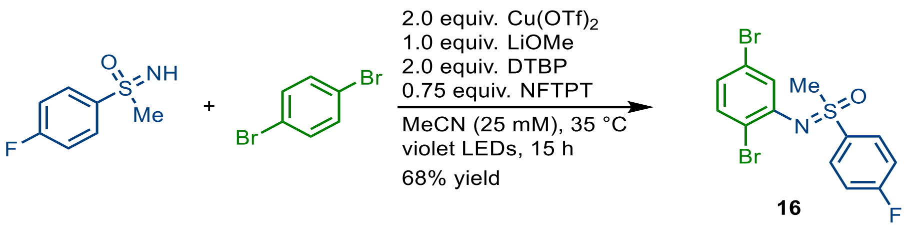

Under an ambient atmosphere, a 10 mL borosilicate vial equipped with a magnetic stir bar was charged with (4-fluorophenyl)(imino)(methyl)-λ^6-sulfanone (34.6 mg, 0.200 mmol, 1.00 equiv.).The vial was transferred into an anhydrous, N_2-filled glovebox. Under nitrogen atmosphere, $Cu(OTf)_2$ (144 mg, 0.400 mmol, 2.00 equiv.), LiOMe (7.5 mg, 0.20 mmol, 1.0 equiv.), and anhydrous MeCN (8.0 mL, *c* = 25 mM) were added into the vial, resulting in a blue suspension. The reaction mixture was stirred without irradiation for 30 min at 25 °C. Subsequently, 1-fluoro-2,4,6-trimethylpyridinium tetrafluoroborate (NFTPT) (34.0 mg, 0.15 mmol, 0.75 equiv.), 1,4-dibromobenzene (2.35 g, 10.0 mmol, 50.0 equiv.) and 2,6-di-*tert*-butylpyridine (90.0 µL, 76.5 mg, 0.400 mmol, 2.00 equiv.) were added into the vial, resulting in a green suspension. The vial was sealed with a Teflon cap and taken out of the glovebox. The sealed vial was placed 5 cm away from two violet LEDs (Kessil PR160L-390 nm LEDs). The reaction mixture was stirred at a speed of 1000 rpm and irradiated for 15 h while maintaining the temperature at approximately 35 °C through cooling with a fan. After irradiation, volatiles were removed from the reaction mixture under reduced pressure. DCM (15 mL) was added to the residue, and the precipitate was removed by filtration through a glass frit. The filtrate was concentrated under reduced pressure, and the residue was purified by flash column chromatography on silica gel (EA/pentane = 1/5, v/v) to yield ((2,5-dibromophenyl)imino)(4-fluorophenyl)(methyl)-λ6-sulfanone (**16**) (55.5 mg, 136 µmol, 68%) as a colorless solid.

R*f* = 0.30 (EA/pentane = 1/5, v/v).

NMR Spectroscopy:

^{1}H NMR (500 MHz, $CDCl_3$, 298 K, δ): 8.07 (dd, *J* = 8.9, 5.0 Hz, 2H), 7.34 (dd, *J* = 5.4, 3.1 Hz, 2H), 7.23 (dd, *J* = 8.9, 8.2 Hz, 2H), 6.90 (dd, *J* = 8.5, 2.3 Hz, 1H), 3.26 (s, 3H).ppm.

^{13}C NMR (126 MHz, $CDCl_3$, 298 K, δ): 166.0 (d, *J* = 256.4 Hz), 144.8, 134.6 (d, *J* = 3.0 Hz), 134.0, 131.5 (d, *J* = 9.5 Hz), 126.3, 126.1, 121.3, 118.2, 117.1 (d, *J* = 22.6 Hz), 46.0. ppm.

^{19}F NMR (471 MHz, $CDCl_3$, 298 K, δ): –103.5 (m) ppm.

HRMS-EI (m/z) calculated for $C_{13}H_{10}NOSFBr_2^+$ $[M]^+$, 404.8828; found, 404.8836; deviation: –1.7 ppm.

((5-(Tert-butyl)-2-chlorophenyl)imino)(4-fluorophenyl)(methyl)-λ⁶-sulfanone (17)

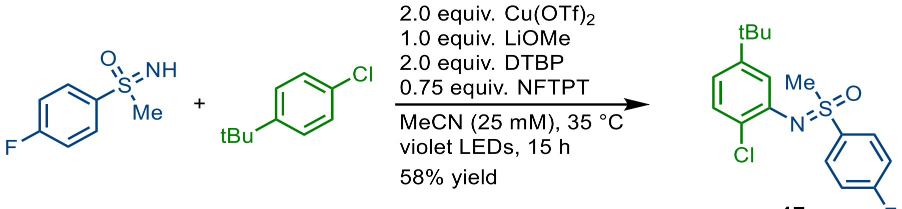

17

Under an ambient atmosphere, a 10 mL borosilicate vial equipped with a magnetic stir bar was charged with (4-fluorophenyl)(imino)(methyl)-λ⁶-sulfanone (34.6 mg, 0.200 mmol, 1.00 equiv.). The vial was transferred into an anhydrous, N_2-filled glovebox. Under nitrogen atmosphere, $Cu(OTf)_2$ (144 mg, 0.400 mmol, 2.00 equiv.), LiOMe (7.5 mg, 0.20 mmol, 1.0 equiv.), and anhydrous MeCN (8.0 mL, *c* = 25 mM) were added into the vial, resulting in a blue suspension. The reaction mixture was stirred without irradiation for 30 min at 25 °C. Subsequently, 1-fluoro-2,4,6-trimethylpyridinium tetrafluoroborate (NFTPT) (34.0 mg, 0.15 mmol, 0.75 equiv.), 1-tert-butyl-4-chlorobenzene (1.68 g, 1.43 mL, 10.0 mmol, 50.0 equiv.), and 2,6-di-*tert*-butylpyridine (90.0 µL, 76.5 mg, 0.400 mmol, 2.00 equiv.) were added into the vial, resulting in a green suspension. The vial was sealed with a Teflon cap and taken out of the glovebox. The sealed vial was placed 5 cm away from two violet LEDs (Kessil PR160L-390 nm LEDs). The reaction mixture was stirred at a speed of 1000 rpm and irradiated for 15 h while maintaining the temperature at approximately 35 °C through cooling with a fan. After irradiation, volatiles were removed from the reaction mixture under reduced pressure. DCM (15 mL) was added to the residue, and the precipitate was removed by filtration through a glass frit. The filtrate was concentrated under reduced pressure, and the residue was purified by flash column chromatography on silica gel (EA/pentane = 1/5, v/v) to yield ((5-(tert-butyl)-2-chlorophenyl)imino)(4-fluorophenyl)(methyl)-λ⁶-sulfanone (**17**) (39.5 mg, 116 µmol, 58%) as a colorless solid.

R*f* = 0.30 (EA/pentane = 1/5, v/v).

NMR Spectroscopy:

^{1}H NMR (500 MHz, $CDCl_3$, 298 K, δ): 8.07 (dd, *J* = 8.9, 5.1 Hz, 2H), 7.28 – 7.15 (m, 4H), 6.87 (dd, *J* = 8.4, 2.3 Hz, 1H), 3.26 (s, 3H), 1.17 (s, 9H). ppm.

^{13}C NMR (126 MHz, $CDCl_3$, 298 K, δ): 165.7 (d, *J* = 255.7 Hz), 150.6, 141.2, 135.1 (d, *J* = 3.3 Hz), 131.5 (d, *J* = 9.6 Hz), 129.1, 125.5, 121.6, 120.2, 116.7 (d, *J* = 22.4 Hz), 45.5, 34.4, 31.2. ppm.

^{19}F NMR (471 MHz, $CDCl_3$, 298 K, δ): –104.4 (m) ppm.

HRMS-EI (m/z) calculated for $C_{17}H_{19}NOSFCl^+$ $[M]^+$, 339.0854; found, 339.0860; deviation: –1.8 ppm.

(((1-Chloro-2-methylpropan-2-yl)phenyl)imino)(4-fluorophenyl)(methyl)-λ⁶-sulfanone (18)

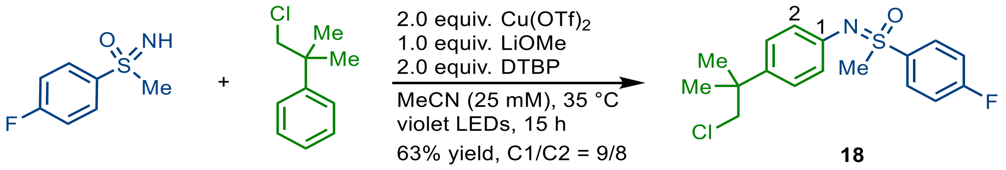

18

In a nitrogen-filled glovebox, a 10 mL borosilicate vial equipped with a magnetic stir bar was charged with and (4-fluorophenyl)(imino)(methyl)-λ6-sulfanone (34.6 mg, 0.200 mmol, 1.00 equiv.), $Cu(OTf)_2$ (145 mg, 0.400 mmol, 2.00 equiv.), LiOMe (7.5 mg, 0.20 mmol, 1.0 equiv.), 2,6-di-tert-butylpyridine (76.5 mg, 0.400 mmol, 2.00 equiv.) and 1-chloro-2-methyl-2-phenylpropane (1.69 g, 10.0 mmol, 50 equiv.), anhydrous MeCN (8.0 mL, c = 25 mM) was then added into the vial. The vial was sealed with a Teflon cap and placed 5 cm away from two violet LEDs (Kessil PR160L-390 nm LEDs). The reaction mixture was irradiated for 15 h while maintaining the temperature at approximately 35 °C through cooling with a fan. After irradiation, the reaction mixture was evaporated under reduced pressure to remove all volatiles. The residue was purified by chromatography on silica gel (EA/DCM = 1/50, v/v) to yield a mixture of ((4-(1-chloro-2-methylpropan-2-yl)phenyl)imino)(4-fluorophenyl)(methyl)-λ6-sulfanone (**18-C1**) and ((3-(1-chloro-2-methylpropan-2-yl)phenyl)imino)(4-fluorophenyl)(methyl)-λ6-sulfanone (**18-C2**) (43.0 mg, 127 μmol, 63%) as a colorless oil. The ratio was determined by ^{1}H NMR.

The following data were obtained from the mixture of two constitutional isomers:

R*f* = 0.19 (DCM).

NMR Spectroscopy:

^{1}H NMR (500 MHz, $CDCl_3$, 298 K, δ): 8.03–7.95 (m, 2H), 7.24–7.17 (m, 2H), 7.15–7.11 (m, 1.08H), 7.08 (t, *J* = 7.9 Hz, 0.48H), 7.01 (t, *J* = 2.1 Hz, 0.46 H), 6.98–6.93 (m, 1.06H), 6.92–6.84 (m, 0.92H), 3.54 (s, 1.94H), 3.27 (s, 1.34H), 3.25 (s, 1.62H), 1.34 (m, 5.95H).ppm.

^{13}C NMR (126 MHz, $CDCl_3$, 298 K, δ): 165.8 (d, *J* = 256.0 Hz), 147.3, 144.1, 142.3, 139.9, 135.1, 134.8, 131.65 (d, *J* = 9.4 Hz), 131.60 (d, *J* = 9.4 Hz), 129.0, 126.8, 123.1, 121.6, 121.2, 120.0, 117.11 (d, *J* = 22.6 Hz), 117.07 (d, *J* = 22.6 Hz), 56.6, 56.5, 46.2, 46.1, 39.8, 39.3, 26.43, 26.41, 26.35 ppm.

^{19}F NMR (471 MHz, $CDCl_3$, 298 K, δ): –104.4 (m) ppm.

HRMS-EI (m/z) calculated for $C_{17}H_{19}NOSFCl^+$ $[M]^+$, 339.0854; found, 339.0858; deviation: –1.0 ppm.

(4-Fluorophenyl)(methyl)((trifluoromethoxy)phenyl)imino)-λ6-sulfanone (19)

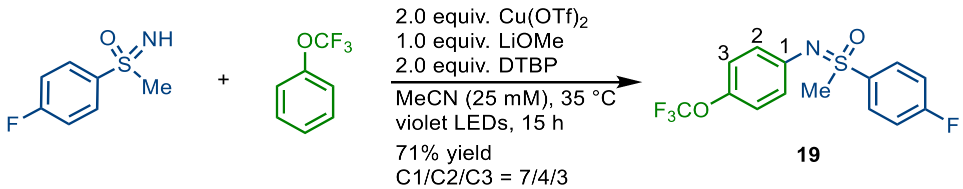

19

In a nitrogen-filled glovebox, a 10 mL borosilicate vial equipped with a magnetic stir bar was charged with (4-fluorophenyl)(imino)(methyl)-λ6-sulfanone (34.6 mg, 0.200 mmol, 1.00 equiv.), LiOMe (7.5 mg, 0.20 mmol, 1.0 equiv.), $Cu(OTf)_2$ (145 mg, 0.400 mmol, 2.00 equiv.), 2,6-di-tert-butylpyridine (76.5 mg, 0.400 mmol, 2.00 equiv.) and trifuoromethoxybenzene (1.30 mL, 1.62 g, 10.0 mmol, 50.0 equiv.), anhydrous MeCN (8.0 mL, c = 25 mM) was then added into the vial. The vial was sealed with a Teflon cap and placed 5 cm away from two violet LEDs (Kessil PR160L-390 nm LEDs). The reaction mixture was irradiated for 15 h while maintaining the temperature

at approximately 35 °C through cooling with a fan. After irradiation, the reaction mixture was evaporated under reduced pressure to remove volatiles. The residue was purified by chromatography on silica gel (DCM) to yield a mixture of (4-fluorophenyl)(methyl)((2/3/4-(trifluoromethoxy)phenyl)imino)-λ^6-sulfanone (**19**) as a colorless oil. Then the mixture was further separated by preparative TLC to yield (4-fluorophenyl)(methyl)((4-(trifluoromethoxy)phenyl)imino)-λ^6-sulfanone (**19-C1**) (23.7 mg, 71.0 μmol, 35%) as a colorless oil, (4-fluorophenyl)(methyl)((3-(trifluoromethoxy)phenyl)imino)-λ^6-sulfanone (**19-C2**) (13.5 mg, 41.0 μmol, 20%) as a colorless oil, and (4-fluorophenyl)(methyl)((2-(trifluoromethoxy)phenyl)imino)-λ^6-sulfanone (**19-C3**) (10.1 mg, 30.0 μmol, 15%) as a colorless oil.

(4-Fluorophenyl)(methyl)((4-(trifluoromethoxy)phenyl)imino)-λ^6-sulfanone (**19-C1**):

R*f* = 0.25 (DCM).

NMR Spectroscopy:

^{1}**H NMR** (600 MHz, $CDCl_3$, 298 K, δ): 8.00–7.94 (m, 2H), 7.24–7.20 (m, 2H), 6.98 (s, 4H), 3.27 (s, 3H) ppm.

^{13}**C NMR** (150 MHz, $CDCl_3$, 298 K, δ): 165.9 (d, *J* = 256.4 Hz), 144.2, 143.3, 134.9, 131.6 (d, *J* = 9.5 Hz), 124.2, 122.0, 120.7 (q, *J* = 256.1 Hz), 117.2 (d, *J* = 22.7 Hz), 46.3 ppm.

^{19}**F NMR** (565 MHz, $CDCl_3$, 298 K, δ): –58.2 (s), –103.8 (m) ppm.

HRMS-EI (m/z) calculated for $C_{14}H_{11}NO_2SF_4^+$ $[M]^+$, 333.0441; found, 333.0444; deviation: –0.9 ppm.

(4-Fluorophenyl)(methyl)((3-(trifluoromethoxy)phenyl)imino)-λ^6-sulfanone (**19-C2**):

R*f* = 0.32 (DCM).

NMR Spectroscopy:

^{1}**H NMR** (600 MHz, $CDCl_3$, 298 K, δ): 7.99–7.95 (m, 2H), 7.24–7.19 (m, 2H), 7.11 (t, *J* = 8.1 Hz, 1H), 6.92–6.88 (m, 1H), 6.87–6.84 (m, 1H), 6.75–6.71 (m, 1H), 3.27 (s, 3H) ppm.

^{13}**C NMR** (150 MHz, $CDCl_3$, 298 K, δ): 165.9 (d, *J* = 256.4 Hz), 149.9 (d, *J* = 1.7 Hz), 146.4, 134.7, 131.6 (d, *J* = 9.5 Hz), 130.0, 121.5, 120.5 (q, *J* = 256.9 Hz), 117.2 (d, *J* = 22.7 Hz), 116.1, 114.3, 46.3 ppm.

^{19}**F NMR** (565 MHz, $CDCl_3$, 298 K, δ): –57.8 (s), –103.8 (m) ppm.

HRMS-EI (m/z) calculated for $C_{14}H_{11}NO_2SF_4^+$ $[M]^+$, 333.0441; found, 333.0444; deviation: –0.8 ppm.

(4-Fluorophenyl)(methyl)((2-(trifluoromethoxy)phenyl)imino)-λ^6-sulfanone (**19-C3**):

R*f* = 0.36 (DCM).

^{1}**H NMR** (600 MHz, $CDCl_3$, 298 K, δ): 8.10–7.92 (m, 2H), 7.25–7.20 (m, 2H), 7.20–7.17 (m, 1H), 7.16 (dd, *J* = 8.0, 1.6 Hz, 1H), 7.04–7.00 (m, 1H), 6.91 (ddd, *J* = 8.1, 7.4, 1.6 Hz, 1H), 3.25 (s, 3H) ppm.

^{13}**C NMR** (150 MHz, $CDCl_3$, 298 K, δ): 165.9 (d, *J* = 256.1 Hz), 142.8 (d, *J* = 1.6 Hz), 138.0, 135.19, 135.17, 131.4 (d, *J* = 9.6 Hz), 127.5, 124.4, 122.5, 121.0 (q, *J* = 256.4 Hz), 117.0 (d, *J* = 22.7 Hz), 45.9 ppm.

^{19}F NMR (565 MHz, $CDCl_3$, 298 K, δ): –57.2 (s), –104.1 (m) ppm.

HRMS-EI (m/z) calculated for $C_{14}H_{11}NO_2SF_4^+$ $[M]^+$, 333.0441; found, 333.0445; deviation: –1.1 ppm.

((Bromophenyl)imino)(4-fluorophenyl)(methyl)-λ^6-sulfanone (20)

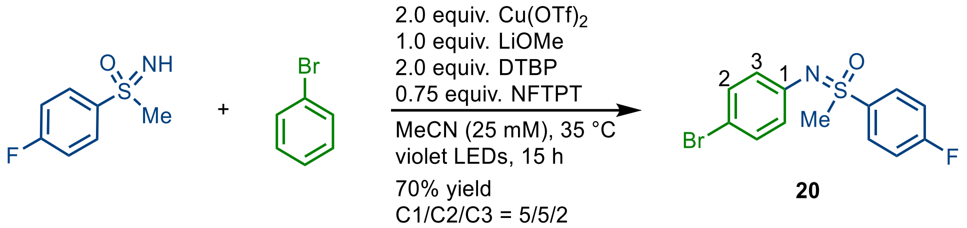

20

Under an ambient atmosphere, a 10 mL borosilicate vial equipped with a magnetic stir bar was charged with (4-fluorophenyl)(imino)(methyl)-λ^6-sulfanone (34.6 mg, 0.200 mmol, 1.00 equiv.). The vial was transferred into an anhydrous, N_2-filled glovebox. Under nitrogen atmosphere, $Cu(OTf)_2$ (144 mg, 0.400 mmol, 2.00 equiv.), LiOMe (7.5 mg, 0.20 mmol, 1.0 equiv.), and anhydrous MeCN (8.0 mL, *c* = 25 mM) were added into the vial, resulting in a blue suspension. The reaction mixture was stirred without irradiation for 30 min at 25 °C. Subsequently, 1-fluoro-2,4,6-trimethylpyridinium tetrafluoroborate (NFTPT) (34.0 mg, 0.15 mmol, 0.75 equiv.), bromobenzene (3.14 g, 2.10 mL, 20.0 mmol, 100 equiv.), and 2,6-di-*tert*-butylpyridine (90.0 µL, 76.5 mg, 0.400 mmol, 2.00 equiv.) were added into the vial, resulting in a green suspension. The vial was sealed with a Teflon cap and taken out of the glovebox. The sealed vial was placed 5 cm away from two violet LEDs (Kessil PR160L-390 nm LEDs). The reaction mixture was stirred at a speed of 1000 rpm and irradiated for 15 h while maintaining the temperature at approximately 35 °C through cooling with a fan. After irradiation, volatiles were removed from the reaction mixture under reduced pressure. DCM (15 mL) was added to the residue, and the precipitate was removed by filtration through a glass frit. The filtrate was concentrated under reduced pressure, and the residue was purified by flash column chromatography on silica gel silica gel (EA/pentane = 1/2, v/v) to yield the mixture of ((4-bromophenyl)imino)(4-fluorophenyl)(methyl)-λ^6-sulfanone (**20-C1**), ((2-bromophenyl)imino)(4-fluorophenyl)(methyl)-λ^6-sulfanone (**20-C2**), and ((3-bromophenyl)imino)(4-fluorophenyl)(methyl)-λ^6-sulfanone (**20-C3**) (46.1 mg, 114 mmol, 70%) as a yellow oil. The residue of mixture containing **20-C1** , **20-C2** , and **20-C3** was purified by another flash column chromatography on silica gel (EA/pentane = 1/4 to 1/2 to 1/1, v/v) to yield **20-C1** (16.0 mg), **20-C2** (18.1 mg) and **20-C3** (5.6 mg) seperately as colorless solid for characterizations. The ratio of **20-C1**, **20-C2,** and **20-C3** was determined by ^{19}F NMR.

((4-bromophenyl)imino)(4-fluorophenyl)(methyl)-λ^6-sulfanone (**20-C1**)

R*f* = 0.20 (EA/pentane = 1/2, v/v).

NMR Spectroscopy:

^{1}H NMR (500 MHz, $CDCl_3$, 298 K, δ): 7.95 (dd, *J* = 8.9, 5.0 Hz, 2H), 7.24 – 7.17 (m, 4H), 6.87 (d, *J* = 8.8 Hz, 2H), 3.24 (s, 3H). ppm.

^{13}C NMR (126 MHz, $CDCl_3$, 298 K, δ): 165.8 (d, *J* = 256.3 Hz), 144.0, 134.9, 132.1, 131.6 (d, *J* = 9.7 Hz), 125.0, 117.1 (d, *J* = 23.0 Hz), 114.8, 46.4. ppm.

^{19}F NMR (471 MHz, $CDCl_3$, 298 K, δ): –104.0 (m) ppm.

HRMS-EI (m/z) calculated for $C_{13}H_{11}NOSFBr^+$ $[M]^+$, 326.9723; found, 326.9729; deviation: –1.7 ppm.

((2-bromophenyl)imino)(4-fluorophenyl)(methyl)-λ^6-sulfanone (**20-C2**)

R*f* = 0.70 (EA/pentane = 1/2, v/v).

NMR Spectroscopy:

^{1}H NMR (500 MHz, $CDCl_3$, 298 K, δ): 8.09 (dd, *J* = 8.9, 5.0 Hz, 2H), 7.51 (dd, *J* = 7.9, 1.5 Hz, 1H), 7.24 – 7.18 (m, 3H), 7.09 – 7.01 (m, 1H), 6.81 – 6.75 (m, 1H), 3.25 (s, 3H). ppm.

^{13}C NMR (151 MHz, $CDCl_3$, 298 K, δ): 165.9 (d, *J* = 256.1 Hz), 143.3, 135.0 (d, *J* = 3.1 Hz), 133.2, 131.6 (d, *J* = 9.5 Hz), 128.1, 123.8, 123.5, 119.5, 117.0 (d, *J* = 22.6 Hz). ppm.

^{19}F NMR (471 MHz, $CDCl_3$, 298 K, δ): –104.1 (m) ppm.

HRMS-EI (m/z) calculated for $C_{13}H_{11}NOSFBr^+$ $[M]^+$, 326.9723; found, 326.9730; deviation: –2.2 ppm.

((3-bromophenyl)imino)(4-fluorophenyl)(methyl)-λ^6-sulfanone (**20-C3**)

R*f* = 0.25 (EA/pentane = 1/2, v/v).

NMR Spectroscopy:

^{1}H NMR (500 MHz, $CDCl_3$, 298 K, δ): 7.97 (dd, *J* = 8.9, 5.0 Hz, 2H), 7.25 – 7.19 (m, 2H), 7.18 (t, *J* = 2.0 Hz, 1H), 7.01 (dt, *J* = 7.9, 1.5 Hz, 1H), 6.97 (t, *J* = 7.8 Hz, 1H), 6.91 (ddd, *J* = 7.8, 2.1, 1.3 Hz, 1H), 3.25 (s, 3H). ppm.

^{13}C NMR (151 MHz, $CDCl_3$, 298 K, δ): 165.9 (d, *J* = 256.3 Hz), 146.4, 134.9 (d, *J* = 3.1 Hz), 131.5 (d, *J* = 9.5 Hz), 130.3, 126.4, 125.0, 122.7, 121.8, 117.1 (d, *J* = 22.8 Hz), 46.4. ppm.

^{19}F NMR (471 MHz, $CDCl_3$, 298 K, δ): –103.9 (m) ppm.

HRMS-EI (m/z) calculated for $C_{13}H_{11}NOSFBr^+$ $[M]^+$, 326.9723; found, 326.9729; deviation: –1.7 ppm.

(4-Fluorophenyl)(methyl)((trifluoromethyl)phenyl)imino)-λ^6-sulfanone (21)

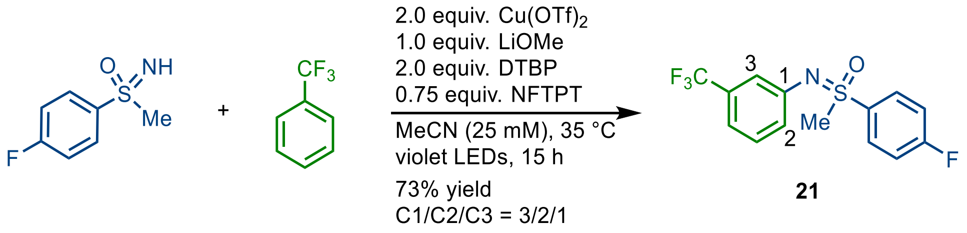

Under an ambient atmosphere, a 10 mL borosilicate vial equipped with a magnetic stir bar was charged with (4-fluorophenyl)(imino)(methyl)-λ^6-sulfanone (34.6 mg, 0.200 mmol, 1.00 equiv.). The vial was transferred into an anhydrous, N_2-filled glovebox. Under nitrogen atmosphere, $Cu(OTf)_2$ (144 mg, 0.400 mmol, 2.00 equiv.), LiOMe (7.5 mg, 0.20 mmol, 1.0 equiv.), and anhydrous MeCN (8.0 mL, *c* = 25 mM) were added into the vial, resulting in a blue suspension. The reaction mixture was stirred without irradiation for 30 min at 25 °C.

Subsequently, 1-fluoro-2,4,6-trimethylpyridinium tetrafluoroborate (NFTPT) (34.0 mg, 0.15 mmol, 0.75 equiv.), trifluromethylbenzene (1.46 g, 1.23 mL, 10.0 mmol, 50.0 equiv.), and 2,6-di-*tert*-butylpyridine (90.0 µL, 76.5 mg, 0.400 mmol, 2.00 equiv.) were added into the vial, resulting in a green suspension. The vial was sealed with a Teflon cap and taken out of the glovebox. The sealed vial was placed 5 cm away from two violet LEDs (Kessil PR160L-390 nm LEDs). The reaction mixture was stirred at a speed of 1000 rpm and irradiated for 15 h while maintaining the temperature at approximately 35 °C through cooling with a fan. After irradiation, volatiles were removed from the reaction mixture under reduced pressure. DCM (15 mL) was added to the residue, and the precipitate was removed by filtration through a glass frit. The filtrate was concentrated under reduced pressure, and the residue was purified by flash column chromatography on silica gel silica gel (EA/pentane = 1/2, v/v) to yield the mixture of (4-fluorophenyl)(methyl)((3-(trifluoromethyl)phenyl)imino)-λ^6-sulfanone (**21-C1**), (4-fluorophenyl)(methyl)((4-(trifluoromethyl)phenyl)imino)-λ^6-sulfanone (**21-C2**) and (4-fluorophenyl)(methyl)((2-(trifluoromethyl)phenyl)imino)-λ^6-sulfanone (**21-C3**) (46.4 mg, 146 mmol, 73%) as a colorless oil. The residue of mixture containing **21-C1** , **21-C2** , and **21-C3** was purified by another flash column chromatography on silica gel (EA/pentane = 1/3, v/v) to yield **21-C1** (21.0 mg), **21-C2** (12.6 mg) and **21-C3** (7.0 mg) seperately as colorless solid for characterizations. The ratio of **21-C1**, **21-C2,** and **21-C3** was determined by ^{19}F NMR.

(4-Fluorophenyl)(methyl)((3-(trifluoromethyl)phenyl)imino)-λ^6-sulfanone (**21-C1**):

R*f* = 0.25 (EA/pentane = 1/3, v/v).

NMR Spectroscopy:

^{1}H NMR (500 MHz, $CDCl_3$, 298 K, δ): 7.98 (dd, *J* = 8.9, 5.0 Hz, 2H), 7.26 – 7.18 (m, 4H), 7.13 (td, *J* = 7.2, 1.9 Hz, 2H), 3.26 (s, 3H). ppm.

^{13}C NMR (126 MHz, $CDCl_3$, 298 K, δ): 165.7 (d, *J* = 256.3 Hz), 145.4, 134.7 (d, *J* = 3.1 Hz), 131.55 (q, *J* = 32.0 Hz), 131.50 (d, *J* = 9.5 Hz), 129.4, 126.0, 124.0 (d, *J* = 272.4 Hz), 120.1 (q, *J* = 3.7 Hz), 118.4 (q, *J* = 3.8 Hz), 117.0 (d, *J* = 23.1 Hz), 46.3. ppm.

^{19}F NMR (471 MHz, $CDCl_3$, 298 K, δ): –62.8 (s), –103.8 (m) ppm.

HRMS-EI (m/z) calculated for $C_{14}H_{11}NOSF_4^+$ $[M]^+$, 317.0492; found, 317.0494; deviation: –0.8 ppm.

(4-Fluorophenyl)(methyl)((4-(trifluoromethyl)phenyl)imino)-λ^6-sulfanone (**21-C2**):

R*f* = 0.20 (EA/pentane = 1/3, v/v).).

NMR Spectroscopy:

^{1}H NMR (500 MHz, $CDCl_3$, 298 K, δ): 7.97 (dd, *J* = 8.9, 5.0 Hz, 2H), 7.39 – 7.34 (m, 2H), 7.22 (dd, *J* = 8.9, 8.2 Hz, 2H), 7.08 – 7.02 (m, 2H), 3.28 (s, 3H). ppm.

^{13}C NMR (151 MHz, $CDCl_3$, 298 K, δ): 165.9 (d, *J* = 256.6 Hz), 148.4 (q, *J* = 1.4 Hz), 134.7 (d, *J* = 3.1 Hz), 131.5 (d, *J* = 9.5 Hz), 126.4 (q, *J* = 3.8 Hz), 124.7 (q, *J* = 271.3 Hz), 123.7 (q, *J* = 32.4 Hz), 123.0, 117.3 (d, *J* = 22.7 Hz), 46.6.ppm.

^{19}F NMR (471 MHz, $CDCl_3$, 298 K, δ): –61.8 (s), –103.6 (m) ppm.

HRMS-EI (m/z) calculated for $C_{14}H_{11}NOSF_4^+$ $[M]^+$, 317.0492; found, 317.0498; deviation: –2.1 ppm.

(4-Fluorophenyl)(methyl)((2-(trifluoromethyl)phenyl)imino)-λ^6-sulfanone (**21-C3**):

R*f* = 0.70 (EA/pentane = 1/3, v/v).).

^{1}H NMR (500 MHz, $CDCl_3$, 298 K, δ): 8.05 (dd, *J* = 8.9, 5.0 Hz, 2H), 7.58 – 7.54 (m, 1H), 7.30 – 7.18 (m, 4H), 6.96 (dddd, *J* = 7.7, 6.9, 1.7, 0.9 Hz, 1H), 3.22 (s, 3H). ppm.

^{13}C NMR (151 MHz, $CDCl_3$, 298 K, δ): 165.9 (d, *J* = 256.2 Hz), 143.8 (q, *J* = 1.5 Hz), 135.2 (d, *J* = 3.1 Hz), 132.6, 131.5 (d, *J* = 9.6 Hz), 127.0 (q, *J* = 5.4 Hz), 124.6 (q, 272.8 Hz), 123.7 (q, *J* = 28.6 Hz), 123.4, 121.3, 117.0 (d, *J* = 22.6 Hz), 45.5. ppm.

^{19}F NMR (565 MHz, $CDCl_3$, 298 K, δ): –62.0 (s), –104.0 (m) ppm.

HRMS-EI (m/z) calculated for $C_{14}H_{11}NOSF_4^+$ $[M]^+$, 317.0492; found, 317.0498; deviation: –2.0 ppm.

((2,5-Difluorophenyl)imino)(4-fluorophenyl)(methyl)-λ^6-sulfanone (22)

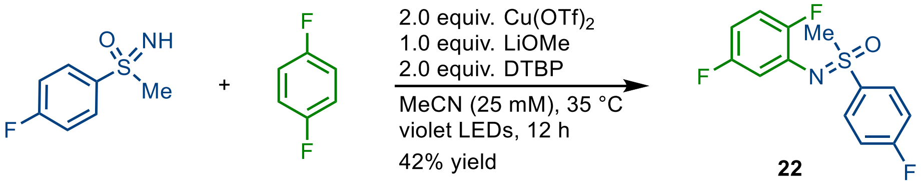

In a nitrogen-filled glovebox, a 10 mL borosilicate vial equipped with a magnetic stir bar was charged with and (4-fluorophenyl)(imino)(methyl)-λ^6-sulfanone (34.6 mg, 0.200 mmol, 1.00 equiv.), LiOMe (7.6 mg, 0.20 mmol, 1.0 equiv.), $Cu(OTf)_2$ (145 mg, 0.400 mmol, 2.00 equiv.), 2,6-di-tert-butylpyridine (76.5 mg, 0.400 mmol, 2.00 equiv.) and 1,4-difluorobenzene (2.28 g, 20.0 mmol, 100 equiv.), anhydrous MeCN (8.0 mL, c = 25 mM) was then added into the vial. The vial was sealed with a Teflon cap and placed 5 cm away from two violet LEDs (Kessil PR160L-390 nm LEDs). The reaction mixture was irradiated for 15 h while maintaining the temperature at approximately 35 °C through cooling with a fan. After irradiation, the reaction mixture was evaporated under reduced pressure to remove all volatiles. The residue was purified by chromatography on silica gel (DCM) to yield ((2,5-difluorophenyl)imino)(4-fluorophenyl)(methyl)-λ^6-sulfanone (**22**) (23.8 mg, 83 μmol, 42%) as a slightly yellow oil.

R*f* = 0.40 (DCM).

NMR Spectroscopy:

^{1}H NMR (500 MHz, $CDCl_3$, 298 K, δ): 8.04–7.97 (m, 2H), 7.25–7.19 (m, 2H), 6.91 (ddd, *J* = 10.1, 9.0, 5.2 Hz, 1H), 6.84 (ddd, *J* = 9.8, 6.7, 3.1 Hz, 1H), 6.53 (ddt, *J* = 8.9, 7.5, 3.3 Hz, 1H), 3.29 (s, 3H) ppm.

^{13}C NMR (126 MHz, $CDCl_3$, 298 K, δ): 166.0 (d, *J* = 256.4 Hz), 158.5 (dd, *J* = 241.7, 2.5 Hz), 152.9 (dd, *J* = 239.7, 2.9 Hz), 134.8 (d, *J* = 3.1 Hz), 133.6 (dd, *J* = 13.9, 1.0 Hz), 131.4 (d, *J* = 9.6 Hz), 117.2 (d, *J* = 22.6 Hz), 116.1 (dd, *J* = 23.4, 10.1 Hz), 111.8 (dd, *J* = 25.1, 2.2 Hz), 109.0 (dd, *J* = 23.9, 7.7 Hz), 46.5 ppm.

^{19}**F NMR** (471 MHz, CDCl$_3$, 298 K, δ): –103.7 (m), –118.4 (m), –131.7 (m) ppm.

HRMS-EI (m/z) calculated for $C_{13}H_{10}NOSF_3^+$ [M]$^+$, 285.0430; found, 285.0431; deviation: –0.5 ppm.

Copper-LMCT enabled C–H sulfoximination: *NH*-sulfoximine scope

Methyl(phenylimino)(thiophen-2-yl)-λ^6-sulfanone (23)

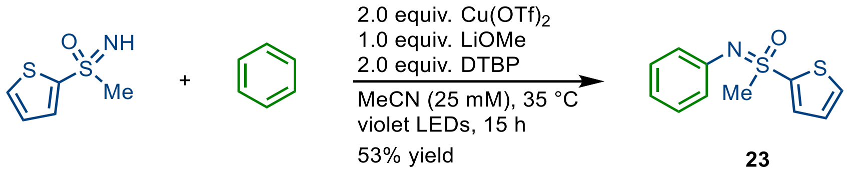

In a nitrogen-filled glovebox, a 10 mL borosilicate vial equipped with a magnetic stir bar was charged with and imino(methyl)(thiophen-2-yl)-λ^6-sulfanone (32.2 mg, 0.200 mmol, 1.00 equiv.), Cu(OTf)$_2$ (145 mg, 0.400 mmol, 2.00 equiv.), 2,6-di-tert-butylpyridine (76.5 mg, 0.400 mmol, 2.00 equiv.) and benzene (1.56 g, 20.0 mmol, 100 equiv.), anhydrous MeCN (8.0 mL, c = 25 mM) was then added into the vial. The vial was sealed with a Teflon cap and placed 5 cm away from two violet LEDs (Kessil PR160L-390 nm LEDs). The reaction mixture was irradiated for 15 h while maintaining the temperature at approximately 35 °C through cooling with a fan. After irradiation, the reaction mixture was evaporated under reduced pressure to remove all volatiles. The residue was purified by chromatography on silica gel (EA/DCM = 1/50, v/v) to yield methyl(phenylimino)(thiophen-2-yl)-λ^6-sulfanone (**23**) (25.2 mg, 106 μmol, 53%) as a slightly yellow oil.

R*f* = 0.31 (DCM).

NMR Spectroscopy:

^{1}**H NMR** (500 MHz, CDCl$_3$, 298 K, δ): 7.66–7.58 (m, 2H), 7.17 (t, *J* = 7.8 Hz, 2H), 7.10–6.99 (m, 3H), 6.89 (t, *J* = 7.0 Hz, 1H), 3.37 (s, 3H) ppm.

^{13}**C NMR** (126 MHz, CDCl$_3$, 298 K, δ): 144.4, 134.4, 134.2, 129.0, 128.2, 123.7, 122.4, 48.0 ppm.

HRMS-ESI (m/z) calculated for $C_{11}H_{11}NOS_2^+$ [M]$^+$, 237.0277; found, 237.0279; deviation: –0.8 ppm.

((2,5-Dichlorophenyl)imino)diphenyl-λ^6-sulfanone (24)

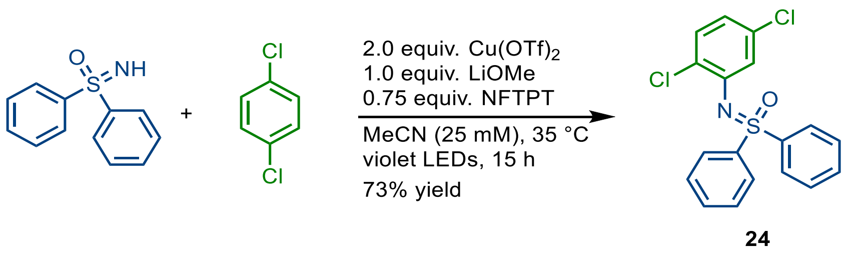

Under an ambient atmosphere, a 10 mL borosilicate vial equipped with a magnetic stir bar was charged with iminodiphenyl-λ^6-sulfanone (43.4 mg, 0.200 mmol, 1.00 equiv.). The vial was transferred into an anhydrous, N$_2$-filled glovebox. Under nitrogen atmosphere, Cu(OTf)$_2$ (144 mg, 0.400 mmol, 2.00 equiv.), LiOMe (7.6 mg, 0.20 mmol, 1.0 equiv.), and anhydrous MeCN (8.0 mL, *c* = 25 mM) were added into the vial, resulting in a blue

suspension. The reaction mixture was stirred without irradiation for 30 min at 25 °C. Subsequently, 1-fluoro-2,4,6-trimethylpyridinium tetrafluoroborate (NFTPT) (34.0 mg, 0.15 mmol, 0.75 equiv.), and 1,4-dichlorobenzene (1.47 g, 10.0 mmol, 50.0 equiv.) were added into the vial, resulting in a green suspension. The vial was sealed with a Teflon cap and taken out of the glovebox. The sealed vial was placed 5 cm away from two violet LEDs (Kessil PR160L-390 nm LEDs). The reaction mixture was stirred at a speed of 1000 rpm and irradiated for 15 h while maintaining the temperature at approximately 35 °C through cooling with a fan. After irradiation, volatiles were removed from the reaction mixture under reduced pressure. DCM (15 mL) was added to the residue, and the precipitate was removed by filtration through a glass frit. The filtrate was concentrated under reduced pressure, and the residue was purified by flash column chromatography on silica gel (EA/pentane = 1/5, v/v) to yield ((2,5-dichlorophenyl)imino)diphenyl-λ^6-sulfanone (**24**) (52.9 mg, 146 μmol, 73%) as a colorless solid.

R*f* = 0.40 (EA/pentane = 1/5, v/v).

NMR Spectroscopy:

^{1}H NMR (500 MHz, $CDCl_3$, 298 K, δ): 8.11 (dd, *J* = 8.3, 1.4 Hz, 4H), 7.56 – 7.43 (m, 6H), 7.27 (d, *J* = 2.4 Hz, 1H), 7.25 (d, *J* = 8.5 Hz, 1H), 6.79 (dd, *J* = 8.5, 2.4 Hz, 1H). ppm.

^{13}C NMR (126 MHz, $CDCl_3$, 298 K, δ): 143.1, 140.3, 133.2, 132.5, 130.4, 129.6, 128.6, 127.6, 123.6, 122.7. ppm.

HRMS-EI (m/z) calculated for $C_{18}H_{13}NOSCl_2^+$ [M+H]$^+$, 361.0089; found, 361.0095; deviation: –1.8 ppm.

1 mmol scale synthesis of ((2,5-dichlorophenyl)imino)diphenyl-λ^6-sulfanone (24)

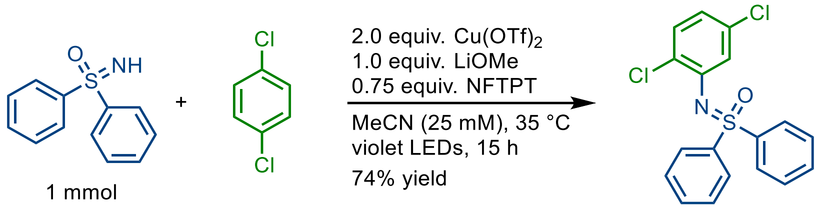

24

Under an ambient atmosphere, a 100 mL Schlenk tube equipped with a magnetic stir bar was charged with iminodiphenyl-λ^6-sulfanone (217 mg, 1.00 mol, 1.00 equiv.). The Schlenk tube was transferred into an anhydrous, N_2-filled glovebox. Under nitrogen atmosphere, $Cu(OTf)_2$ (723 mg, 2.00 mmol, 2.00 equiv.), LiOMe (37.9 mg, 1.00 mmol, 1.00 equiv.), and anhydrous MeCN (32 mL, *c* = 25 mM) were added into the schlenk tube, resulting in a blue suspension. The reaction mixture was stirred without irradiation for 30 min at 25 °C. Subsequently, 1-fluoro-2,4,6-trimethylpyridinium tetrafluoroborate (NFTPT) (0.17 g, 0.75 mmol, 0.75 equiv.), 1,4-dichlorobenze (7.35 g, 50.0 mmol, 50.0 equiv) were added into the vial, resulting in a green suspension. The vial was sealed with a rubber septum and taken out of the glovebox. The sealed schlenk tube was placed 5 cm away from two violet LEDs (Kessil PR160L-390 nm LEDs). The reaction mixture was stirred at a speed of 1000 rpm and irradiated for 15 h while maintaining the temperature at approximately 35 °C through cooling with a fan. After irradiation, volatiles were removed from the reaction mixture under reduced pressure. DCM

(30 mL) was added to the residue, and the precipitate was removed by filtration through a glass frit. The filtrate was concentrated under reduced pressure, and the residue was purified by flash column chromatography on silica gel (EA/pentane = 1/5, v/v) to yield ((2,5-dichlorophenyl)imino)diphenyl-λ6-sulfanone (**24**) (267 mg, 737 μmol, 74%) as a colorless solid.

Recovery of the unreacted arene 1,4-dichlorobenzene

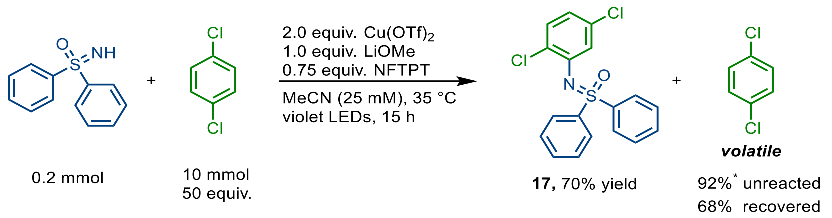

Under an ambient atmosphere, a 10 mL borosilicate vial equipped with a magnetic stir bar was charged with iminodiphenyl-λ6-sulfanone (43.4 mg, 0.200 mmol, 1.00 equiv.). The vial was transferred into an anhydrous, N_2-filled glovebox. Under nitrogen atmosphere, $Cu(OTf)_2$ (144 mg,0.400 mmol, 2.00 equiv.), LiOMe (7.6 mg, 0.20 mmol, 1.0 equiv.), and anhydrous MeCN (8.0 mL, *c* = 25 mM) were added into the vial, resulting in a blue suspension. The reaction mixture was stirred without irradiation for 30 min at 25 °C. Subsequently, 1-fluoro-2,4,6-trimethylpyridinium tetrafluoroborate (NFTPT) (34.0 mg, 0.15 mmol, 0.75 equiv.), and 1,4-dichlorobenzene (1.47 g, 10.0 mmol, 50.0 equiv.) were added into the vial, resulting in a green suspension. The vial was sealed with a Teflon cap and taken out of the glovebox. The sealed vial was placed 5 cm away from two violet LEDs (Kessil PR160L-390 nm LEDs). The reaction mixture was stirred at a speed of 1000 rpm and irradiated for 15 h while maintaining the temperature at approximately 35 °C through cooling with a fan. After irradiation, MeCN was removed from the reaction mixture under reduced pressure. DCM (15 mL) was added to the residue, and the resulting suspension was filtered through a silica pad, eluting with EtOAc (15 mL). The filtrate was concentrated to roughtly 0.5 mL under reduced pressure, and then 1 mL $CDCl_3$ was added to the reaction mixture. Dibromomethane (869 mg, 5.00 mmol) was added as the internal standard. Due to the volatility of the arene, a ^{1}H NMR was measured to determine the amount of unreacted 1,4-dichlorobenzene (9.2 mmol, 92%*) before chromatography. The NMR sample was recycled and concentrated under reduced pressure, and the residue was purified by flash column chromatography on silica gel (eluting with pentane first to obtain the arene, then EA/pentane = 1/4, v/v to obtain the N-arylated sulfoximine) to yield 1,4-dichlorobenze (997 mg, 6.78 mmol, 68% recoved) as a beige solid, and to yield ((2,5-dichlorophenyl)imino)diphenyl-λ6-sulfanone (**24**) (50.7 mg, 140 μmol, 70%) as a colorless solid.

^{1}H NMR of the reaction mixture

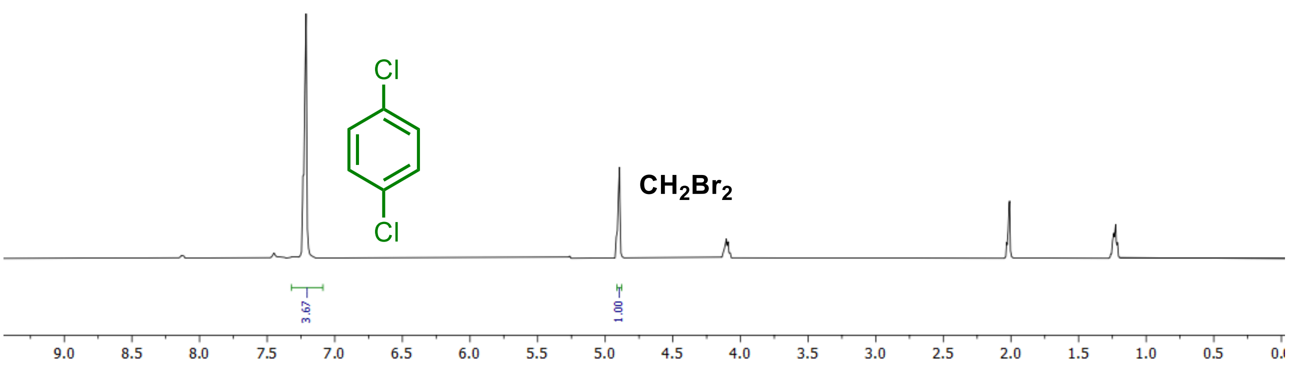

^{1}H NMR of the recovered 1,4-dichlorobenzene

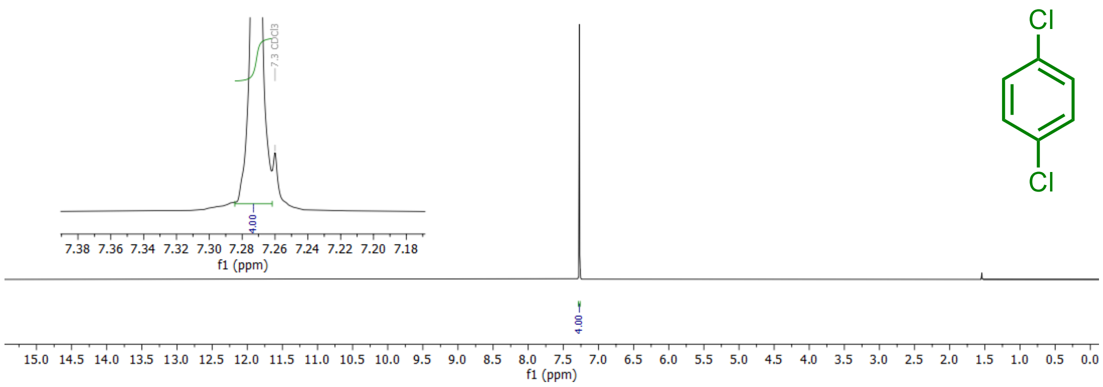

^{13}C NMR of the recovered 1,4-dichlorobenze

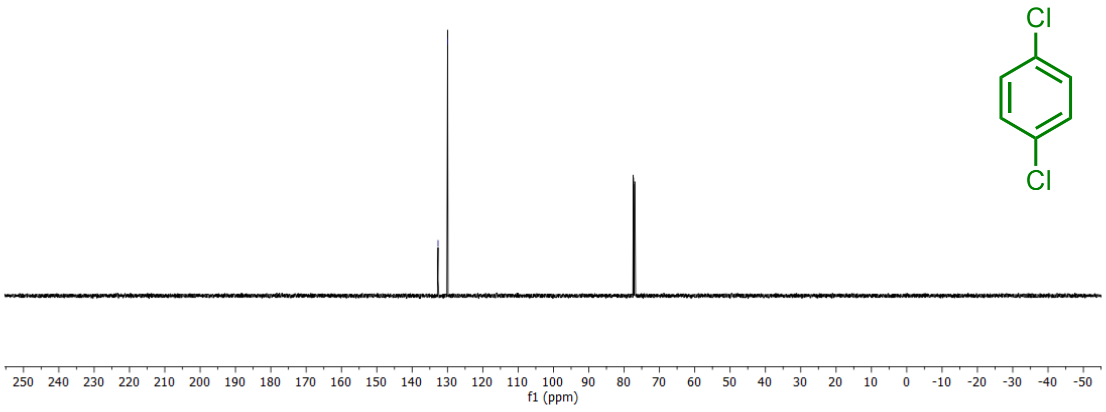

1,4-dichlorobenze

R*f* = 0.95 (EA/pentane = 1/4, v/v).

NMR Spectroscopy:

^{1}H NMR (500 MHz, $CDCl_3$, 298 K, δ): 7.27 (s, 4H). ppm.

^{13}C NMR (126 MHz, $CDCl_3$, 298 K, δ): 132.7, 130.0. ppm.

((2,5-Dichlorophenyl)imino)(4-iodophenyl)(methyl)-λ^6-sulfanone (25)

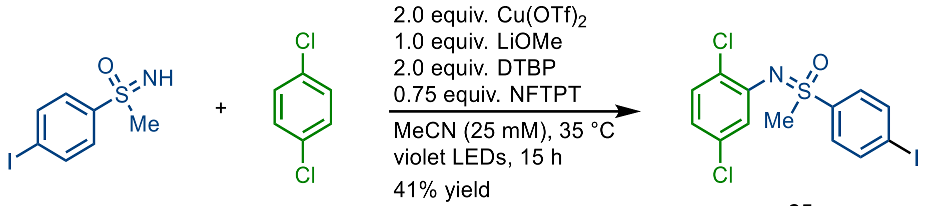

25

Under an ambient atmosphere, a 10 mL borosilicate vial equipped with a magnetic stir bar was charged with (4-iodophenyl)(imino)(methyl)-λ^6-sulfanone (56.2 mg, 0.200 mmol, 1.00 equiv.). The vial was transferred into an anhydrous, N_2-filled glovebox. Under nitrogen atmosphere, $Cu(OTf)_2$ (144 mg, 0.400 mmol, 2.00 equiv.), LiOMe (7.5 mg, 0.20 mmol, 1.0 equiv.), and anhydrous MeCN (8.0 mL, *c* = 25 mM) were added into the vial, resulting in a blue suspension. The reaction mixture was stirred without irradiation for 30 min at 25 °C. Subsequently, 1-fluoro-2,4,6-trimethylpyridinium tetrafluoroborate (NFTPT) (34.0 mg, 0.15 mmol, 0.75 equiv.), 1,4-dichlorobenzene (1.47 g, 10.0 mmol, 50.0 equiv.), and 2,6-di-*tert*-butylpyridine (90.0 µL, 76.5 mg, 0.400 mmol, 2.00 equiv.) were added into the vial, resulting in a green suspension. The vial was sealed with a Teflon cap and taken out of the glovebox. The sealed vial was placed 5 cm away from two violet LEDs (Kessil PR160L-390 nm LEDs). The reaction mixture was stirred at a speed of 1000 rpm and irradiated for 15 h while maintaining the temperature at approximately 35 °C through cooling with a fan. After irradiation, volatiles were removed from the reaction mixture under reduced pressure. DCM (15 mL) was added to the residue, and the precipitate was removed by filtration through a glass frit. The filtrate was concentrated under reduced pressure, and the residue was purified by flash column chromatography on silica gel (EA/pentane = 1/8, v/v) to yield ((2,5-dichlorophenyl)imino)(4-iodophenyl)(methyl)-λ6-sulfanone (**25**) (34.7 mg, 81 µmol, 41%) as a colorless solid.

R*f* = 0.20 (EA/pentane = 1/8, v/v).

NMR Spectroscopy:

^{1}**H NMR** (500 MHz, $CDCl_3$, 298 K, δ): 7.91 (d, *J* = 8.6 Hz, 2H), 7.72 (d, *J* = 8.6 Hz, 2H), 7.22 (d, *J* = 8.5 Hz, 1H), 7.19 (d, *J* = 2.4 Hz, 1H), 6.82 (dd, *J* = 8.5, 2.5 Hz, 1H), 3.27 (s, 3H). ppm.

^{13}**C NMR** (126 MHz, $CDCl_3$, 298 K, δ): 142.9, 139.0, 138.5, 132.5, 130.5, 127.0, 123.5, 123.0, 101.8, 45.9. ppm.

HRMS-ESI (m/z) calculated for $C_{13}H_{10}NOSICl_2Na^+$ [M+Na]$^+$, 447.8797; found, 447.8800; deviation: –0.7 ppm.

((2,5-Dichlorophenyl)imino)(methyl)(4-(trifluoromethoxy)phenyl)-λ^6-sulfanone (26)

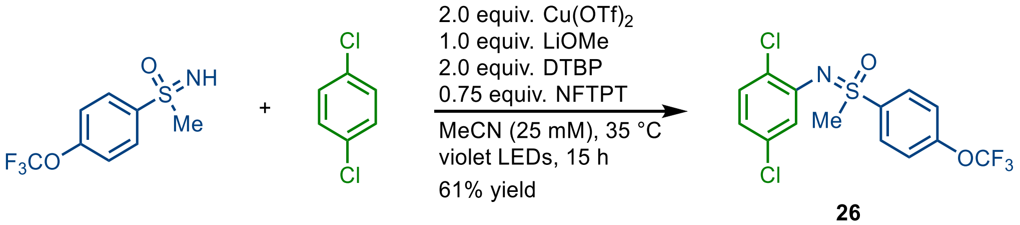

26

Under an ambient atmosphere, a 10 mL borosilicate vial equipped with a magnetic stir bar was charged with (4-trifluoromethoxyphenyl)(imino)(methyl)-λ^6-sulfanone (47.8 mg, 0.200 mmol, 1.00 equiv.). The vial was transferred into an anhydrous, N_2-filled glovebox. Under nitrogen atmosphere, $Cu(OTf)_2$ (144 mg, 0.400 mmol, 2.00 equiv.), LiOMe (7.5 mg, 0.20 mmol, 1.0 equiv.), and anhydrous MeCN (8.0 mL, *c* = 25 mM) were added into the vial, resulting in a blue suspension. The reaction mixture was stirred without irradiation for 30 min at 25 °C. Subsequently, 1-fluoro-2,4,6-trimethylpyridinium tetrafluoroborate (NFTPT) (34.0 mg, 0.15 mmol, 0.75 equiv.), 1,4-dichlorobenzene (1.47 g, 10.0 mmol, 50.0 equiv.), and 2,6-di-*tert*-butylpyridine (90.0 μL, 76.5 mg, 0.400 mmol, 2.00 equiv.) were added into the vial, resulting in a green suspension. The vial was sealed with a Teflon cap and taken out of the glovebox. The sealed vial was placed 5 cm away from two violet LEDs (Kessil PR160L-390 nm LEDs). The reaction mixture was stirred at a speed of 1000 rpm and irradiated for 15 h while maintaining the temperature at approximately 35 °C through cooling with a fan. After irradiation, volatiles were removed from the reaction mixture under reduced pressure. DCM (15 mL) was added to the residue, and the precipitate was removed by filtration through a glass frit. The filtrate was concentrated under reduced pressure, and the residue was purified by flash column chromatography on silica gel (EA/pentane = 1/5, v/v) to yield ((2,5-dichlorophenyl)imino)(4-trifluoromethoxyphenyl)(methyl)-λ6-sulfanone (**26**) (46.3 mg, 121 μmol, 61%) as a colorless solid.

R*f* = 0.30 (EA/pentane = 1/5, v/v).

NMR Spectroscopy:

^{1}H NMR (500 MHz, $CDCl_3$, 298 K, δ): 8.09 (d, *J* = 8.9 Hz, 2H), 7.38 (dd, *J* = 9.0, 1.1 Hz, 2H), 7.23 (d, *J* = 8.5 Hz, 1H), 7.22 (d, *J* = 2.4 Hz, 1H), 6.84 (dd, *J* = 8.5, 2.4 Hz, 1H), 3.28 (s, 3H). ppm.

^{13}C NMR (126 MHz, $CDCl_3$, 298 K, δ): 153.2 (q, *J* = 1.8 Hz), 142.9, 137.0, 132.7, 131.0, 130.6, 127.1, 123.7, 123.2, 121.5, 120.3 (q, *J* = 260.0 Hz), 46.0. ppm.

^{19}F NMR (471 MHz, $CDCl_3$, 298 K, δ): –57.6 (s) ppm.

HRMS-EI (m/z) calculated for $C_{14}H_{10}NO_2SF_3Cl_2^+$ [M]$^+$, 382.9756; found, 382.9760; deviation: –1.2 ppm.

10-((2,5-Dichlorophenyl)imino)-10*H*-10-λ^4--phenoxathiine 10-oxide (27)

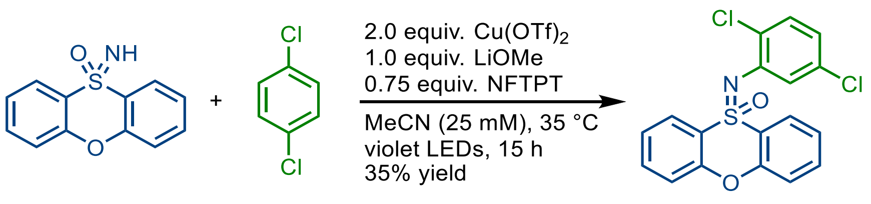

27

Under an ambient atmosphere, a 10 mL borosilicate vial equipped with a magnetic stir bar was charged with 10-imino-10*H*-10 λ^4-phenoxathiine 10-oxide (46.2 mg, 0.200 mmol, 1.00 equiv.). The vial was transferred into an anhydrous, N_2-filled glovebox. Under nitrogen atmosphere, $Cu(OTf)_2$ (144 mg, 0.400 mmol, 2.00 equiv.), LiOMe (7.5 mg, 0.20 mmol, 1.0 equiv.), and anhydrous MeCN (8.0 mL, *c* = 25 mM) were added into the vial,

resulting in a blue suspension. The reaction mixture was stirred without irradiation for 30 min at 25 °C. Subsequently, 1-fluoro-2,4,6-trimethylpyridinium tetrafluoroborate (NFTPT) (34.0 mg, 0.15 mmol, 0.75 equiv.), 1,4-dichlorobenzene (1.47 g, 10.0 mmol, 50.0 equiv.) were added into the vial, resulting in a green suspension. The vial was sealed with a Teflon cap and taken out of the glovebox. The sealed vial was placed 5 cm away from two violet LEDs (Kessil PR160L-390 nm LEDs). The reaction mixture was stirred at a speed of 1000 rpm and irradiated for 15 h while maintaining the temperature at approximately 35 °C through cooling with a fan. After irradiation, volatiles were removed from the reaction mixture under reduced pressure. DCM (15 mL) was added to the residue, and the precipitate was removed by filtration through a glass frit. The filtrate was concentrated under reduced pressure, and the residue was purified by flash column chromatography on silica gel (EA/pentane = 1/8, v/v) to yield 10-((2,5-dichlorophenyl)imino)-10H-10-λ^4--phenoxathiine 10-oxide (**27**) (26.3 mg, 70 μmol, 35%) as a colorless solid.

R*f* = 0.30 (EA/pentane = 1/8, v/v).

NMR Spectroscopy:

^{1}H NMR (500 MHz, $CDCl_3$, 298 K, δ): 7.96 (dd, *J* = 8.0, 1.7 Hz, 2H), 7.63 (ddd, *J* = 8.7, 7.3, 1.6 Hz, 2H), 7.41 (dd, *J* = 8.4, 1.1 Hz, 2H), 7.35 (ddd, *J* = 8.2, 7.3, 1.1 Hz, 2H), 7.25 (d, *J* = 2.4 Hz, 1H), 7.15 (d, *J* = 8.5 Hz, 1H), 6.89 (dd, *J* = 8.5, 2.4 Hz, 1H). ppm.

^{13}C NMR (126 MHz, $CDCl_3$, 298 K, δ): 151.6, 141.6, 134.3, 132.3, 130.5, 128.7, 125.5, 124.9, 124.8, 124.3, 124.0, 119.1. ppm.

HRMS-ESI (m/z) calculated for $C_{18}H_{11}NO_2SCl_2^+$ [M+H]$^+$, 375.9960; found, 375.9960; deviation: –0.07 ppm.

((2,5-Dichlorophenyl)imino)(ethyl)(phenyl)-λ^6-sulfanone (28)

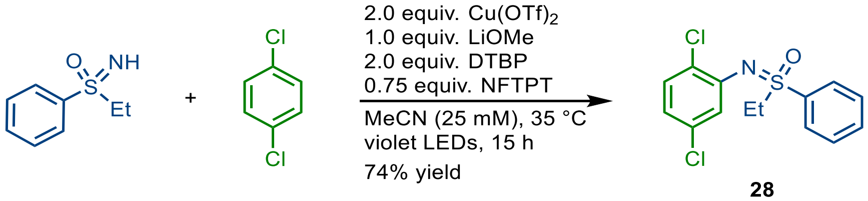

28

Under an ambient atmosphere, a 10 mL borosilicate vial equipped with a magnetic stir bar was charged with (phenyl)(imino)(ethyl)-λ^6-sulfanone (33.8 mg, 0.200 mmol, 1.00 equiv.). The vial was transferred into an anhydrous, N_2-filled glovebox. Under nitrogen atmosphere, $Cu(OTf)_2$ (144 mg, 0.400 mmol, 2.00 equiv.), LiOMe (7.5 mg, 0.20 mmol, 1.0 equiv.), and anhydrous MeCN (8.0 mL, *c* = 25 mM) were added into the vial, resulting in a blue suspension. The reaction mixture was stirred without irradiation for 30 min at 25 °C. Subsequently, 1-fluoro-2,4,6-trimethylpyridinium tetrafluoroborate (NFTPT) (34.0 mg, 0.15 mmol, 0.75 equiv.), 1,4-dichlorobenzene (1.47 g, 10.0 mmol, 50.0 equiv.), and 2,6-di-*tert*-butylpyridine (90.0 μL, 76.5 mg, 0.400 mmol, 2.00 equiv.) were added into the vial, resulting in a green suspension. The vial was sealed with a Teflon cap and taken out of the glovebox. The sealed vial was placed 5 cm away from two violet LEDs (Kessil PR160L-390 nm LEDs). The reaction mixture was stirred at a speed of 1000 rpm and irradiated for 15 h while maintaining the temperature at approximately 35 °C through cooling with a fan. After irradiation, volatiles were removed

from the reaction mixture under reduced pressure. DCM (15 mL) was added to the residue, and the precipitate was removed by filtration through a glass frit. The filtrate was concentrated under reduced pressure, and the residue was purified by flash column chromatography on silica gel (EA/pentane = 1/4, v/v) to yield ((2,5-dichlorophenyl)imino)(ethyl)(phenyl)-λ^6-sulfanone (**28**) (46.5 mg, 148 μmol, 74%) as a colorless solid.

R*f* = 0.50 (EA/pentane = 1/4, v/v).

NMR Spectroscopy:

^{1}**H NMR** (500 MHz, $CDCl_3$, 298 K, δ): 7.95 (dd, *J* = 8.4, 1.4 Hz, 2H), 7.66 – 7.57 (m, 1H), 7.54 (dd, *J* = 8.2, 7.0 Hz, 2H), 7.21 (d, *J* = 8.5 Hz, 1H), 7.19 (d, *J* = 2.4 Hz, 1H), 6.77 (dd, *J* = 8.5, 2.3 Hz, 1H), 3.37 (qd, *J* = 7.3, 2.5 Hz, 2H), 1.31 (t, *J* = 7.4 Hz, 3H). ppm.

^{13}**C NMR** (126 MHz, $CDCl_3$, 298 K, δ): 143.6, 136.6, 133.8, 132.5, 130.4, 129.7, 129.4, 127.0, 123.3, 122.4, 52.2, 7.9. ppm.

HRMS-ESI (m/z) calculated for $C_{14}H_{13}NOSCl_2^+$ $[M+H]^+$, 313.0089; found, 313.0094; deviation: –1.6 ppm.

((2,5-Dichlorophenyl)imino)(methyl)(m-tolyl)-λ^6-sulfanone (29)

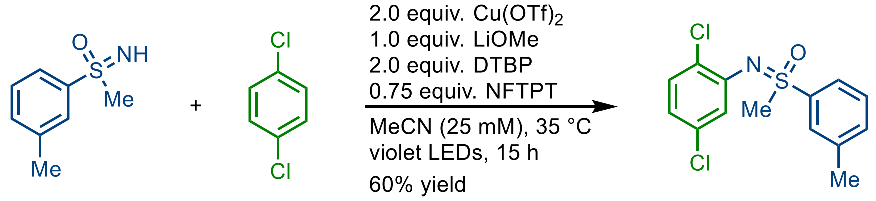

29

Under an ambient atmosphere, a 10 mL borosilicate vial equipped with a magnetic stir bar was charged with (m-tolyl)(imino)(methyl)-λ^6-sulfanone (33.8 mg, 0.200 mmol, 1.00 equiv.). The vial was transferred into an anhydrous, N_2-filled glovebox. Under nitrogen atmosphere, $Cu(OTf)_2$ (144 mg, 0.400 mmol, 2.00 equiv.), LiOMe (7.5 mg, 0.20 mmol, 1.0 equiv.), and anhydrous MeCN (8.0 mL, *c* = 25 mM) were added into the vial, resulting in a blue suspension. The reaction mixture was stirred without irradiation for 30 min at 25 °C. Subsequently, 1-fluoro-2,4,6-trimethylpyridinium tetrafluoroborate (NFTPT) (34.0 mg, 0.15 mmol, 0.75 equiv.), 1,4-dichlorobenzene (1.47 g, 10.0 mmol, 50.0 equiv.), and 2,6-di-*tert*-butylpyridine (90.0 μL, 76.5 mg, 0.400 mmol, 2.00 equiv.) were added into the vial, resulting in a green suspension. The vial was sealed with a Teflon cap and taken out of the glovebox. The sealed vial was placed 5 cm away from two violet LEDs (Kessil PR160L-390 nm LEDs). The reaction mixture was stirred at a speed of 1000 rpm and irradiated for 15 h while maintaining the temperature at approximately 35 °C through cooling with a fan. After irradiation, volatiles were removed from the reaction mixture under reduced pressure. DCM (15 mL) was added to the residue, and the precipitate was removed by filtration through a glass frit. The filtrate was concentrated under reduced pressure, and the residue was purified by flash column chromatography on silica gel (EA/pentane = 1/6, v/v) to yield ((2,5-dichlorophenyl)imino)(methyl)(m-tolyl)-λ6-sulfanone (**29**) (38.0 mg, 121 μmol, 60%) as a colorless solid.

R*f* = 0.40 (EA/pentane = 1/6, v/v).

NMR Spectroscopy:

[1]**H NMR** (500 MHz, $CDCl_3$, 298 K, δ): 7.84 (s, 1H), 7.82 – 7.80 (m, 1H), 7.47 – 7.36 (m, 2H), 7.25 – 7.16 (m, 2H), 6.79 (dd, J = 8.5, 2.5 Hz, 1H), 3.25 (s, 3H), 2.43 (s, 3H). ppm.

[13]**C NMR** (126 MHz, $CDCl_3$, 298 K, δ): 143.5, 140.1, 138.6, 134.7, 132.5, 130.5, 129.6, 128.9, 127.1, 125.6, 123.6, 122.7, 46.1, 21.5. ppm.

HRMS-EI (m/z) calculated for $C_{14}H_{13}NOSCl_2^+$ $[M]^+$, 313.0089; found, 313.0091; deviation: –0.4 ppm.

(4-Bromophenyl)((2,5-dichlorophenyl)imino)(methyl)-λ^6-sulfanone (30)

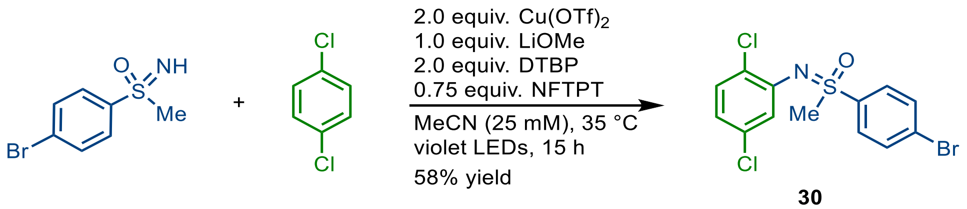

30

Under an ambient atmosphere, a 10 mL borosilicate vial equipped with a magnetic stir bar was charged with (4-bromophenyl)(imino)(methyl)-λ^6-sulfanone (46.8 mg, 0.200 mmol, 1.00 equiv.). The vial was transferred into an anhydrous, N_2-filled glovebox. Under nitrogen atmosphere, $Cu(OTf)_2$ (144 mg, 0.400 mmol, 2.00 equiv.), LiOMe (7.5 mg, 0.20 mmol, 1.0 equiv.), and anhydrous MeCN (8.0 mL, c = 25 mM) were added into the vial, resulting in a blue suspension. The reaction mixture was stirred without irradiation for 30 min at 25 °C. Subsequently, 1-fluoro-2,4,6-trimethylpyridinium tetrafluoroborate (NFTPT) (34.0 mg, 0.15 mmol, 0.75 equiv.), 1,4-dichlorobenzene (1.47 g, 10.0 mmol, 50.0 equiv.), and 2,6-di-*tert*-butylpyridine (90.0 µL, 76.5 mg, 0.400 mmol, 2.00 equiv.) were added into the vial, resulting in a green suspension. The vial was sealed with a Teflon cap and taken out of the glovebox. The sealed vial was placed 5 cm away from two violet LEDs (Kessil PR160L-390 nm LEDs). The reaction mixture was stirred at a speed of 1000 rpm and irradiated for 15 h while maintaining the temperature at approximately 35 °C through cooling with a fan. After irradiation, volatiles were removed from the reaction mixture under reduced pressure. DCM (15 mL) was added to the residue, and the precipitate was removed by filtration through a glass frit. The filtrate was concentrated under reduced pressure, and the residue was purified by flash column chromatography on silica gel (EA/pentane = 1/4, v/v) to yield (4-Bromophenyl)((2,5-dichlorophenyl)imino)(methyl)-λ6-sulfanone (**30**) (44.4 mg, 116 µmol, 58%) as a colorless solid.

R*f* = 0.5 (EA/pentane = 1/4, v/v).

NMR Spectroscopy:

[1]**H NMR** (500 MHz, $CDCl_3$, 298 K, δ): 7.88 (d, J = 8.6 Hz, 2H), 7.69 (d, J = 8.7 Hz, 2H), 7.22 (d, J = 8.5 Hz, 1H), 7.19 (d, J = 2.4 Hz, 1H), 6.82 (dd, J = 8.5, 2.4 Hz, 1H), 3.27 (s, 3H). ppm.

[13]**C NMR** (126 MHz, $CDCl_3$, 298 K, δ): 142.9, 137.7, 133.0, 132.5, 130.1, 129.2, 127.0, 123.5, 123.0, 45.9. ppm.

HRMS-EI (m/z) calculated for $C_{13}H_{10}NOSBrCl_2^+$ $[M]^+$, 376.9038; found, 376.9041; deviation: –0.8 ppm.

DFT calculation

Method

Density Functional Theory (DFT) calculations were performed on the Max-Planck-Institut für Kohlenforschung computer cluster using the ORCA program package (Version 5.0-Stable)[10]. Unless denoted otherwise, structural optimizations were performed with the B3LYP functional[11,12] with D3 dispersion[13] correction and Becke-Johnson damping (BJ)[14] along with RI approximation, utilizing the def2/J auxiliary basis set[15] and the def2-TZVPP basis set[16] on all atoms. The libint2 library was used for the computation of 2-el integrals.[17] Tight SCF convergence and geometry optimization criterions were chosen. The reported energies are enthalpies in gas phase. Input files and images were created using Avogadro 1.2.[18]

Bond disscociation energy of different *NH*-sulfoximines

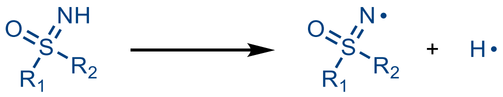

Entry	R_1, R_2	BDE_{N-H}(kcal/mol)
1	R_1 = Me, R_2 = Me	105.6
2	R_1 = Me, R_2 = Ph	104.2
3	R_1 = Ph, R_2 = Ph	104.3

Cartesian coordinates of the optimized species

$Me_2S(O)NH$

C	0.22419	-2.51719	0.04548
H	-0.16594	-3.02724	0.92285
H	-0.11518	-3.00761	-0.86331
H	1.30773	-2.46048	0.09276
C	0.19672	-0.13884	-1.41884
H	-0.17468	0.88430	-1.43734
H	1.28296	-0.14340	-1.37761
H	-0.17594	-0.68054	-2.28535
O	-1.90356	-0.94412	-0.12109
H	-0.18601	0.65098	1.53785
N	0.16226	-0.28224	1.34180
S	-0.46544	-0.87545	0.08928

$Me_2S(O)N\cdot$

C	0.21919	-2.56124	0.05577

H	-0.09263	-3.02910	0.98725
H	-0.21629	-3.09056	-0.78845
H	1.30206	-2.51001	0.00042
C	0.22860	-0.08169	-1.35826
H	-0.07517	0.96048	-1.29317
H	1.31018	-0.16897	-1.35288
H	-0.21958	-0.54672	-2.23376
O	-1.89822	-1.03772	-0.11368
N	0.20100	-0.15200	1.26998
S	-0.46693	-0.89401	0.09110

MePhS(O)NH

C	0.33189	-2.46455	-0.03719
H	-0.02212	-3.00956	0.83687
H	0.02065	-2.97493	-0.94525
H	1.41086	-2.34084	-0.00097
O	-1.86789	-1.10251	-0.18412
S	-0.45289	-0.84019	0.03077
C	0.21601	-0.03604	-1.40763
C	-0.47210	-0.18520	-2.60573
C	1.39200	0.70185	-1.33396
C	0.03384	0.41039	-3.75460
H	-1.39885	-0.74076	-2.62291
C	1.88839	1.29252	-2.48869
H	1.88421	0.82088	-0.38060
C	1.21338	1.14430	-3.69655
H	-0.49692	0.30713	-4.69113
H	2.79901	1.87430	-2.44402
H	1.60377	1.60828	-4.59241
H	-0.17806	-0.38707	2.08566
N	0.08005	-0.00180	1.18287

MePhS(O)N·

C	0.43304	-2.41029	0.12231
H	0.12894	-2.84244	1.07328
H	0.16441	-3.07104	-0.69845
H	1.49092	-2.17025	0.12427
O	-1.88563	-1.30551	-0.44426
N	-0.14657	-0.05988	1.15323
S	-0.53690	-0.90413	-0.07913

C	0.16112	-0.07817	-1.50961
C	-0.55780	-0.08581	-2.69890
C	1.39029	0.56424	-1.40338
C	-0.02235	0.55421	-3.81009
H	-1.51836	-0.57808	-2.73494
C	1.91717	1.19466	-2.52227
H	1.90405	0.58038	-0.45333
C	1.21226	1.18856	-3.72254
H	-0.57117	0.55917	-4.74190
H	2.87136	1.69892	-2.45506
H	1.62402	1.68630	-4.59017

$Ph_2S(O)NH$

O	-2.04985	-0.81602	-0.01707
C	0.14853	-0.14689	-1.29639
C	-0.55403	-0.26295	-2.49025
C	1.40527	0.44425	-1.25024
C	0.01775	0.21747	-3.66088
H	-1.53848	-0.70770	-2.48952
C	1.96647	0.92344	-2.42684
H	1.91130	0.53840	-0.30170
C	1.27727	0.80663	-3.62982
H	-0.52216	0.13674	-4.59440
H	2.94099	1.39190	-2.40314
H	1.71976	1.18102	-4.54319
H	-0.15802	-0.45125	2.20457
N	0.10393	-0.08070	1.29623
S	-0.60678	-0.81399	0.16810
C	-0.12021	-2.54479	0.09905
C	1.15391	-2.90887	0.52146
C	-1.01797	-3.49379	-0.37553
C	1.53469	-4.24275	0.46177
H	1.82800	-2.15218	0.89715
C	-0.62907	-4.82680	-0.43065
H	-2.00615	-3.18258	-0.68038
C	0.64482	-5.20021	-0.01605
H	2.52295	-4.53546	0.78962
H	-1.32188	-5.57370	-0.79409
H	0.94399	-6.23879	-0.06138

$Ph_2S(O)N\cdot$

O	-2.11563	-0.94738	-0.26380
C	0.09824	-0.16744	-1.41472
C	-0.67742	0.04831	-2.54737
C	1.44763	0.16890	-1.37956
C	-0.08091	0.60109	-3.67362
H	-1.72551	-0.21077	-2.52946
C	2.03148	0.72020	-2.51161
H	2.01645	0.01393	-0.47573
C	1.26925	0.93406	-3.65618
H	-0.67195	0.77341	-4.56271
H	3.07873	0.98926	-2.49800
H	1.72812	1.36677	-4.53497
N	-0.02538	-0.18178	1.21845
S	-0.68998	-0.89248	0.02196
C	-0.11947	-2.59095	0.10059
C	1.14033	-2.88285	0.61276
C	-0.96566	-3.59127	-0.36423
C	1.56293	-4.20516	0.64440
H	1.76198	-2.08652	0.99251
C	-0.52943	-4.90951	-0.33074
H	-1.94522	-3.32877	-0.73493
C	0.73208	-5.21507	0.16982
H	2.53801	-4.44698	1.04467
H	-1.17586	-5.69742	-0.69252
H	1.06614	-6.24361	0.19605

SPECTROSCOPIC DATA

^{1}H NMR of ((4-fluorophenyl)imino)(methyl)-λ^6-sulfanone (1)

500 MHz, CD_2Cl_2, 298 K

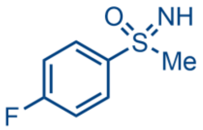

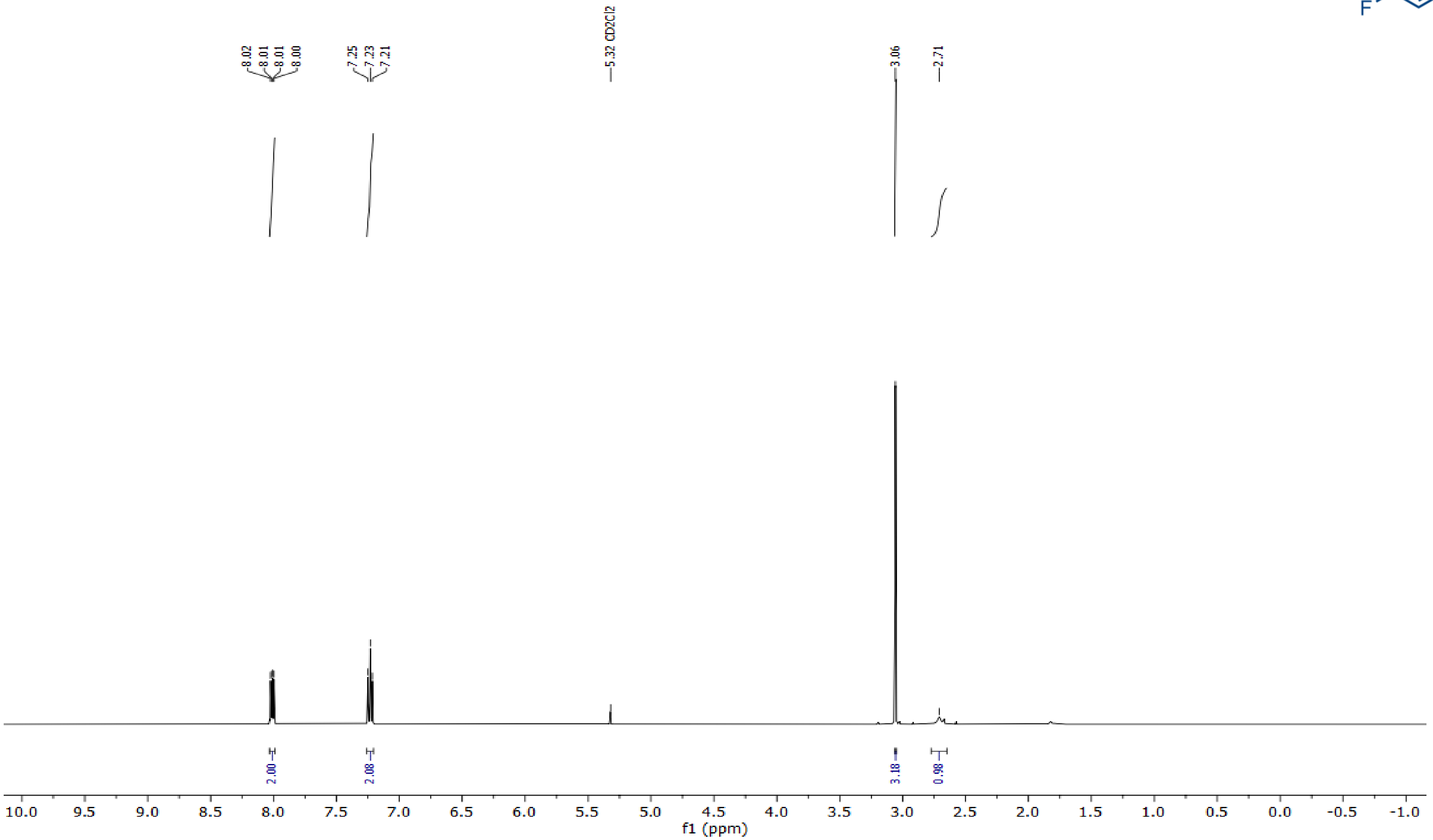

^{13}C NMR of ((4-fluorophenyl)imino)(methyl)-λ^6-sulfanone (1)

126 MHz, CD_2Cl_2, 298 K

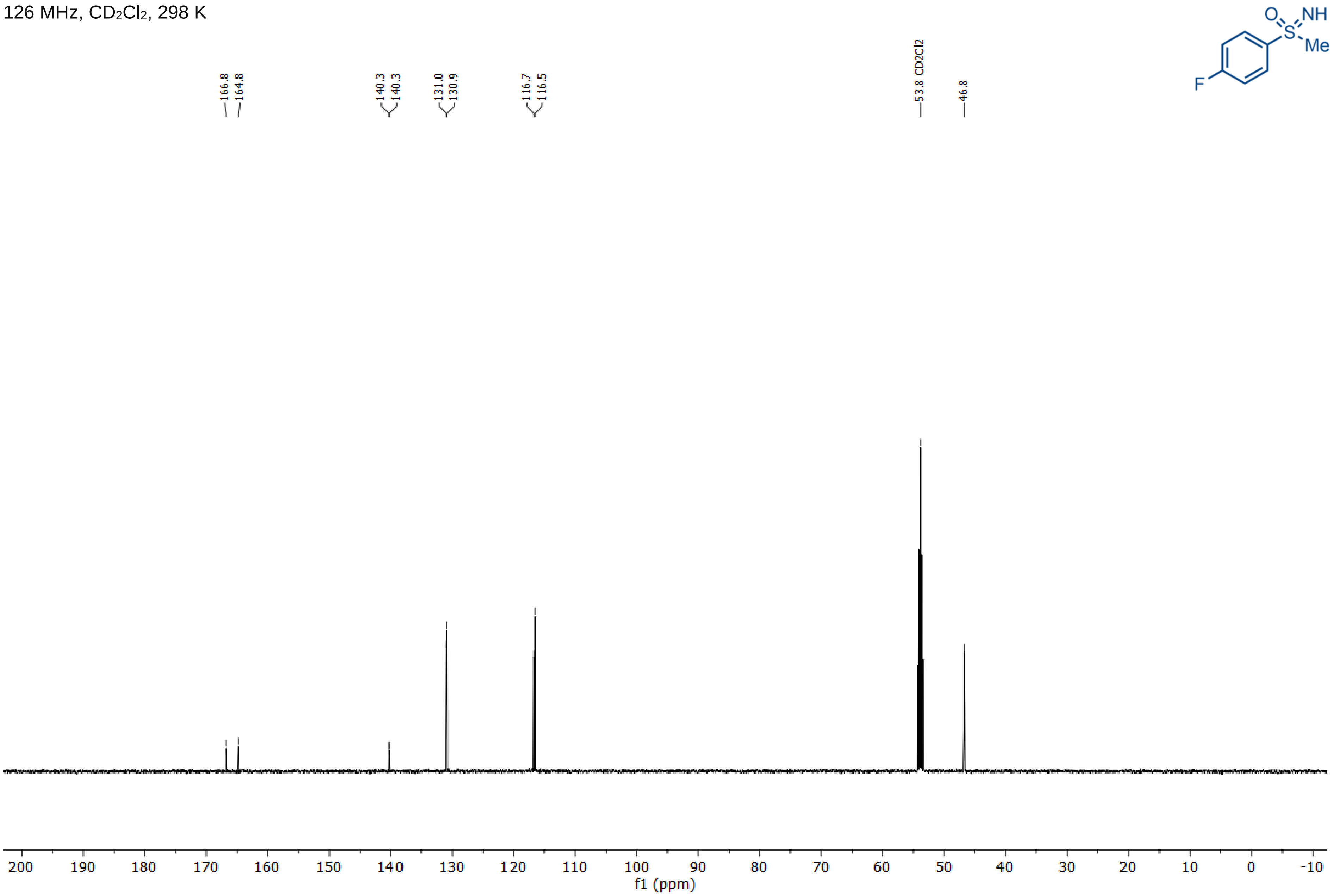

^{19}F NMR of ((4-fluorophenyl)imino)(methyl)-λ^6-sulfanone (1)

471 MHz, CD_2Cl_2, 298 K

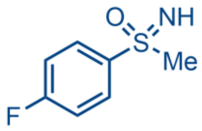

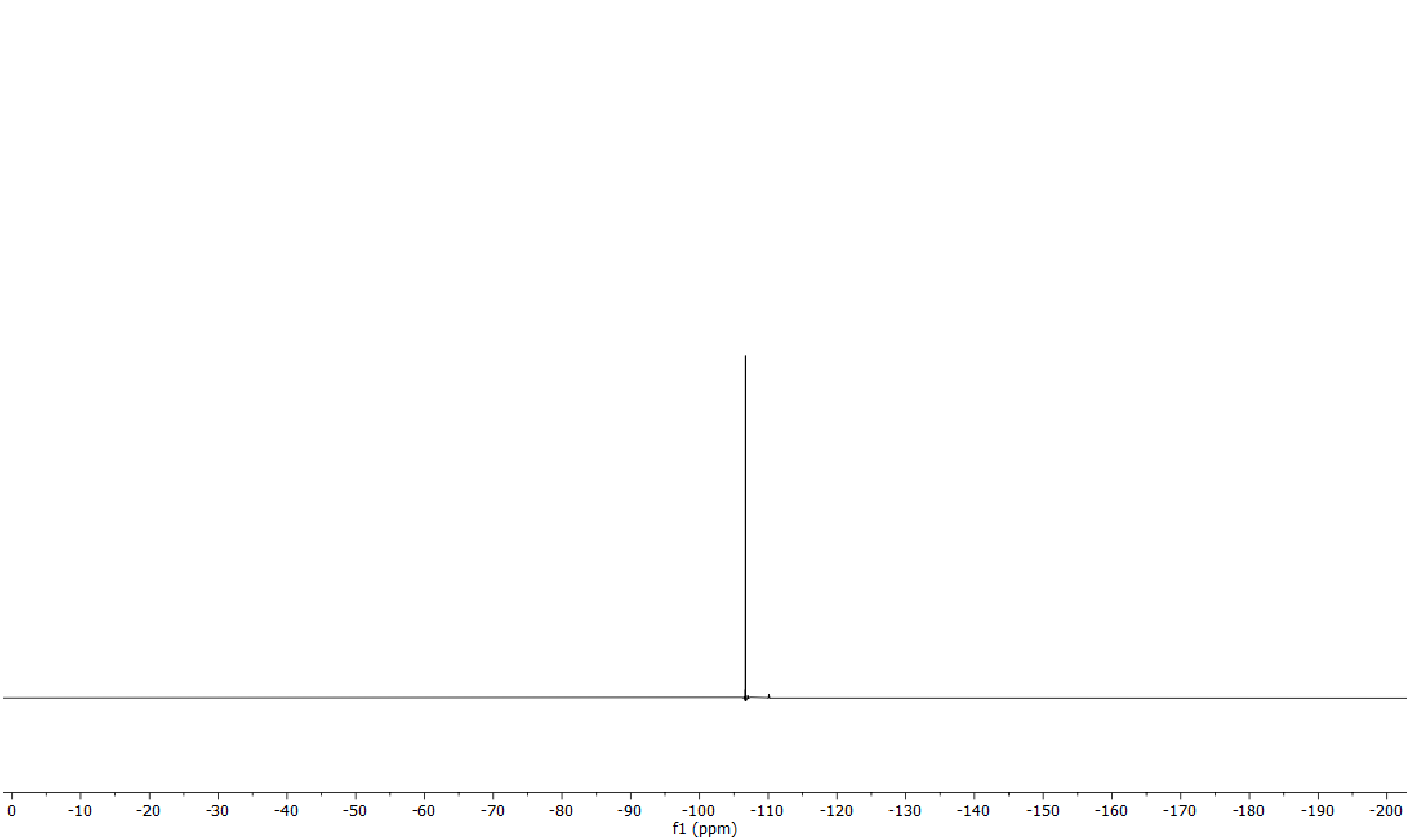

^{1}H NMR of iminodiphenyl-λ[6]-sulfanone (6)

500 MHz, $CDCl_3$, 298 K

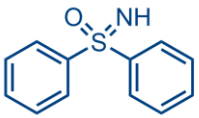

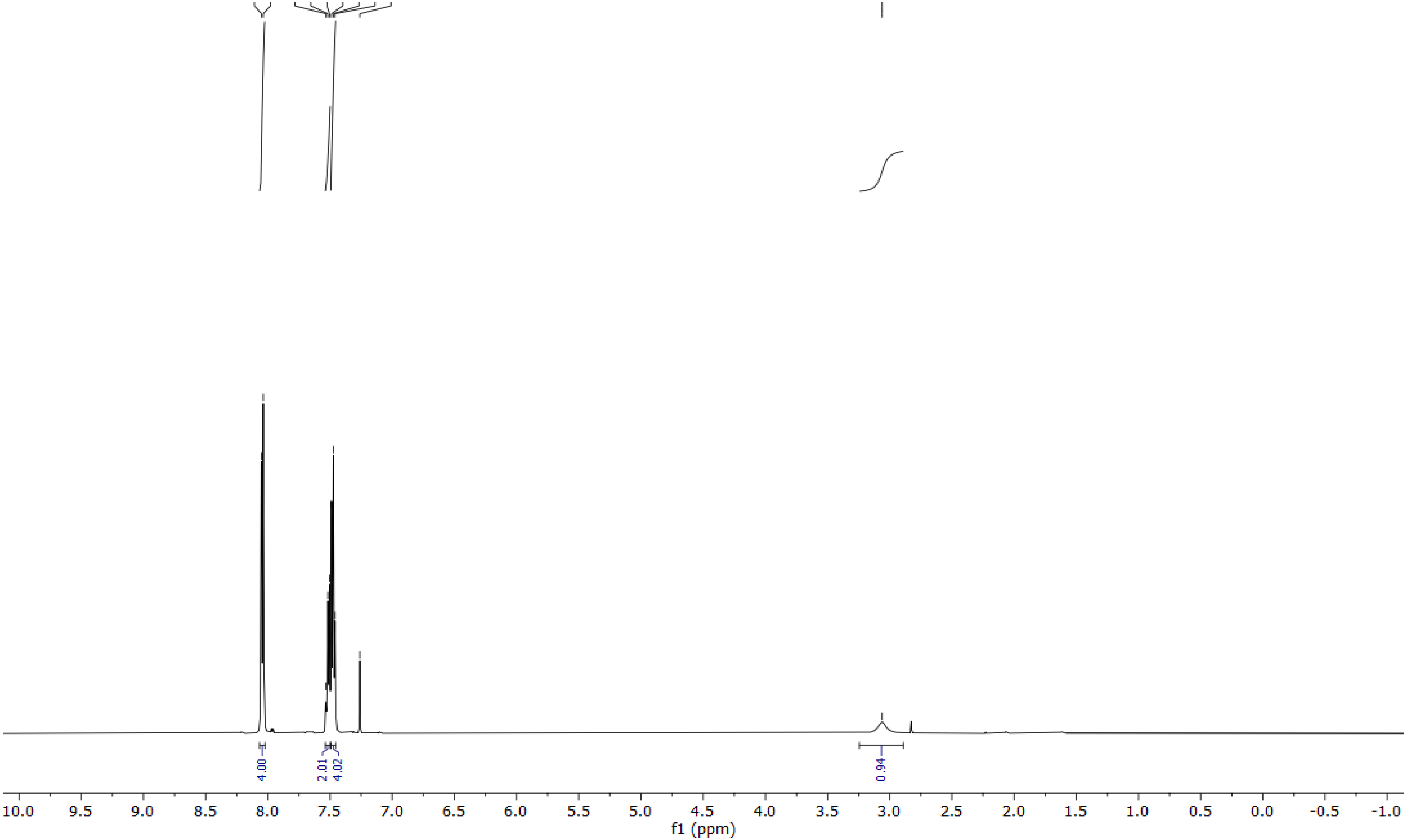

^{13}C NMR of iminodiphenyl-λ^6-sulfanone (6)

126 MHz, $CDCl_3$, 298 K

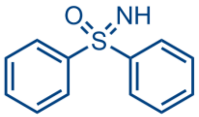

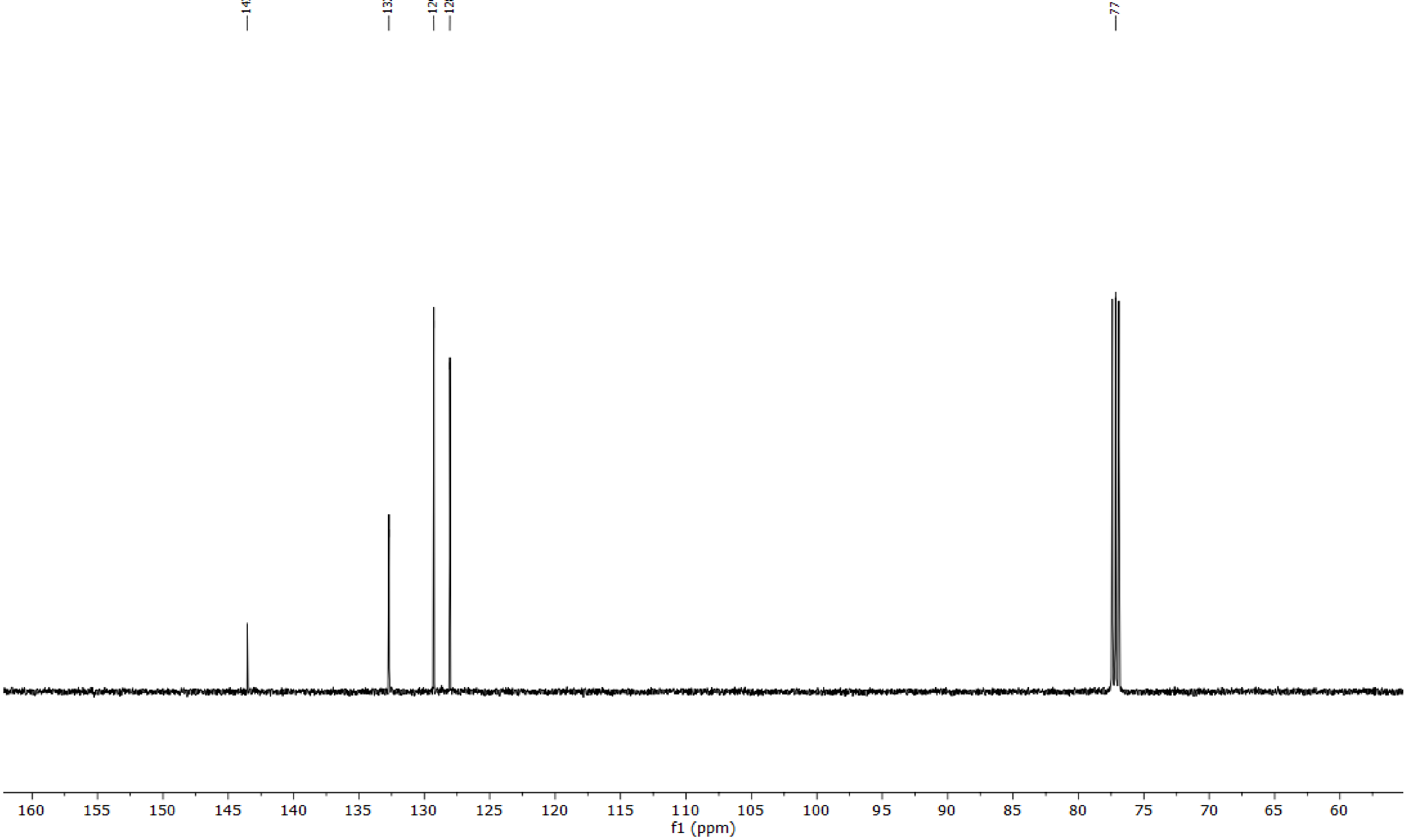

^{1}H NMR of imino(methyl)(phenyl)-λ6-sulfanone (S1)

500 MHz, $CDCl_3$, 298 K

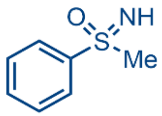

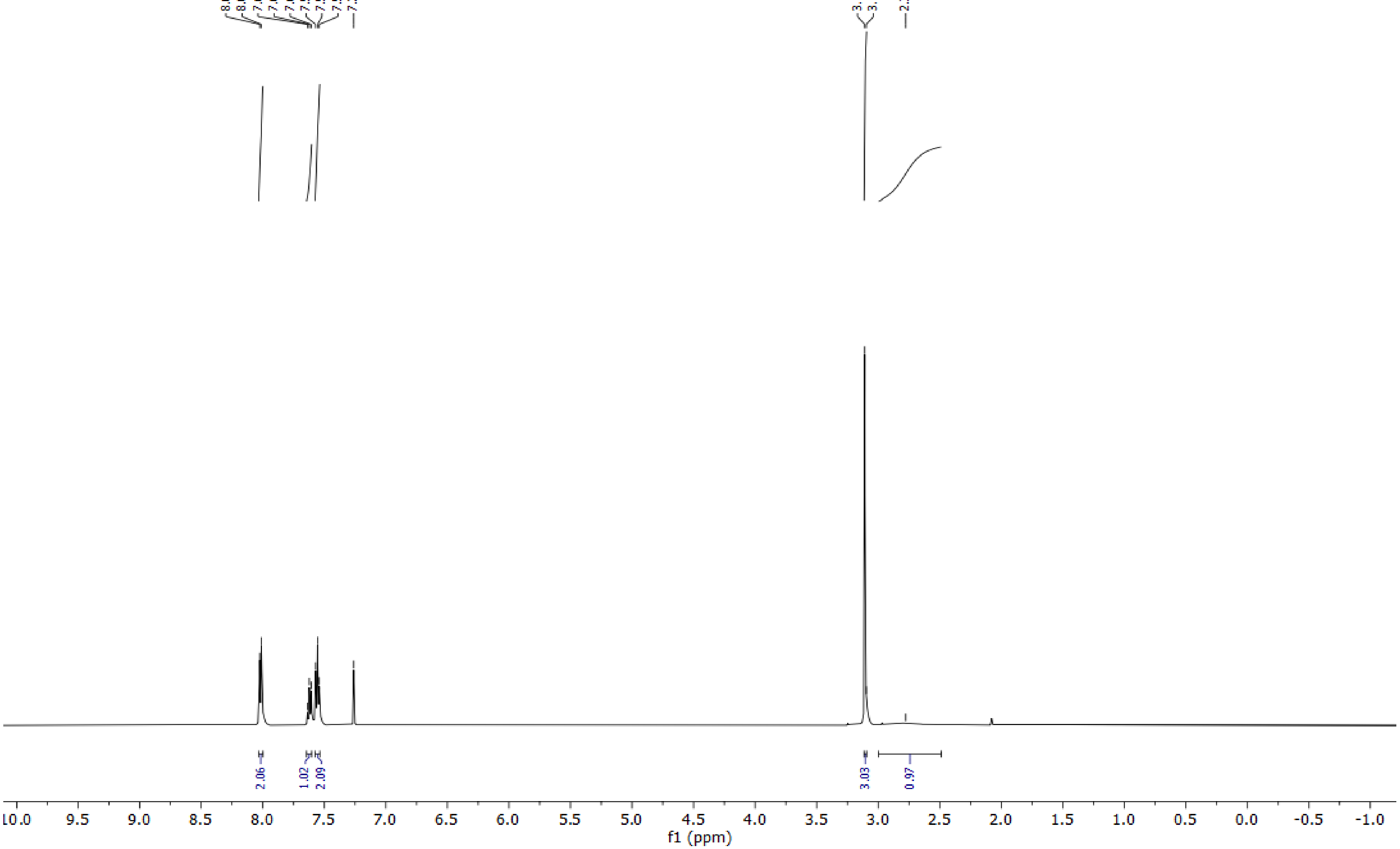

^{13}C NMR of imino(methyl)(phenyl)-λ^6-sulfanone (S1)

126 MHz, $CDCl_3$, 298 K

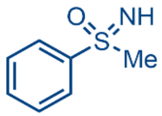

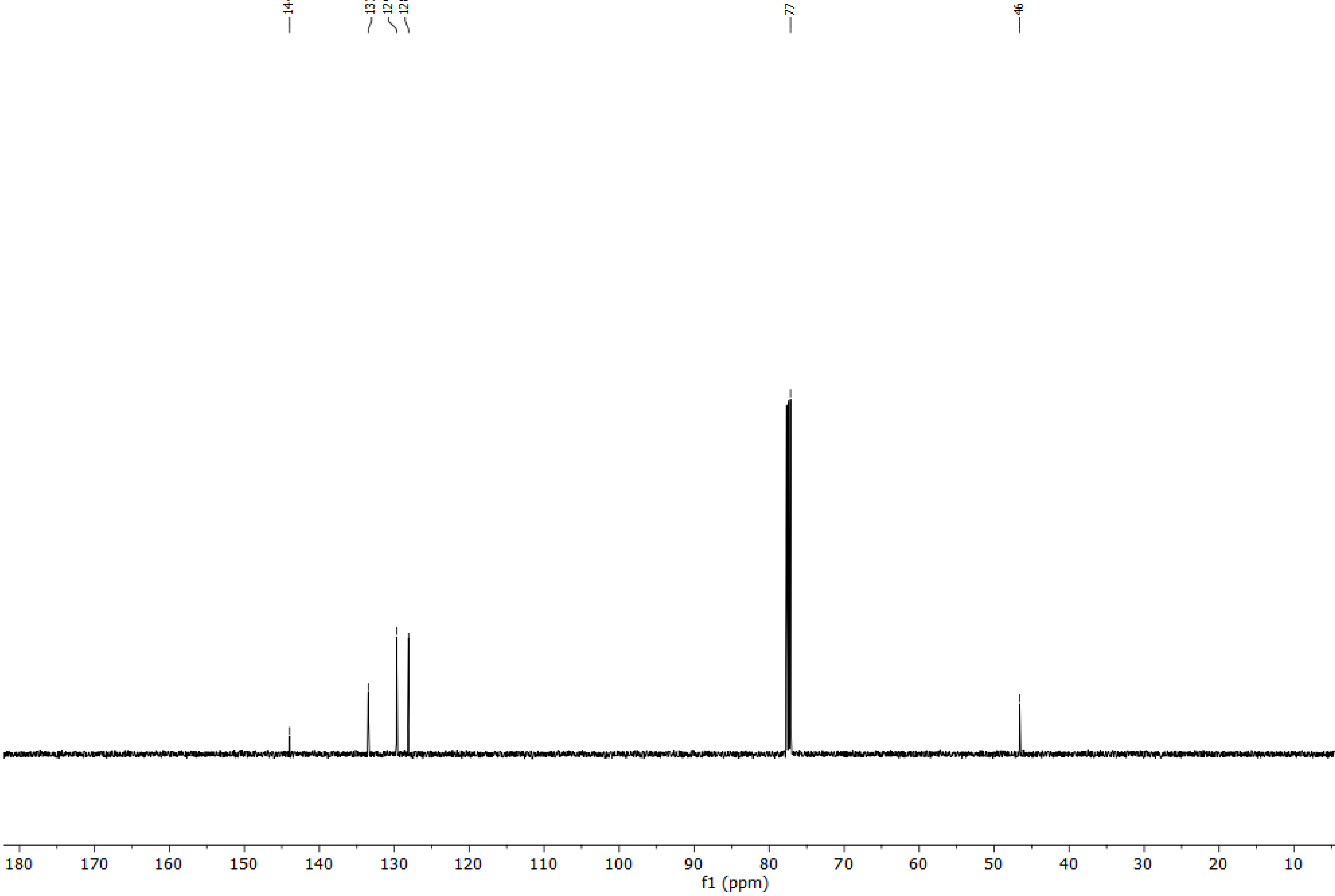

^{1}H NMR of ethyl(imino)(phenyl)-λ[6]-sulfanone (S4)

500 MHz, $CDCl_3$, 298 K

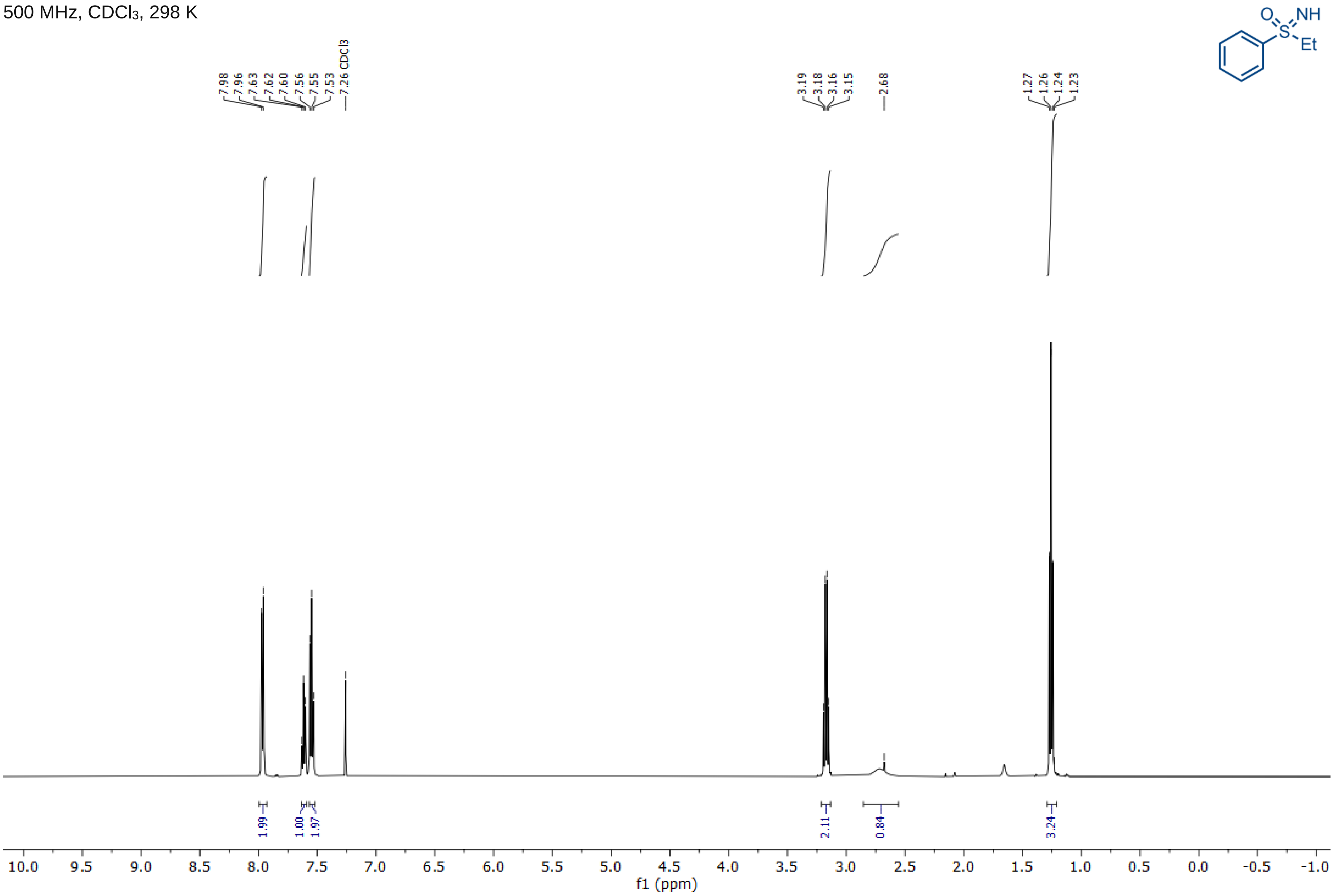

^{13}C NMR of ethyl(imino)(phenyl)-λ^6-sulfanone (S4)

126 MHz, $CDCl_3$, 298 K

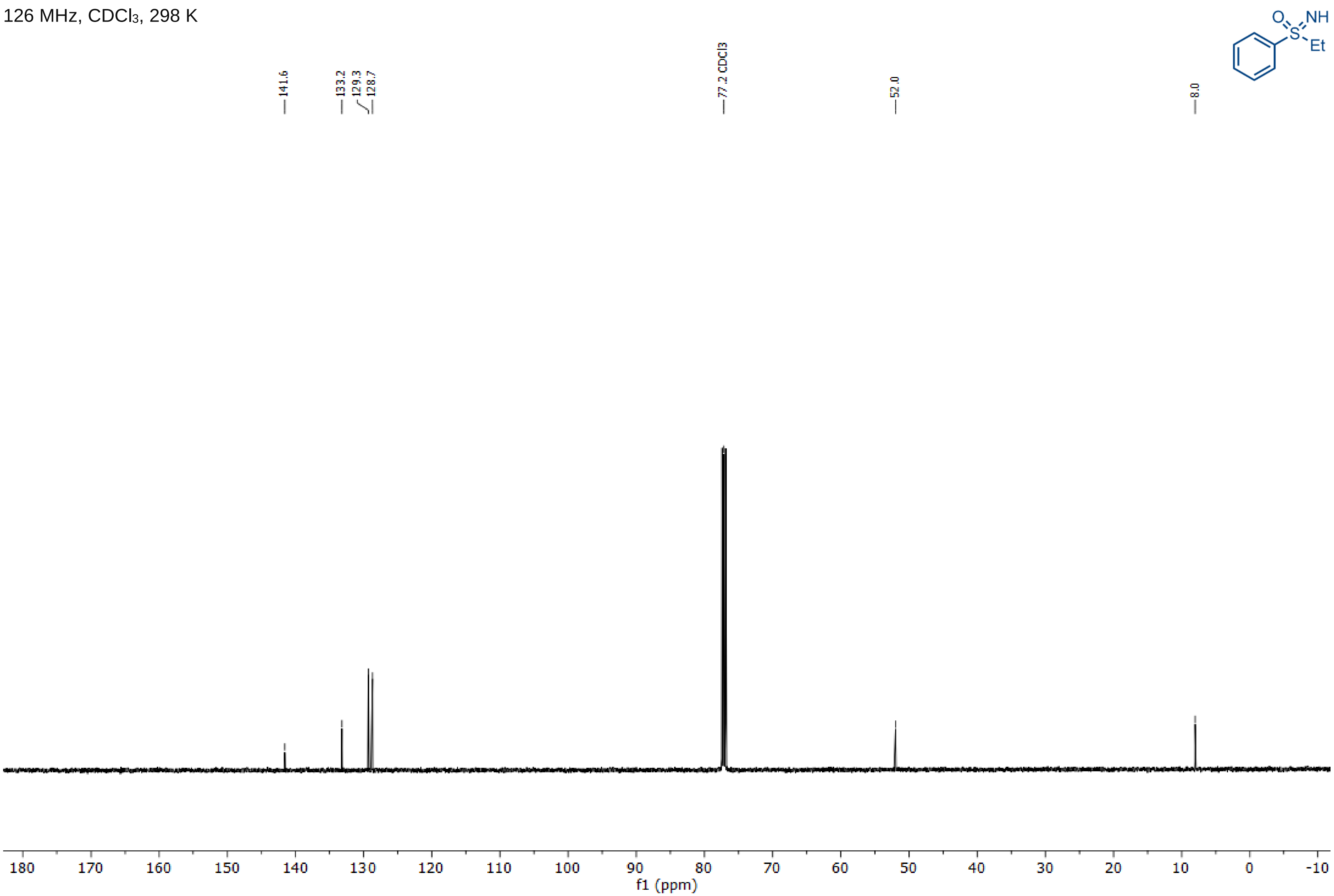

^{1}H NMR of imino(methyl)(m-tolyl)-λ6-sulfanone (S5)

500 MHz, CD_2Cl_2, 298 K

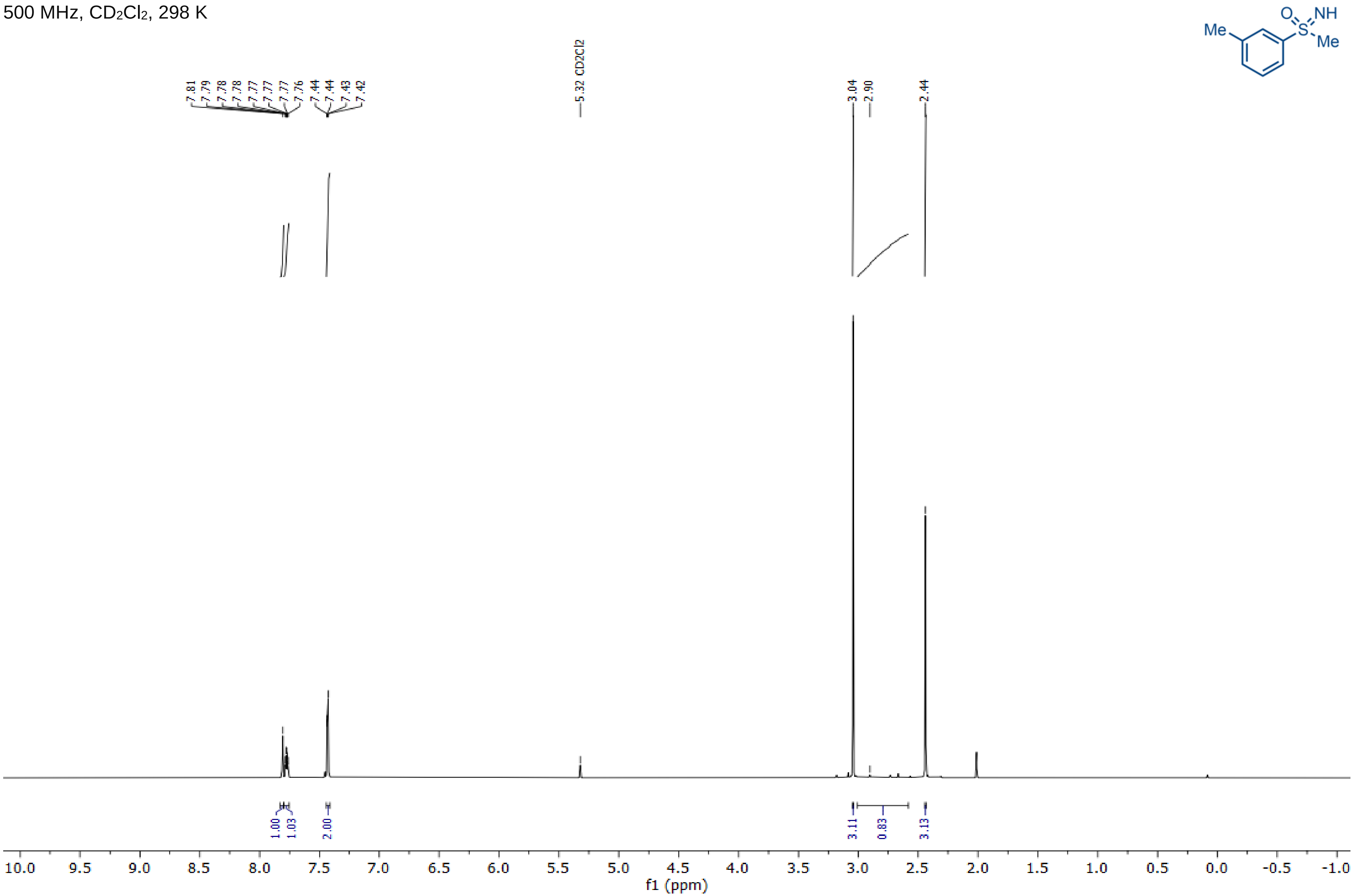

^{13}C NMR of imino(methyl)(m-tolyl)-λ[6]-sulfanone (S5)

126 MHz, CD_2Cl_2, 298 K

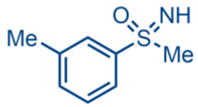

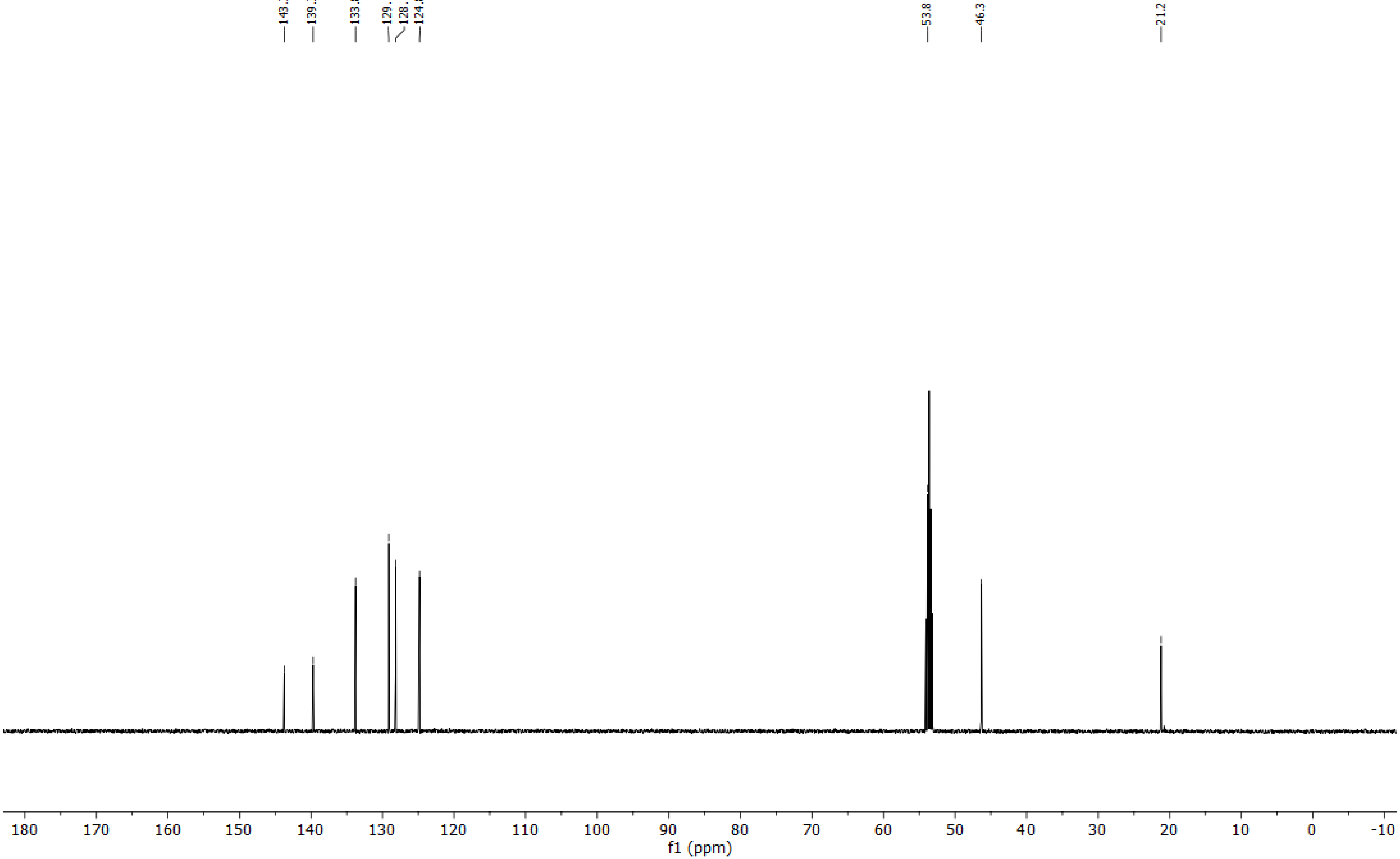

^{1}H NMR of imino(methyl)(4-(trifluoromethoxy)phenyl)-λ[6]-sulfanone (S6)

500 MHz, CD_2Cl_2, 298 K

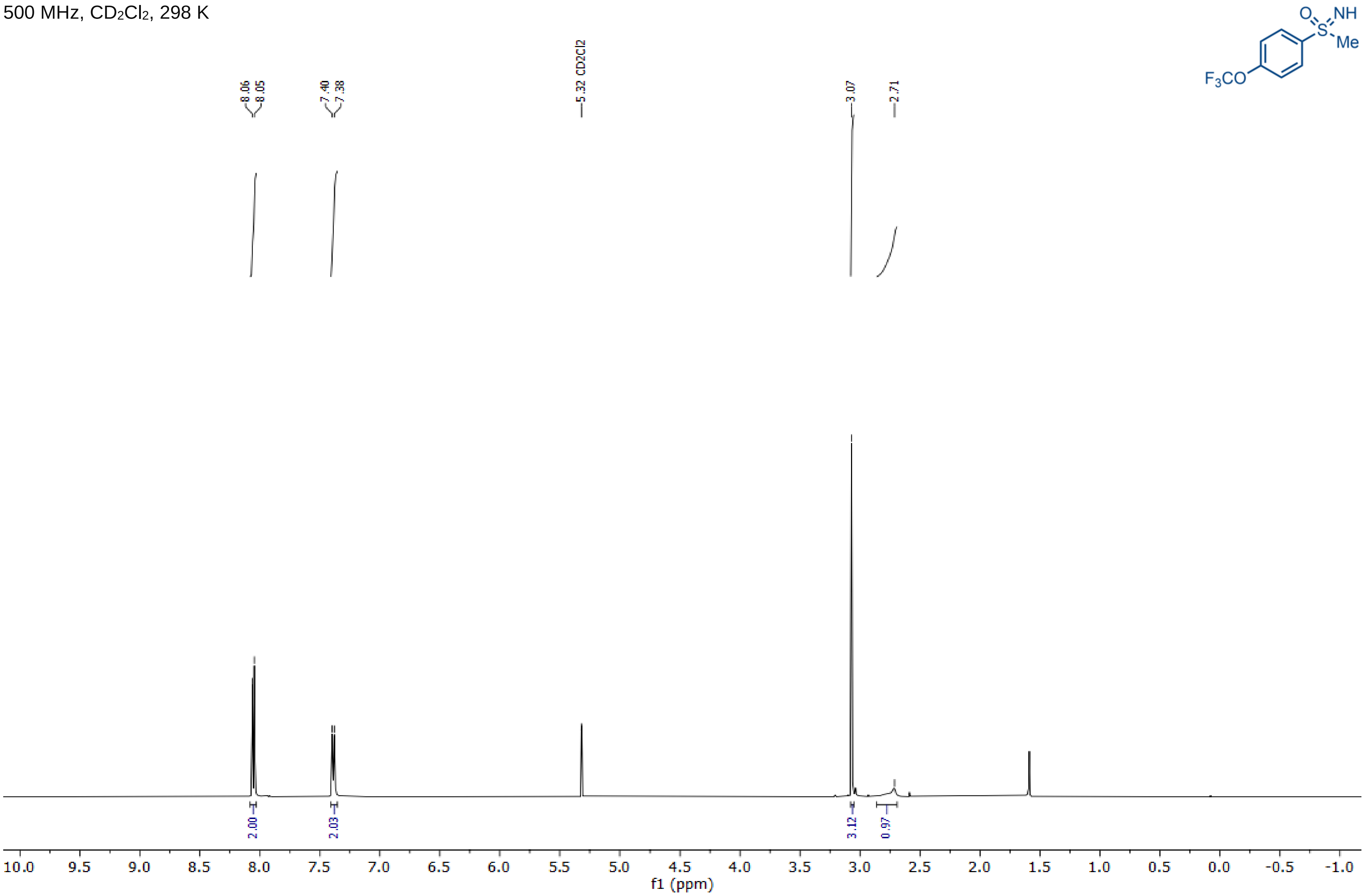

^{13}C NMR of imino(methyl)(4-(trifluoromethoxy)phenyl)-λ^{6}-sulfanone (S6)

126 MHz, CD_2Cl_2, 298 K

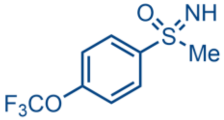

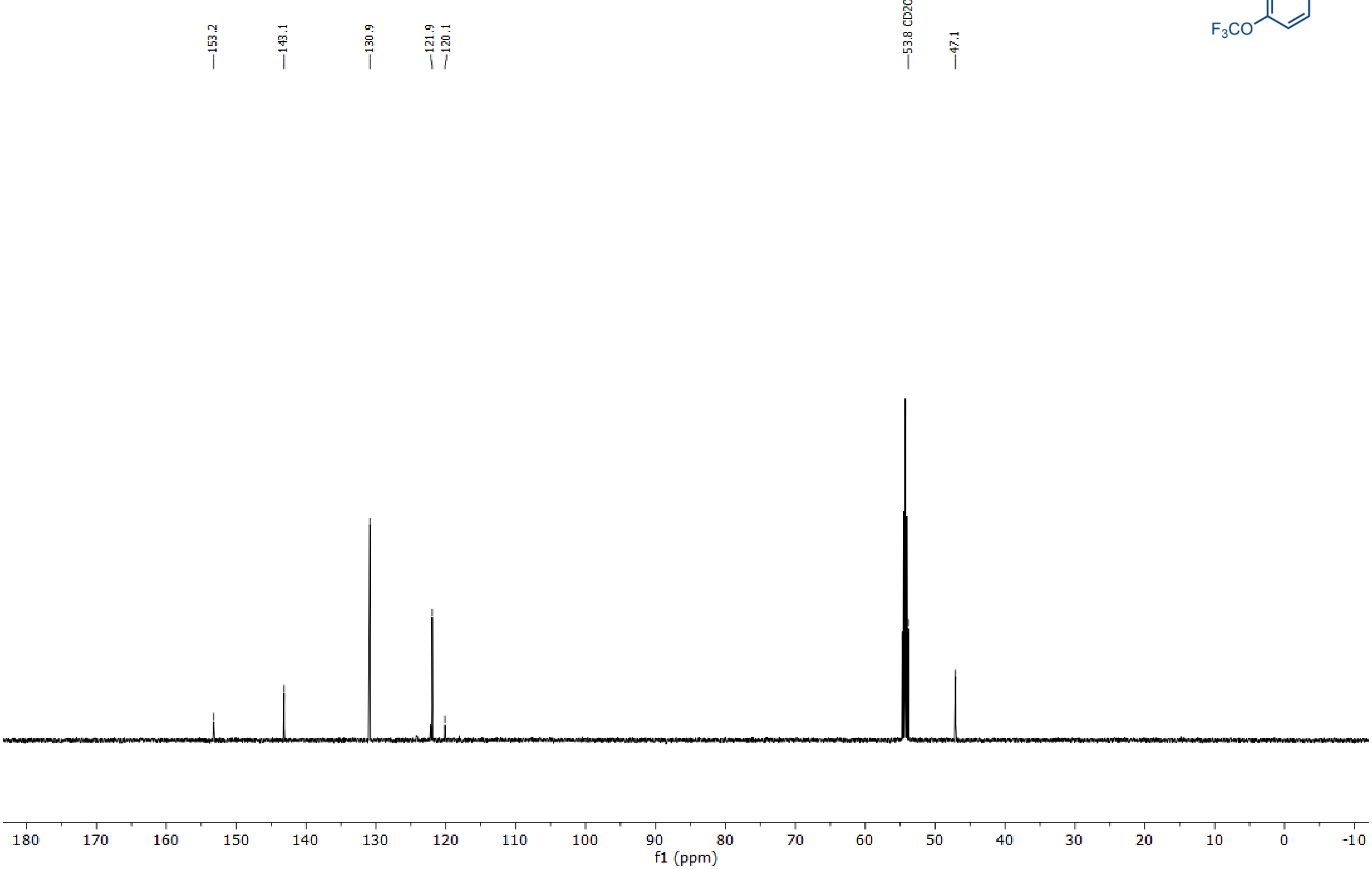

^{19}F NMR of imino(methyl)(4-(trifluoromethoxy)phenyl)-λ[6]-sulfanone (S6)

471 MHz, CD_2Cl_2, 298 K

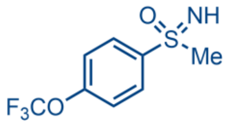

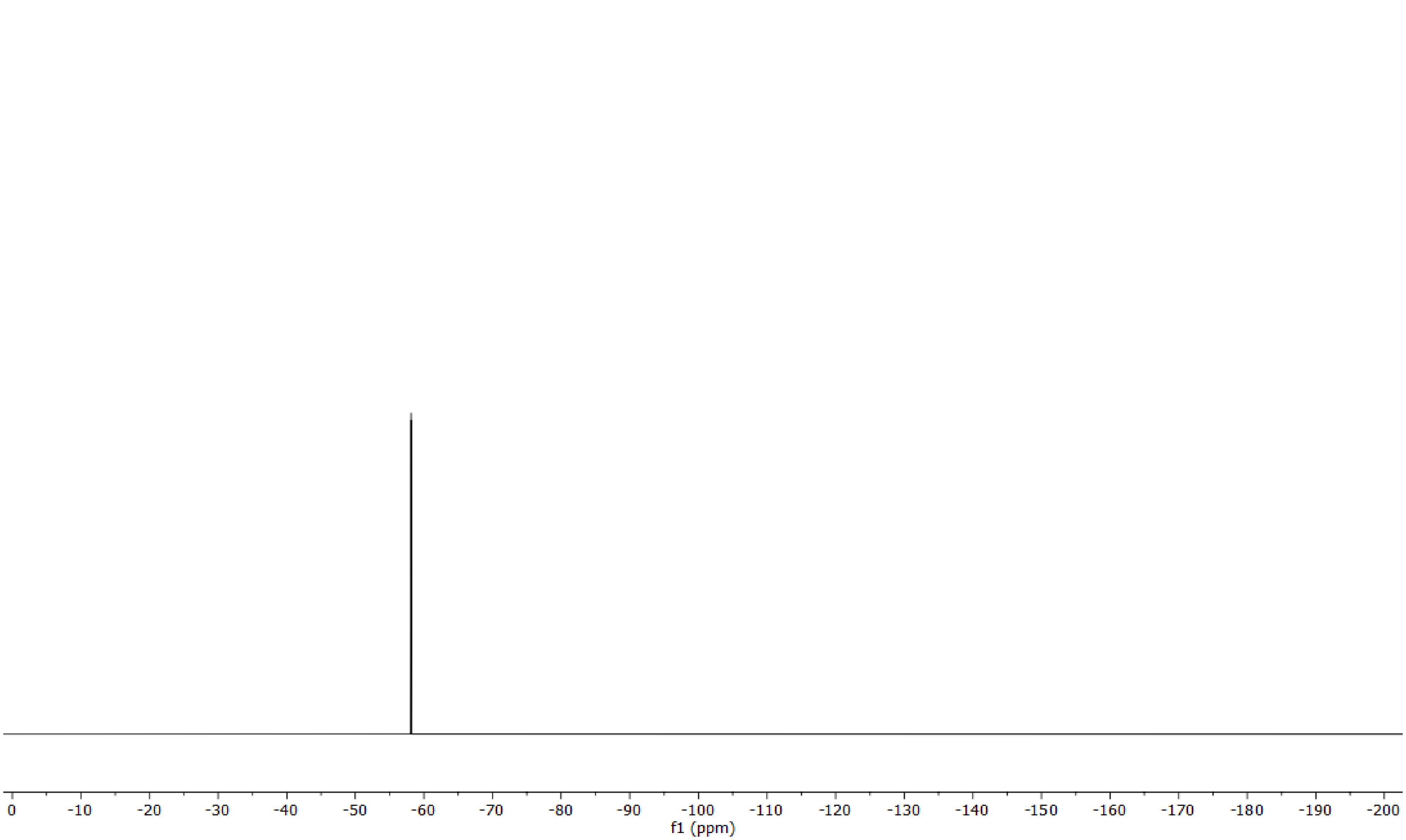

^{1}H NMR of imino(methyl)(thiophen-2-yl)-λ[6]-sulfanone (S7)

500 MHz, $CDCl_3$, 298 K

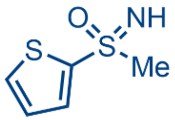

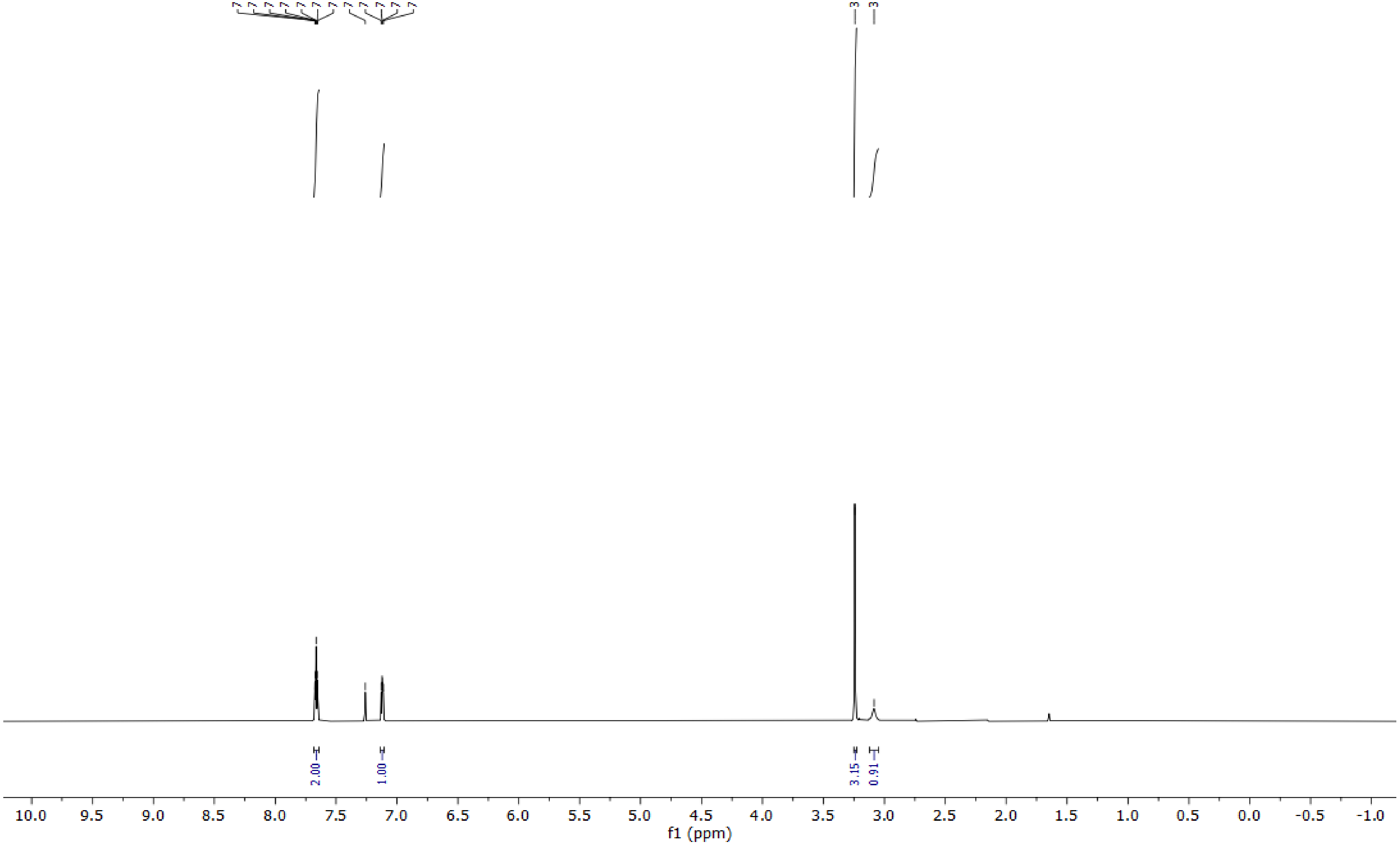

^{13}C NMR of imino(methyl)(thiophen-2-yl)-λ^{6}-sulfanone (S7)

126 MHz, $CDCl_3$, 298 K

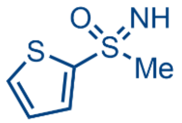

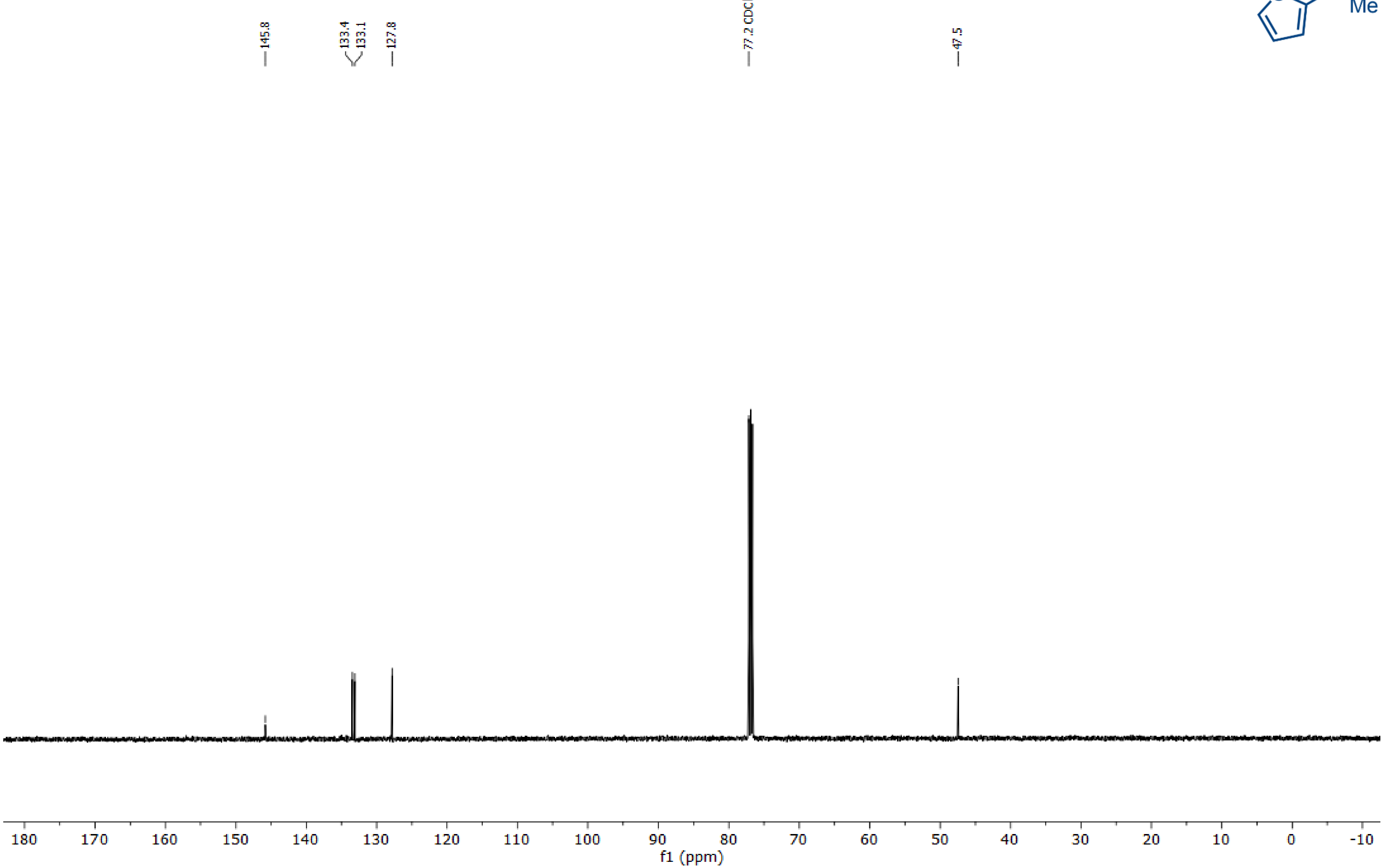

^{1}H NMR of (4-bromophenyl)(imino)(methyl)-λ^6-sulfanone (S8)

500 MHz, CD_2Cl_2, 298 K

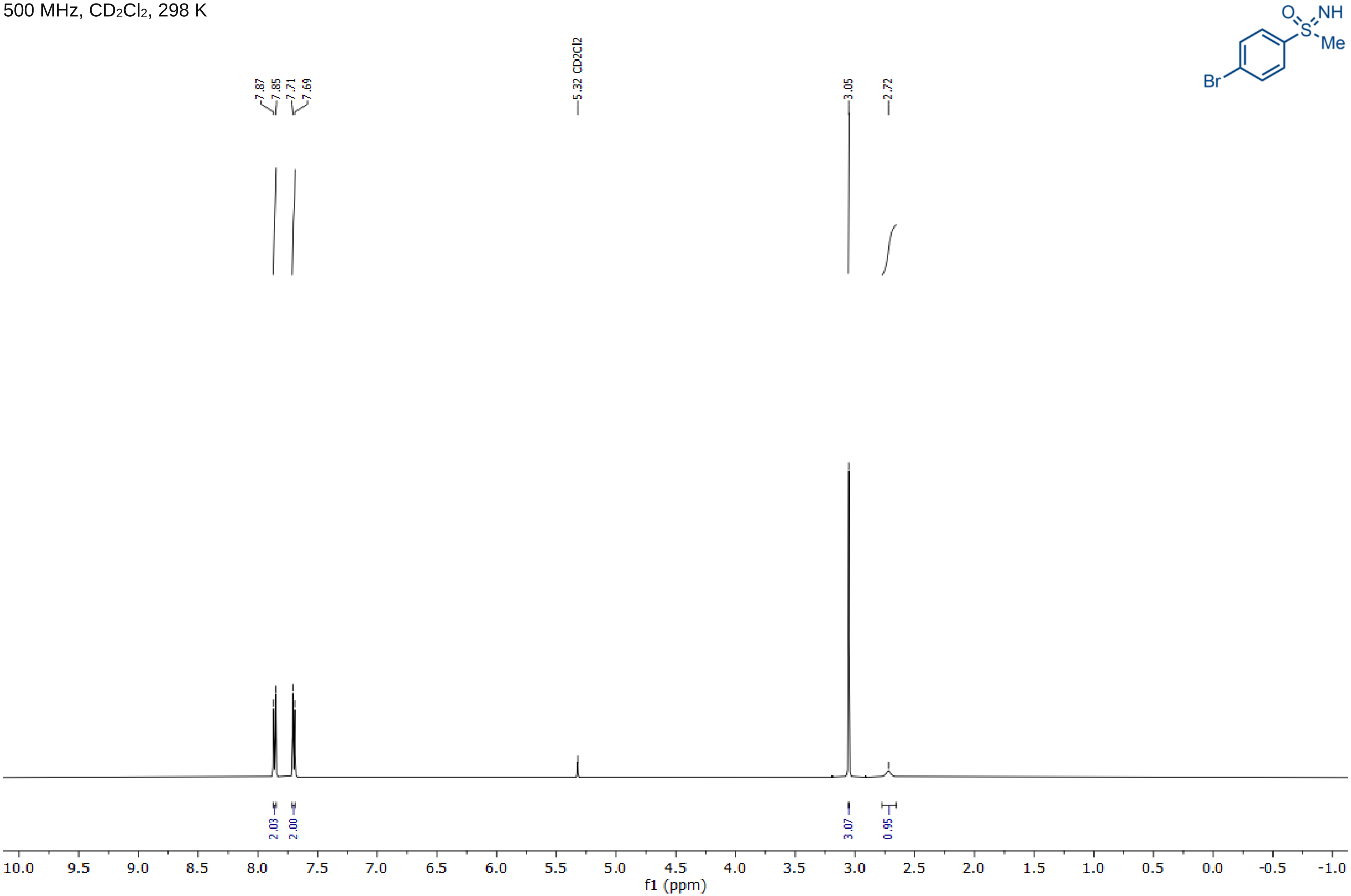

^{13}C NMR of (4-bromophenyl)(imino)(methyl)-λ^6-sulfanone (S8)

126 MHz, CD_2Cl_2, 298 K

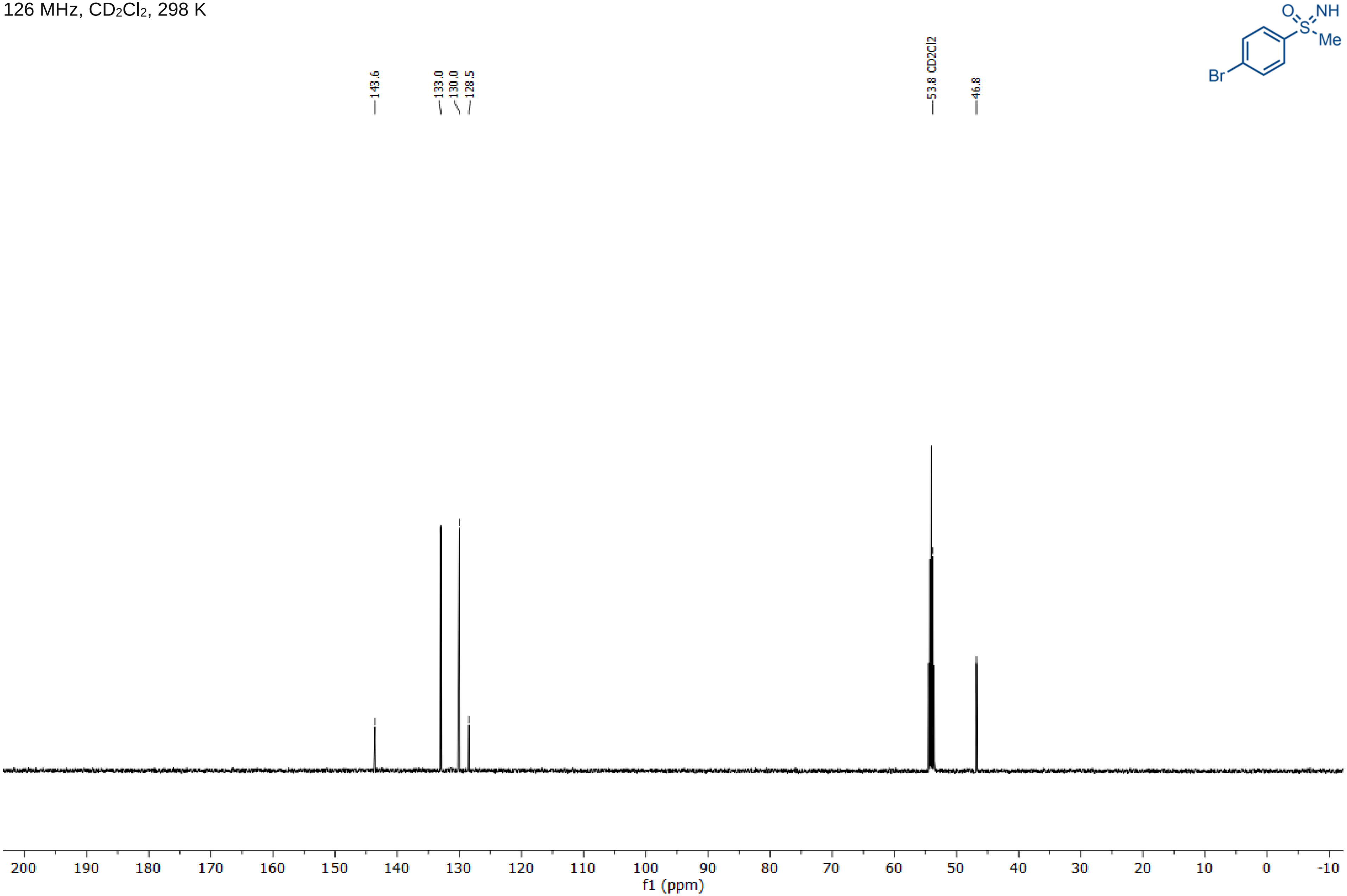

^{1}H NMR of 10-imino-10H-10 λ4-phenoxathiine 10-oxide (S9)

500 MHz, CD_2Cl_2, 298 K

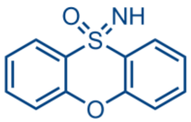

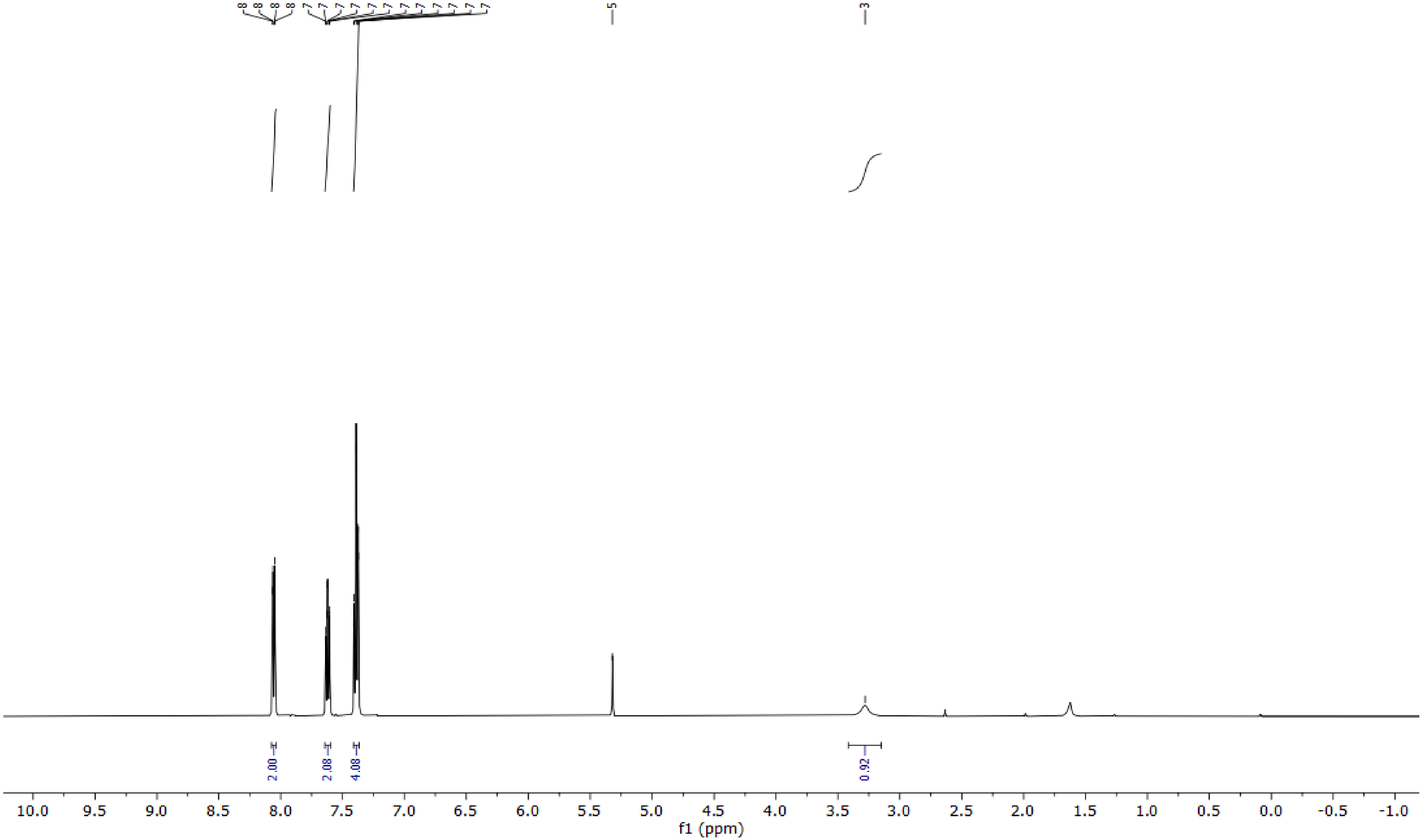

^{13}C NMR of 10-imino-10H-10 λ^4-phenoxathiine 10-oxide (S9)

126 MHz, CD_2Cl_2, 298 K

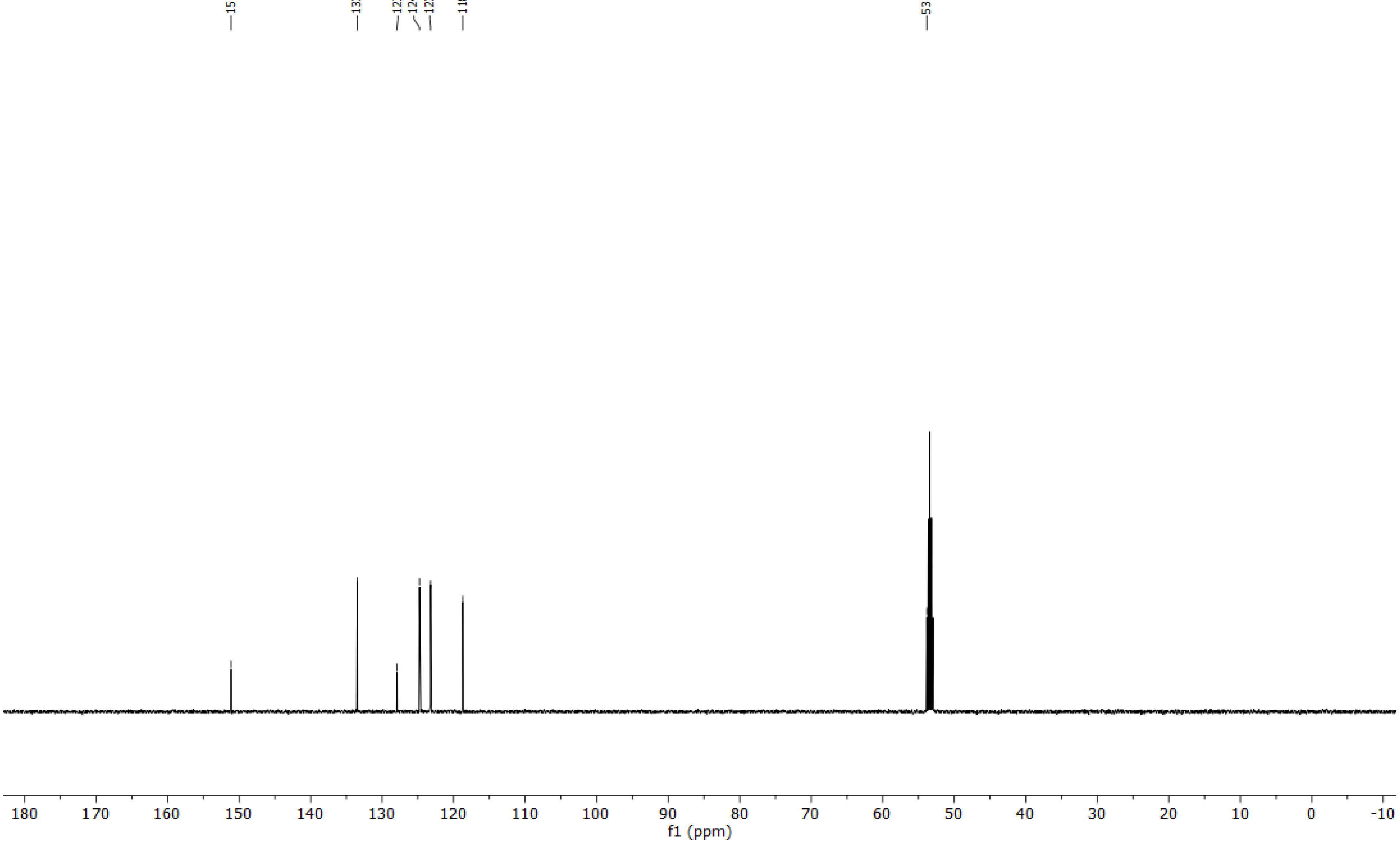

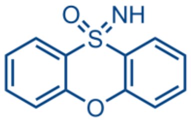

^{1}H NMR of imino(4-iodophenyl)(methyl)-λ[6]-sulfanone (S10)

500 MHz, CD_2Cl_2, 298 K

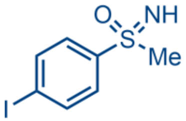

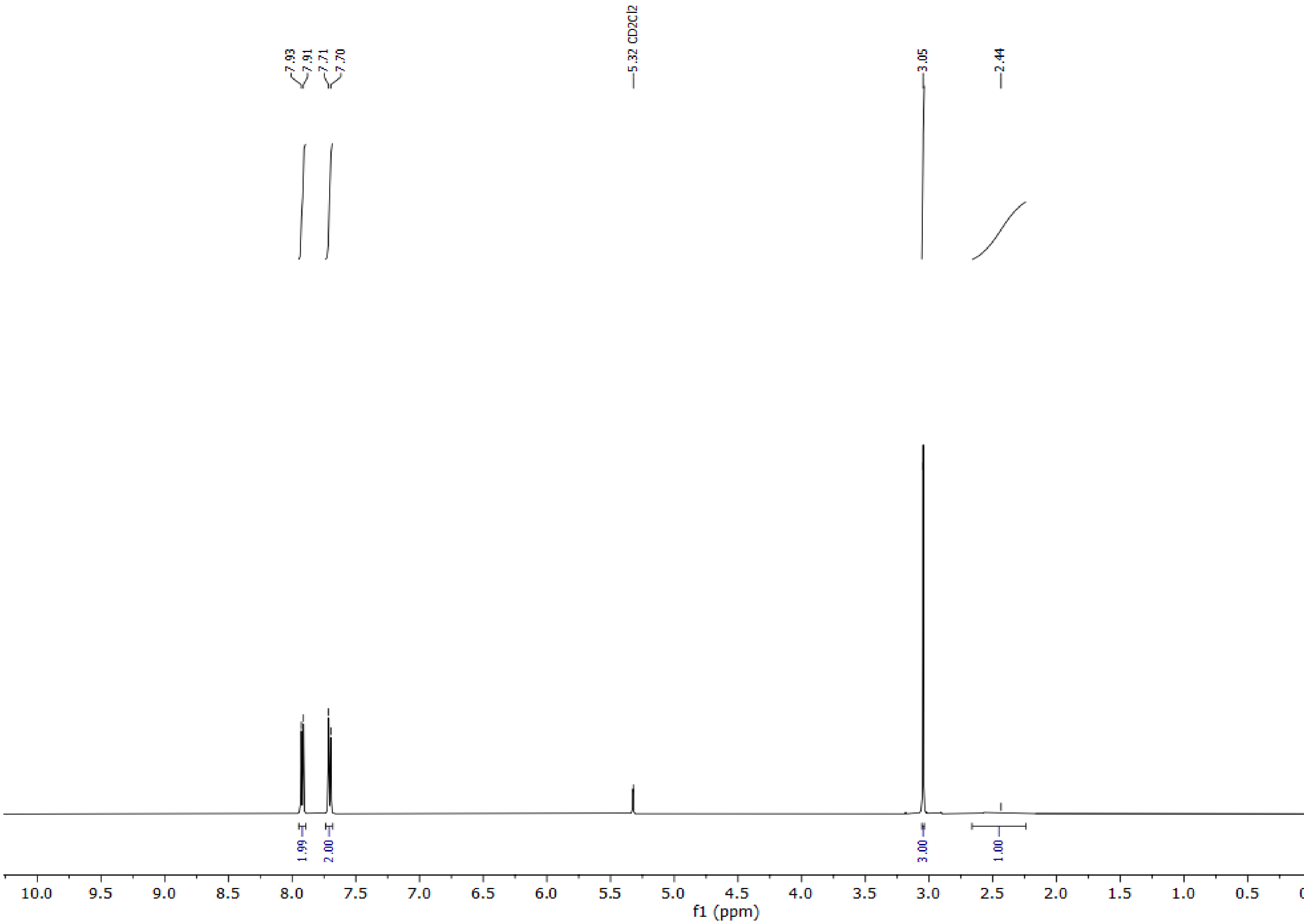

^{13}C NMR of imino(4-iodophenyl)(methyl)-λ^6-sulfanone (S10)

126 MHz, CD_2Cl_2, 298 K

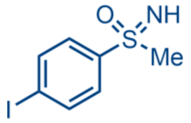

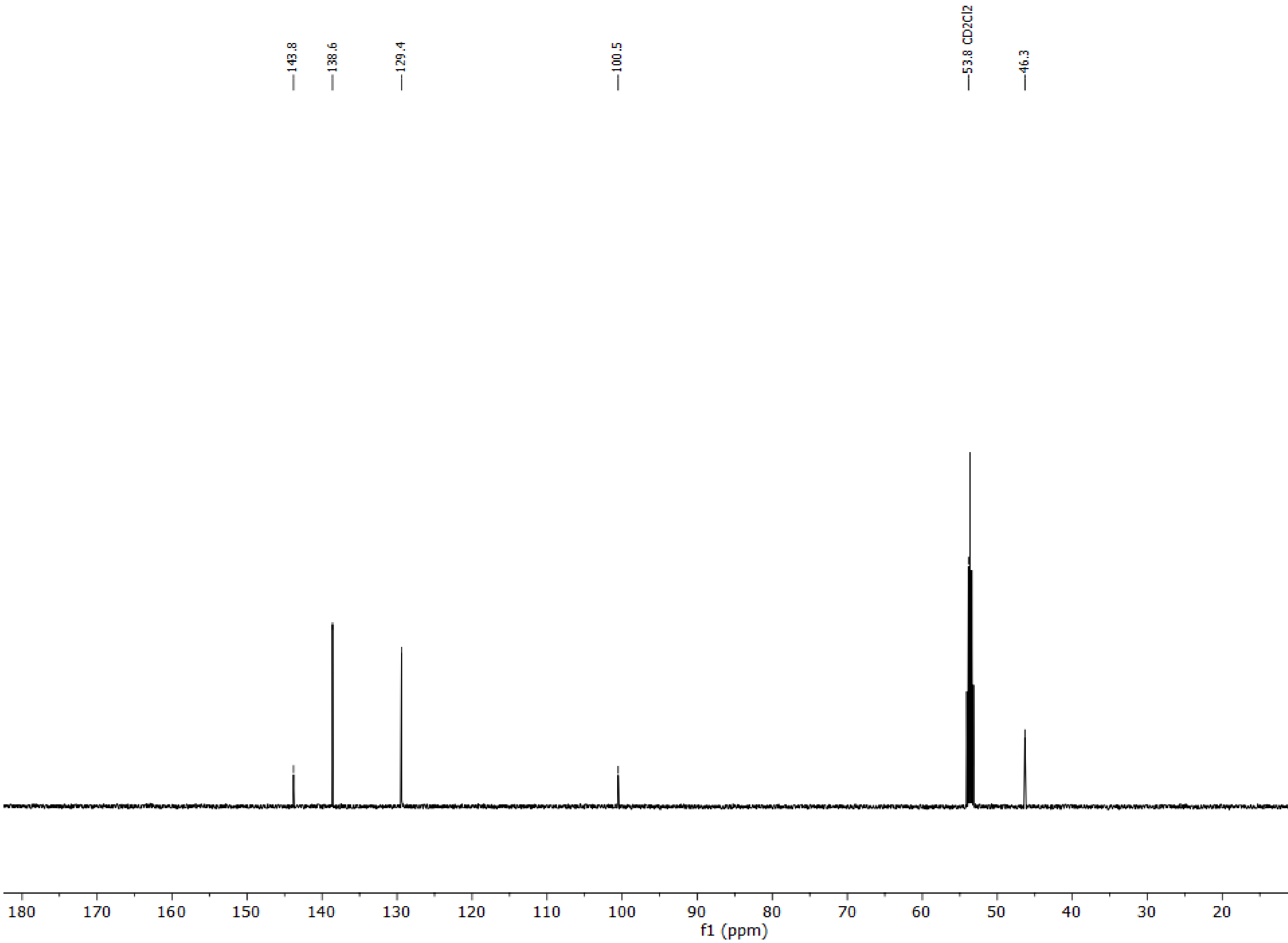

^{1}H NMR of N,N-diphenylmethacrylamide (7)

500 MHz, $CDCl_3$, 298 K

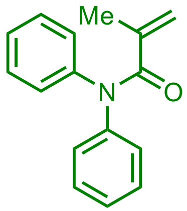

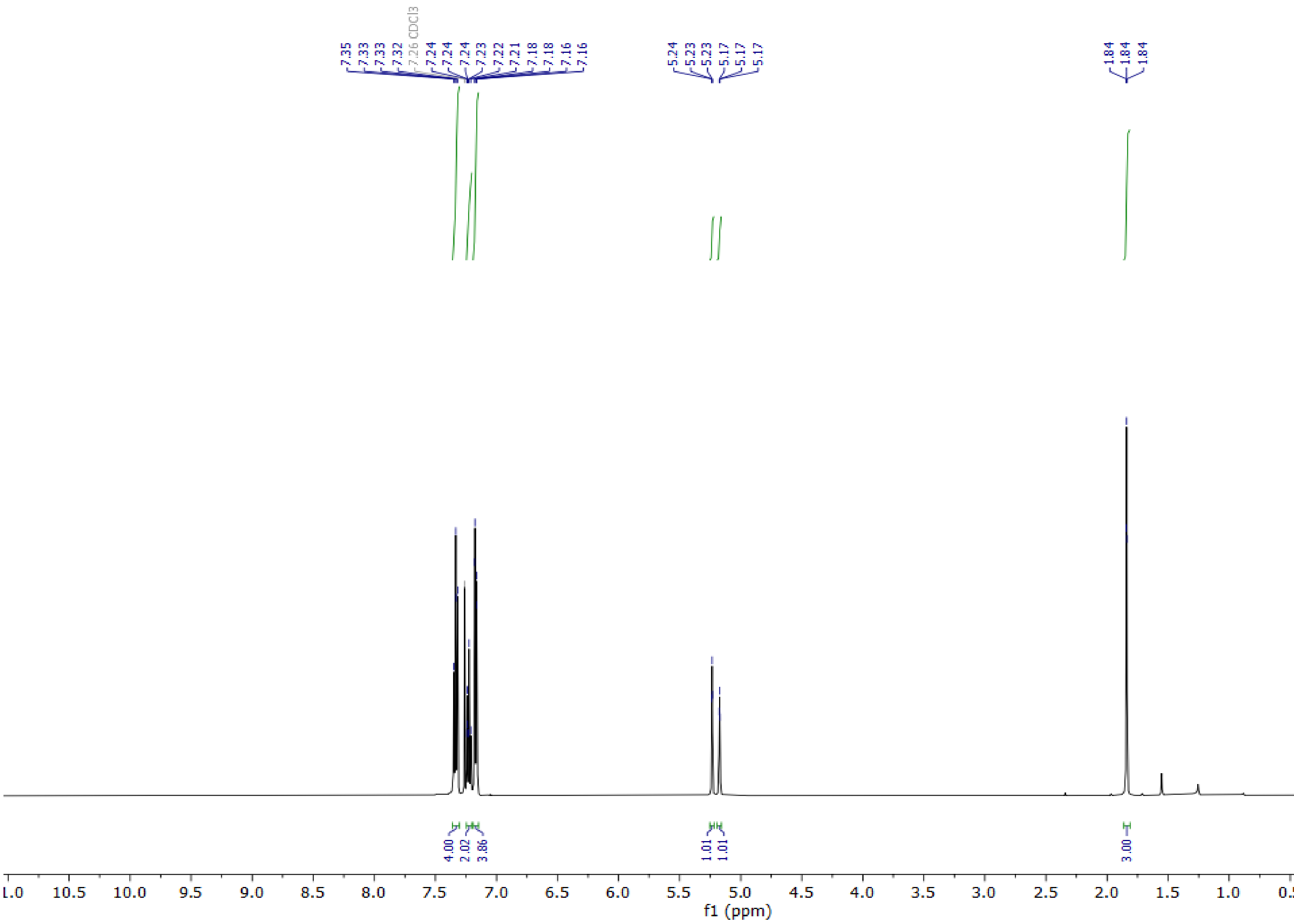

^{13}C NMR of *N,N*-diphenylmethacrylamide (7)

126 MHzCDCl$_3$, 298 K

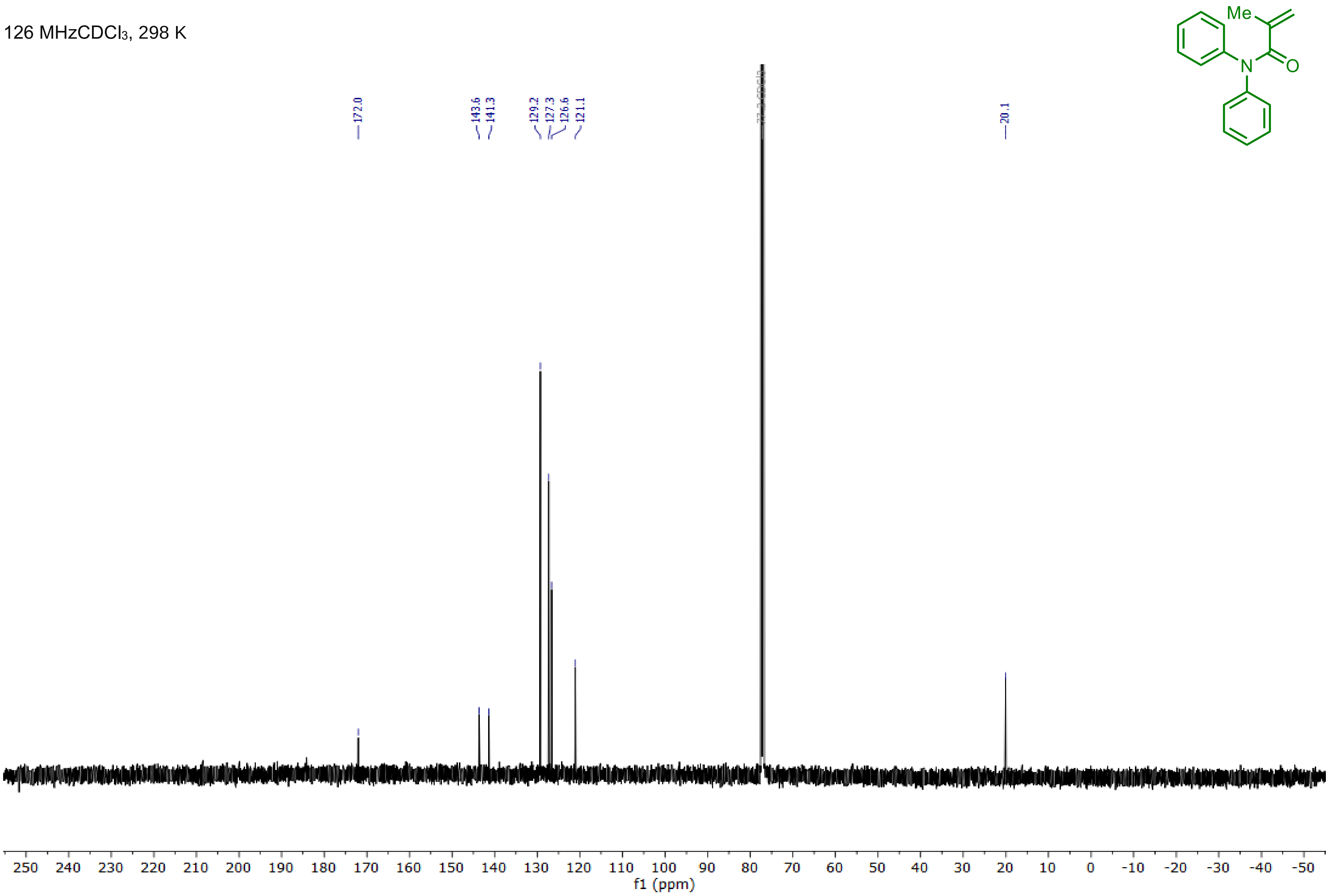

^{1}H NMR of 3-methyl-3-(((oxodiphenyl-λ[6]-sulfaneylidene)amino)methyl)-1-phenylindolin-2-one (8)

600 MHz, $CDCl_3$, 298 K

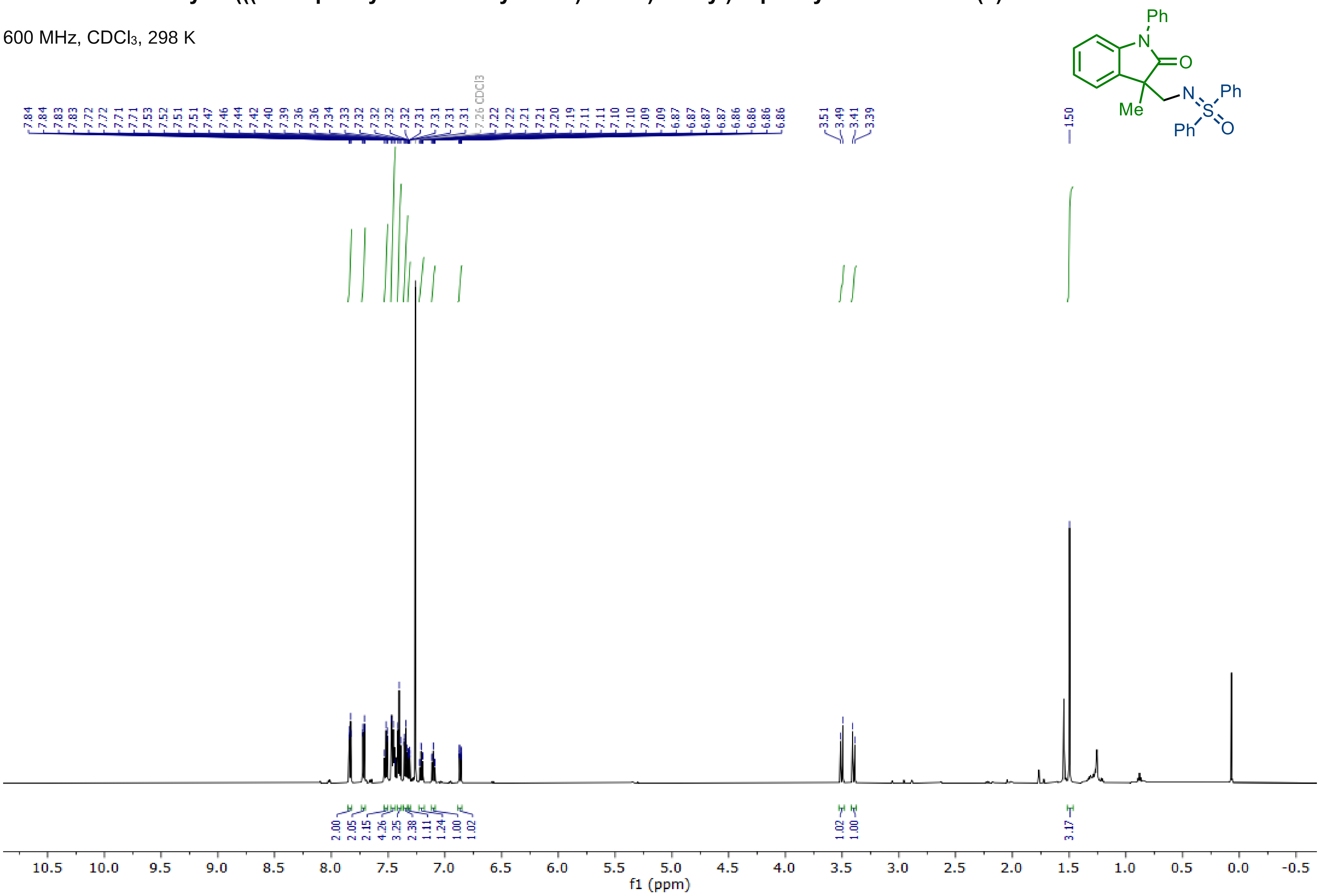

^{13}C NMR of 3-methyl-3-(((oxodiphenyl-λ^6-sulfaneylidene)amino)methyl)-1-phenylindolin-2-one (8)

151 MHz, $CDCl_3$, 298 K

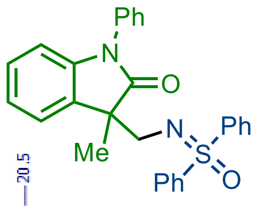

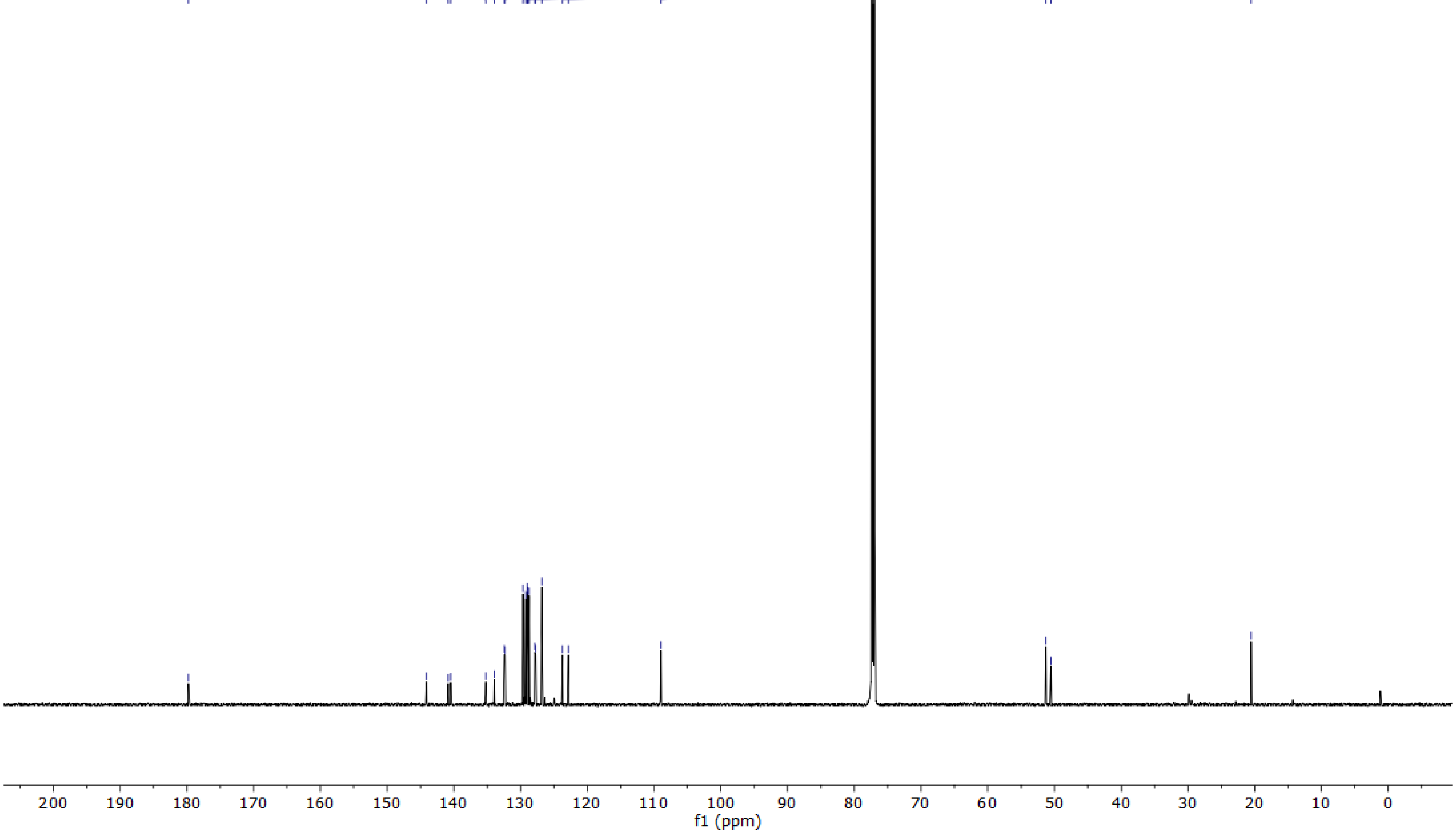

^{1}H NMR of sulfoximinyl containing I(III) reagent **9**

500 MHz, $(CD_3)_2SO$, 298 K

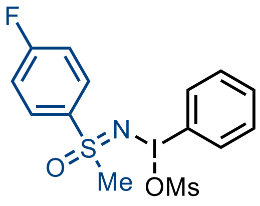

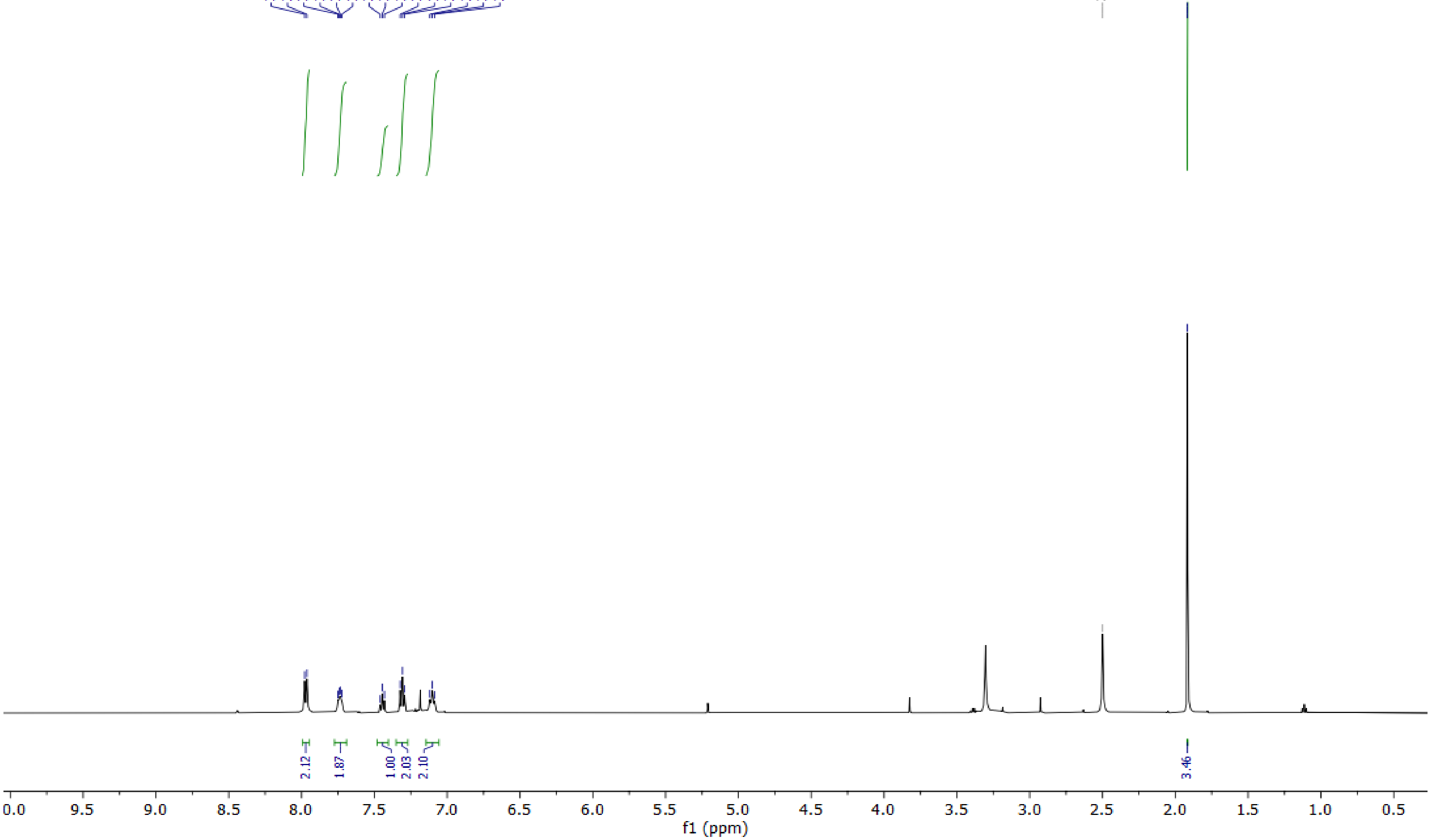

^{19}F NMR of sulfoximinyl containing I(III) reagent 9

471 MHz, $(CD_3)_2SO$, 298 K

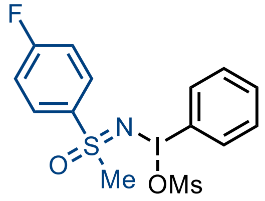

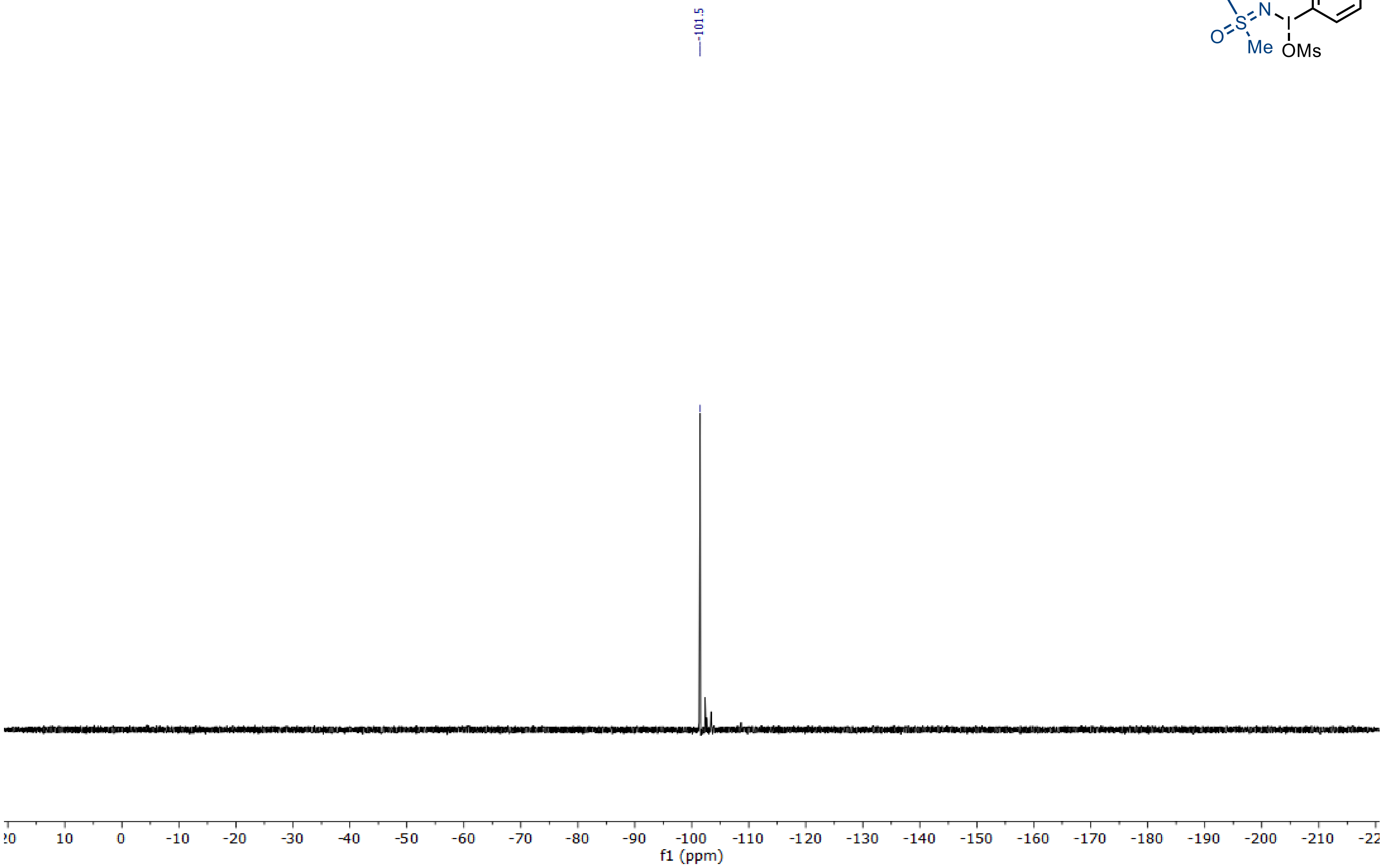

^{1}H NMR of (4-fluorophenyl)(methyl)(phenylimino)-λ^6-sulfanone (3)

500 MHz, $CDCl_3$, 298 K

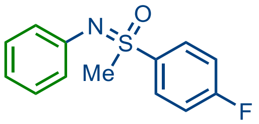

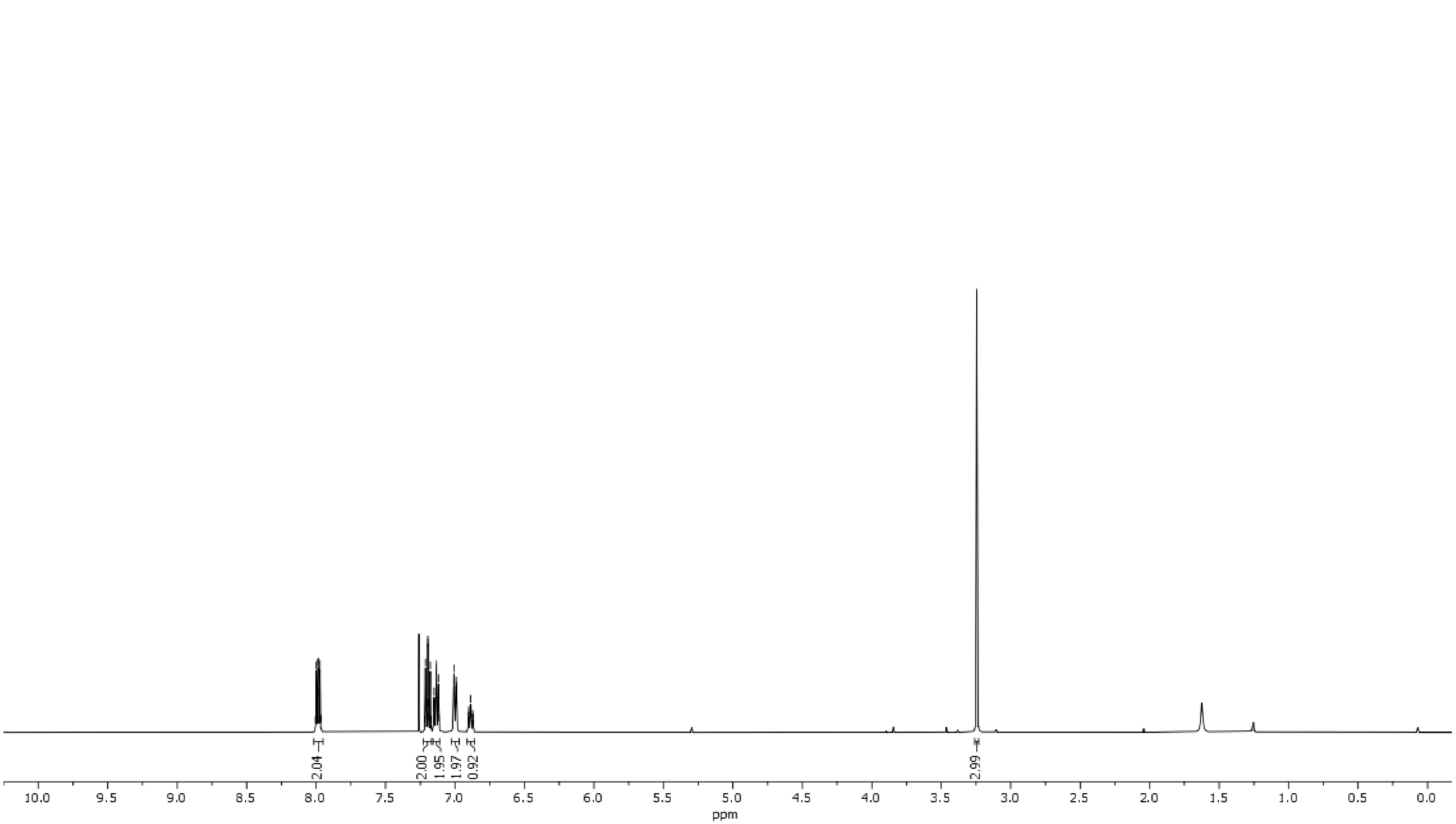

^{13}C NMR of (4-fluorophenyl)(methyl)(phenylimino)-λ^6-sulfanone (3)

126 MHz, $CDCl_3$, 298 K

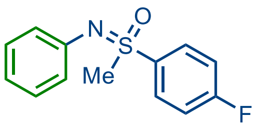

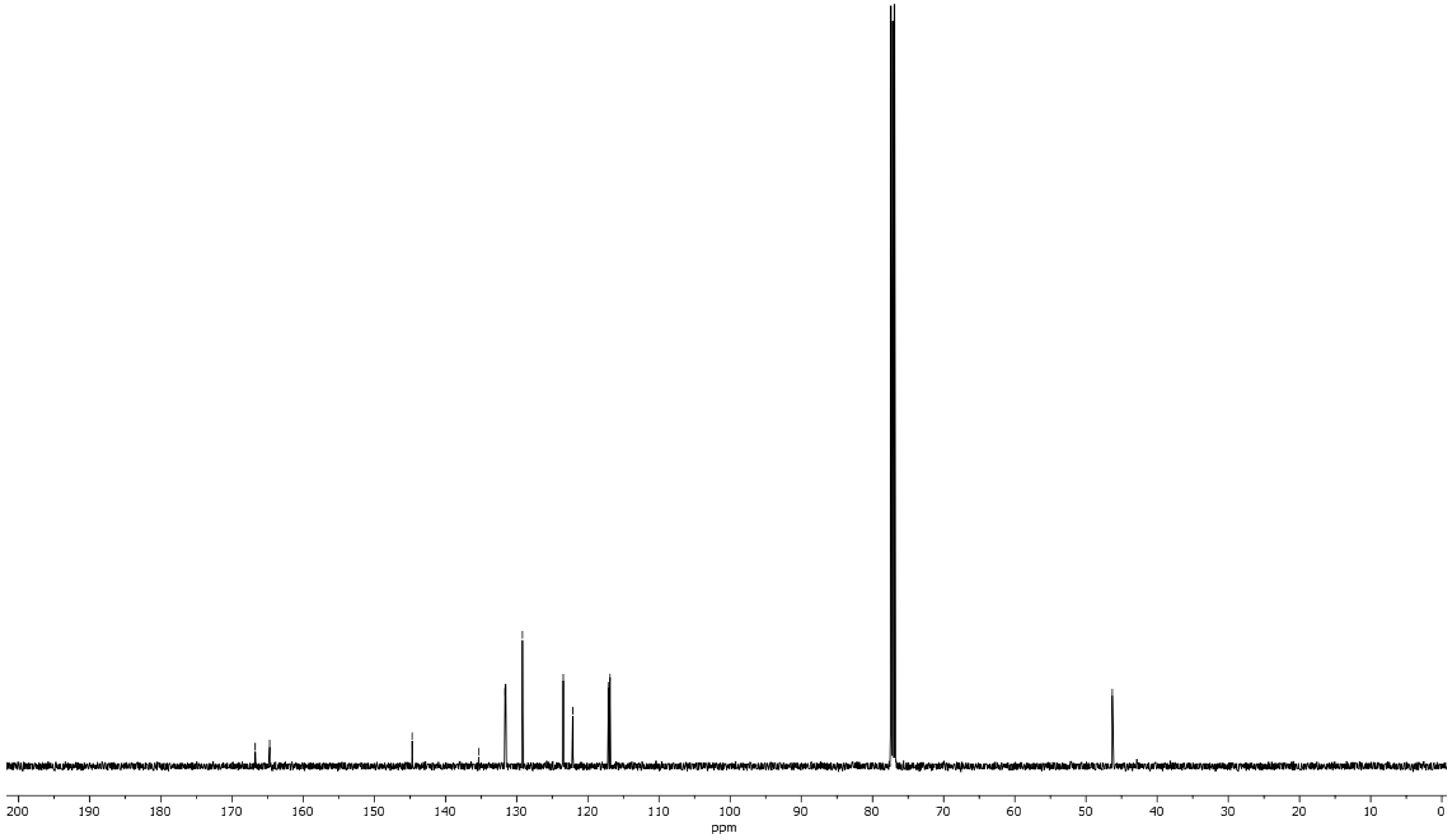

^{19}F NMR of (4-fluorophenyl)(methyl)(phenylimino)-λ^6-sulfanone (3)

471 MHz, $CDCl_3$, 298 K

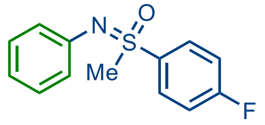

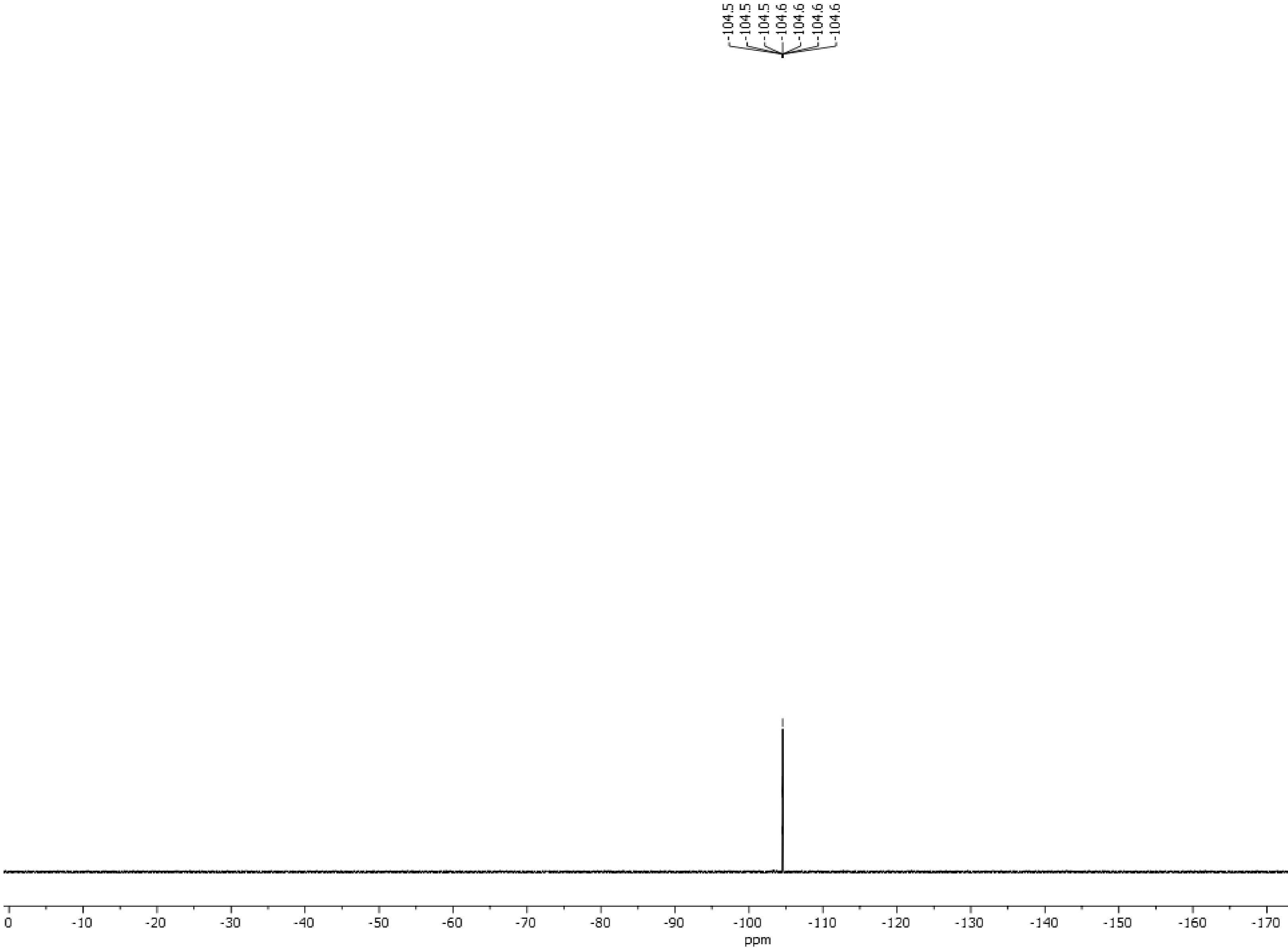

^{1}H NMR of ((2,5-dichlorophenyl)imino)(4-fluorophenyl)(methyl)-λ[6]-sulfanone (11)

500 MHz, $CDCl_3$, 298 K

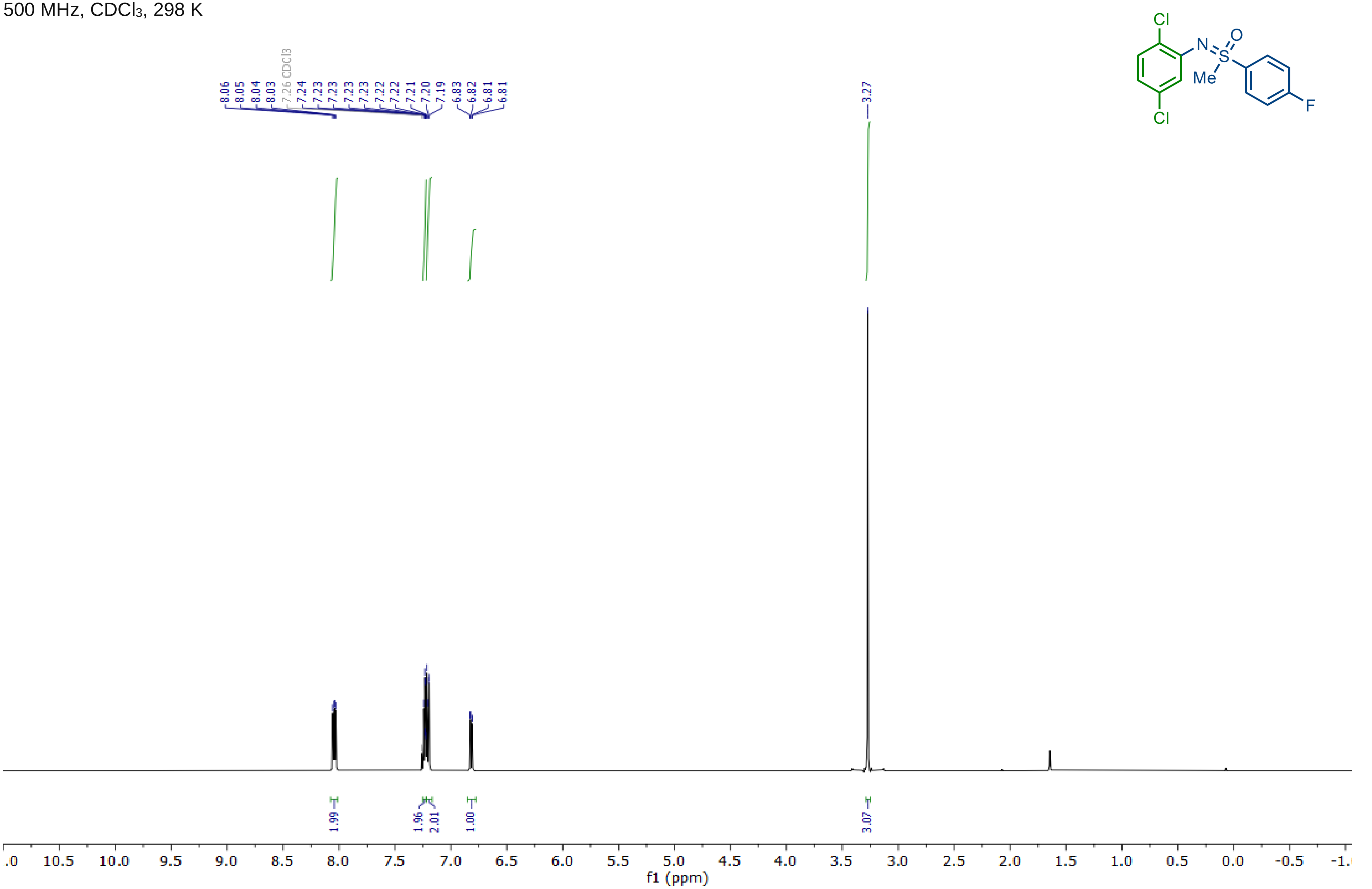

^{13}C NMR of ((2,5-dichlorophenyl)imino)(4-fluorophenyl)(methyl)-λ^6-sulfanone (11)

126 MHz, $CDCl_3$, 298 K

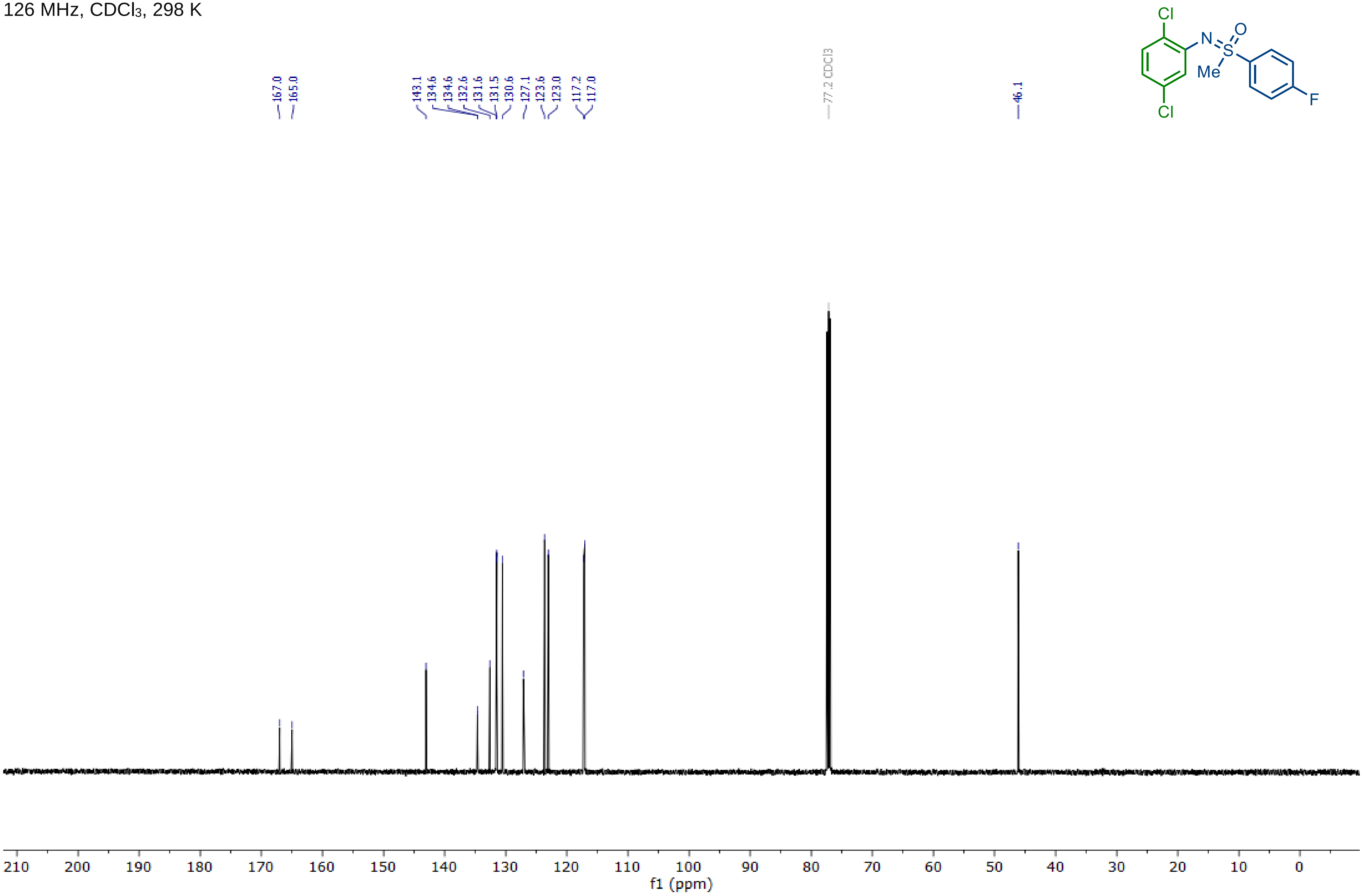

^{19}F NMR of ((2,5-dichlorophenyl)imino)(4-fluorophenyl)(methyl)-λ^6-sulfanone (11)

471 MHz, $CDCl_3$, 298 K

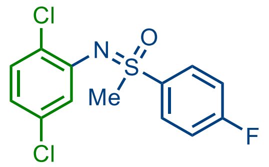

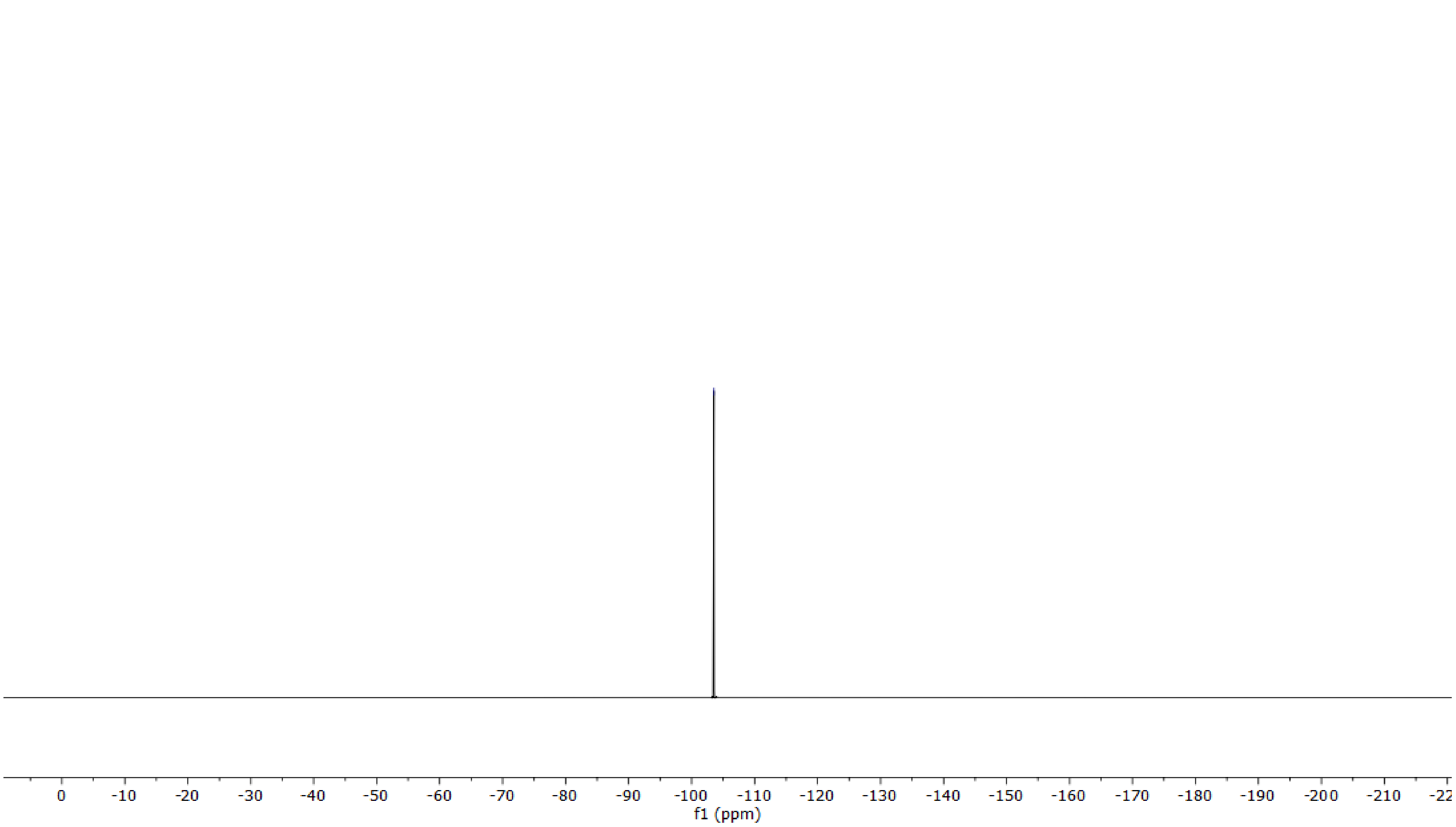

^{1}H NMR of ((2-bromo-5-(trifluoromethoxy)phenyl)imino)(4-fluorophenyl)(methyl)-λ[6]-sulfanone (12-C1)

500 MHz, $CDCl_3$, 298 K

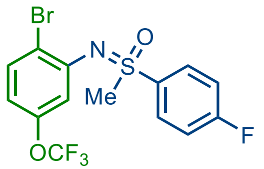

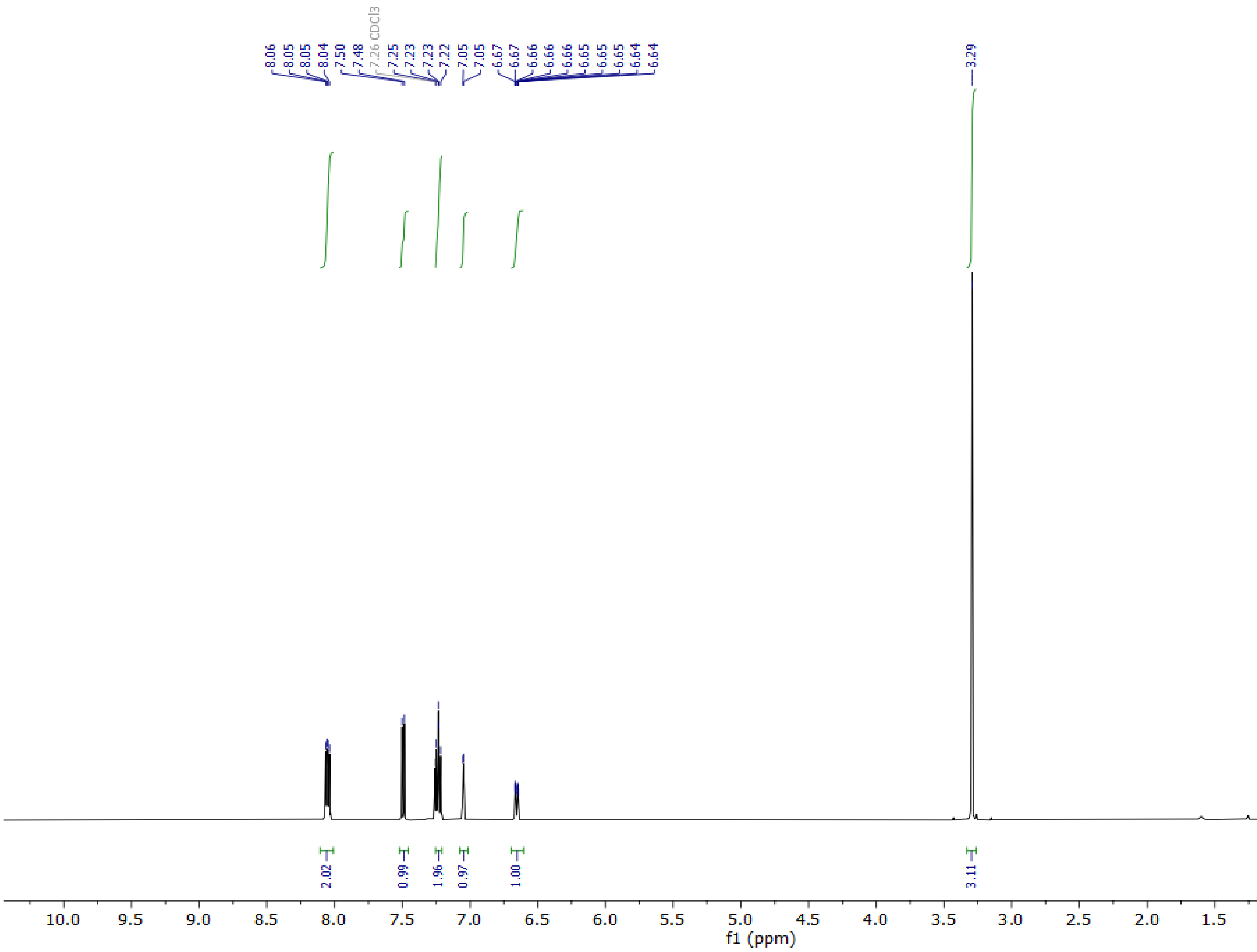

^{13}C NMR of ((2-bromo-5-(trifluoromethoxy)phenyl)imino)(4-fluorophenyl)(methyl)-λ^6-sulfanone (12-C1)

126 MHz, $CDCl_3$, 298 K

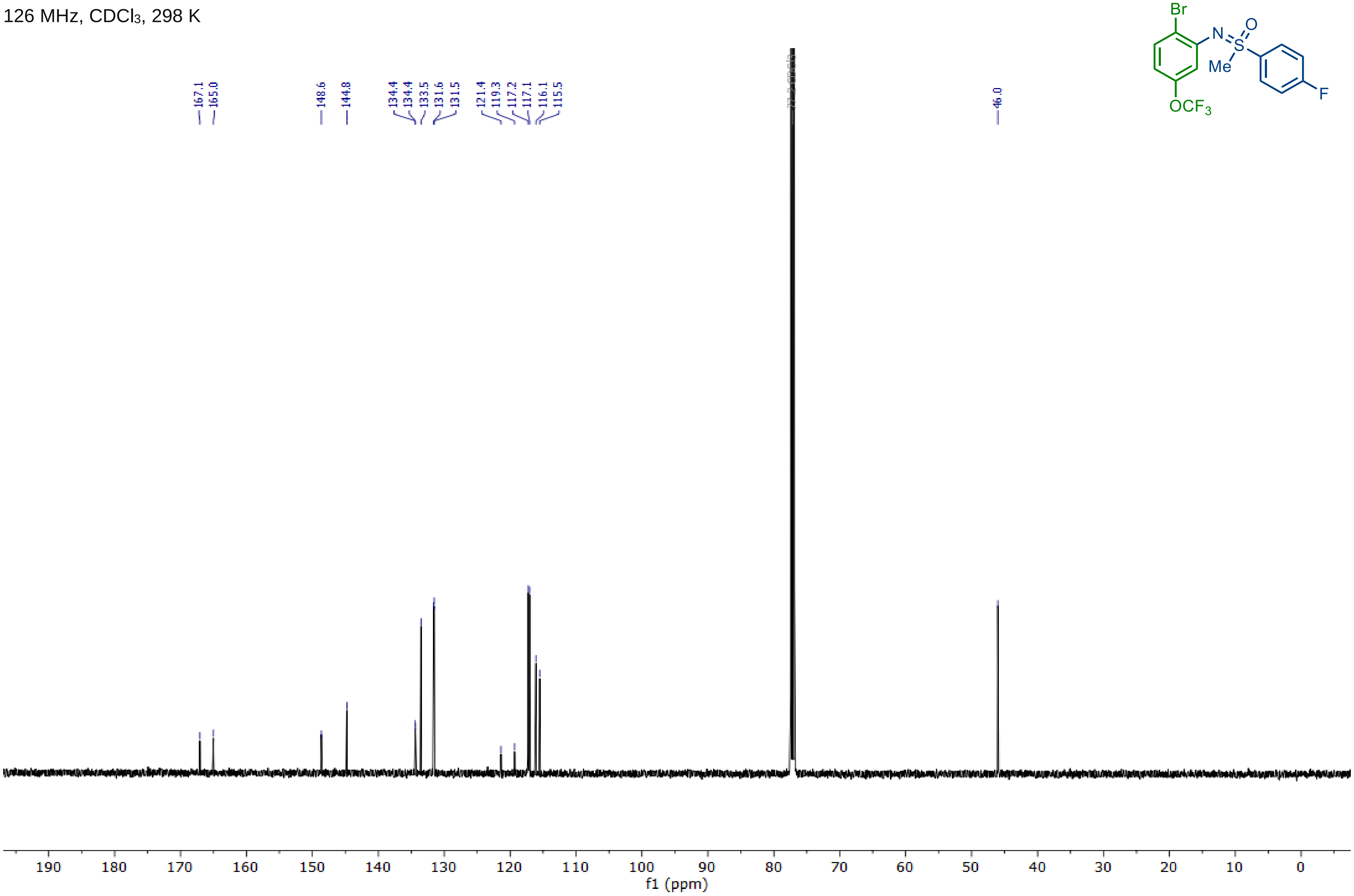

^{19}F NMR of ((2-bromo-5-(trifluoromethoxy)phenyl)imino)(4-fluorophenyl)(methyl)-λ^6-sulfanone (12-C1)

471 MHz, $CDCl_3$, 298 K

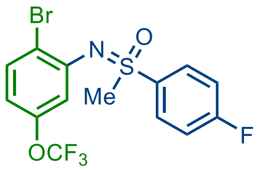

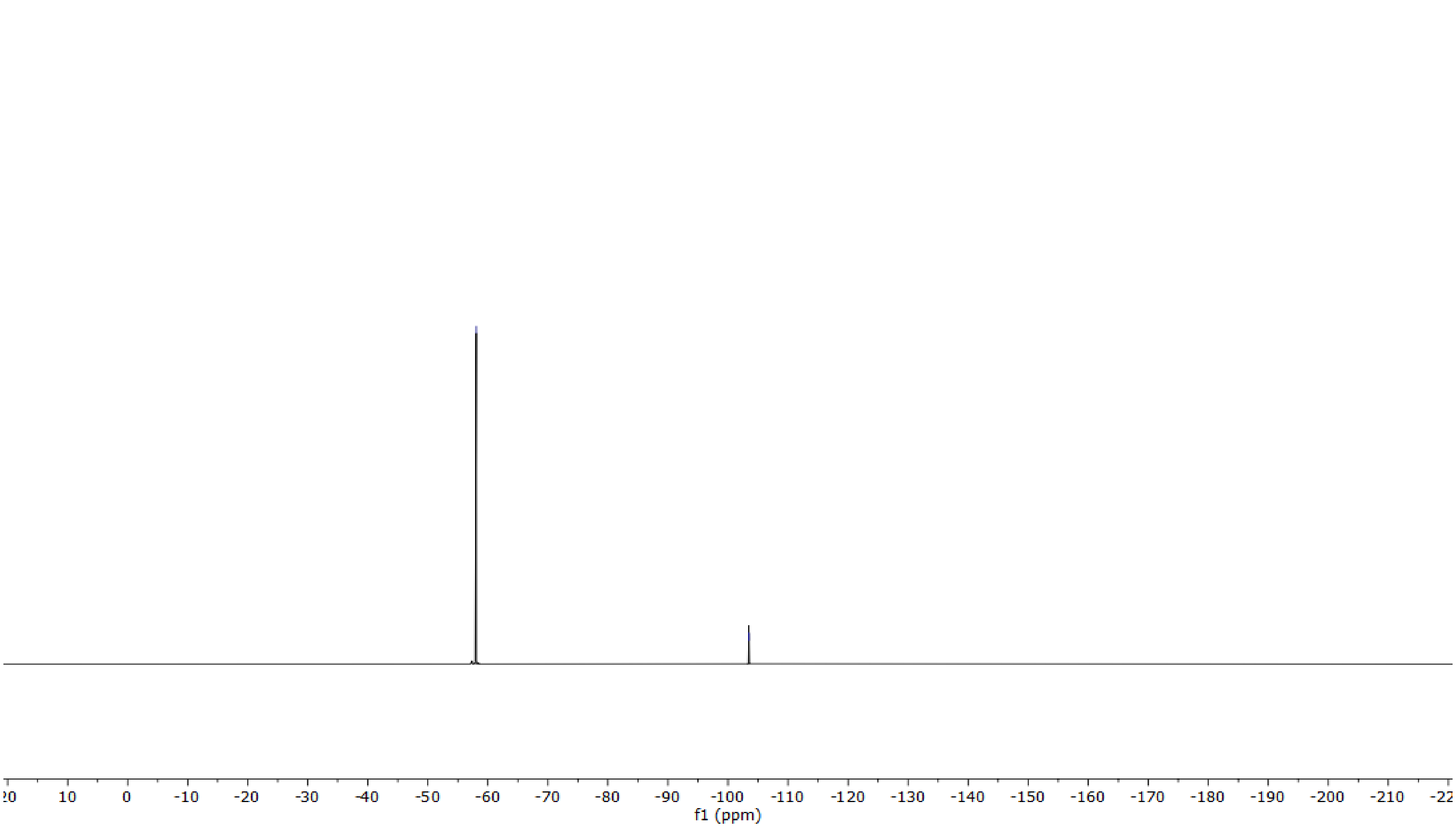

^{1}H NMR of ((5-bromo-2-(trifluoromethoxy)phenyl)imino)(4-fluorophenyl)(methyl)-λ^{6}-sulfanone (12-C2)

600 MHz, $CDCl_3$, 298 K

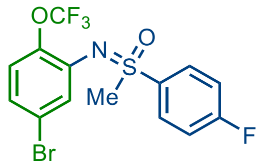

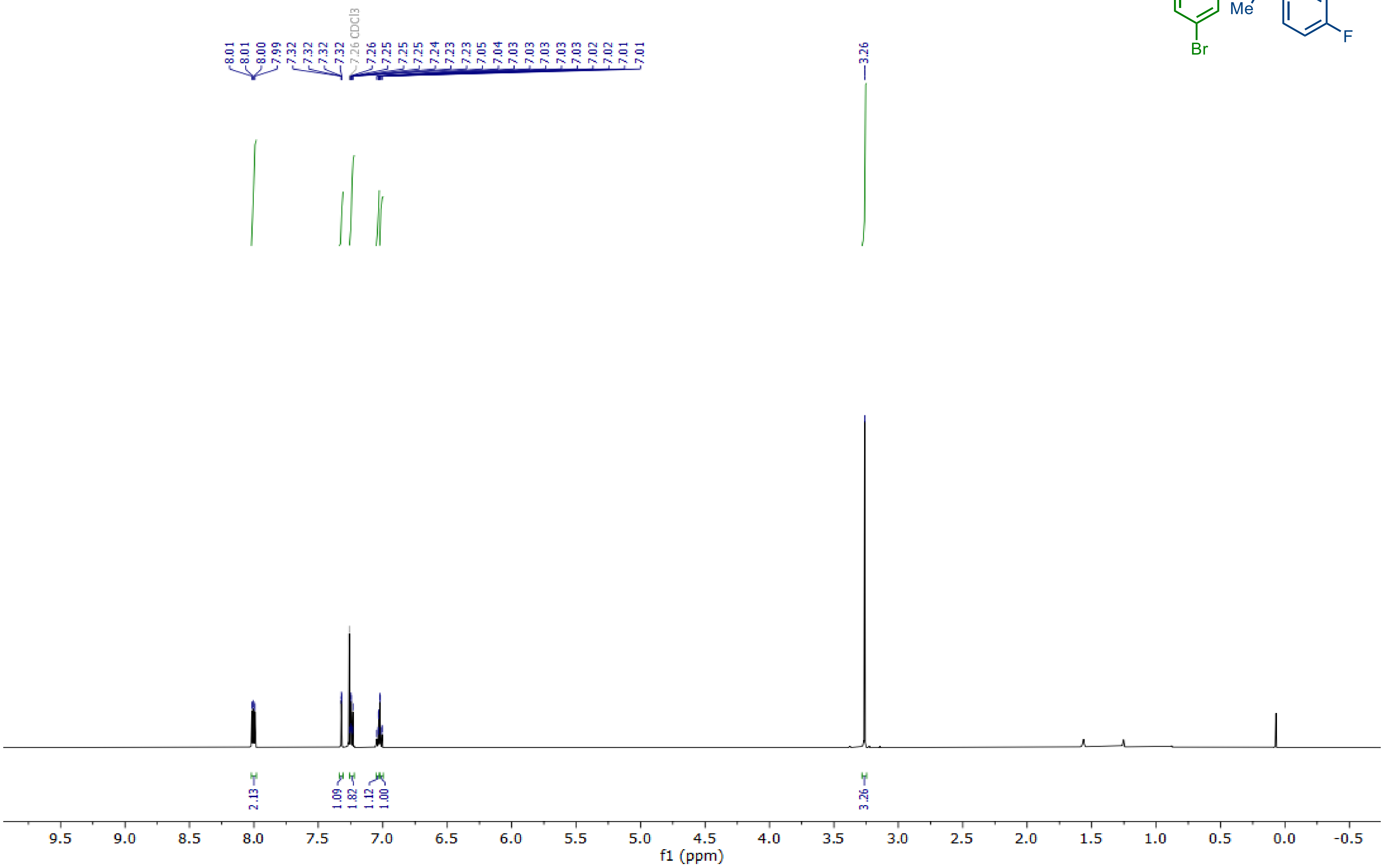

^{13}C NMR of ((5-bromo-2-(trifluoromethoxy)phenyl)imino)(4-fluorophenyl)(methyl)-λ^6-sulfanone (12-C2)

151 MHz, $CDCl_3$, 298 K

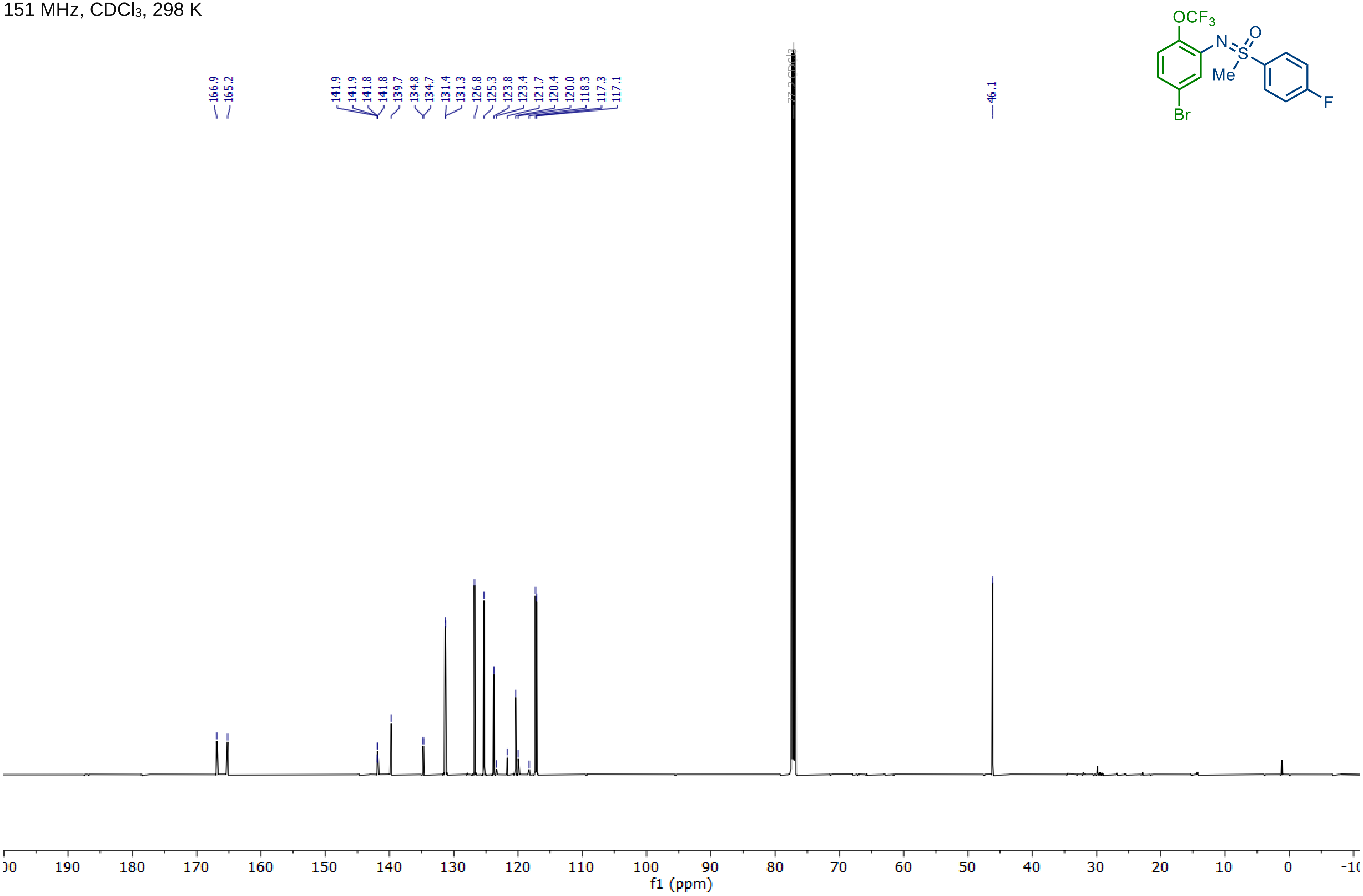

^{19}F NMR of ((5-bromo-2-(trifluoromethoxy)phenyl)imino)(4-fluorophenyl)(methyl)-λ^6-sulfanone (12-C2)

565 MHz, $CDCl_3$, 298 K

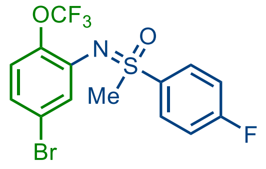

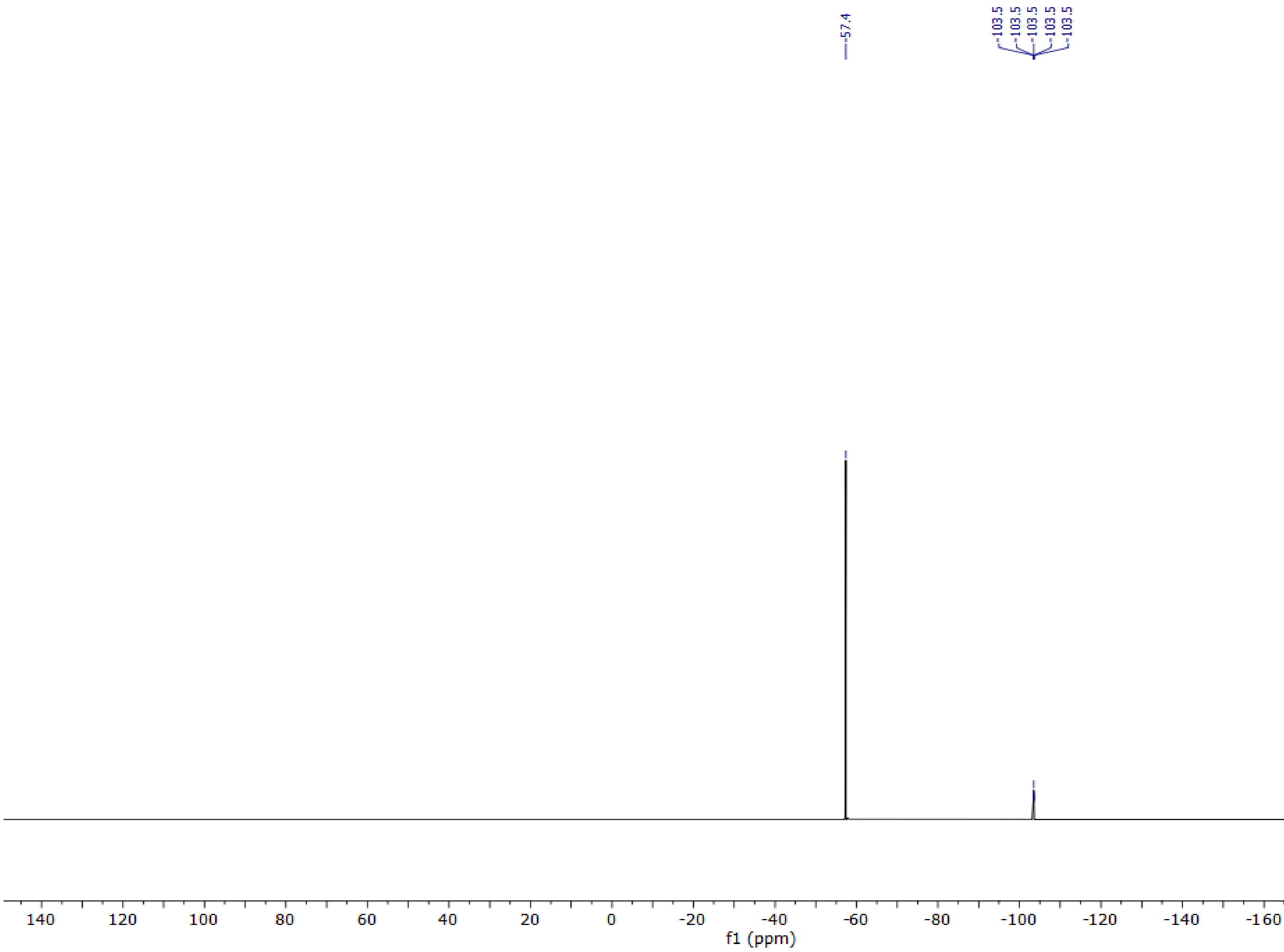

^{1}H NMR of ((tert-butyl)phenyl)imino)(4-fluorophenyl)(methyl)-λ[6]-sulfanone (13)

600 MHz, $CDCl_3$, 298 K

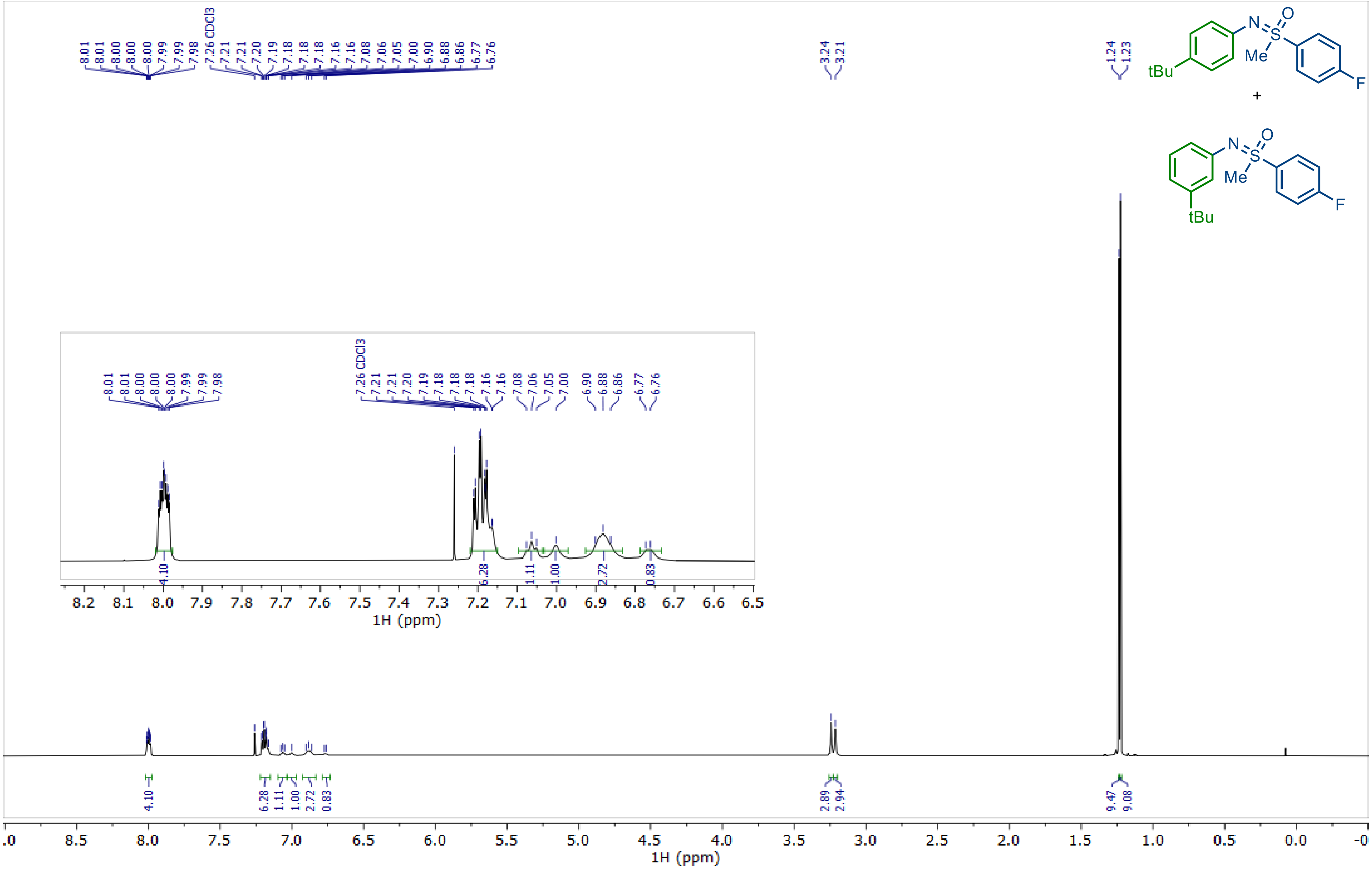

^{13}C NMR of ((tert-butyl)phenyl)imino)(4-fluorophenyl)(methyl)-λ^6-sulfanone (13)

151 MHz, $CDCl_3$, 298 K

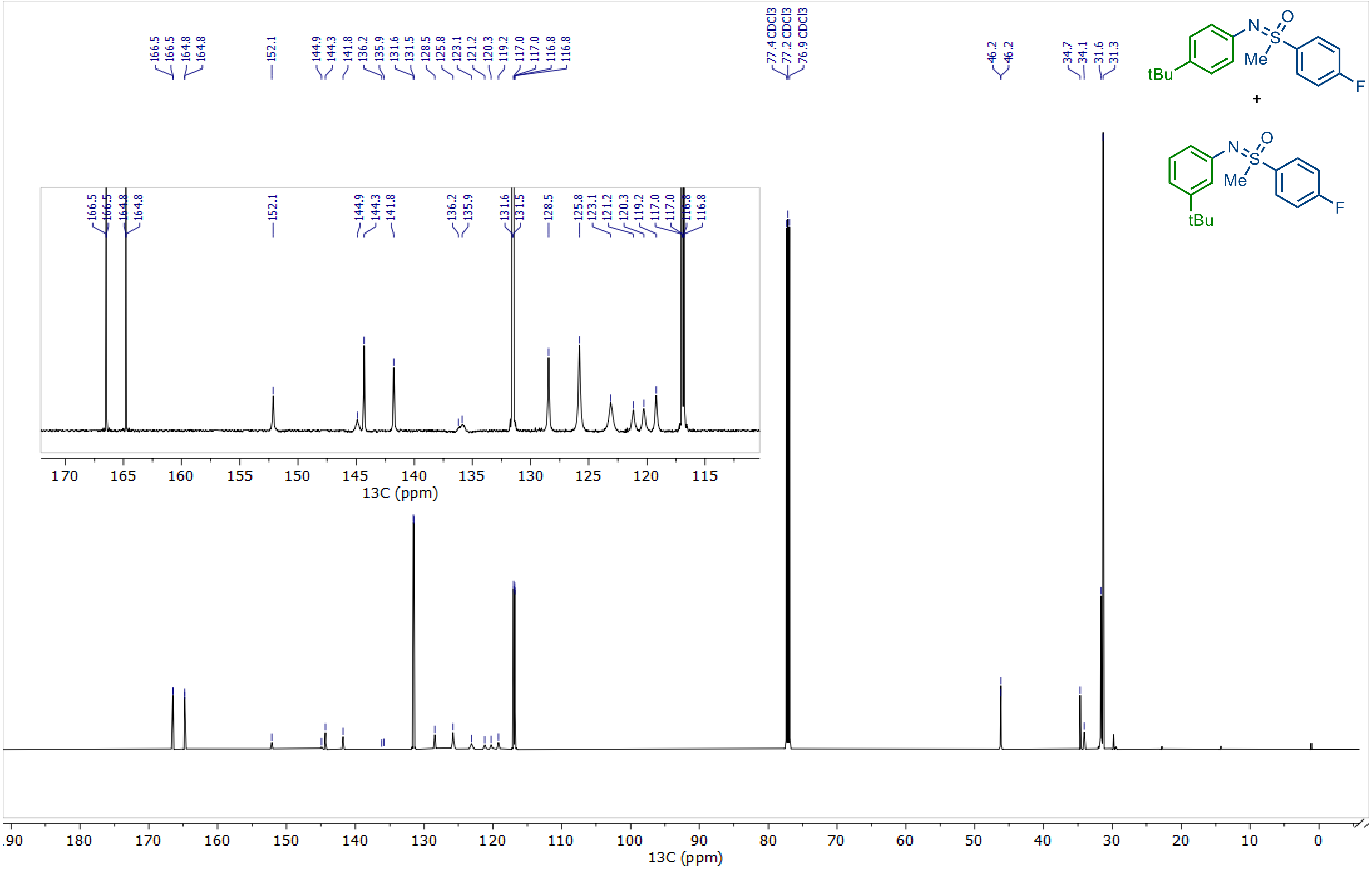

^{19}F NMR of ((tert-butyl)phenyl)imino)(4-fluorophenyl)(methyl)-λ^{6}-sulfanone (13)

565 MHz, $CDCl_3$, 298 K

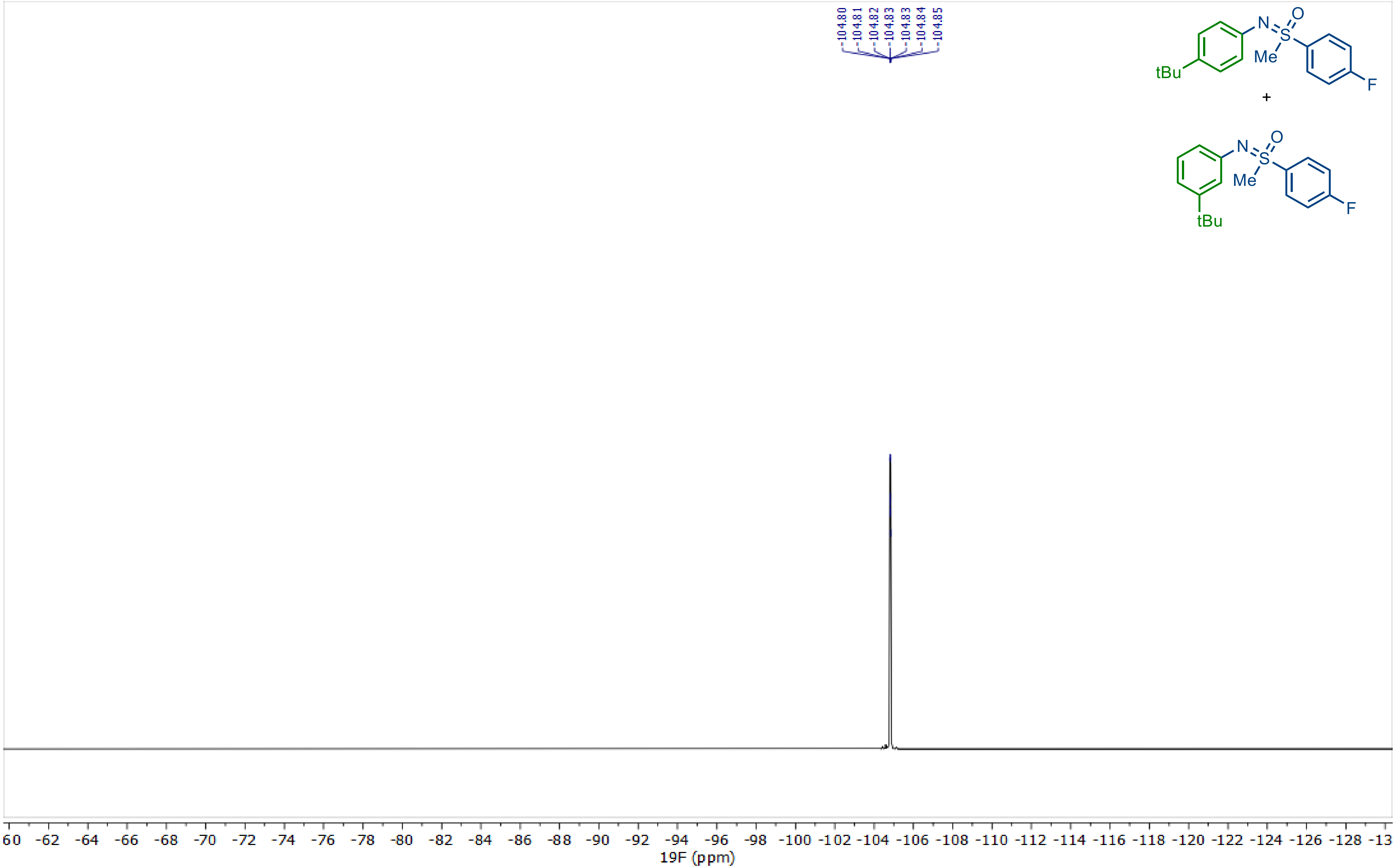

^{1}H NMR of 4-(((4-fluorophenyl)(methyl)(oxo)-λ^6-sulfaneylidene)amino)phenyl trifluoromethanesulfonate (14-C1)

500 MHz, $CDCl_3$, 298 K

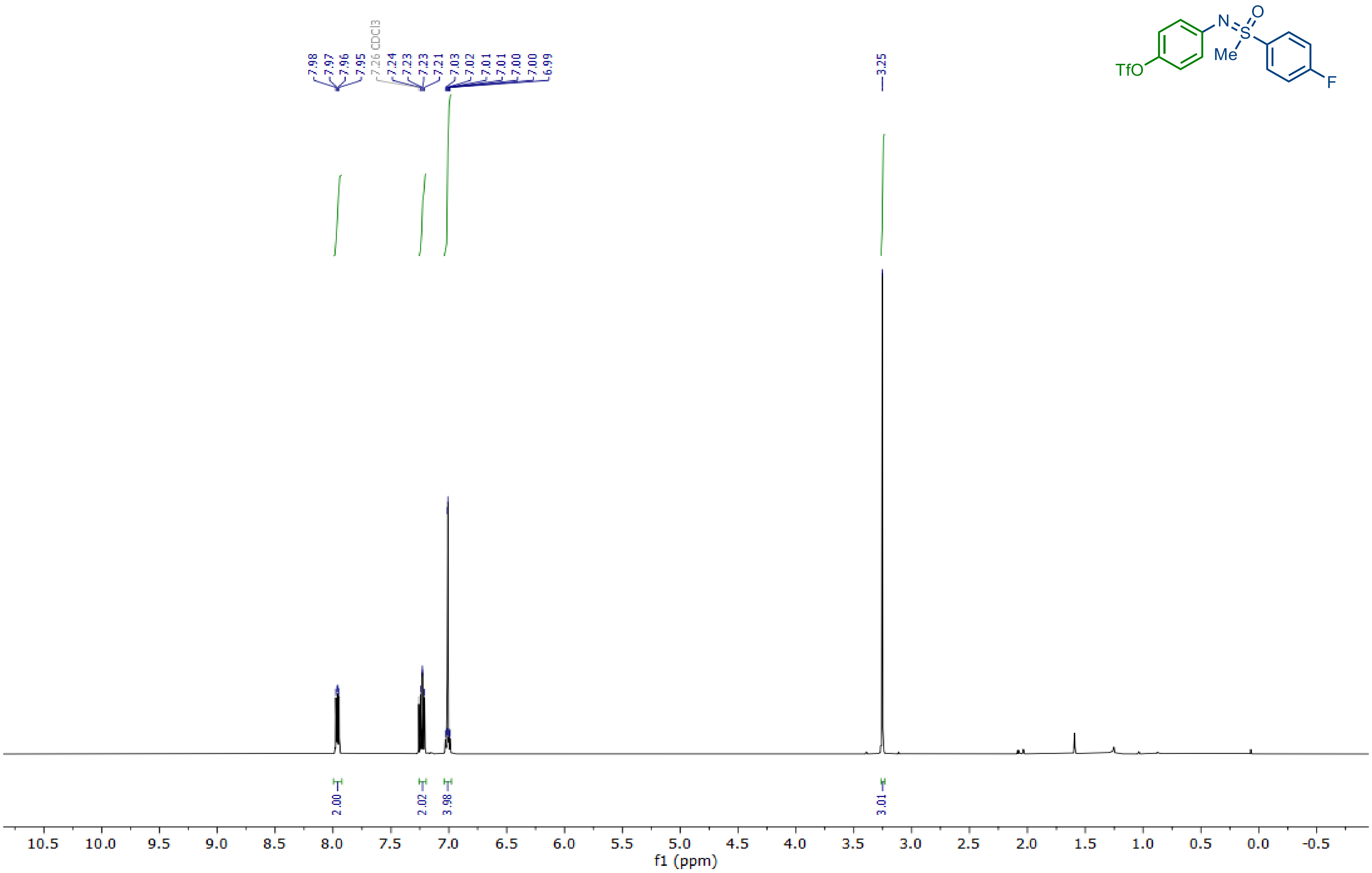

^{13}C NMR of 4-(((4-fluorophenyl)(methyl)(oxo)-λ^6-sulfaneylidene)amino)phenyl trifluoromethanesulfonate (14-C1)

126 MHz, $CDCl_3$, 298 K

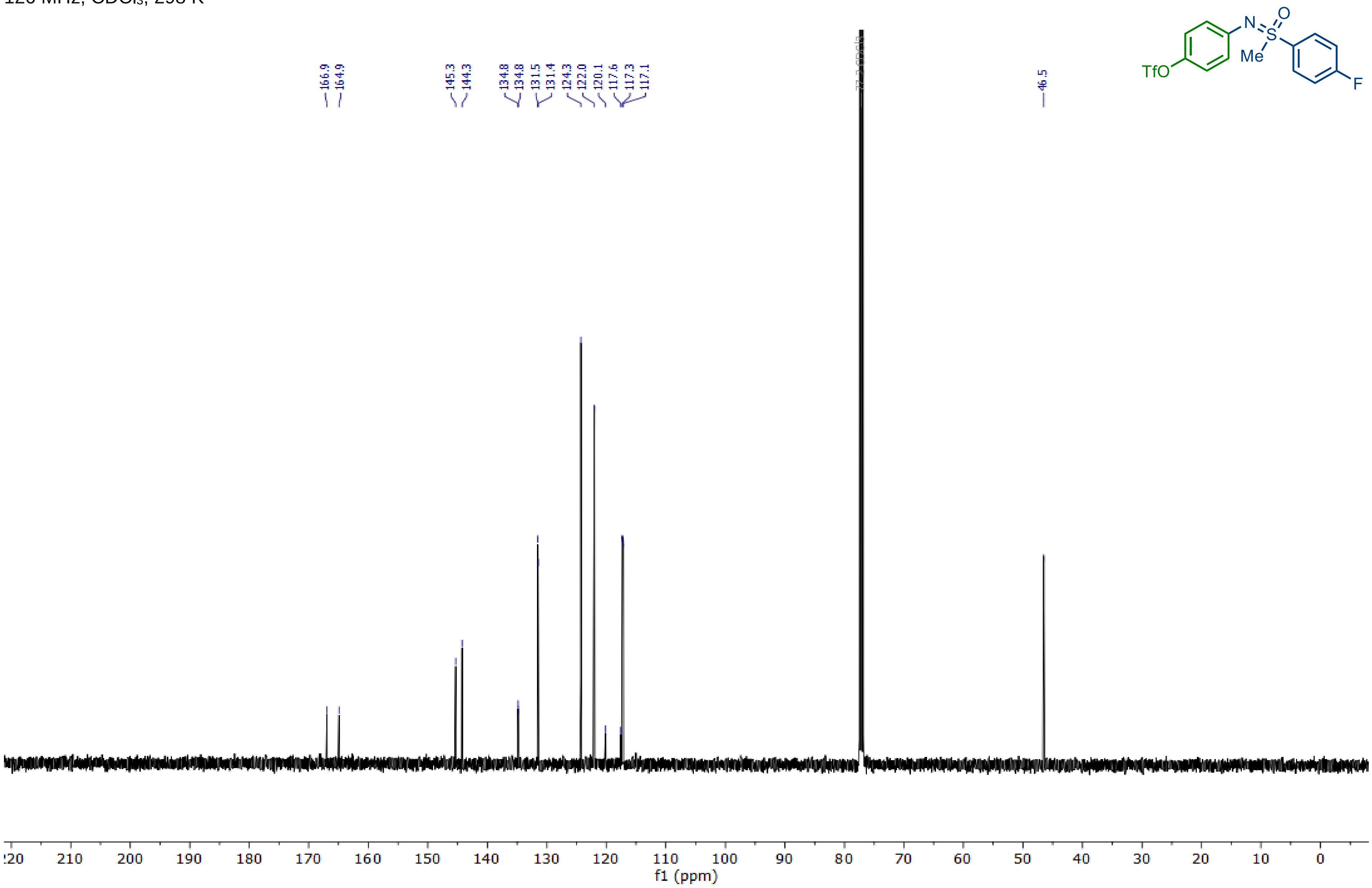

^{19}F NMR of 4-(((4-fluorophenyl)(methyl)(oxo)-λ^{6}-sulfaneylidene)amino)phenyl trifluoromethanesulfonate (14-C1)

471 MHz, $CDCl_3$, 298 K

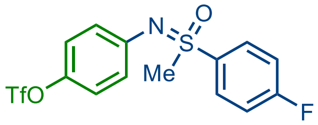

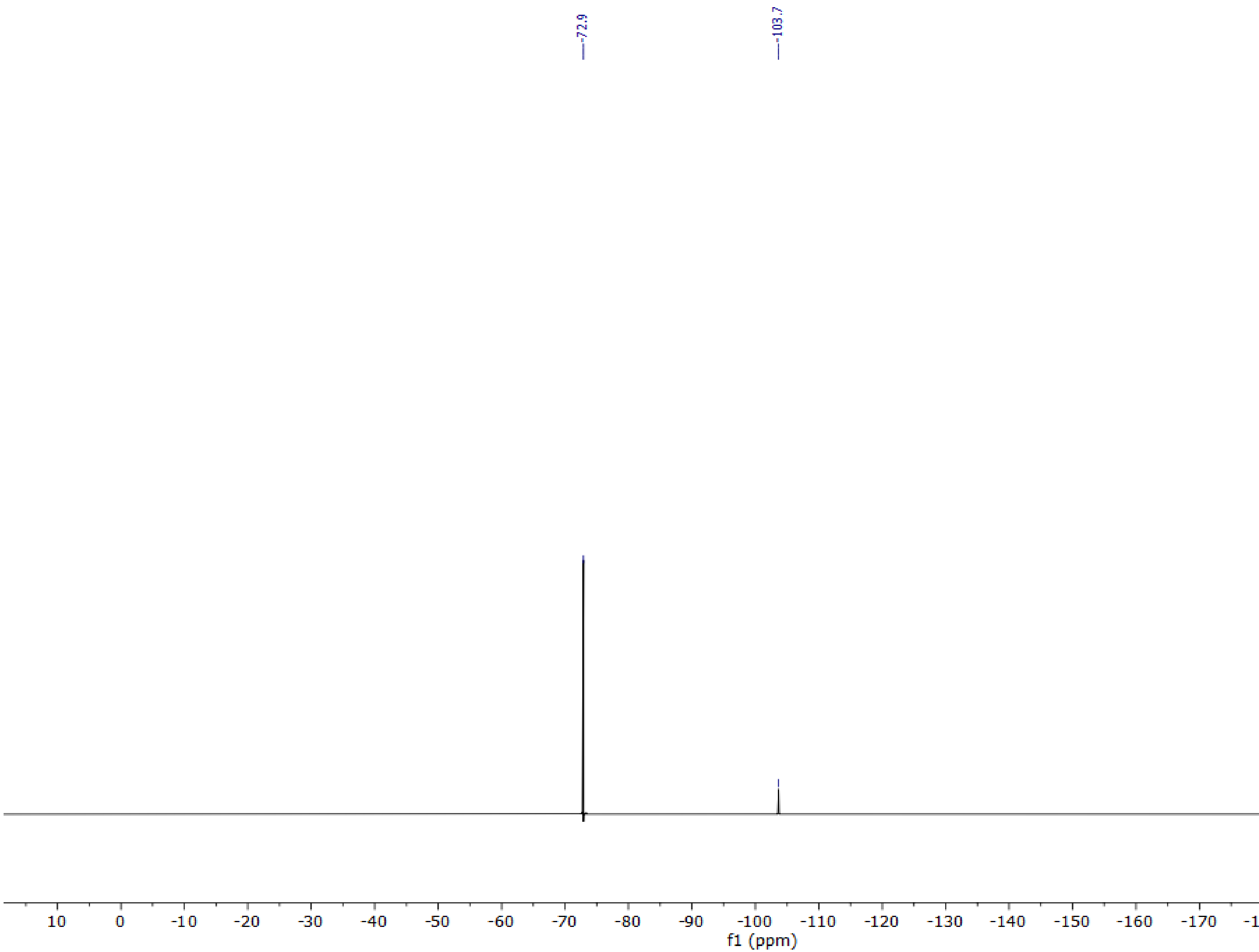

^{1}H NMR of 3-(((4-fluorophenyl)(methyl)(oxo)-λ^{6}-sulfaneylidene)amino)phenyl trifluoromethanesulfonate (14-C2)

500 MHz, $CDCl_3$, 298 K

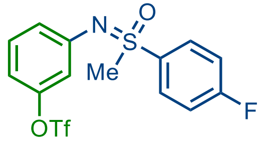

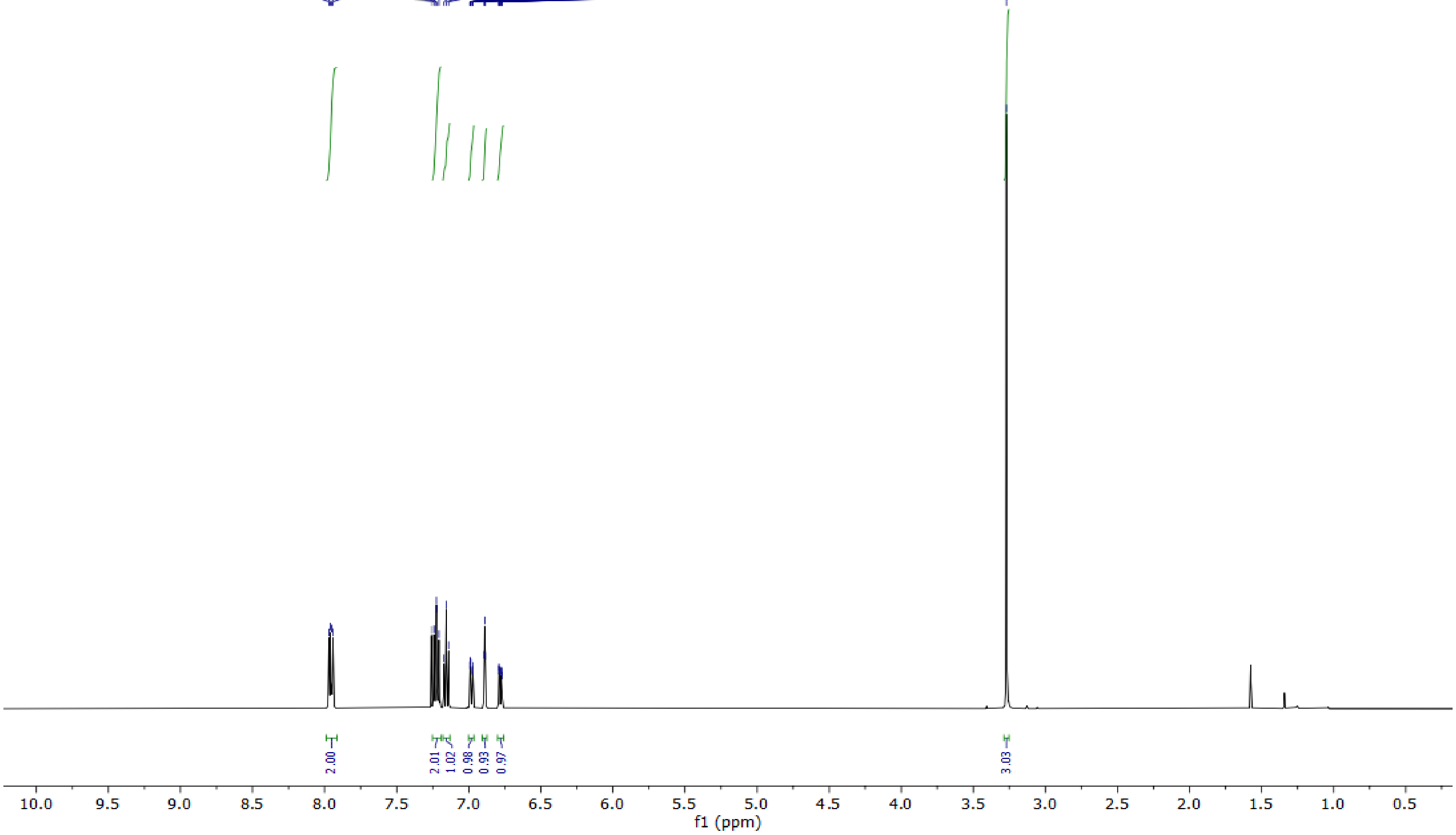

^{13}C NMR of 3-(((4-fluorophenyl)(methyl)(oxo)-λ^6-sulfaneylidene)amino)phenyl trifluoromethanesulfonate (14-C2)

126 MHz, $CDCl_3$, 298 K

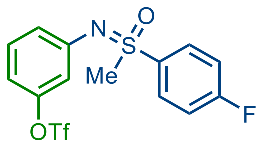

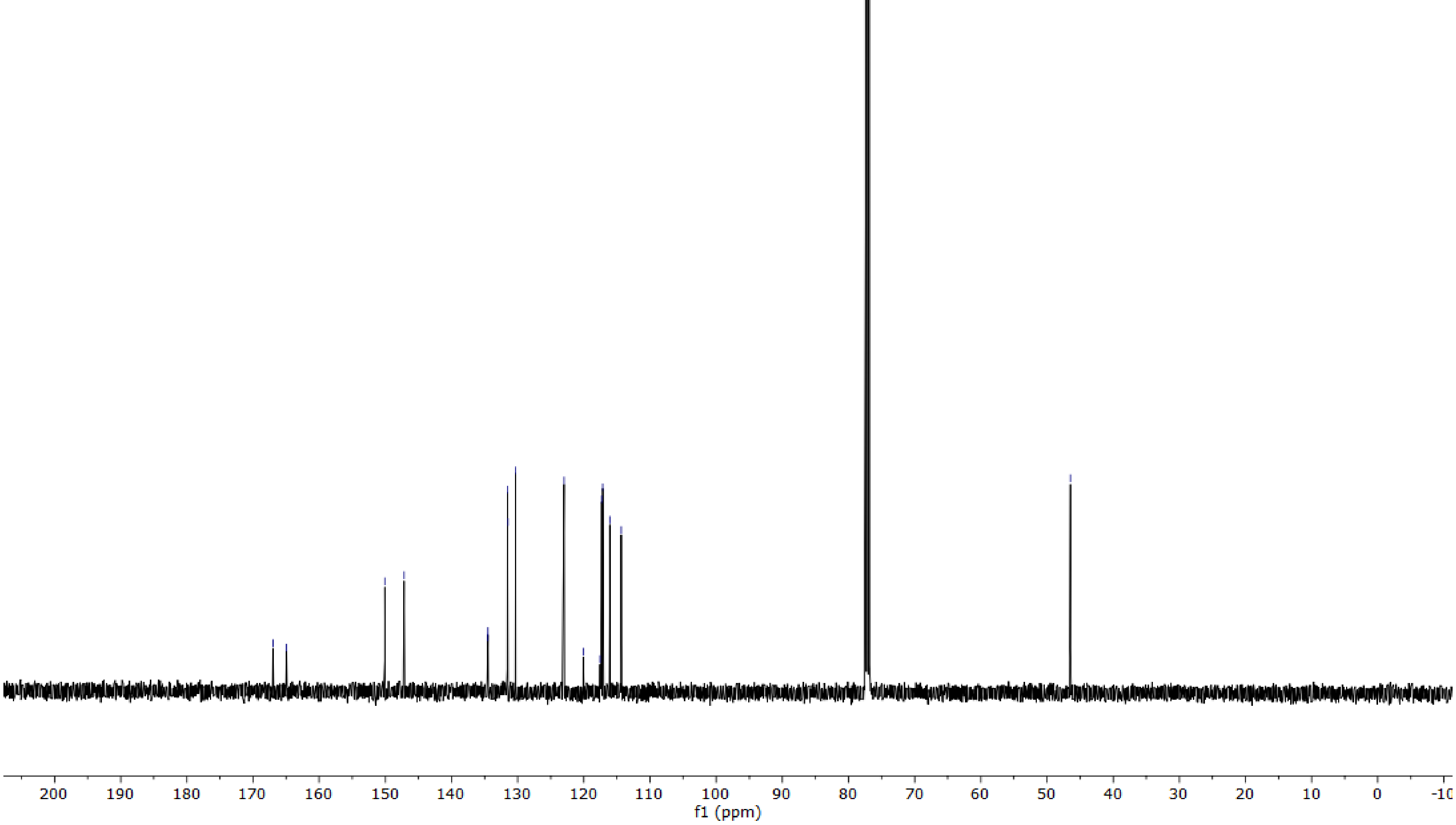

^{19}F NMR of 3-(((4-fluorophenyl)(methyl)(oxo)-λ^6-sulfaneylidene)amino)phenyl trifluoromethanesulfonate (14-C2)

471 MHz, $CDCl_3$, 298 K

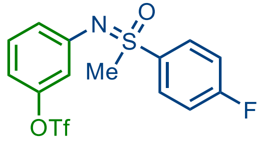

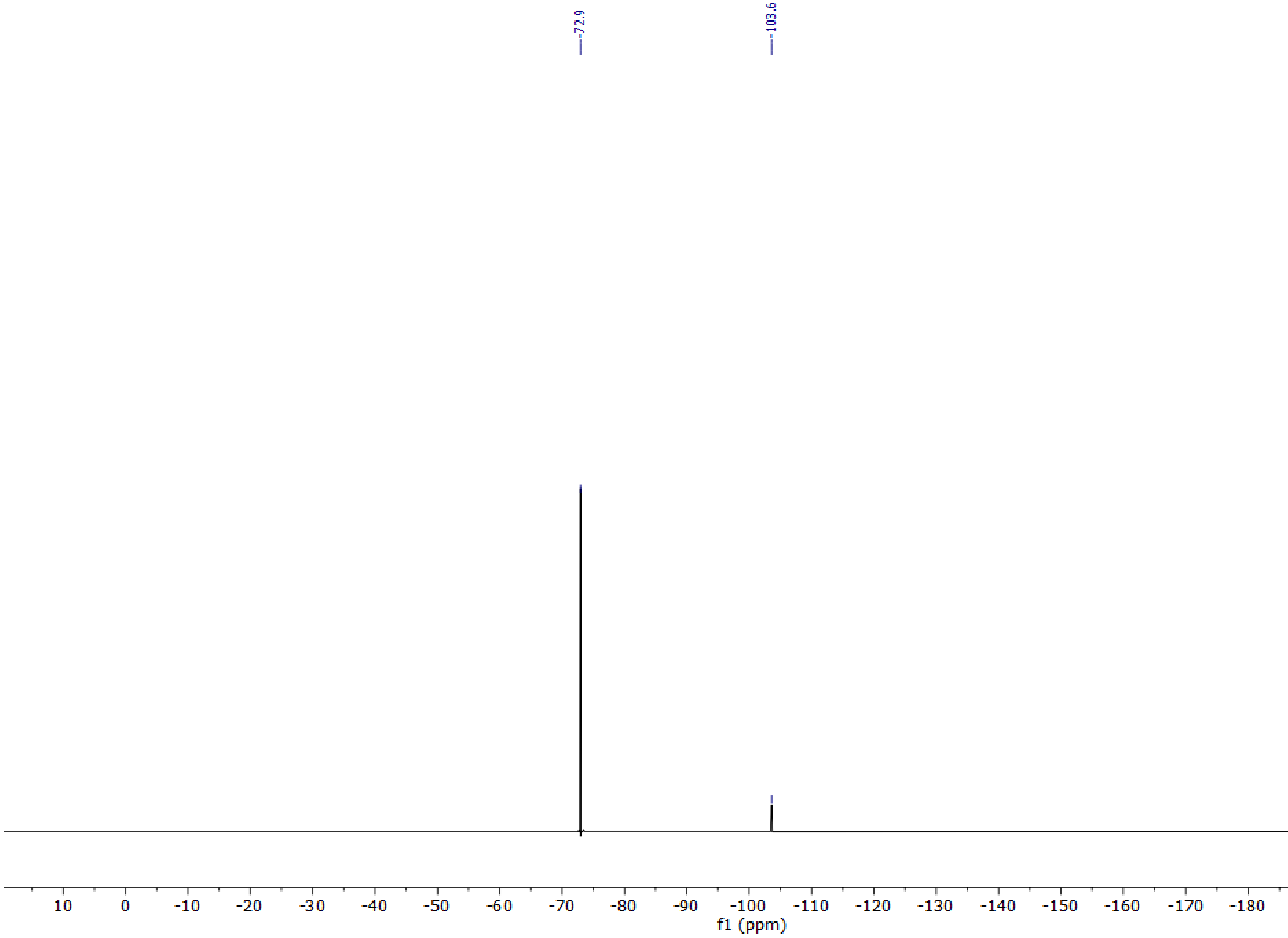

^{1}H NMR of 2-(((4-fluorophenyl)(methyl)(oxo)-λ^{6}-sulfaneylidene)amino)phenyl trifluoromethanesulfonate (14-C3)

500 MHz, $CDCl_3$, 298 K

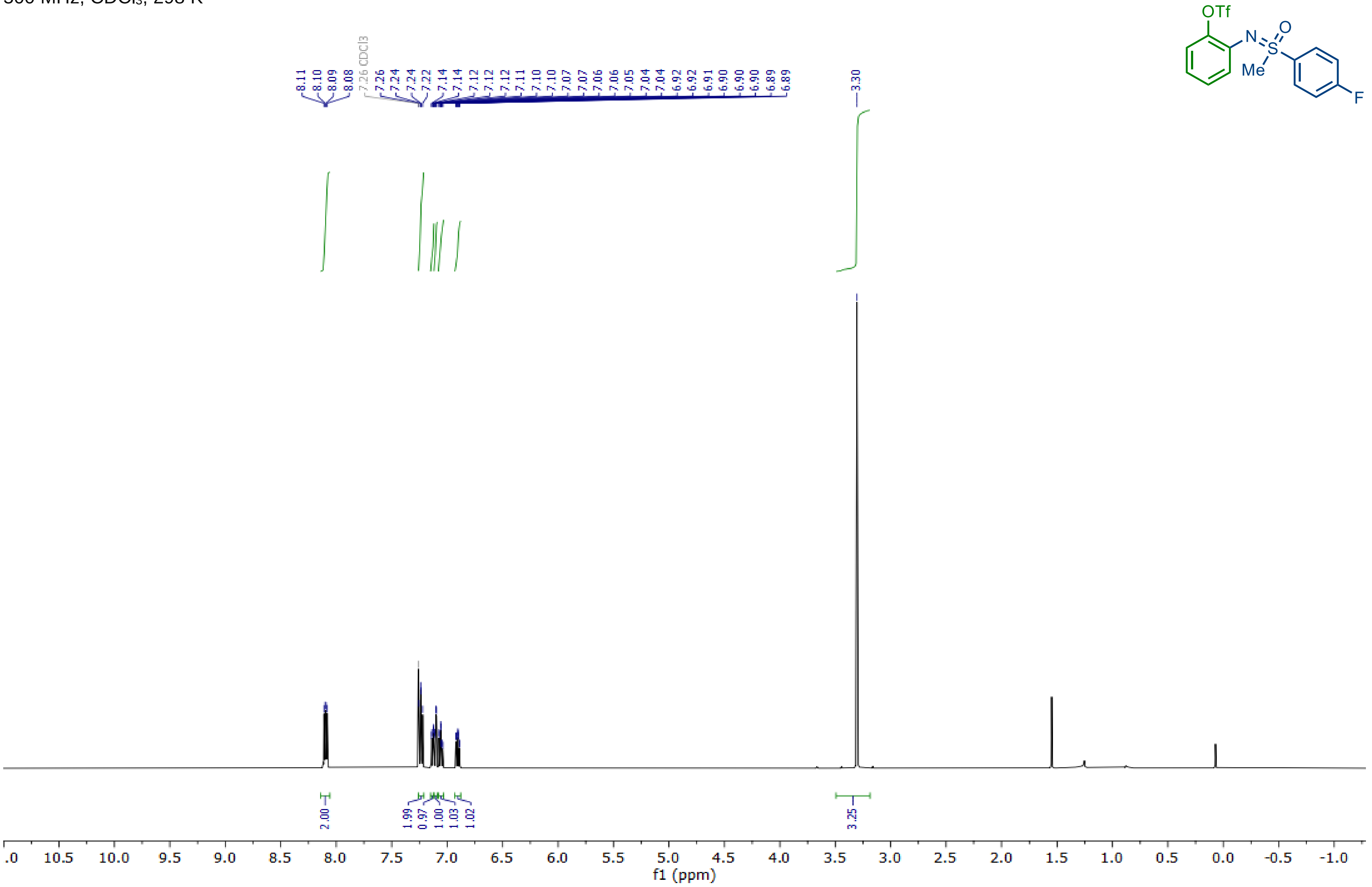

^{13}C NMR of 2-(((4-fluorophenyl)(methyl)(oxo)-λ^{6}-sulfaneylidene)amino)phenyl trifluoromethanesulfonate (14-C3)

126 MHz, $CDCl_3$, 298 K

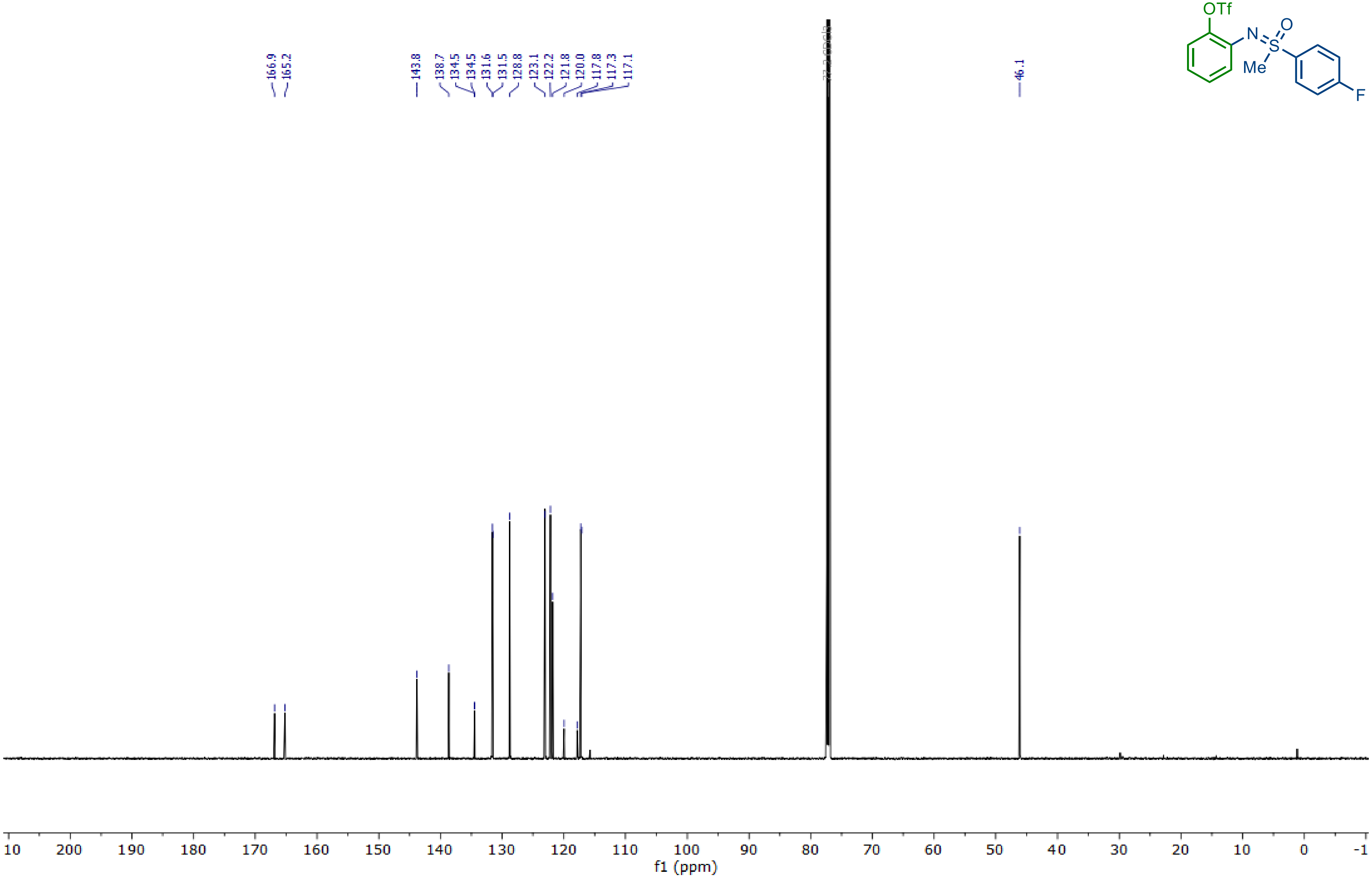

^{19}F NMR of 2-(((4-fluorophenyl)(methyl)(oxo)-λ^{6}-sulfaneylidene)amino)phenyl trifluoromethanesulfonate (14-C3)

471 MHz, $CDCl_3$, 298 K

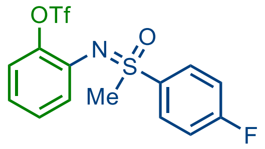

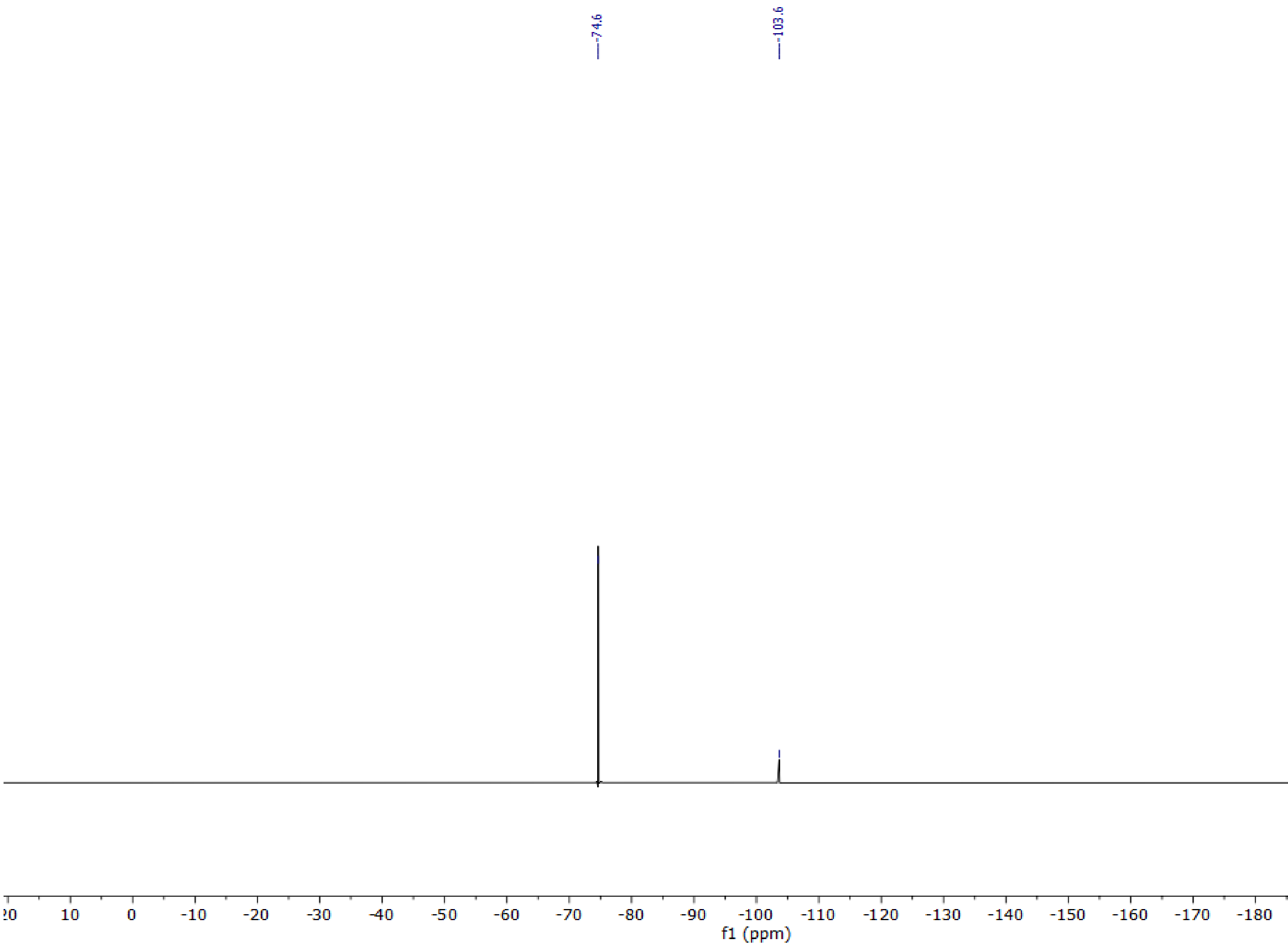

^{1}H NMR of ((4-chlorophenyl)imino)(4-fluorophenyl)(methyl)-λ^{6}-sulfanone (15-C1)

500 MHz, $CDCl_3$, 298 K

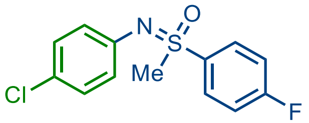

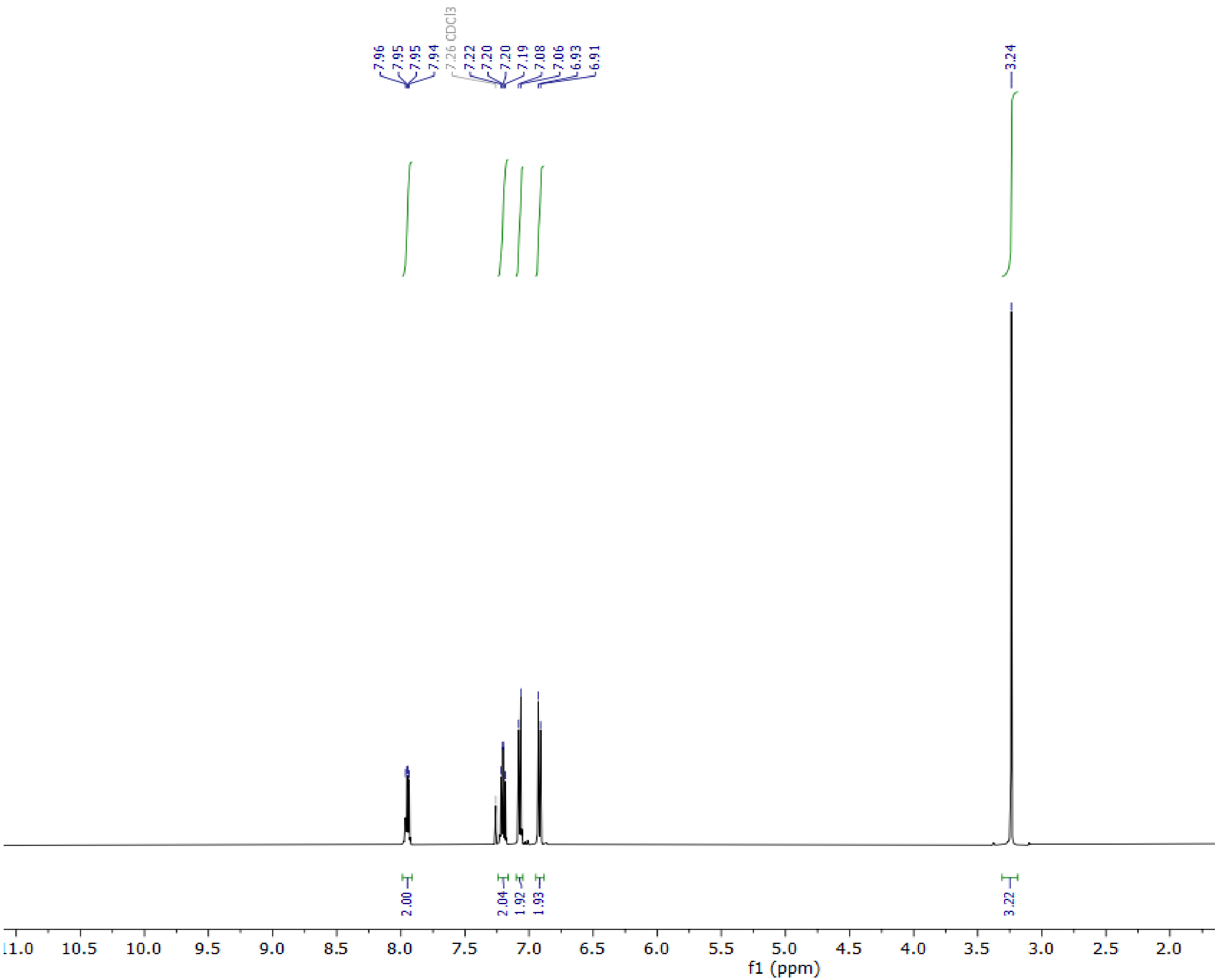

^{13}C NMR of ((4-chlorophenyl)imino)(4-fluorophenyl)(methyl)-λ^6-sulfanone (15-C1)

126 MHz, $CDCl_3$, 298 K

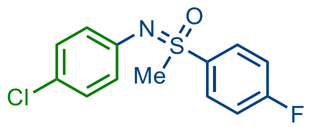

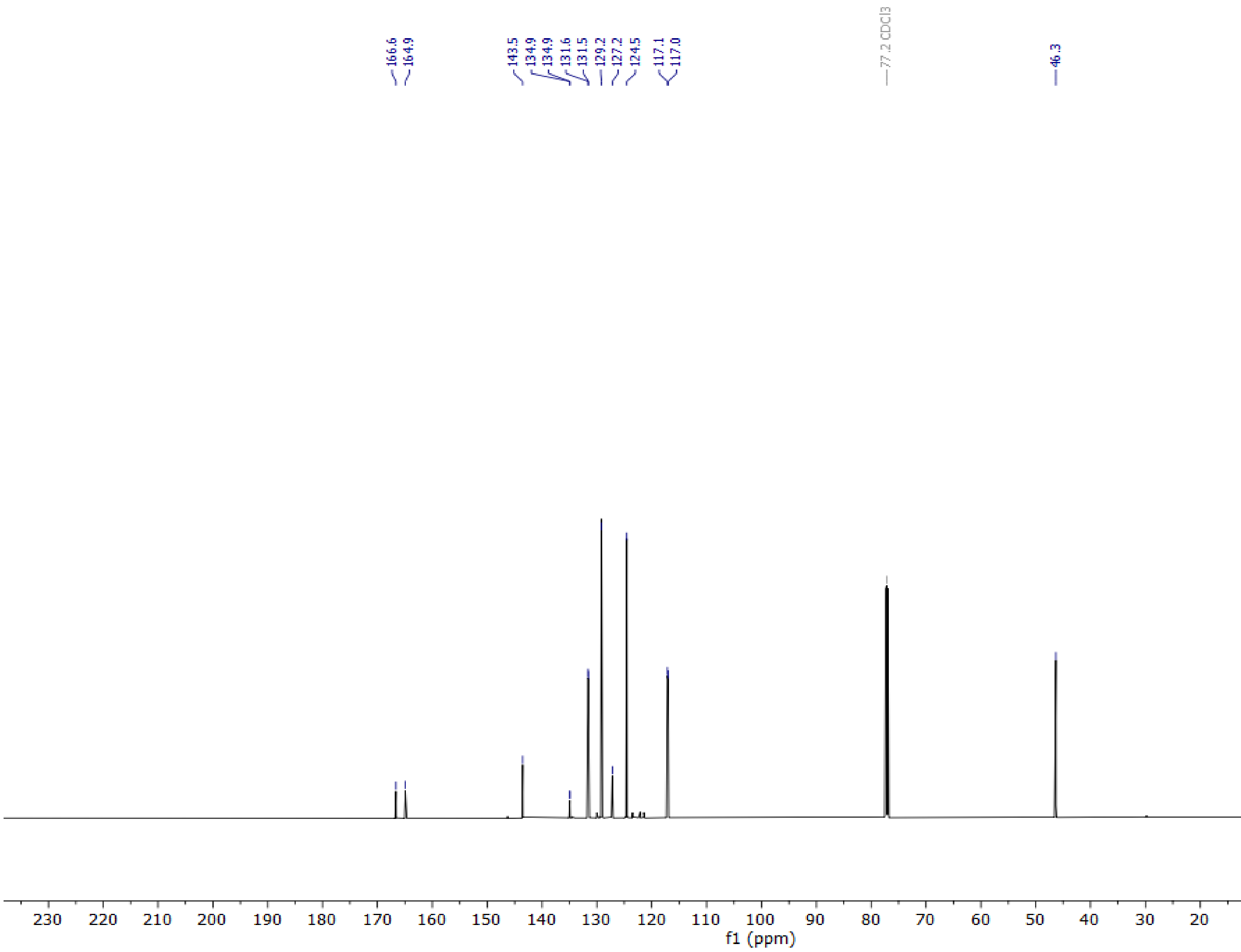

^{19}F NMR of ((4-chlorophenyl)imino)(4-fluorophenyl)(methyl)-λ^{6}-sulfanone (15-C1)

471 MHz, $CDCl_3$, 298 K

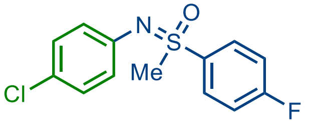

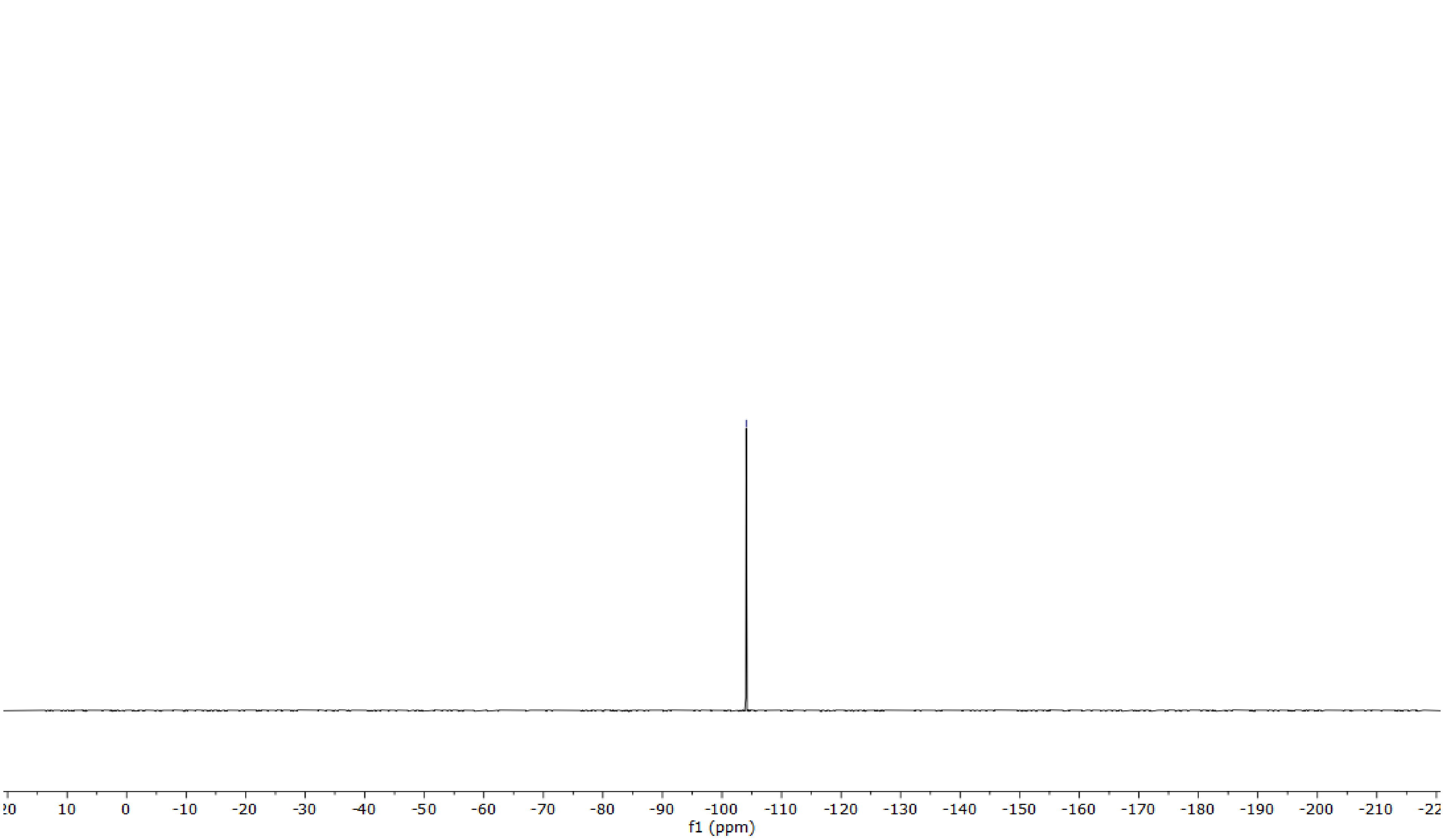

¹H NMR of ((2-chlorophenyl)imino)(4-fluorophenyl)(methyl)-λ⁶-sulfanone (15-C2)

500 MHz, $CDCl_3$, 298 K

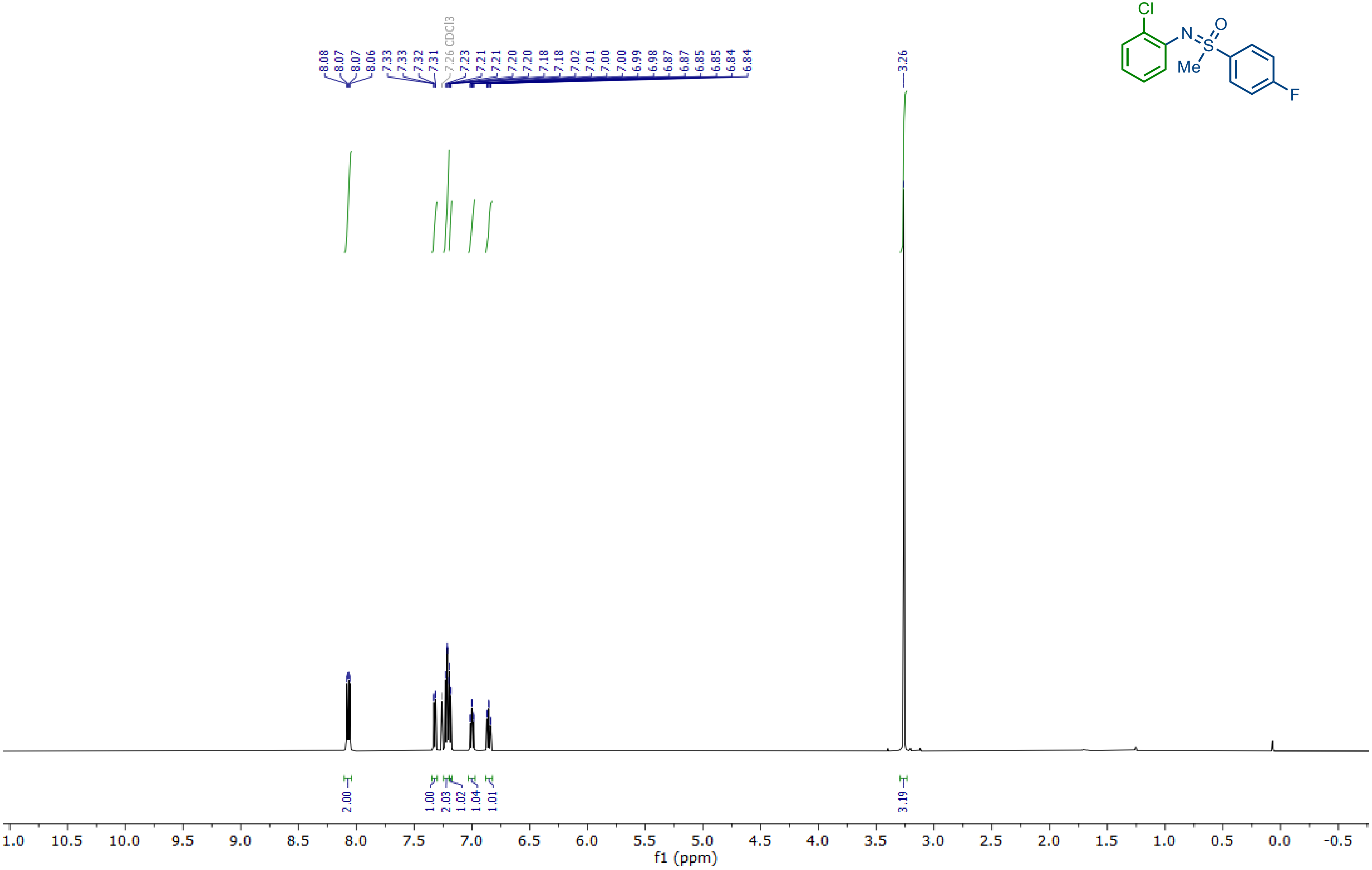

^{13}C NMR of ((2-chlorophenyl)imino)(4-fluorophenyl)(methyl)-λ^6-sulfanone (15-C2)

126 MHz, $CDCl_3$, 298 K

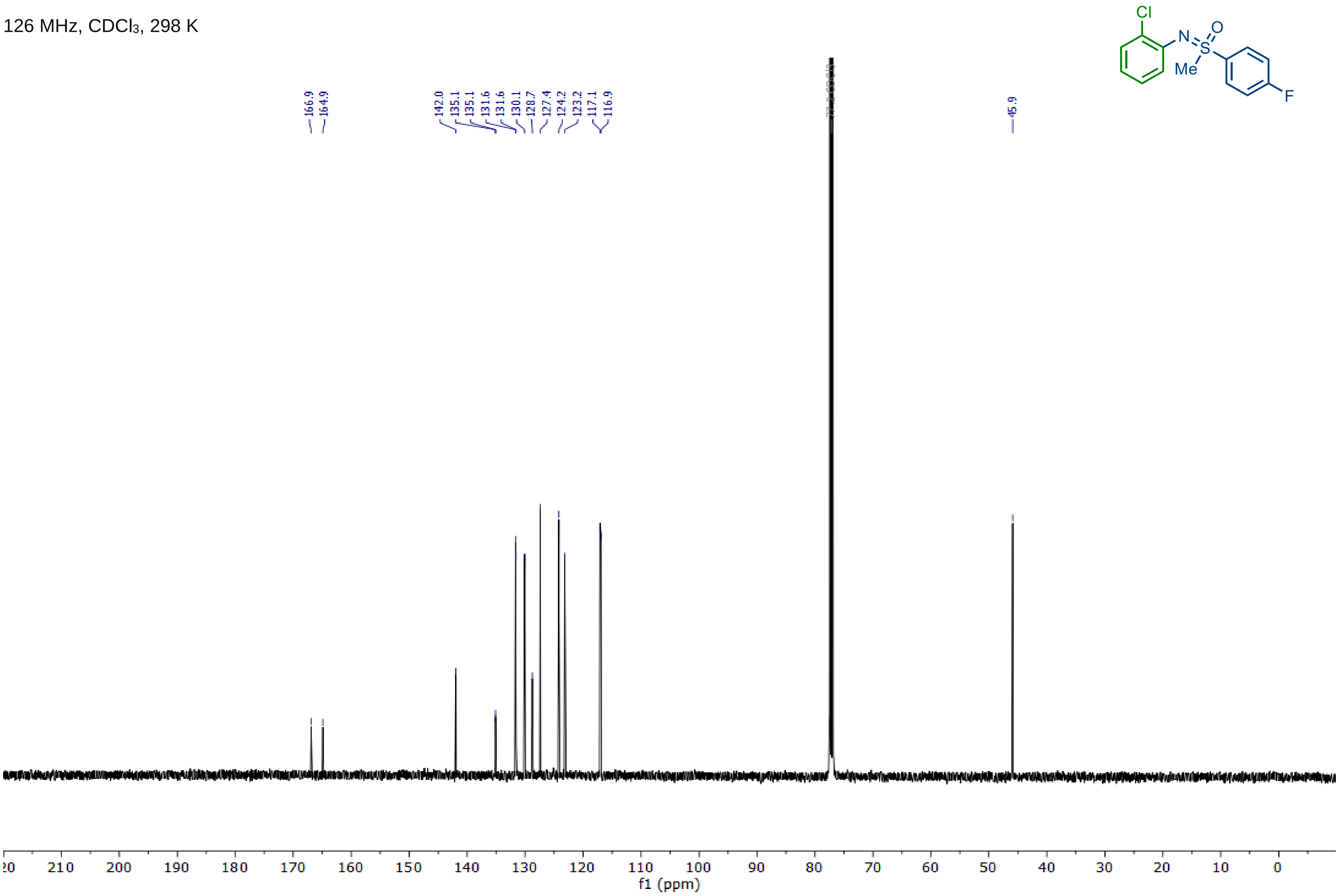

^{19}F NMR of ((2-chlorophenyl)imino)(4-fluorophenyl)(methyl)-λ^6-sulfanone (15-C2)

471 MHz, $CDCl_3$, 298 K

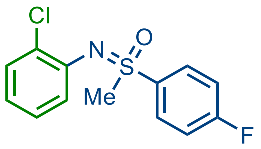

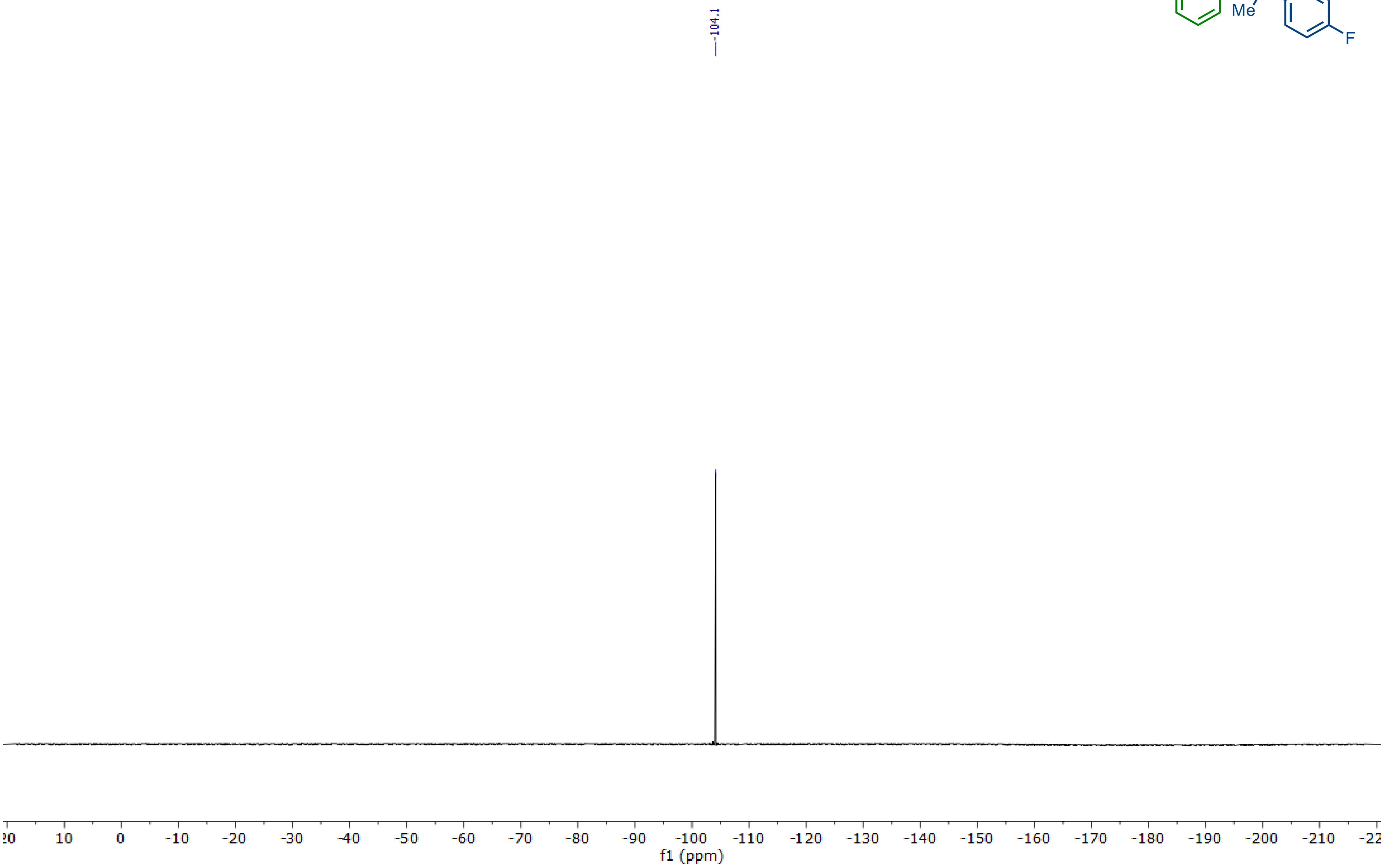

^{1}H NMR of ((3-chlorophenyl)imino)(4-fluorophenyl)(methyl)-λ^6-sulfanone (15-C3)

500 MHz, $CDCl_3$, 298 K

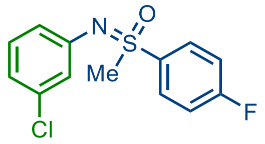

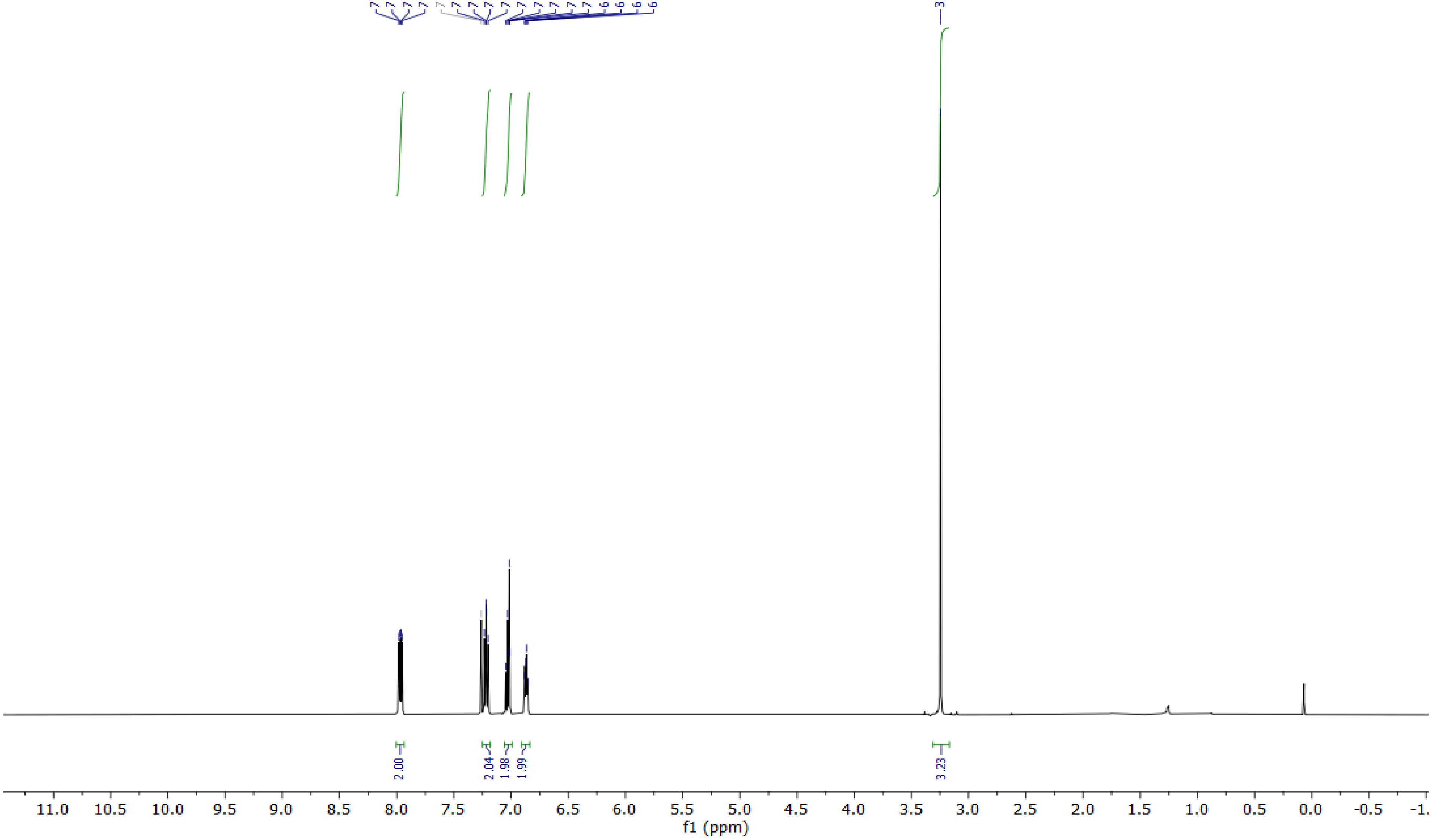

^{13}C NMR of ((3-chlorophenyl)imino)(4-fluorophenyl)(methyl)-λ^6-sulfanone (15-C3)

151 MHz, $CDCl_3$, 298 K

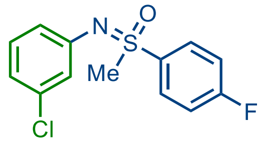

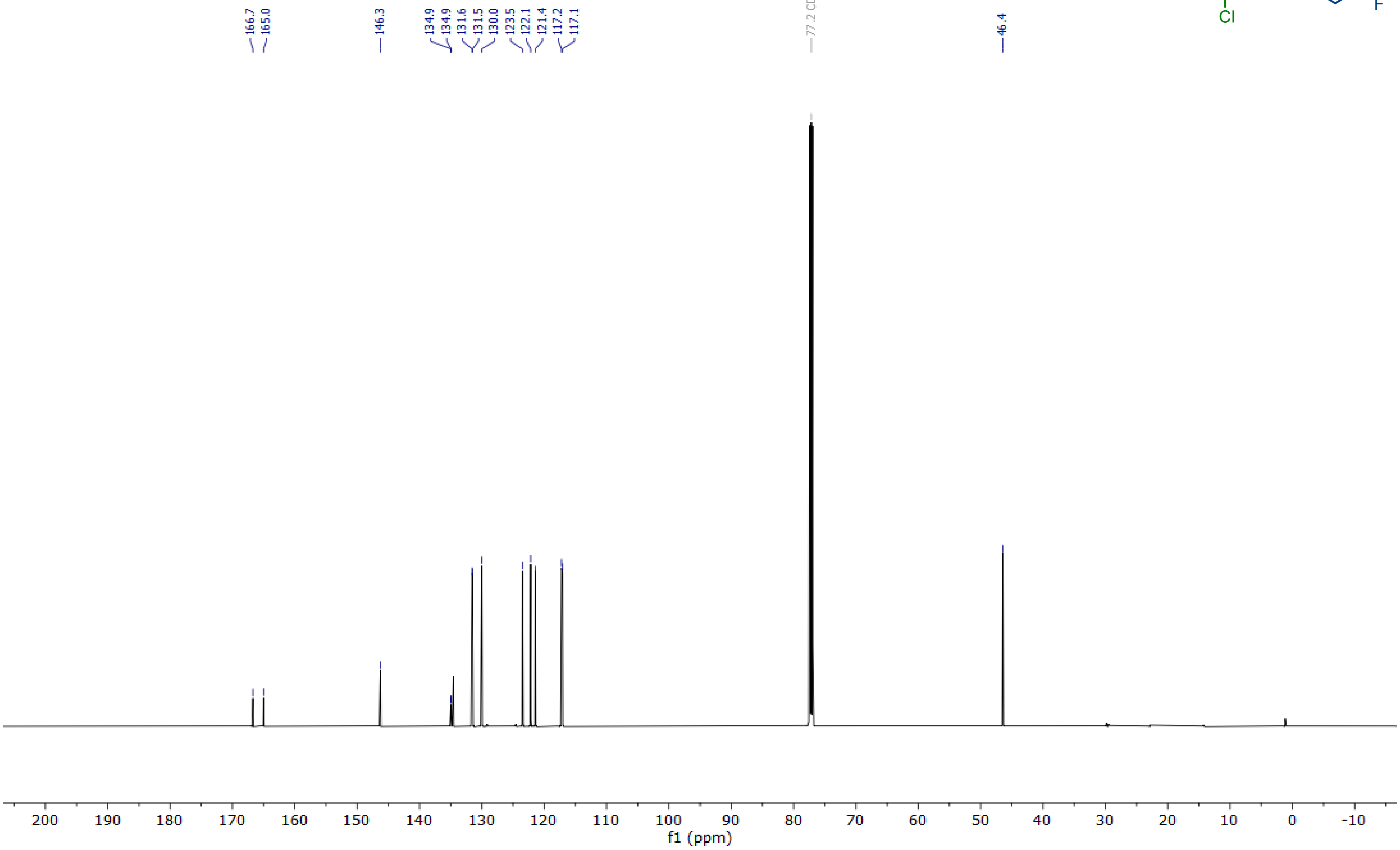

^{19}F NMR of ((3-chlorophenyl)imino)(4-fluorophenyl)(methyl)-λ^6-sulfanone (15-C3)

471 MHz, $CDCl_3$, 298 K

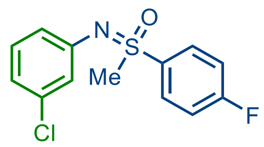

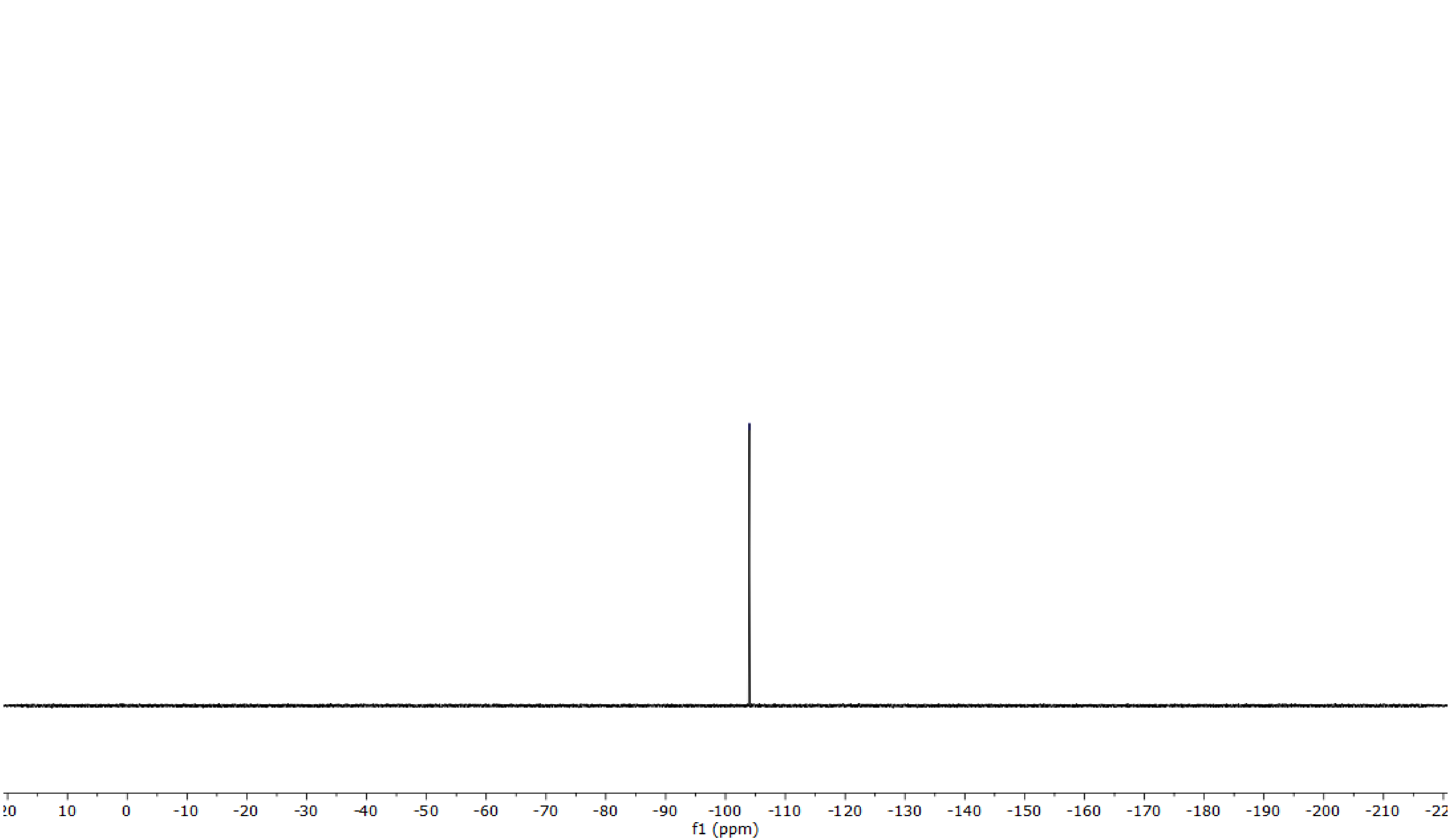

^{1}H NMR of ((2,5-dibromophenyl)imino)(4-fluorophenyl)(methyl)-λ^6-sulfanone (16)

500 MHz, $CDCl_3$, 298 K

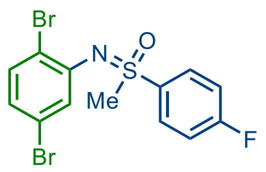

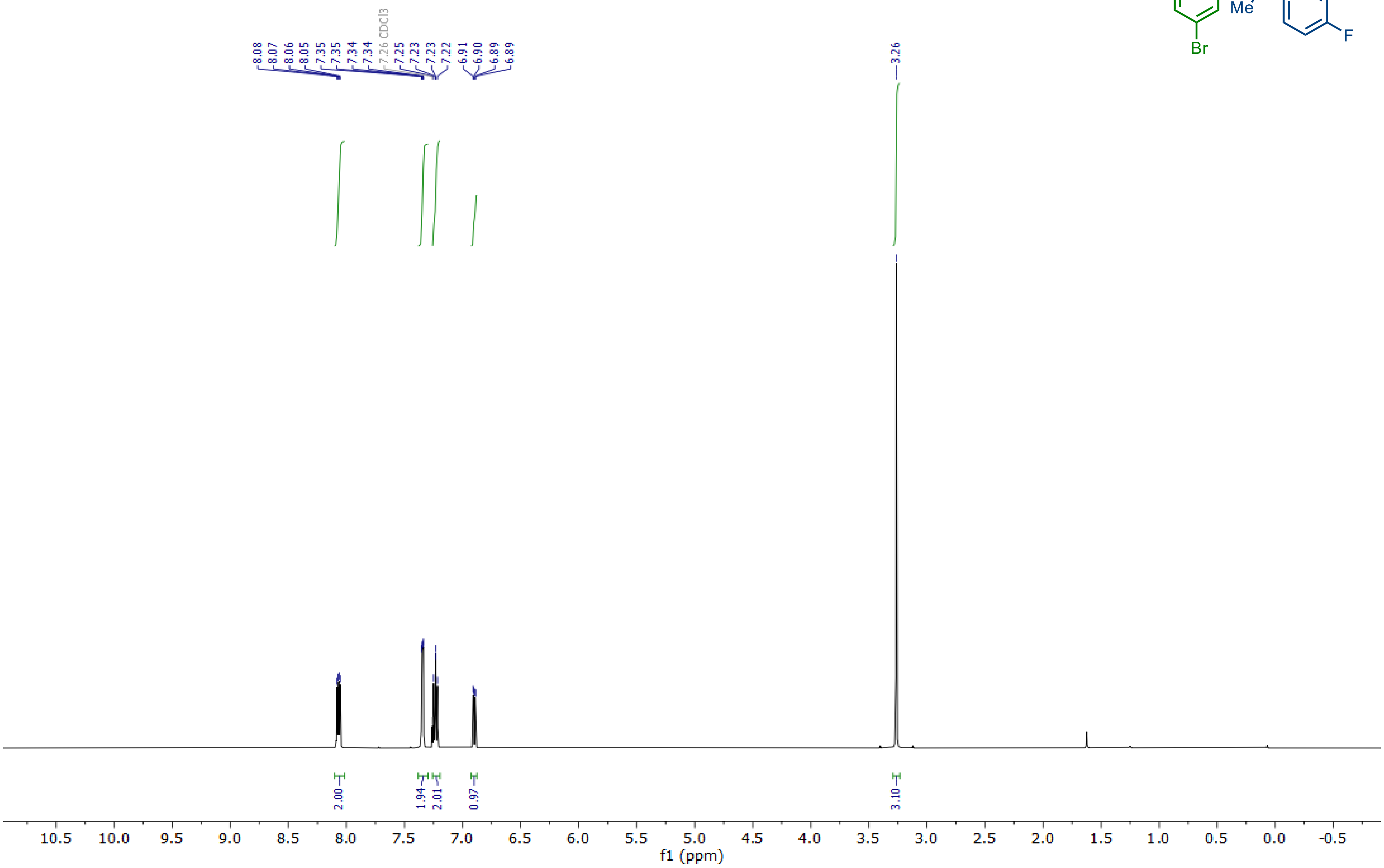

^{13}C NMR of ((2,5-dibromophenyl)imino)(4-fluorophenyl)(methyl)-λ^6-sulfanone (16)

126 MHz, $CDCl_3$, 298 K

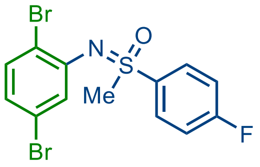

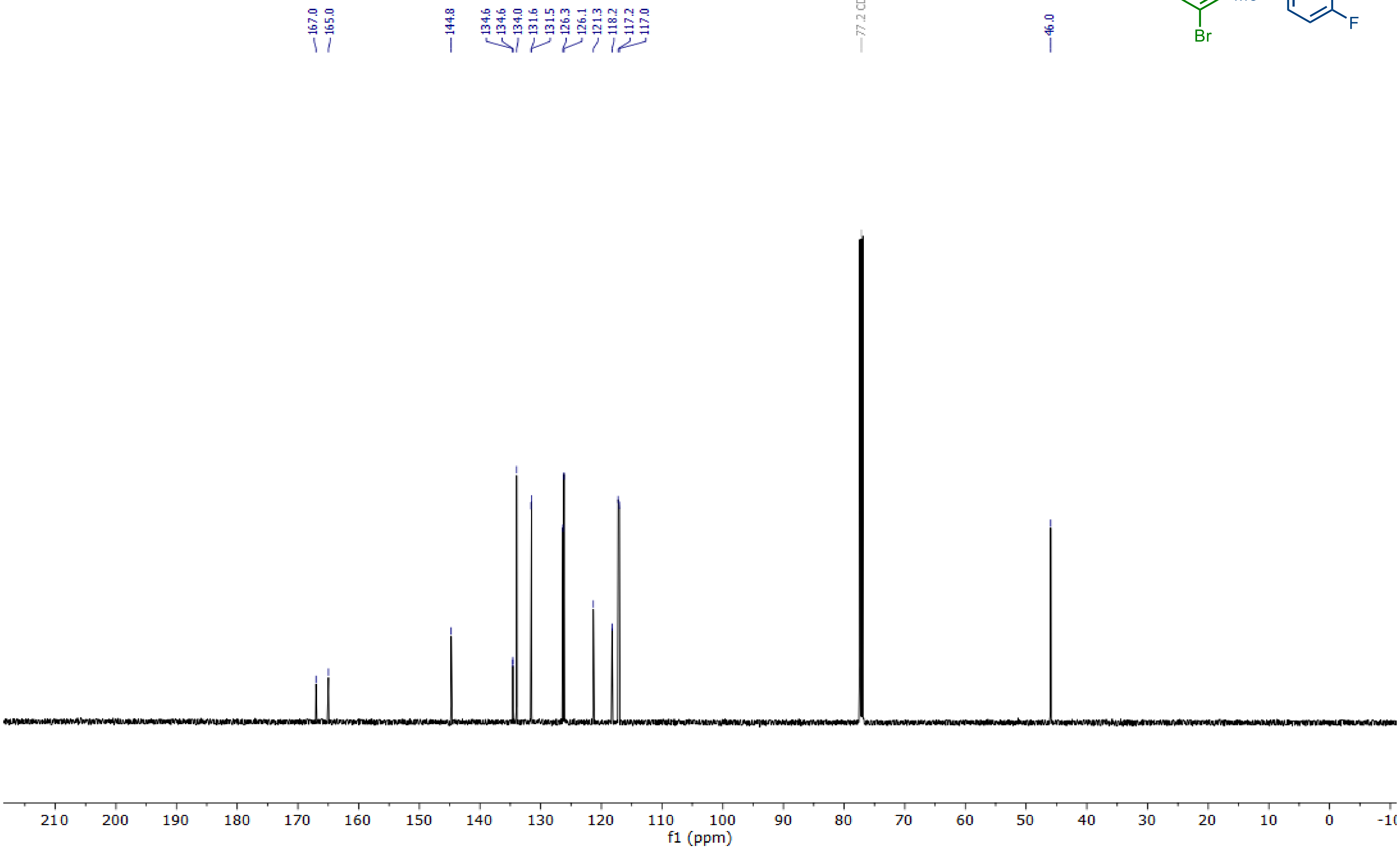

^{19}F NMR of ((2,5-dibromophenyl)imino)(4-fluorophenyl)(methyl)-λ^{6}-sulfanone (16)

471 MHz, $CDCl_3$, 298 K

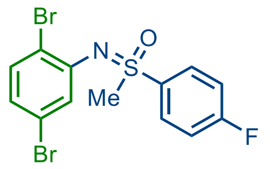

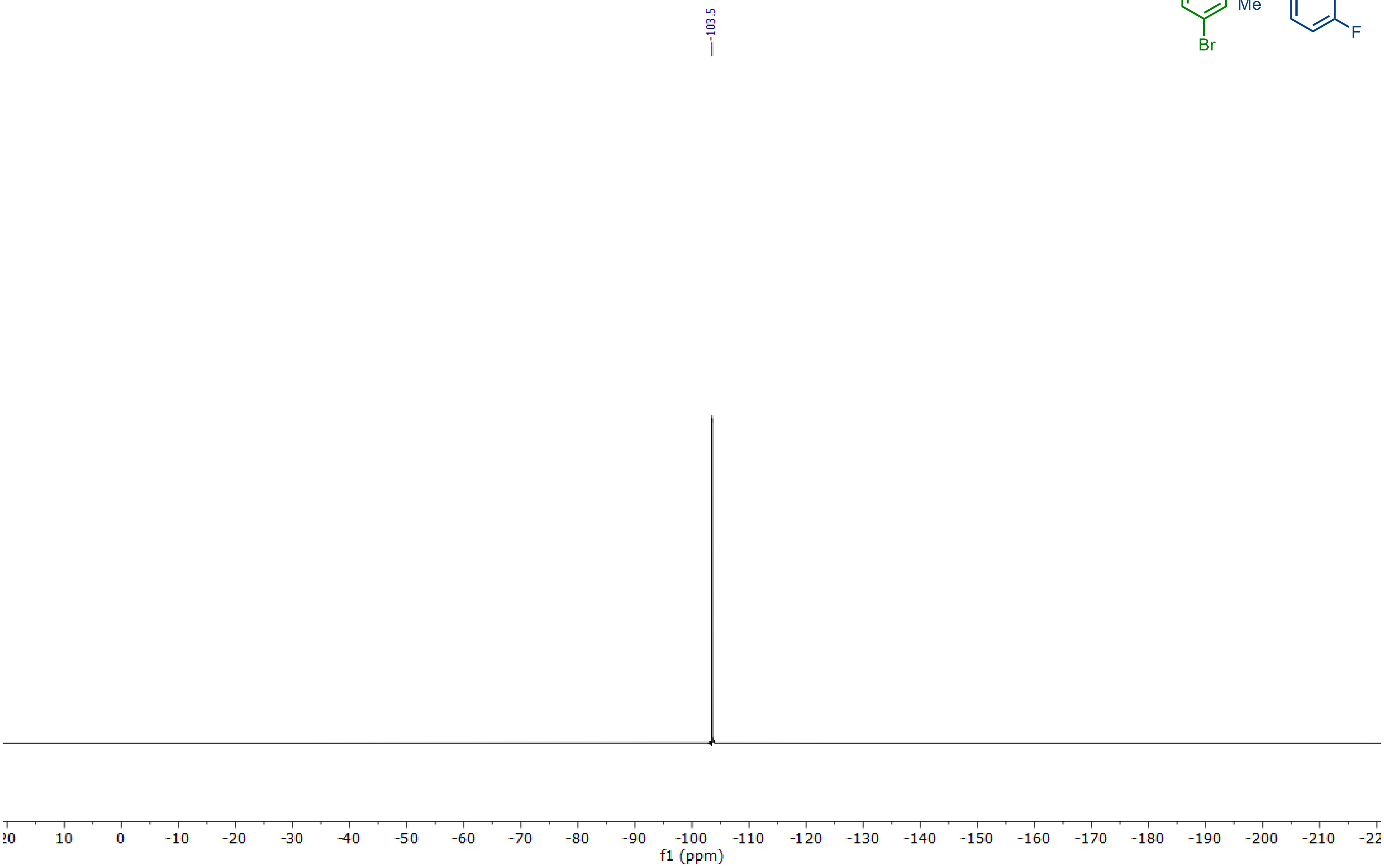

^{1}H NMR of ((5-(tert-butyl)-2-chlorophenyl)imino)(4-fluorophenyl)(methyl)-λ[6]-sulfanone (17)

500 MHz, $CDCl_3$, 298 K

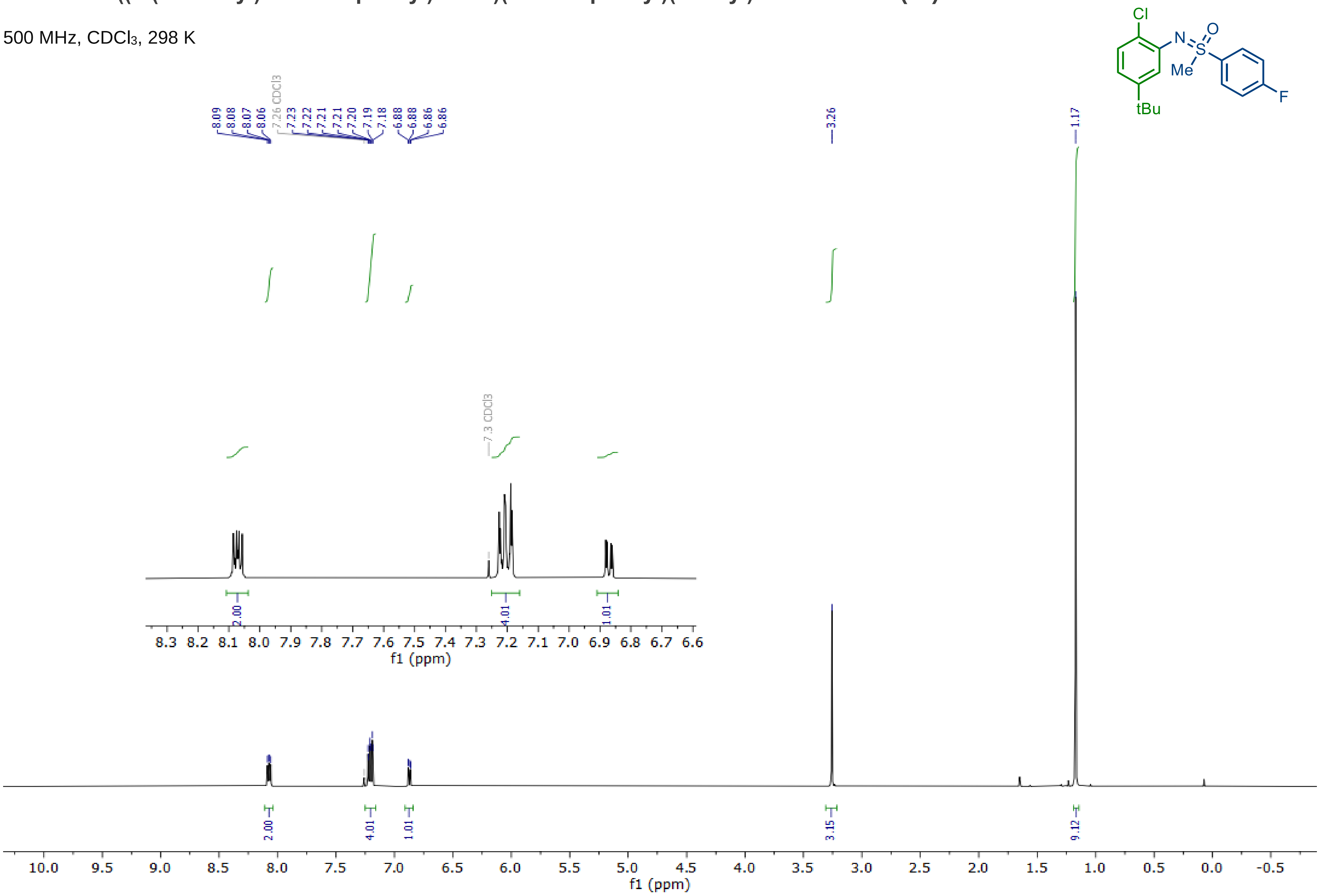

^{13}C NMR of ((5-(tert-butyl)-2-chlorophenyl)imino)(4-fluorophenyl)(methyl)-λ^{6}-sulfanone (17)

126 MHz, $CDCl_3$, 298 K

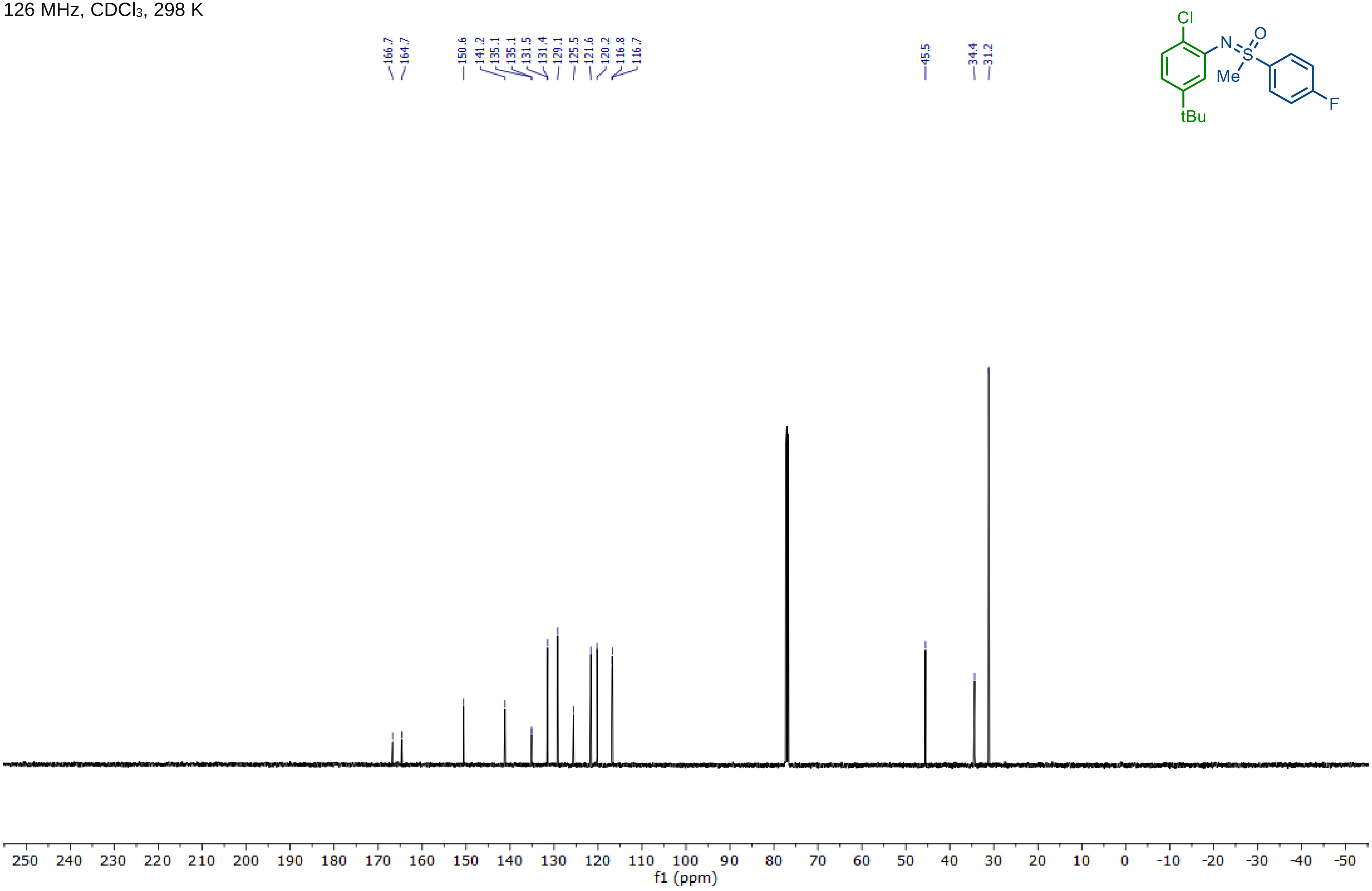

^{19}F NMR of ((5-(tert-butyl)-2-chlorophenyl)imino)(4-fluorophenyl)(methyl)-λ^6-sulfanone (17)

471 MHz, $CDCl_3$, 298 K

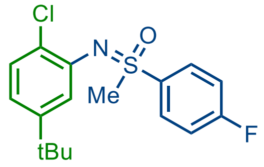

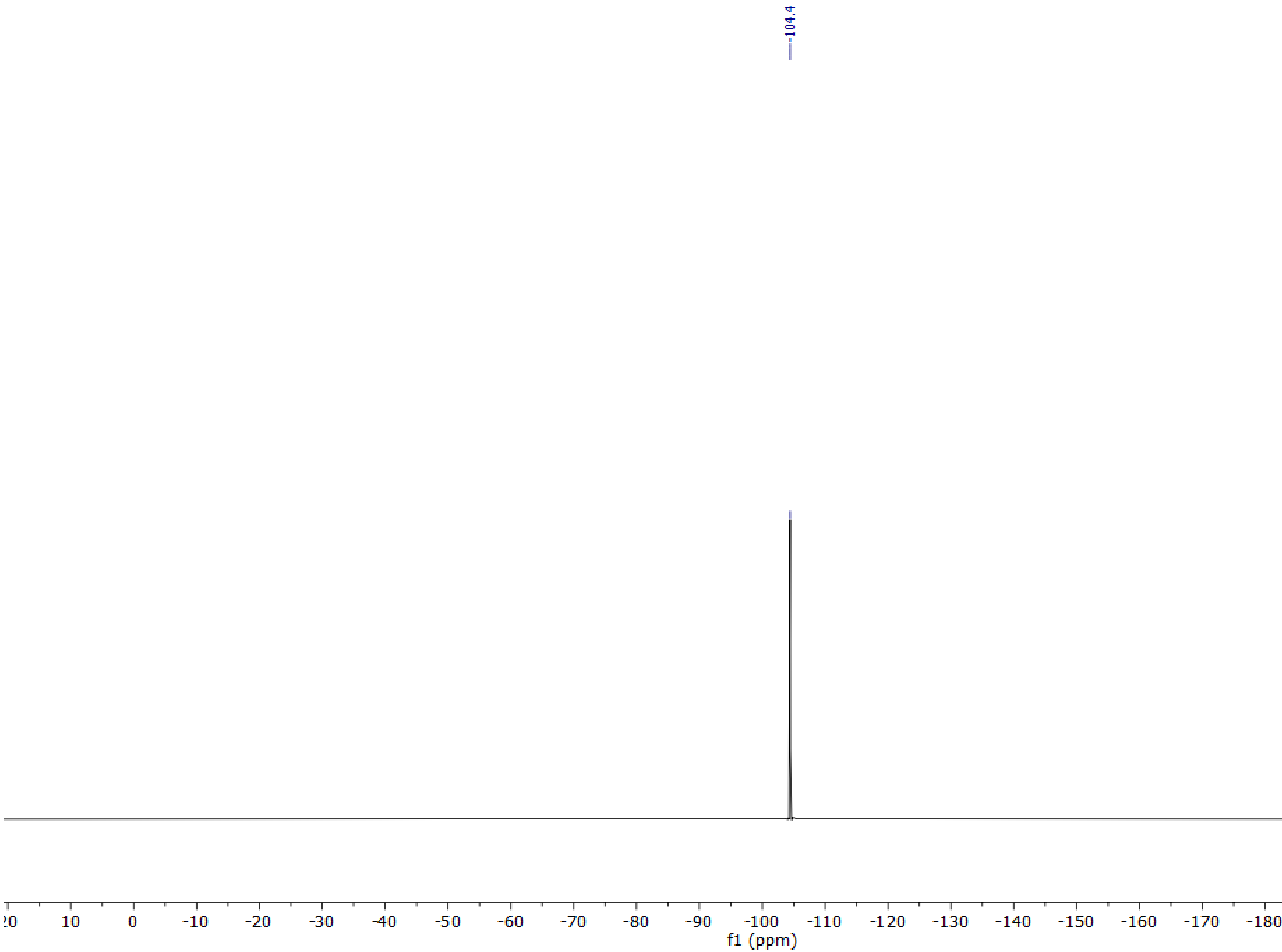

^{1}H NMR of (((1-chloro-2-methylpropan-2-yl)phenyl)imino)(4-fluorophenyl)(methyl)-λ[6]-sulfanone (18)

500 MHz, $CDCl_3$, 298 K

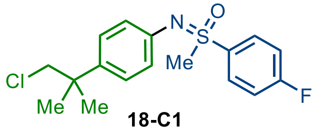

18-C1

+

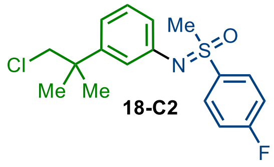

18-C2

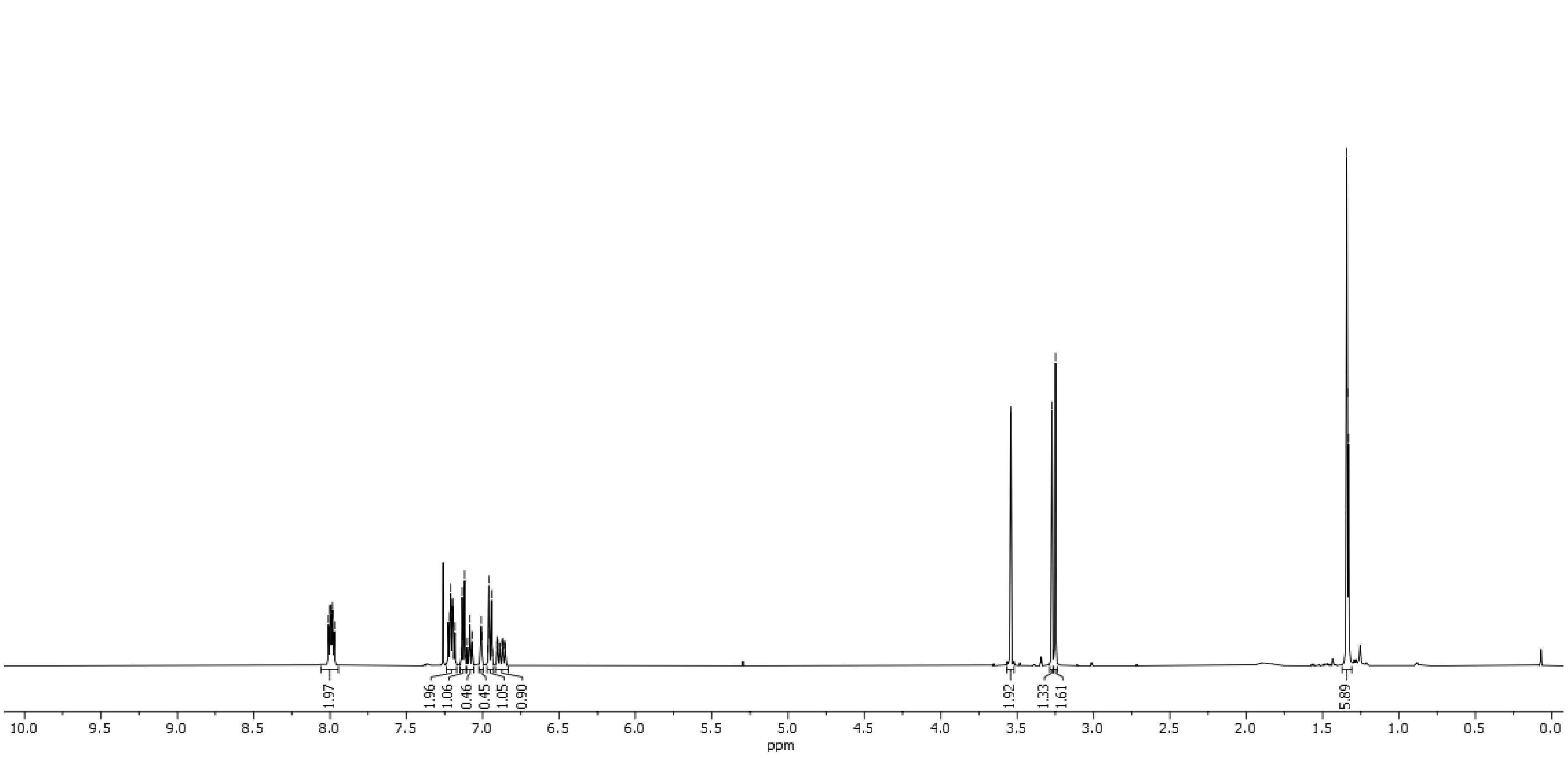

^{13}C NMR of (((1-chloro-2-methylpropan-2-yl)phenyl)imino)(4-fluorophenyl)(methyl)-λ^{6}-sulfanone (18)

126 MHz, $CDCl_3$, 298 K

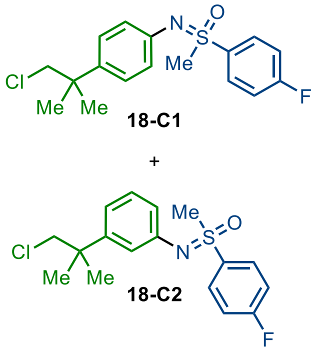

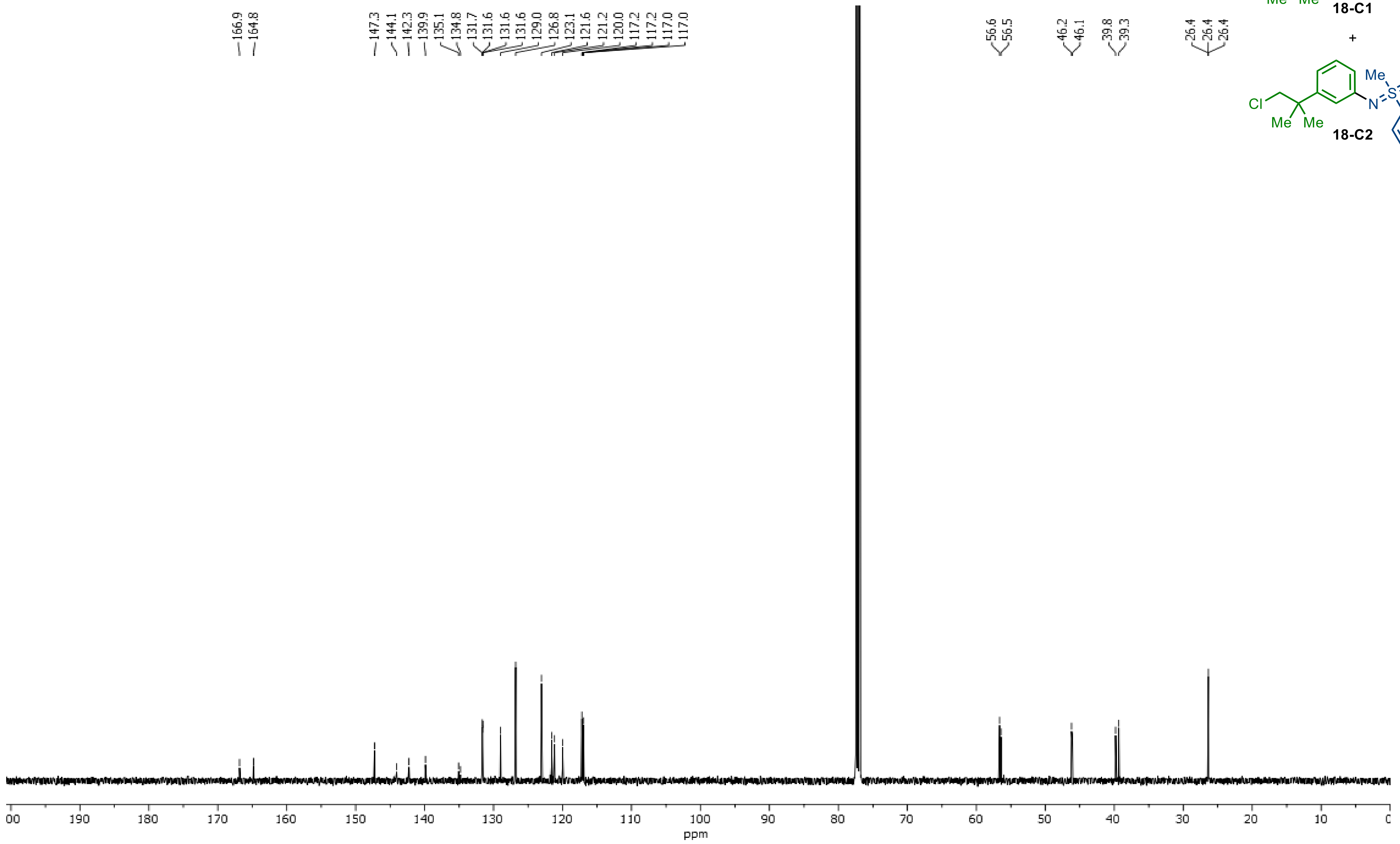

^{19}F NMR of (((1-chloro-2-methylpropan-2-yl)phenyl)imino)(4-fluorophenyl)(methyl)-λ^{6}-sulfanone (18)

471 MHz, $CDCl_3$, 298 K

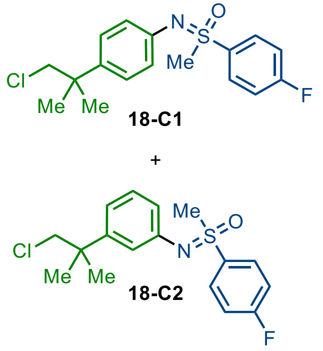

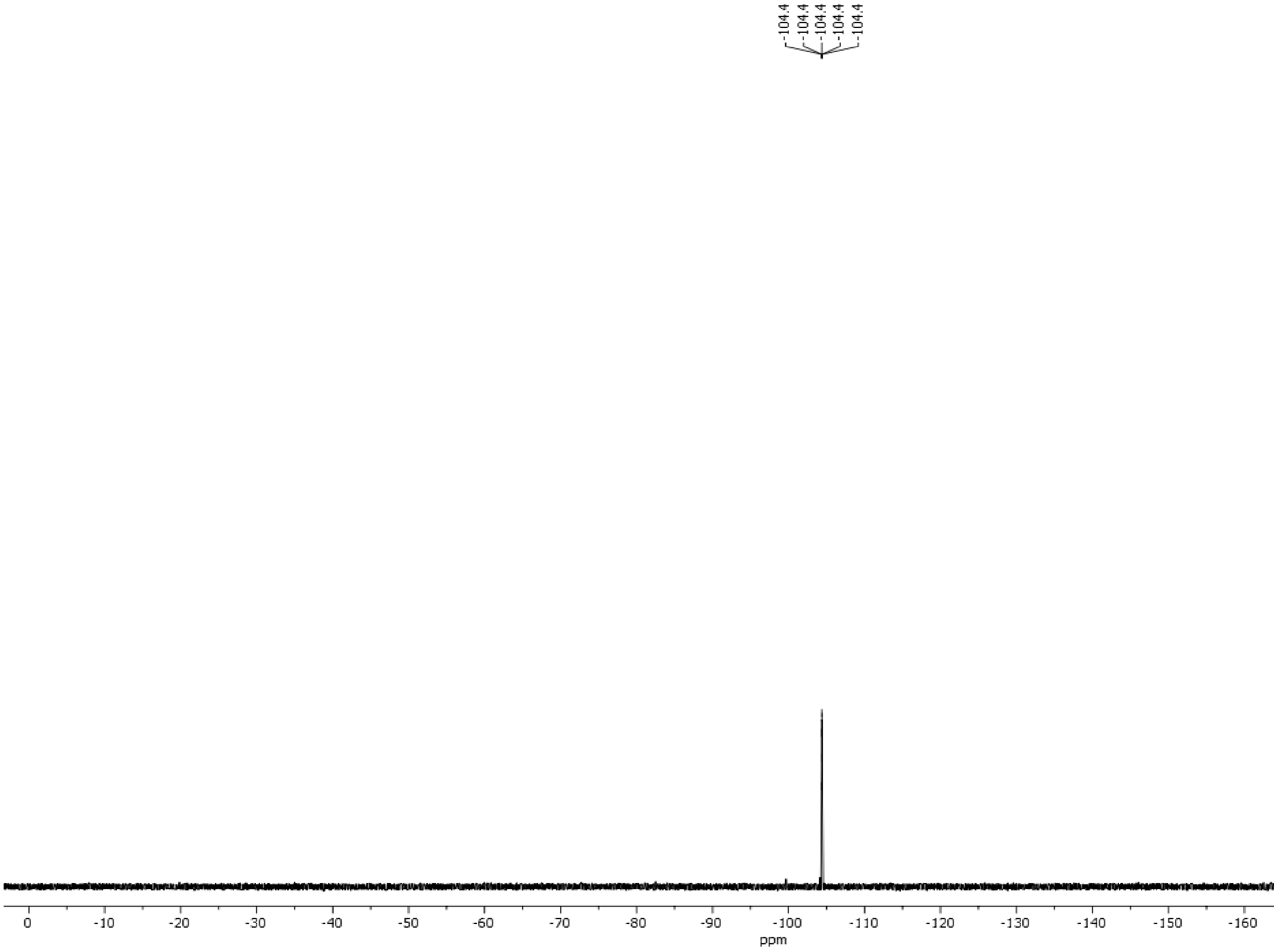

^{1}H NMR of (4-fluorophenyl)(methyl)((4-(trifluoromethoxy)phenyl)imino)-λ^{6}-sulfanone (19-C1)

600 MHz, $CDCl_3$, 298 K

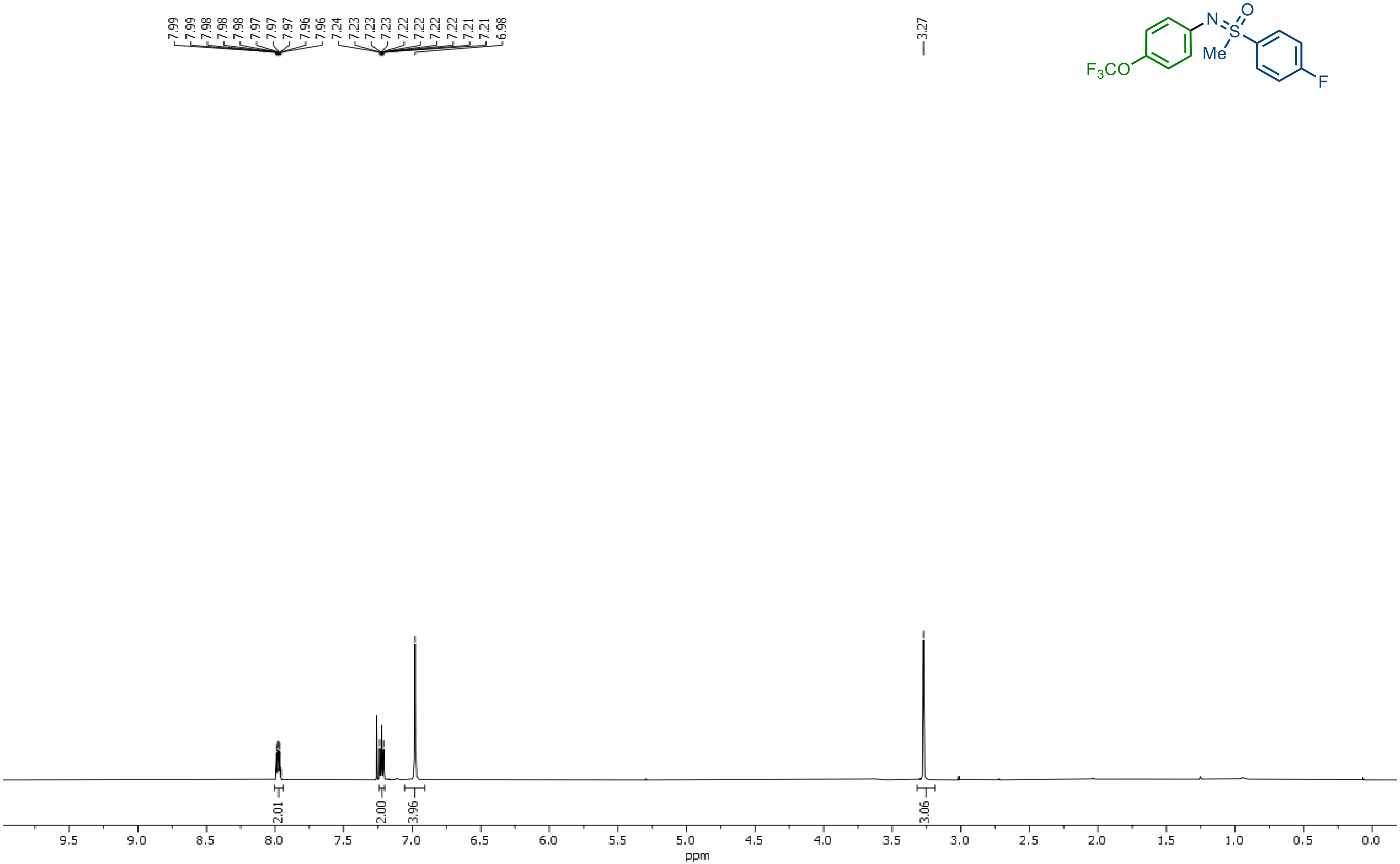

^{13}C NMR of (4-fluorophenyl)(methyl)((4-(trifluoromethoxy)phenyl)imino)-λ^6-sulfanone (19-C1)

151 MHz, $CDCl_3$, 298 K

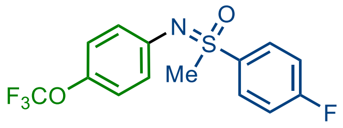

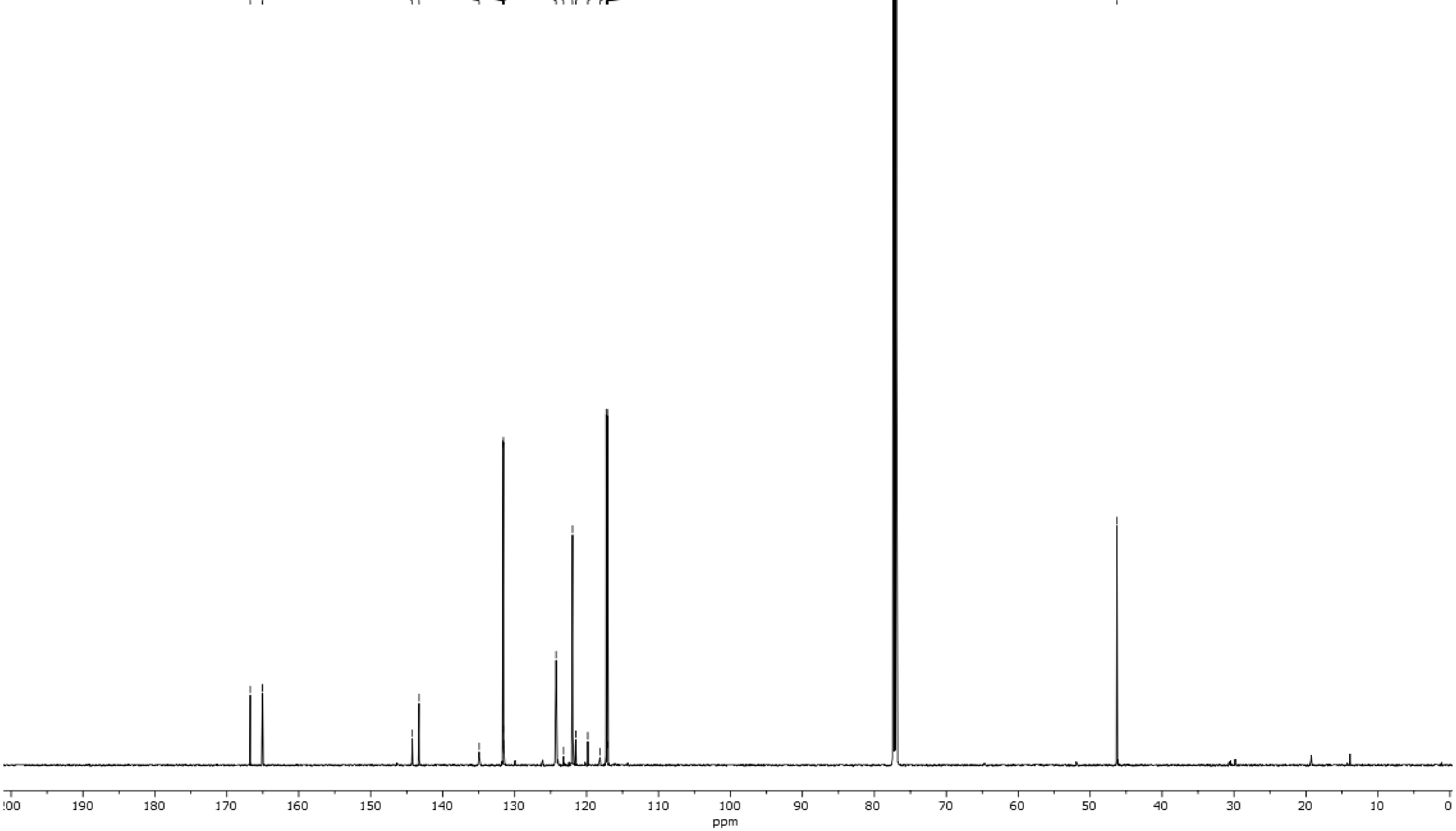

^{19}F NMR of (4-fluorophenyl)(methyl)((4-(trifluoromethoxy)phenyl)imino)-λ^{6}-sulfanone (19-C1)

565 MHz, $CDCl_3$, 298 K

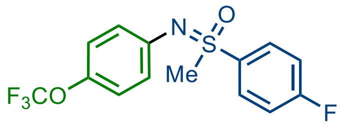

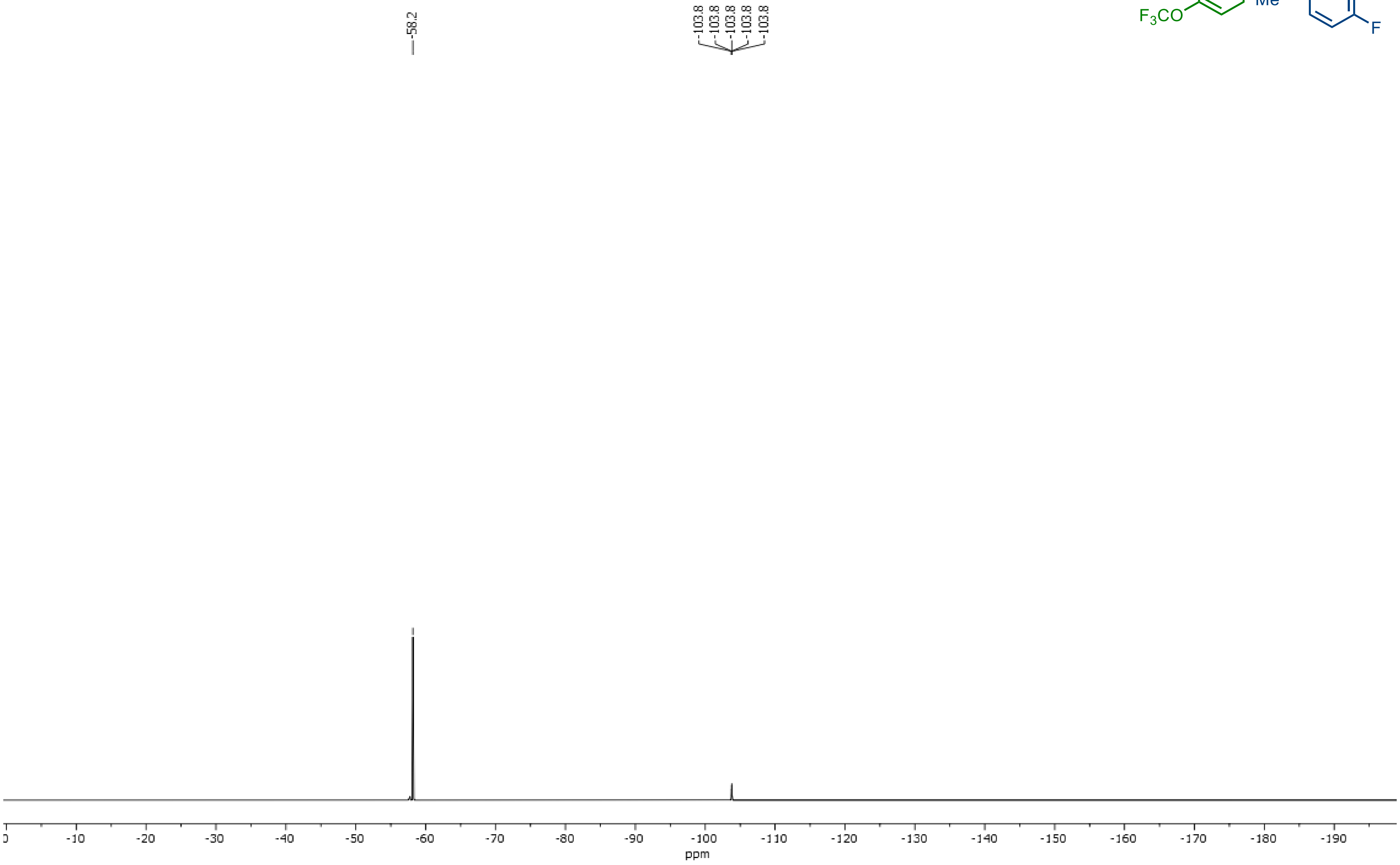

^{1}H NMR of (4-fluorophenyl)(methyl)((3-(trifluoromethoxy)phenyl)imino)-λ[6]-sulfanone (19-C2)

600 MHz, $CDCl_3$, 298 K

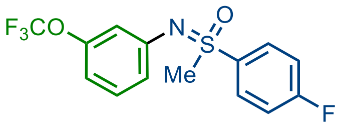

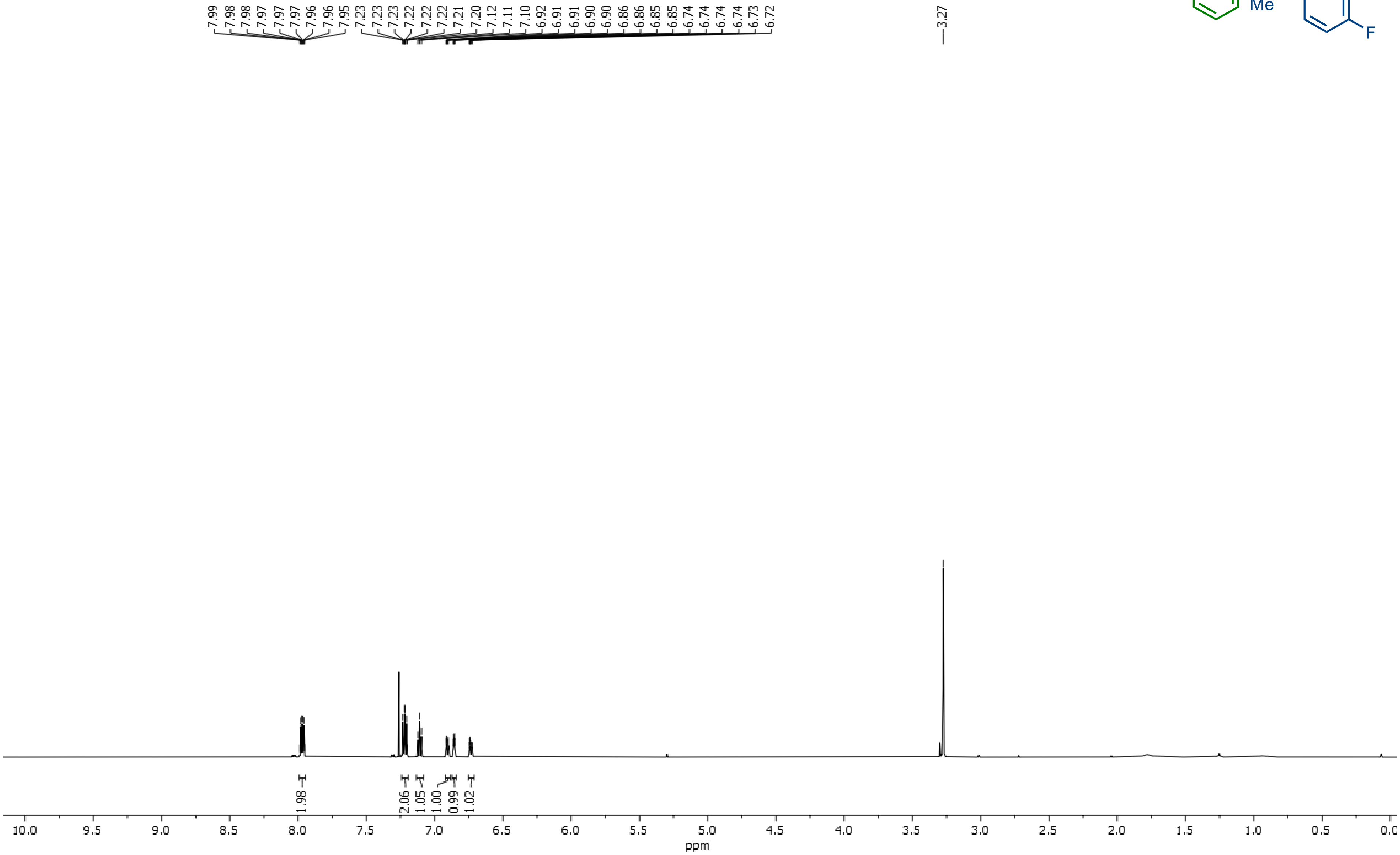

^{13}C NMR of (4-fluorophenyl)(methyl)((3-(trifluoromethoxy)phenyl)imino)-λ^6-sulfanone (19-C2)

151 MHz, $CDCl_3$, 298 K

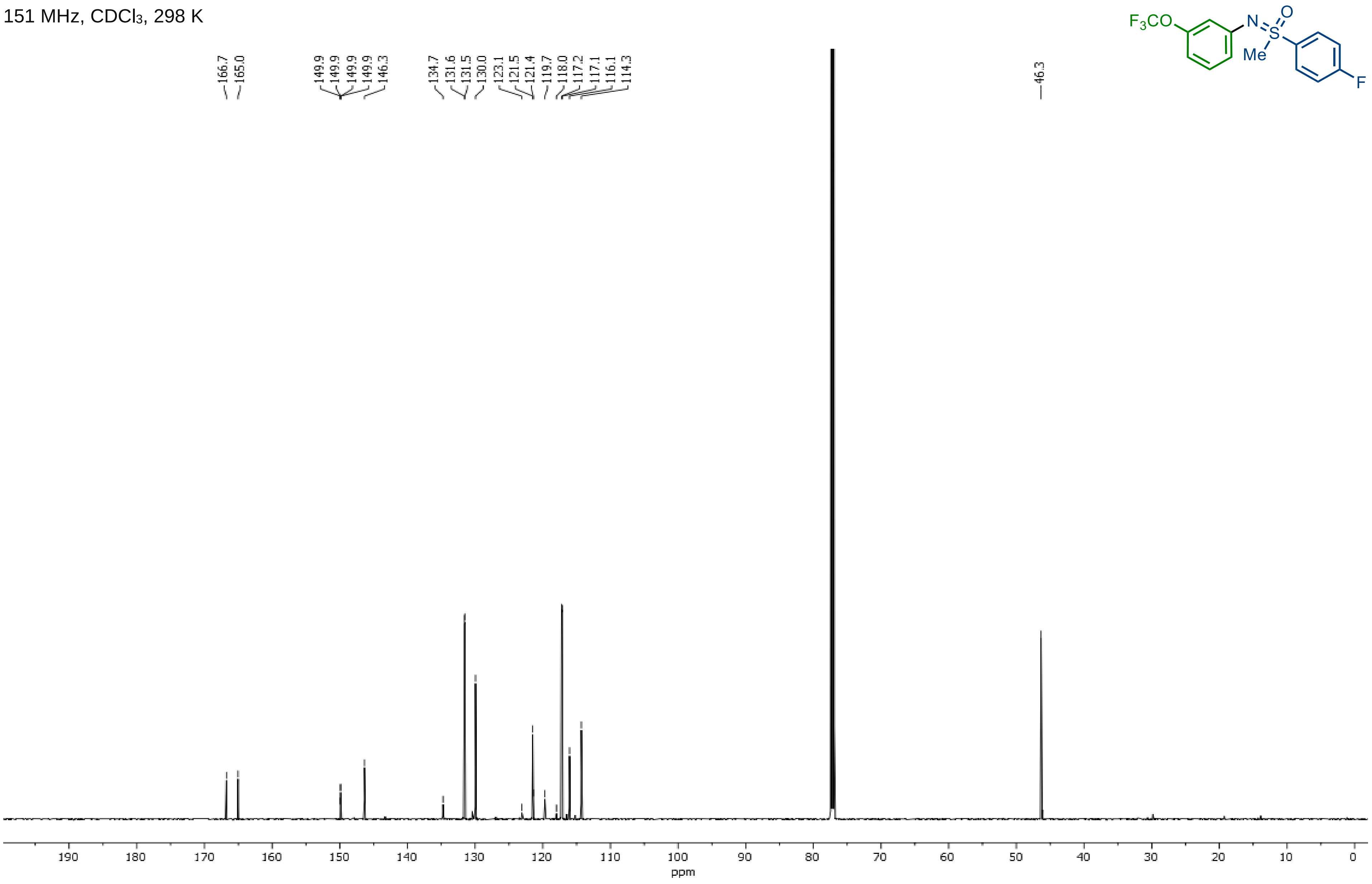

^{19}F NMR of (4-fluorophenyl)(methyl)((3-(trifluoromethoxy)phenyl)imino)-λ^6-sulfanone (19-C2)

565 MHz, $CDCl_3$, 298 K

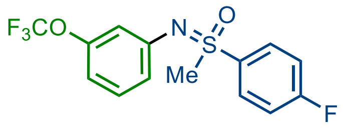

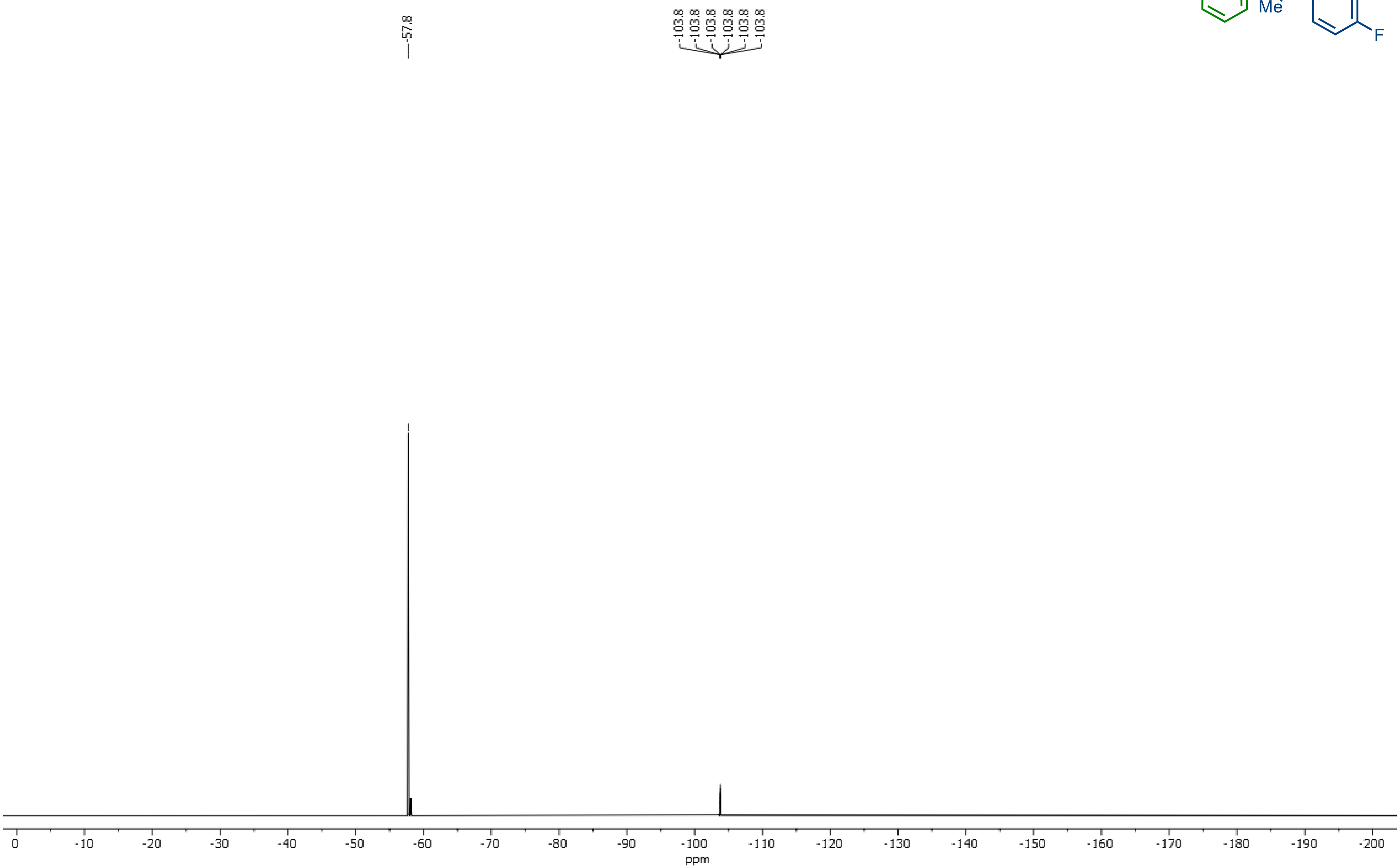

^{1}H NMR of (4-fluorophenyl)(methyl)((2-(trifluoromethoxy)phenyl)imino)-λ[6]-sulfanone (19-C3)

600 MHz, $CDCl_3$, 298 K

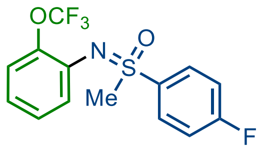

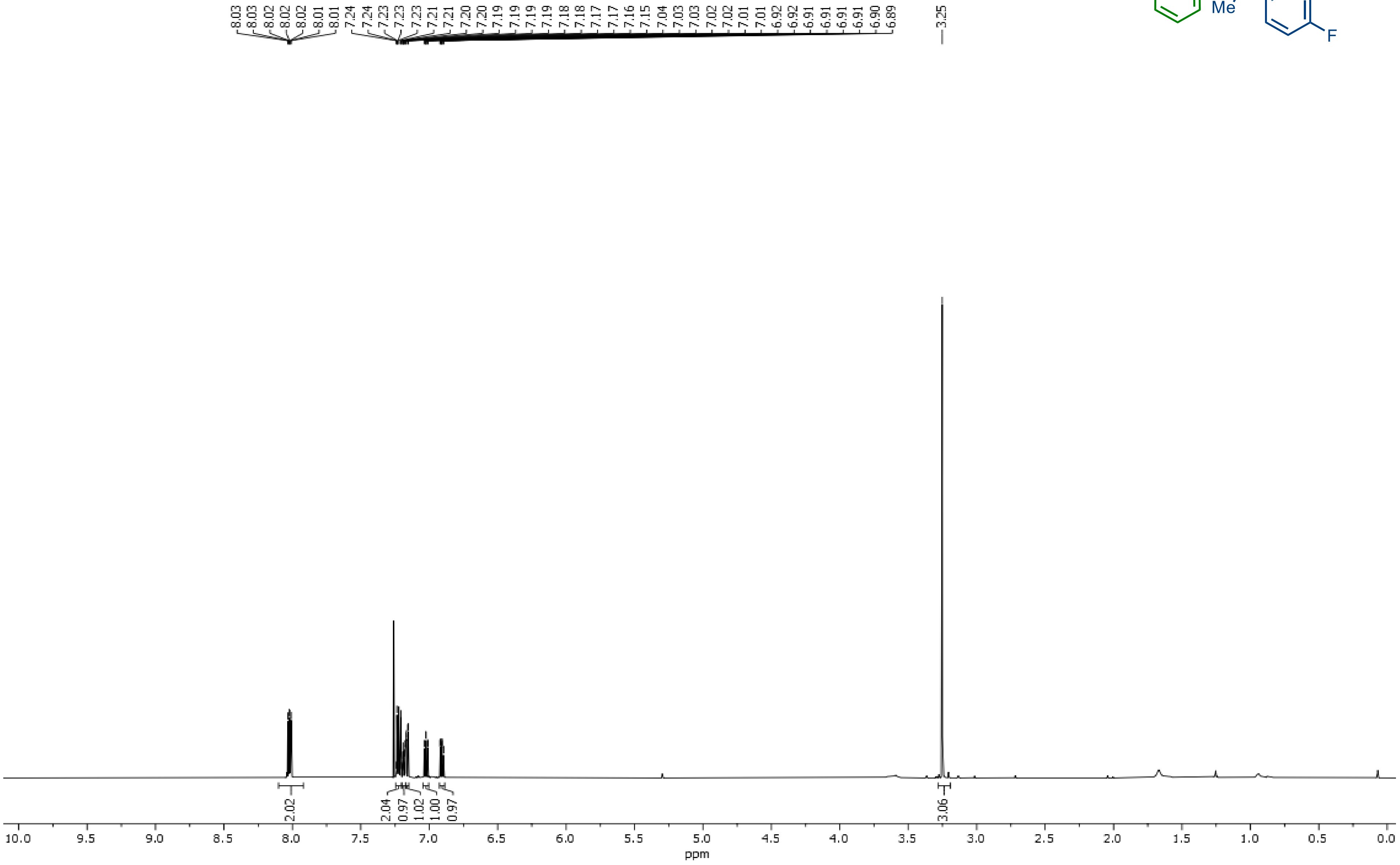

^{13}C NMR of (4-fluorophenyl)(methyl)((2-(trifluoromethoxy)phenyl)imino)-λ^6-sulfanone (19-C3)

151 MHz, $CDCl_3$, 298 K

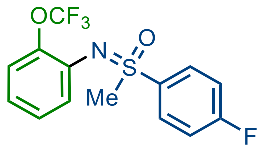

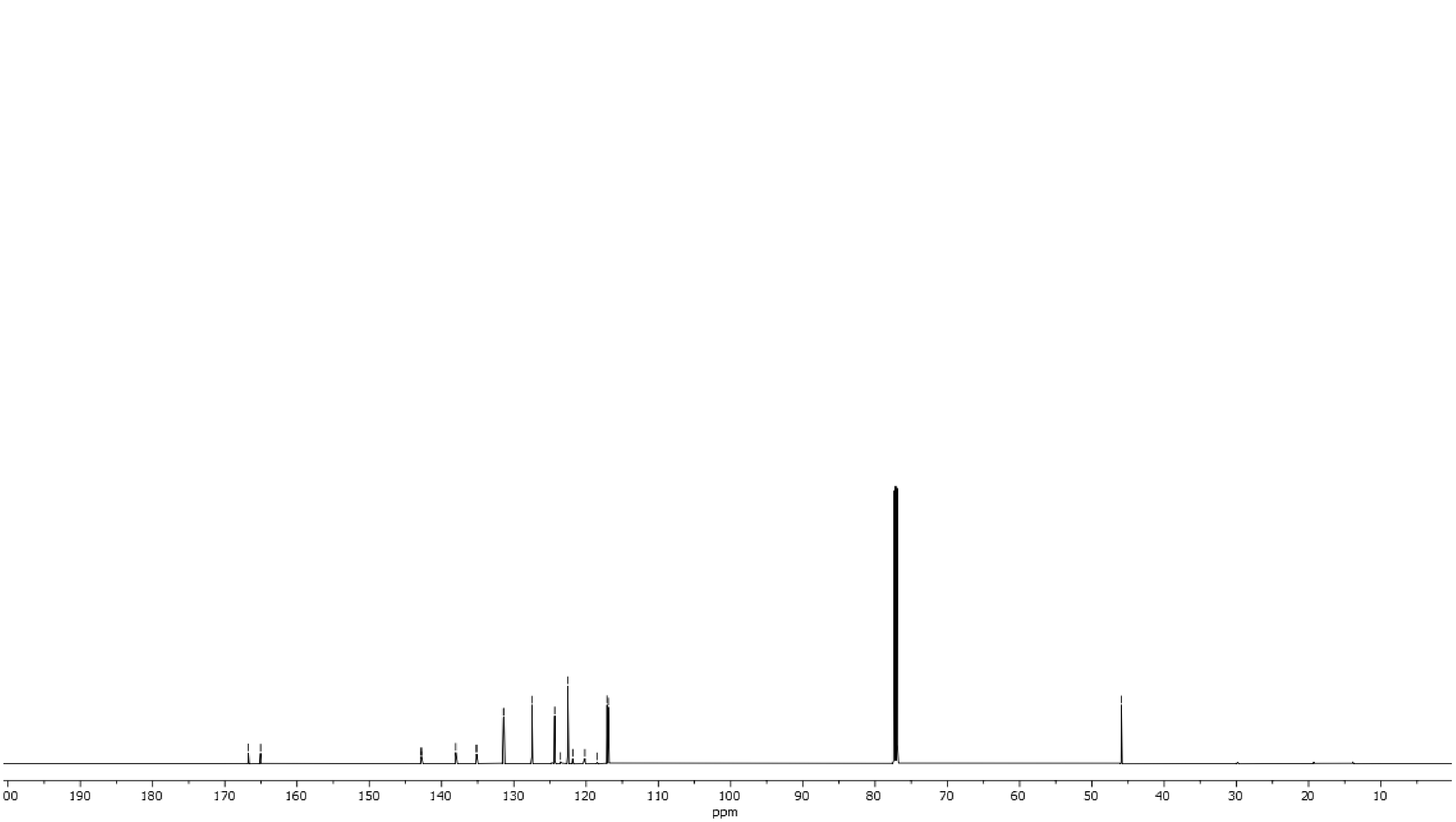

^{19}F NMR of (4-fluorophenyl)(methyl)((2-(trifluoromethoxy)phenyl)imino)-λ^{6}-sulfanone (19-C3)

565 MHz, $CDCl_3$, 298 K

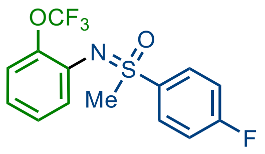

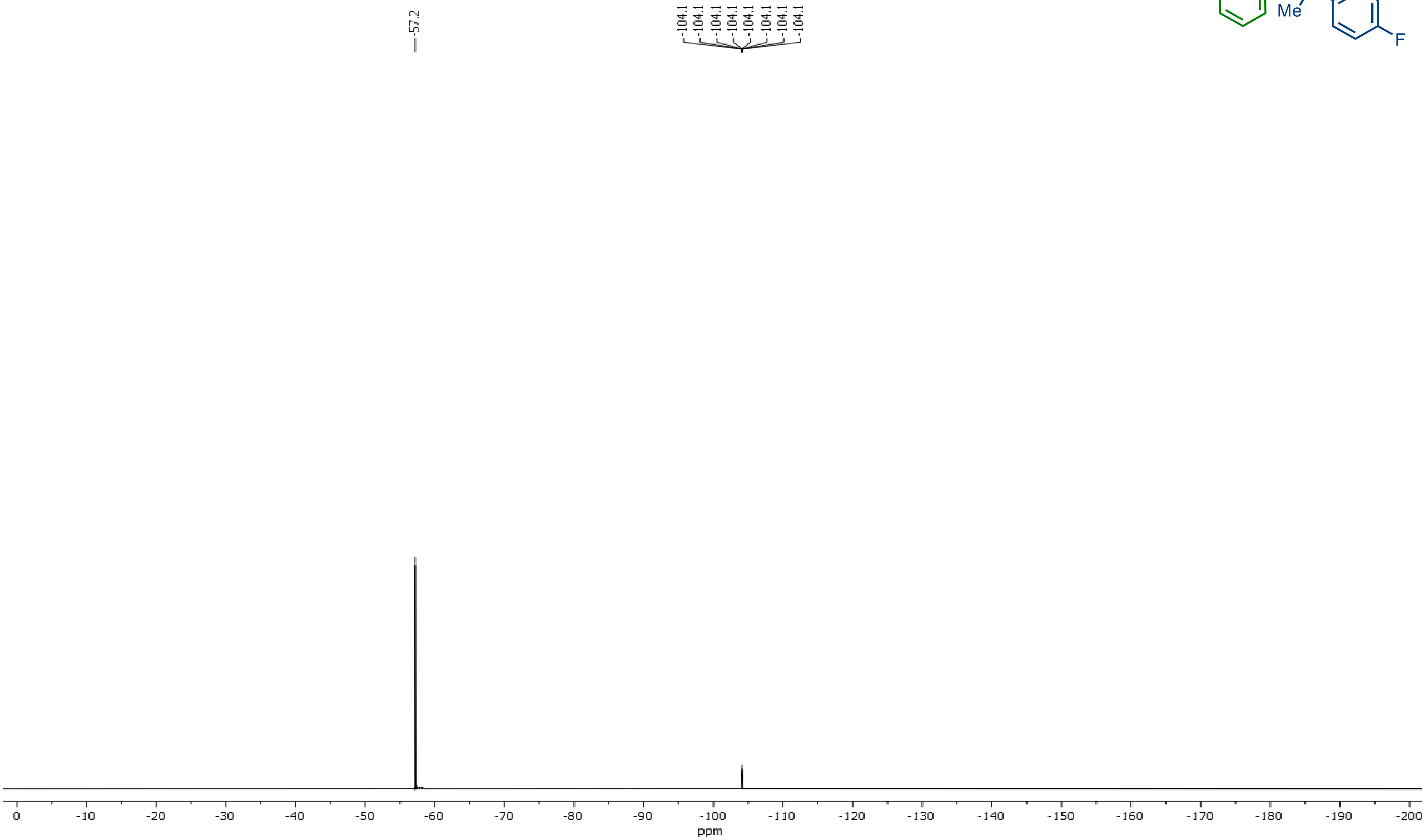

^{1}H NMR of ((4-bromophenyl)imino)(4-fluorophenyl)(methyl)-λ^{6}-sulfanone (20-C1)

500 MHz, $CDCl_3$, 298 K

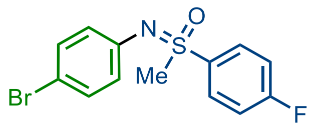

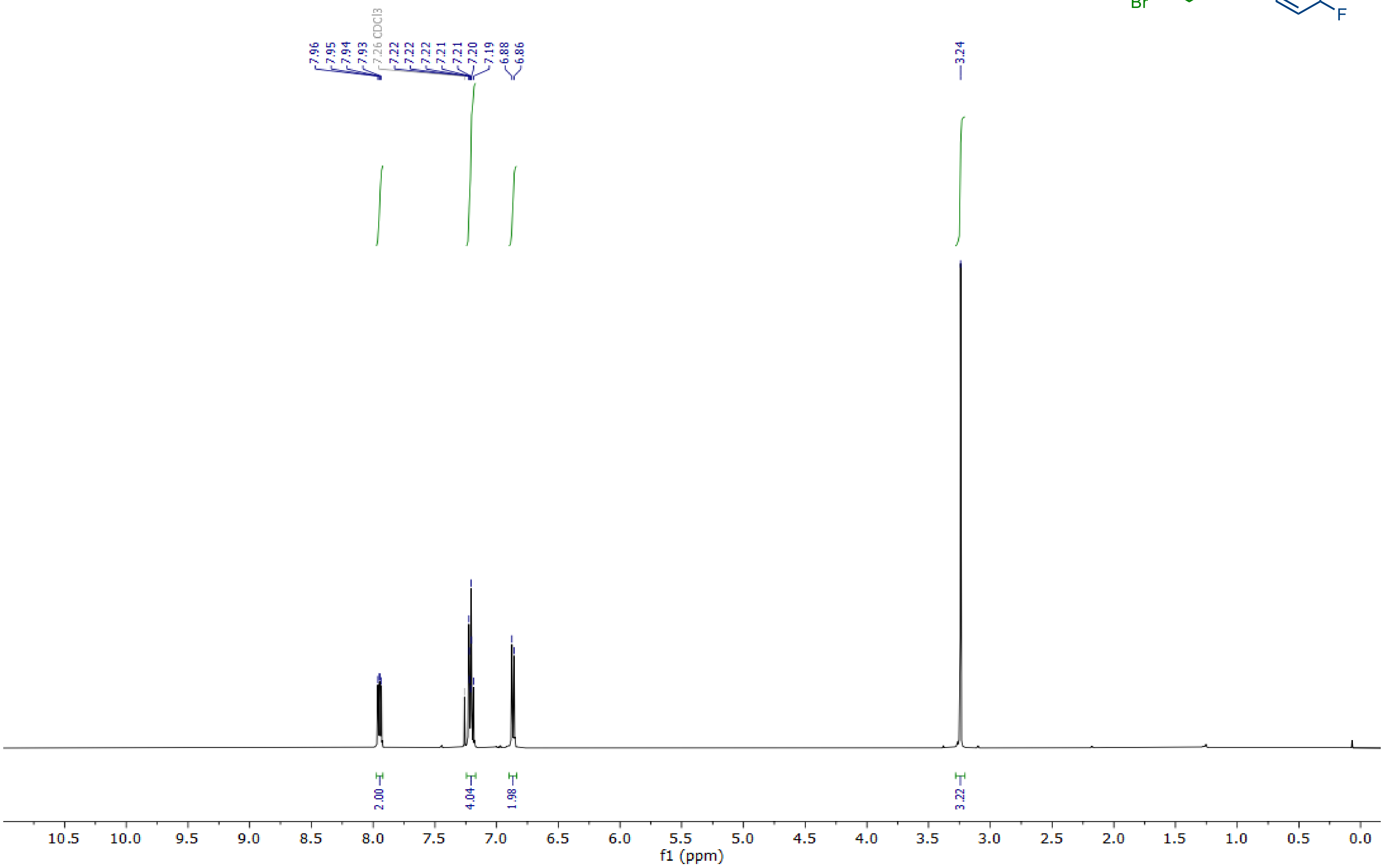

^{13}C NMR of ((4-bromophenyl)imino)(4-fluorophenyl)(methyl)-λ^6-sulfanone (20-C1)

126 MHz, $CDCl_3$, 298 K

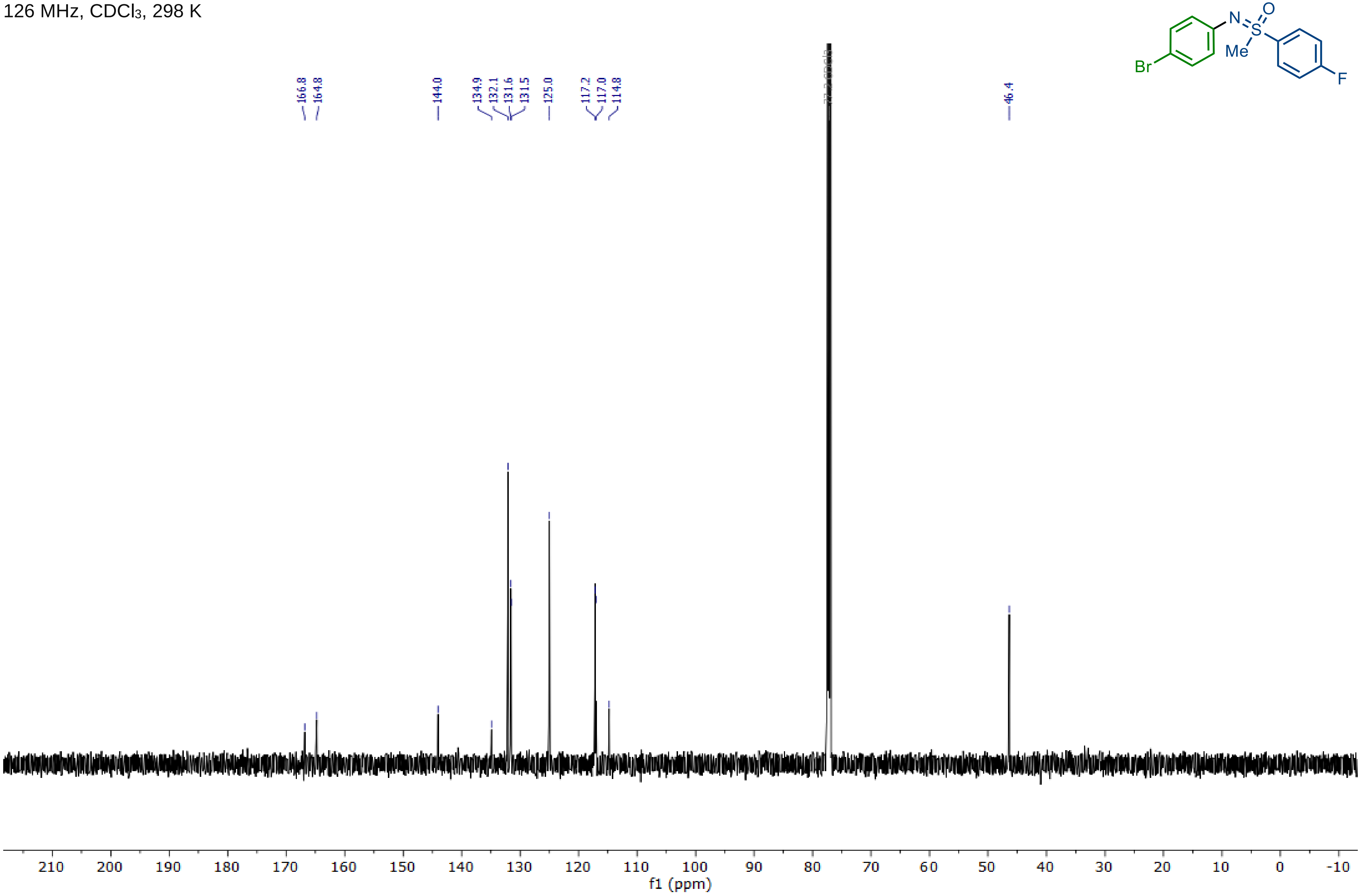

^{19}F NMR of ((4-bromophenyl)imino)(4-fluorophenyl)(methyl)-λ^6-sulfanone (20-C1)

471 MHz, $CDCl_3$, 298 K

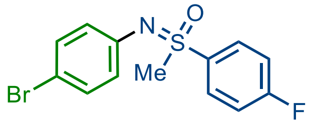

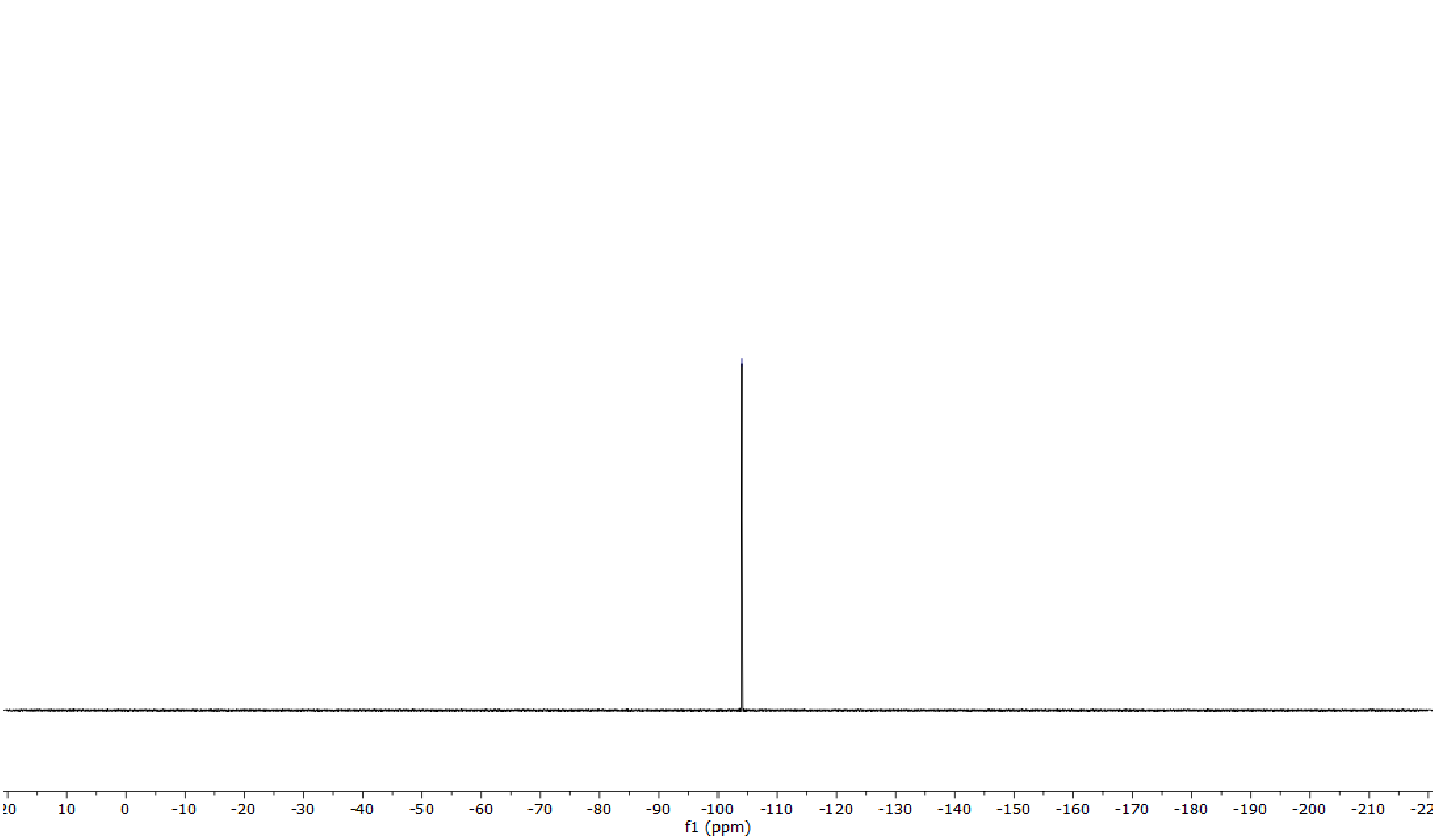

^{1}H NMR of ((2-bromophenyl)imino)(4-fluorophenyl)(methyl)-λ^{6}-sulfanone (20-C2)

500 MHz, $CDCl_3$, 298 K

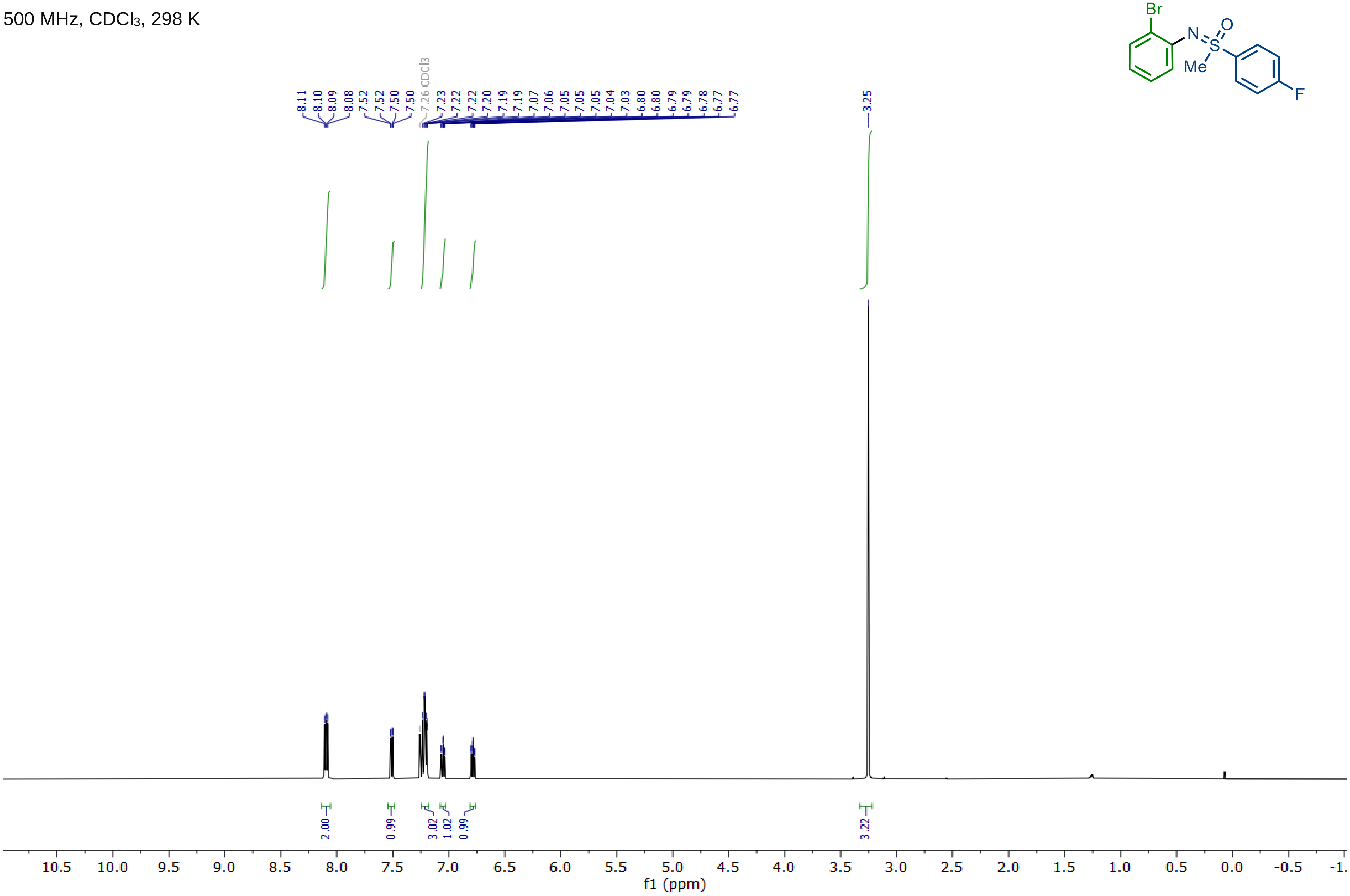

^{13}C NMR of ((2-bromophenyl)imino)(4-fluorophenyl)(methyl)-λ^6-sulfanone (20-C2)

151 MHz, $CDCl_3$, 298 K

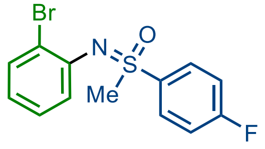

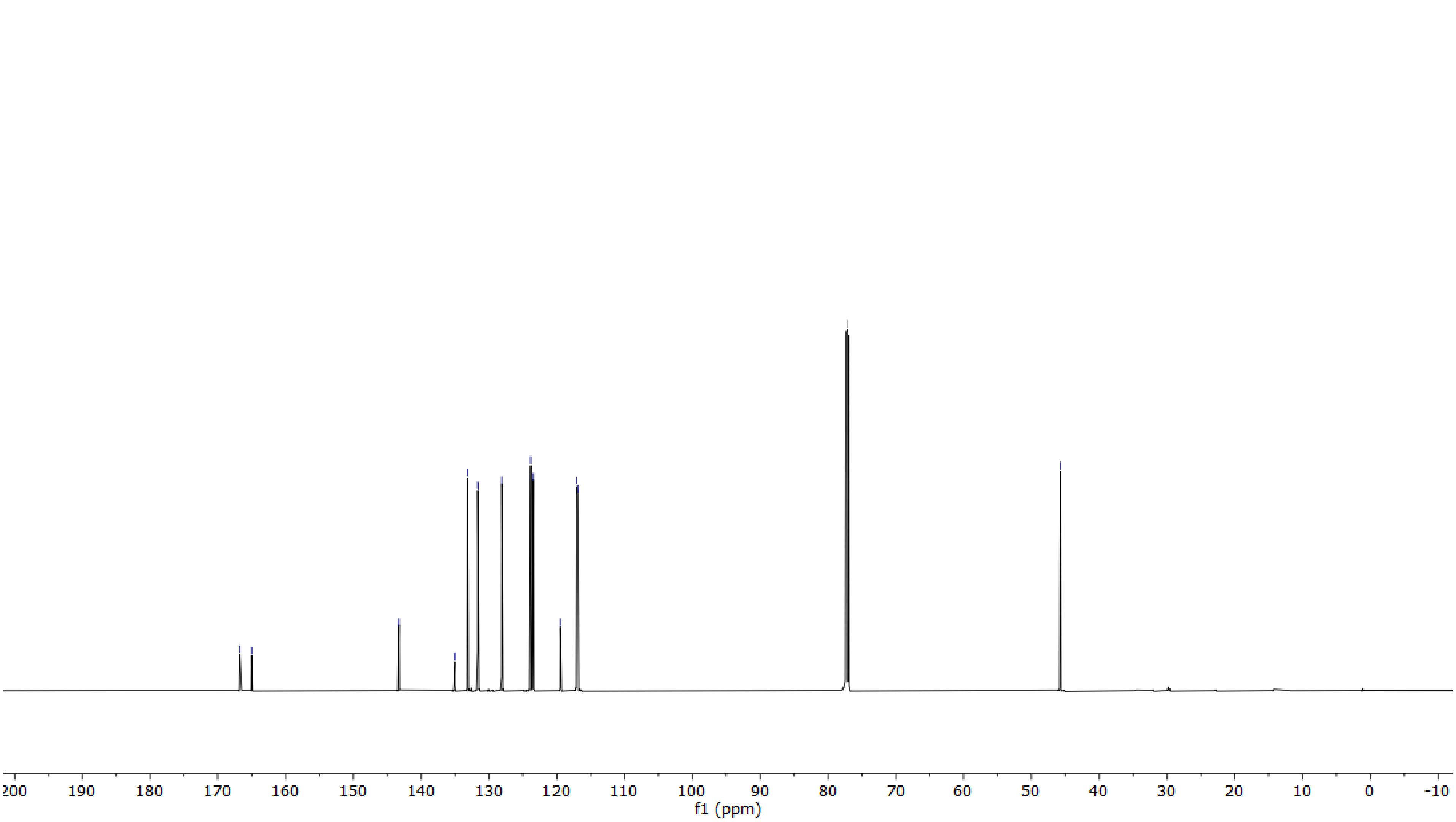

^{19}F NMR of ((2-bromophenyl)imino)(4-fluorophenyl)(methyl)-λ^{6}-sulfanone (20-C2)

471 MHz, $CDCl_3$, 298 K

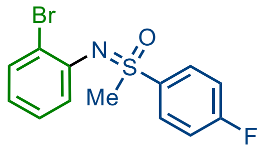

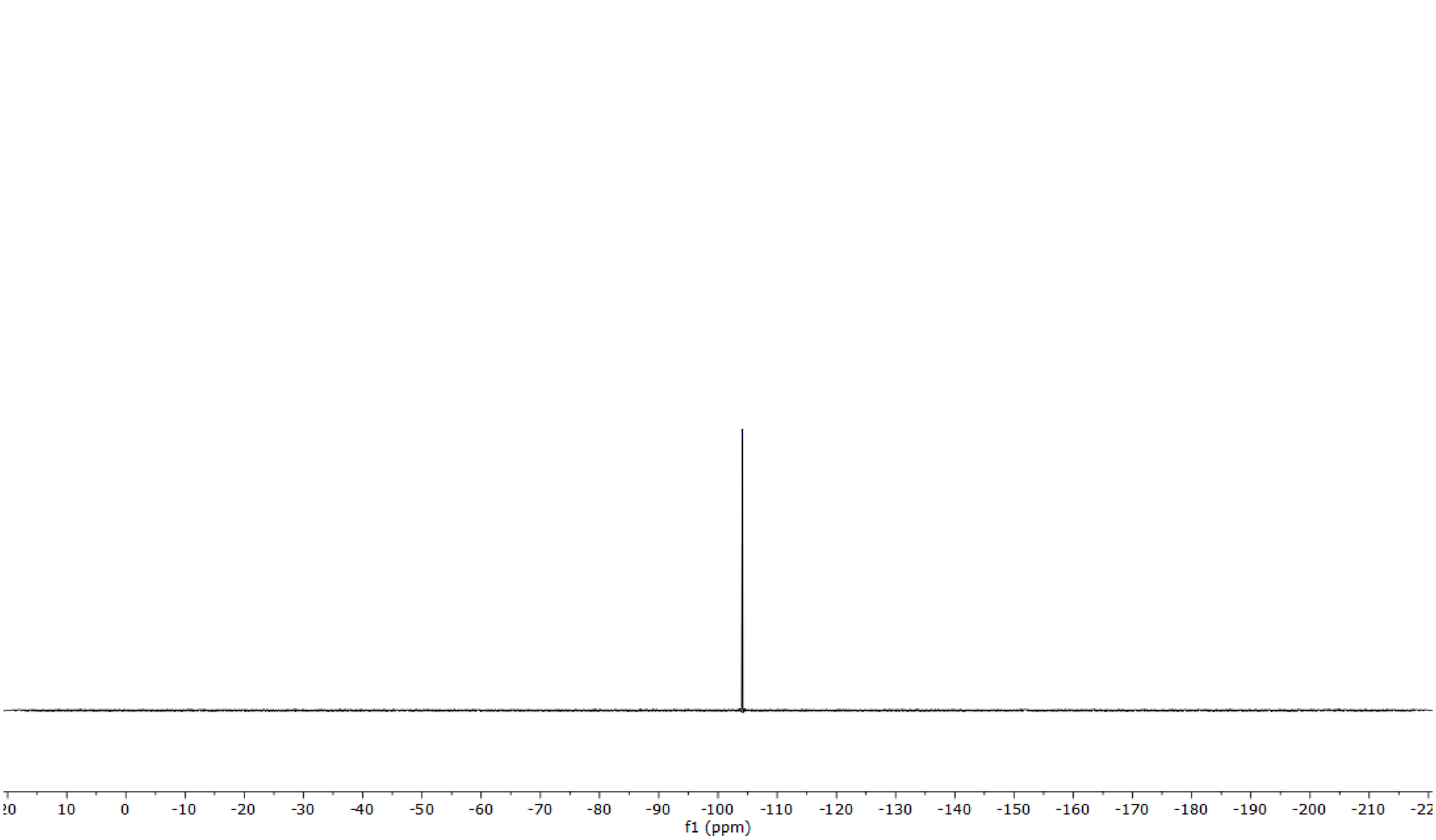

^{1}H NMR of ((3-bromophenyl)imino)(4-fluorophenyl)(methyl)-λ[6]-sulfanone (20-C3)

500 MHz, $CDCl_3$, 298 K

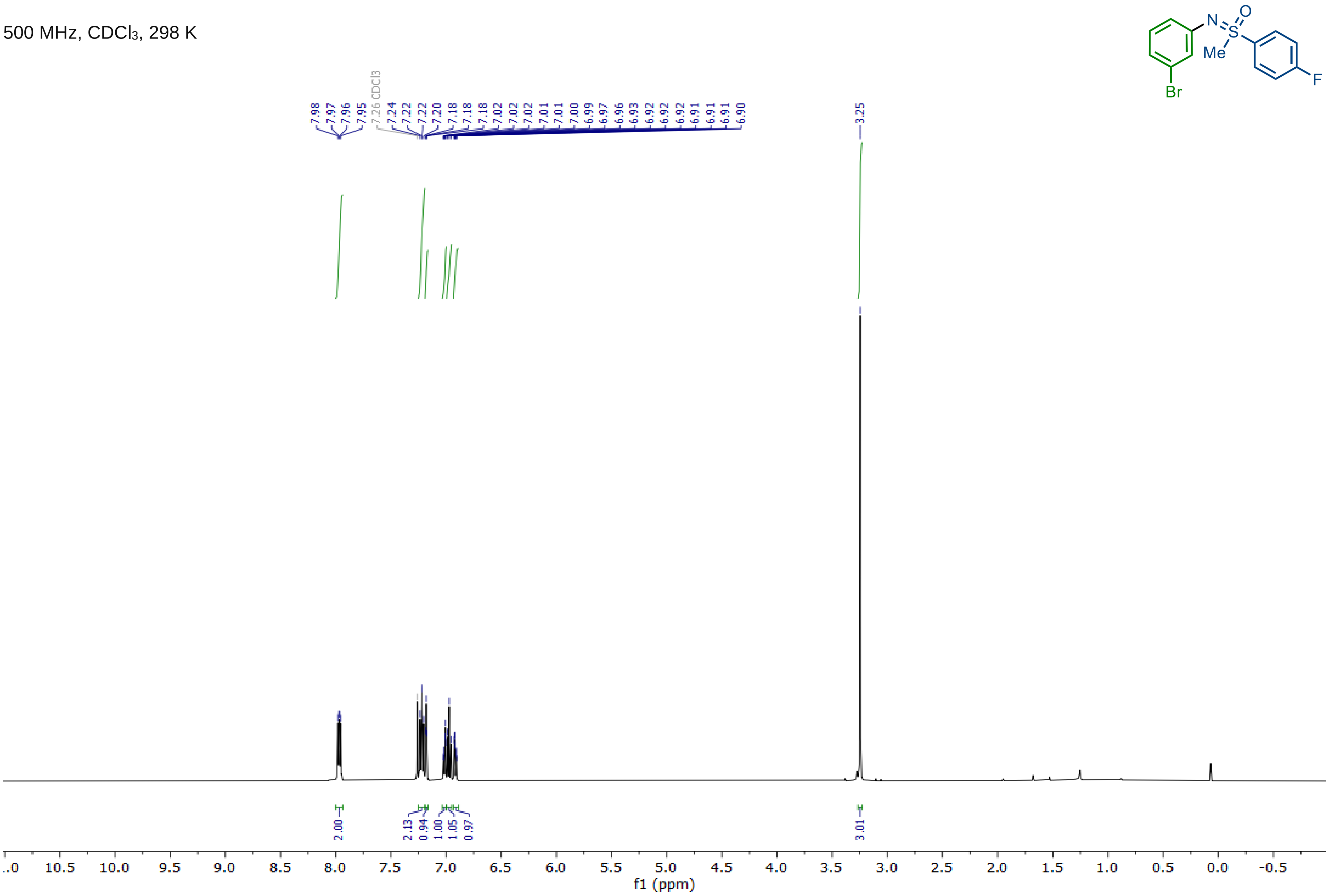

^{13}C NMR of ((3-bromophenyl)imino)(4-fluorophenyl)(methyl)-λ^6-sulfanone (20-C3)

151 MHz, $CDCl_3$, 298 K

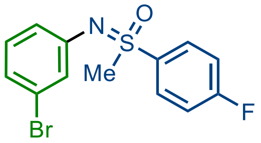

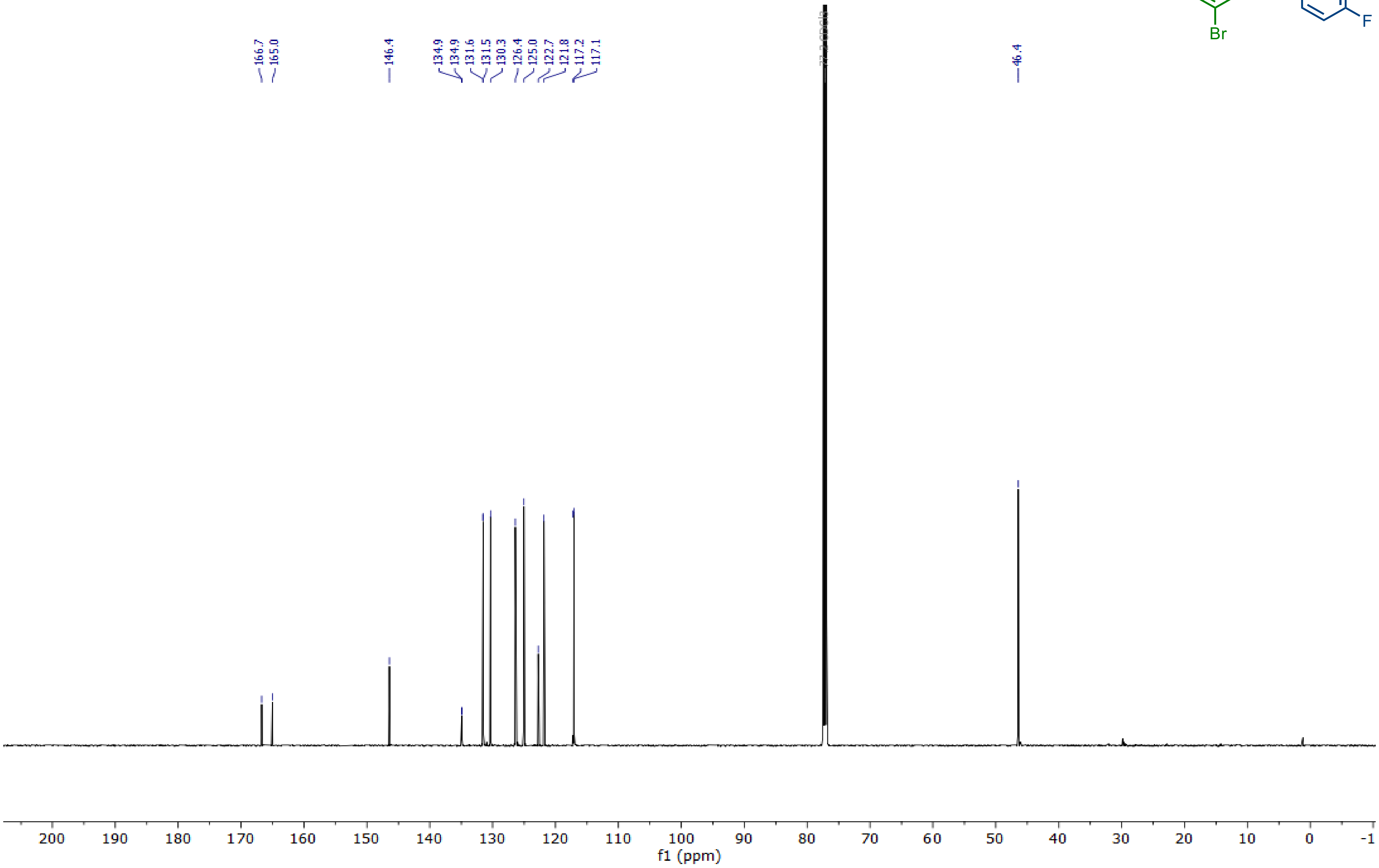

^{19}F NMR of ((3-bromophenyl)imino)(4-fluorophenyl)(methyl)-λ^{6}-sulfanone (20-C3)

471 MHz, $CDCl_3$, 298 K

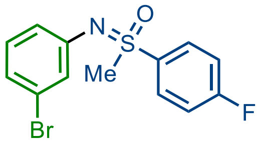

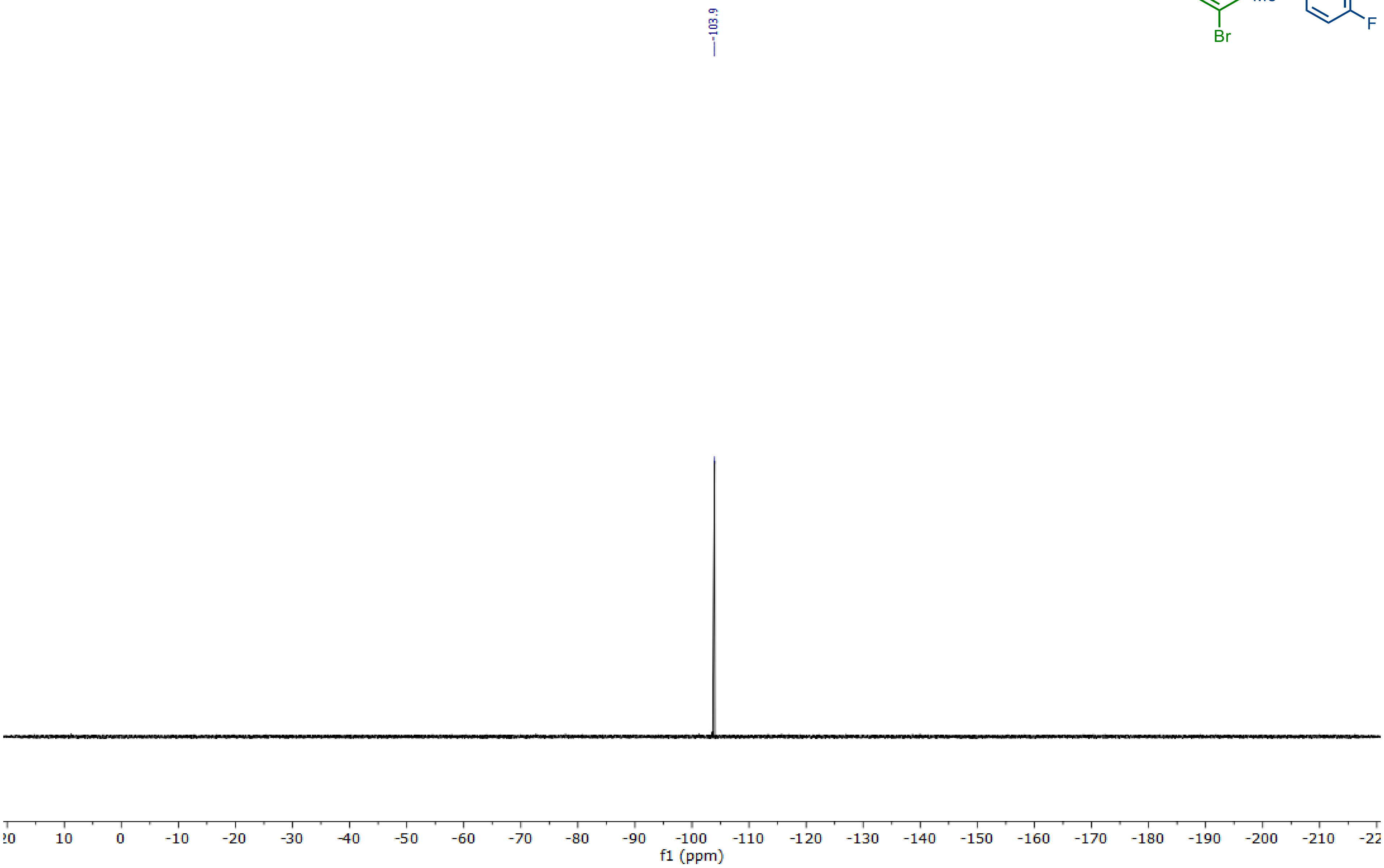

^{1}H NMR of (4-fluorophenyl)(methyl)((3-(trifluoromethyl)phenyl)imino)-λ[6]-sulfanone (21-C1)

500 MHz, $CDCl_3$, 298 K

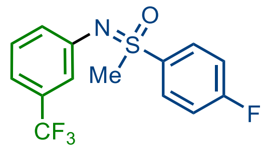

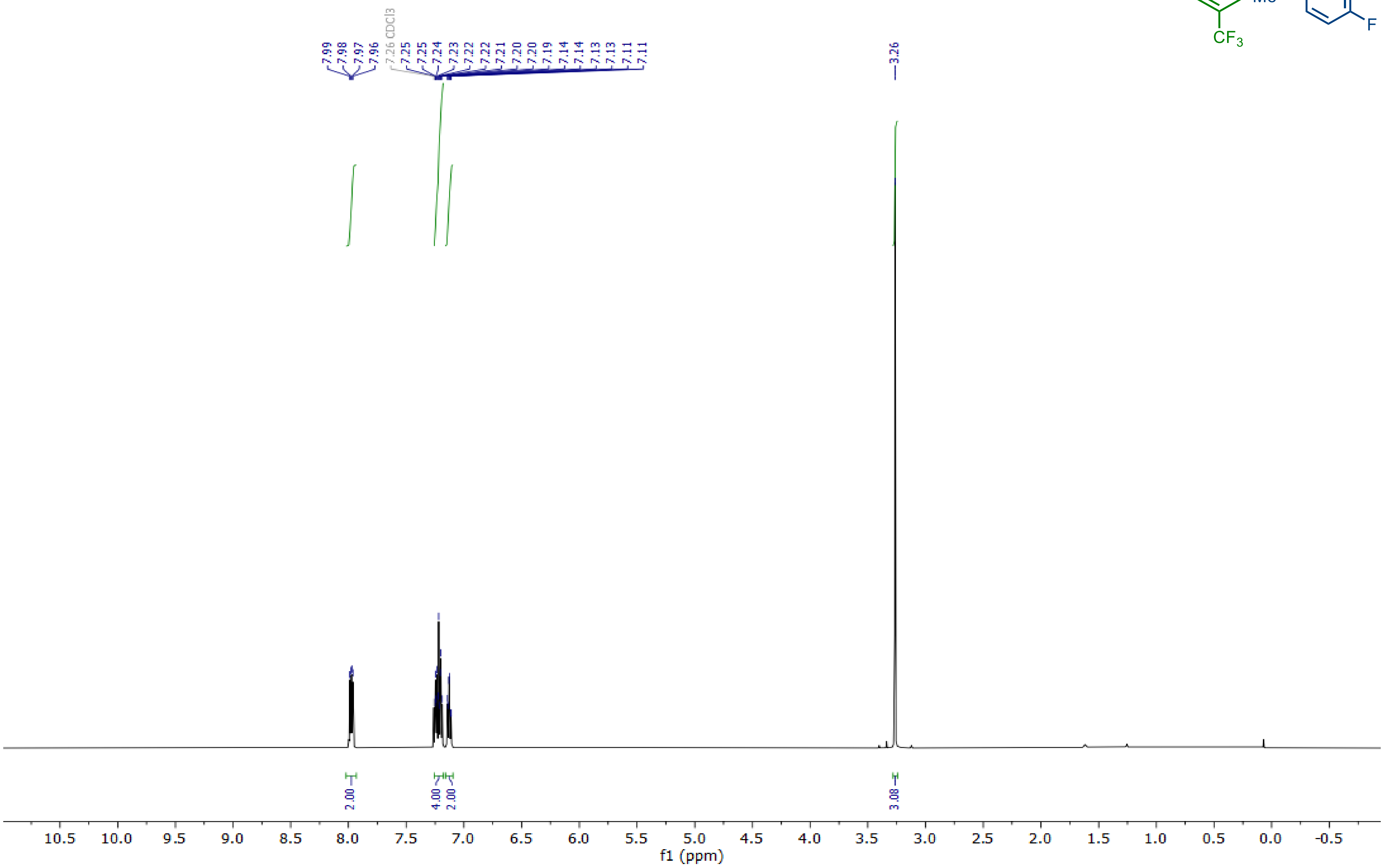

^{13}C NMR of (4-fluorophenyl)(methyl)((3-(trifluoromethyl)phenyl)imino)-λ^6-sulfanone (21-C1)

126 MHz, $CDCl_3$, 298 K

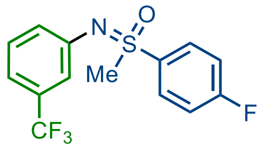

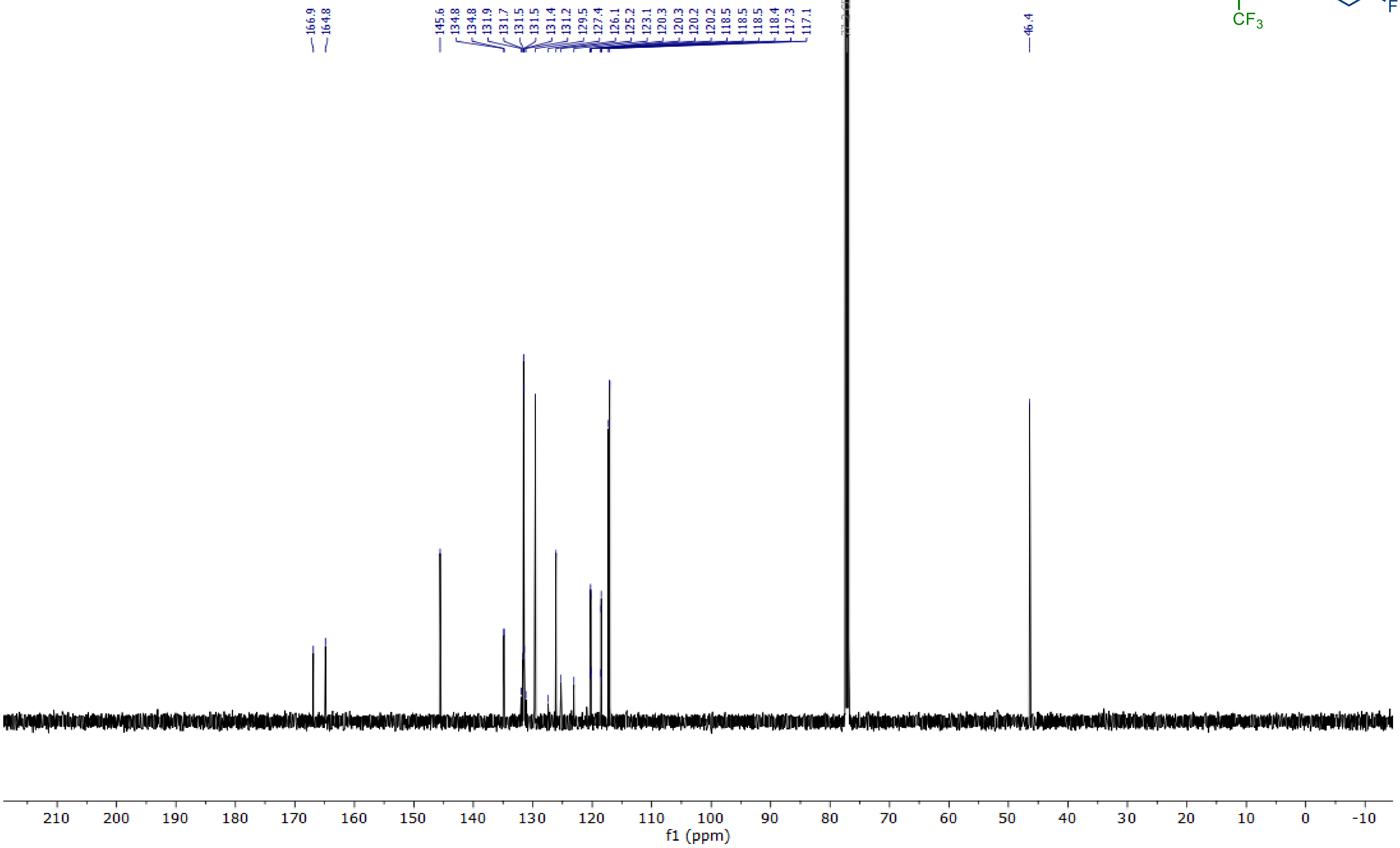

^{19}F NMR of (4-fluorophenyl)(methyl)((3-(trifluoromethyl)phenyl)imino)-λ[6]-sulfanone (21-C1)

471 MHz, $CDCl_3$, 298 K

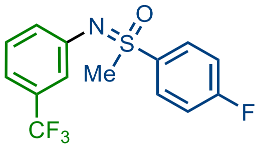

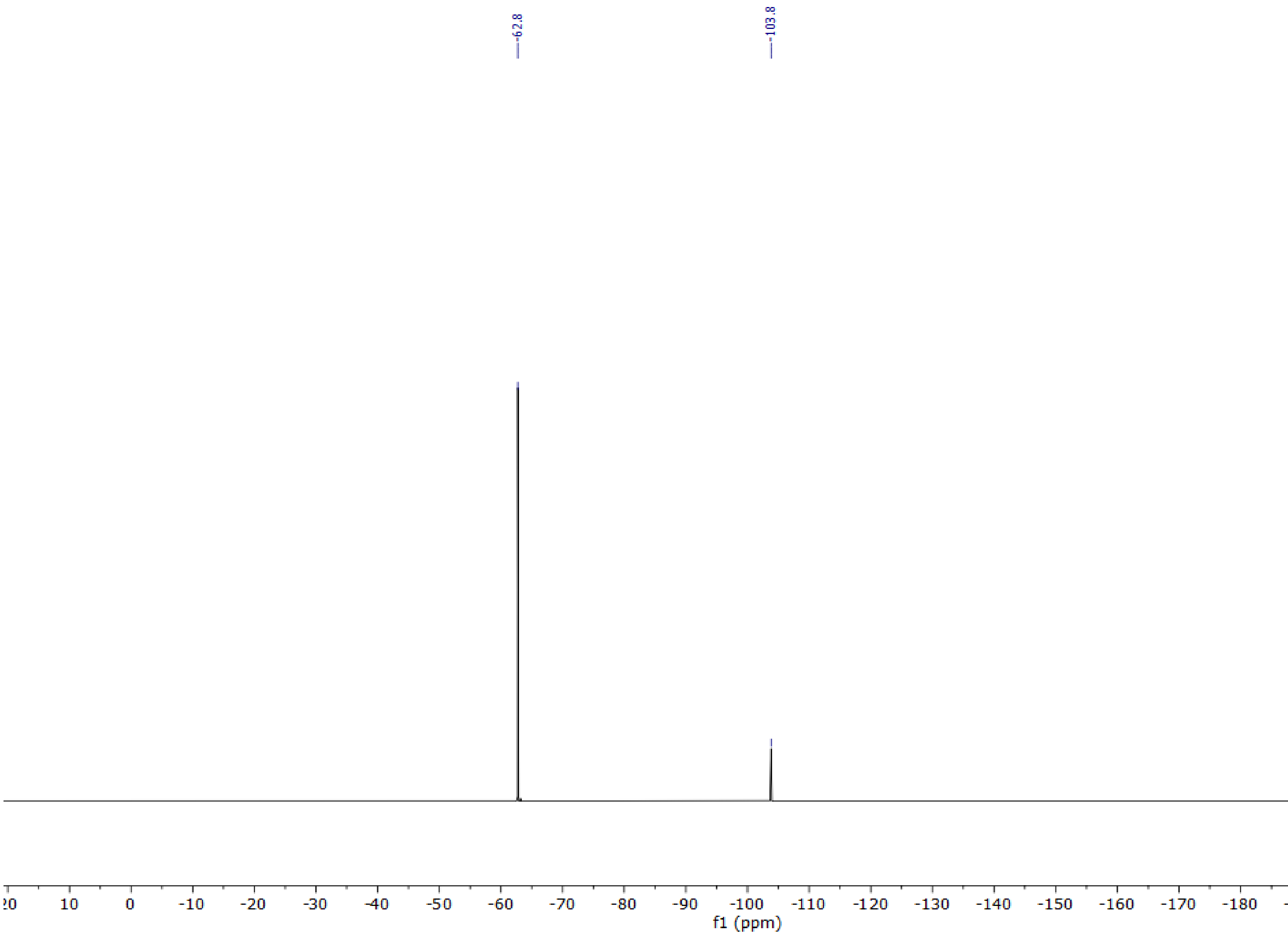

^{1}H NMR of (4-fluorophenyl)(methyl)((4-(trifluoromethyl)phenyl)imino)-λ^6-sulfanone (21-C2)

500 MHz, $CDCl_3$, 298 K

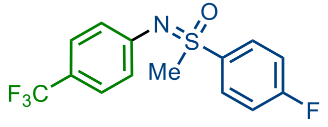

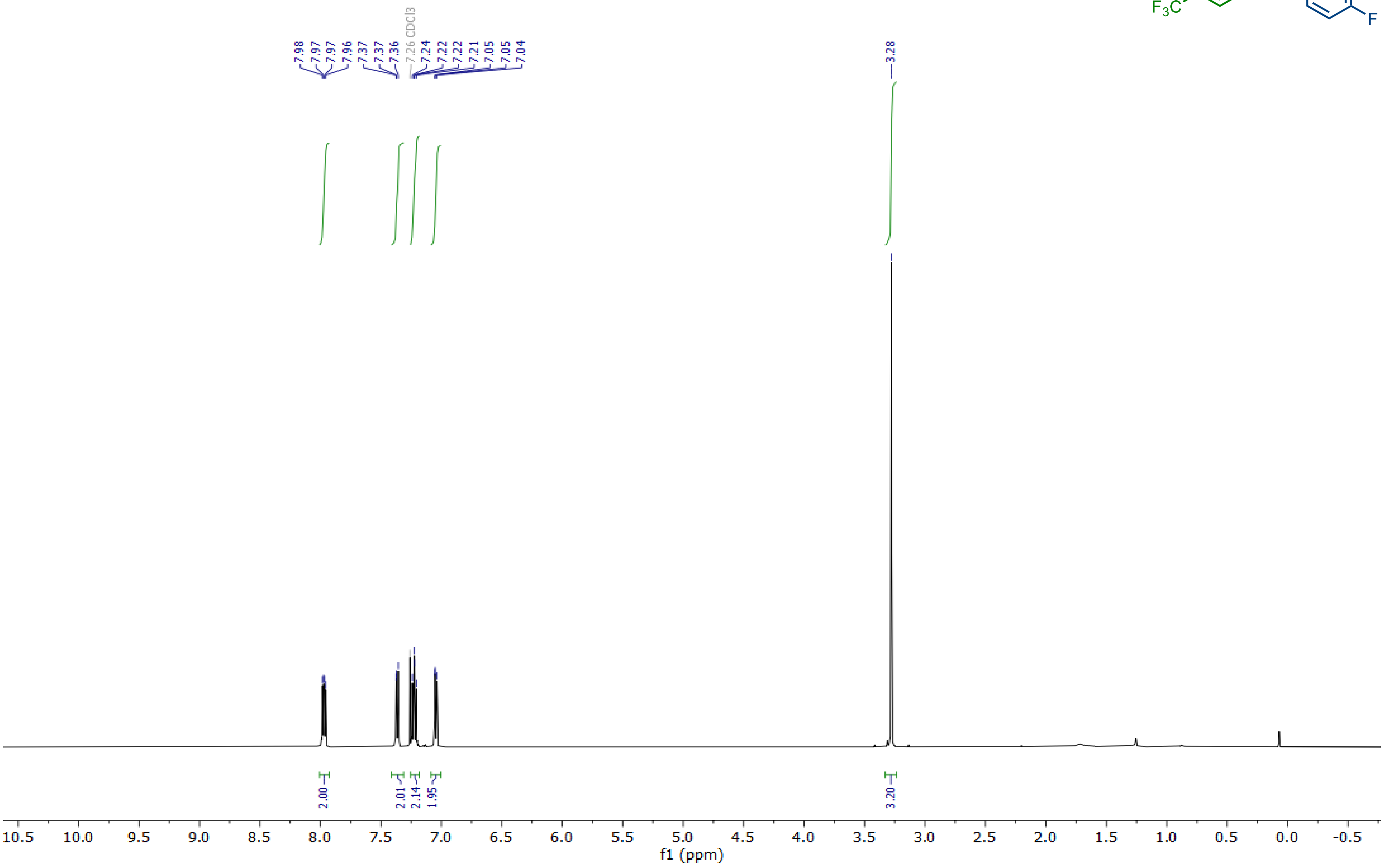

^{13}C NMR of (4-fluorophenyl)(methyl)((4-(trifluoromethyl)phenyl)imino)-λ^6-sulfanone (21-C2)

126 MHz, $CDCl_3$, 298 K

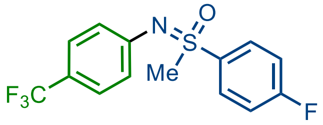

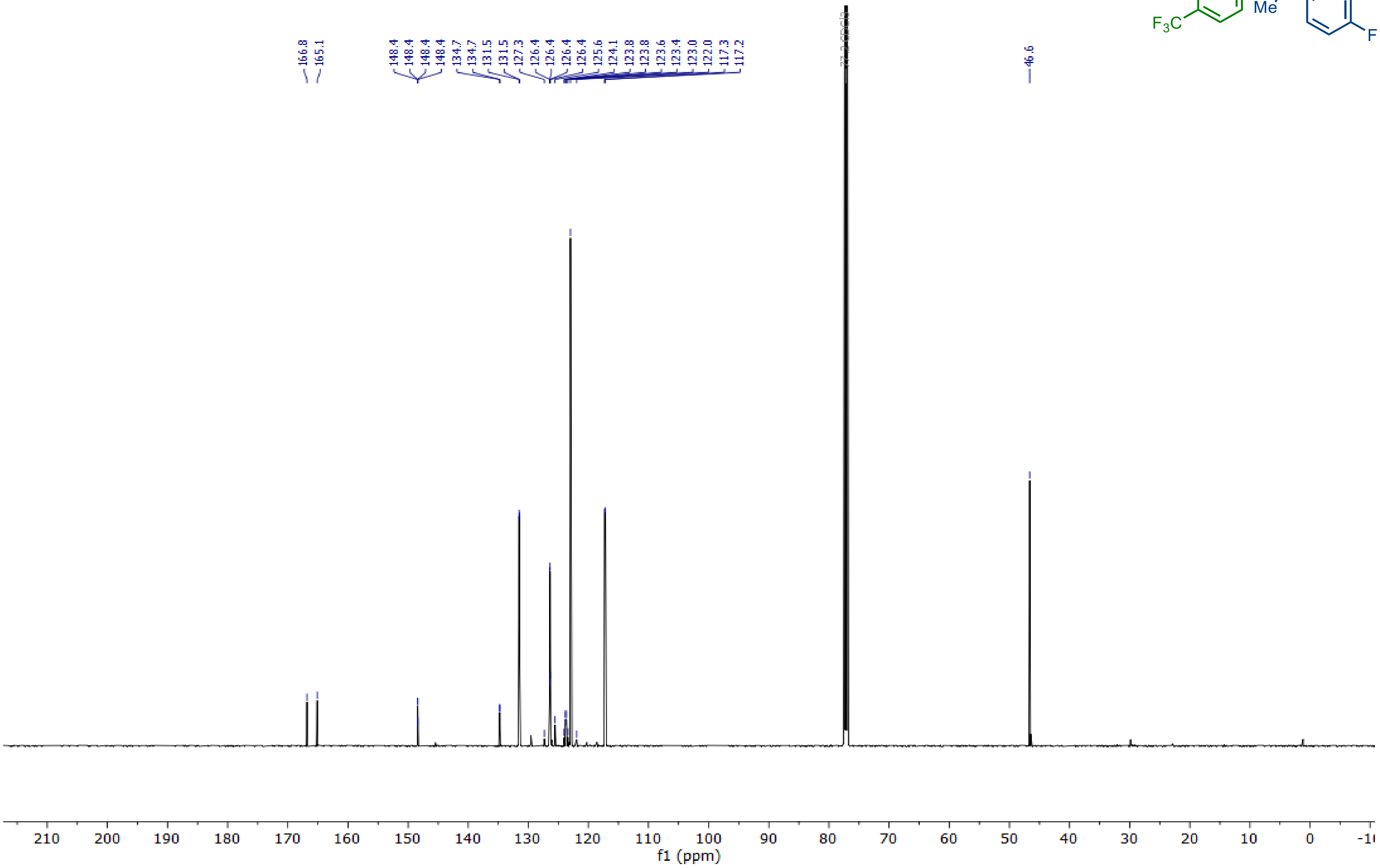

^{19}F NMR of (4-fluorophenyl)(methyl)((4-(trifluoromethyl)phenyl)imino)-λ^6-sulfanone (21-C2)

471 MHz, $CDCl_3$, 298 K

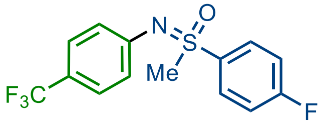

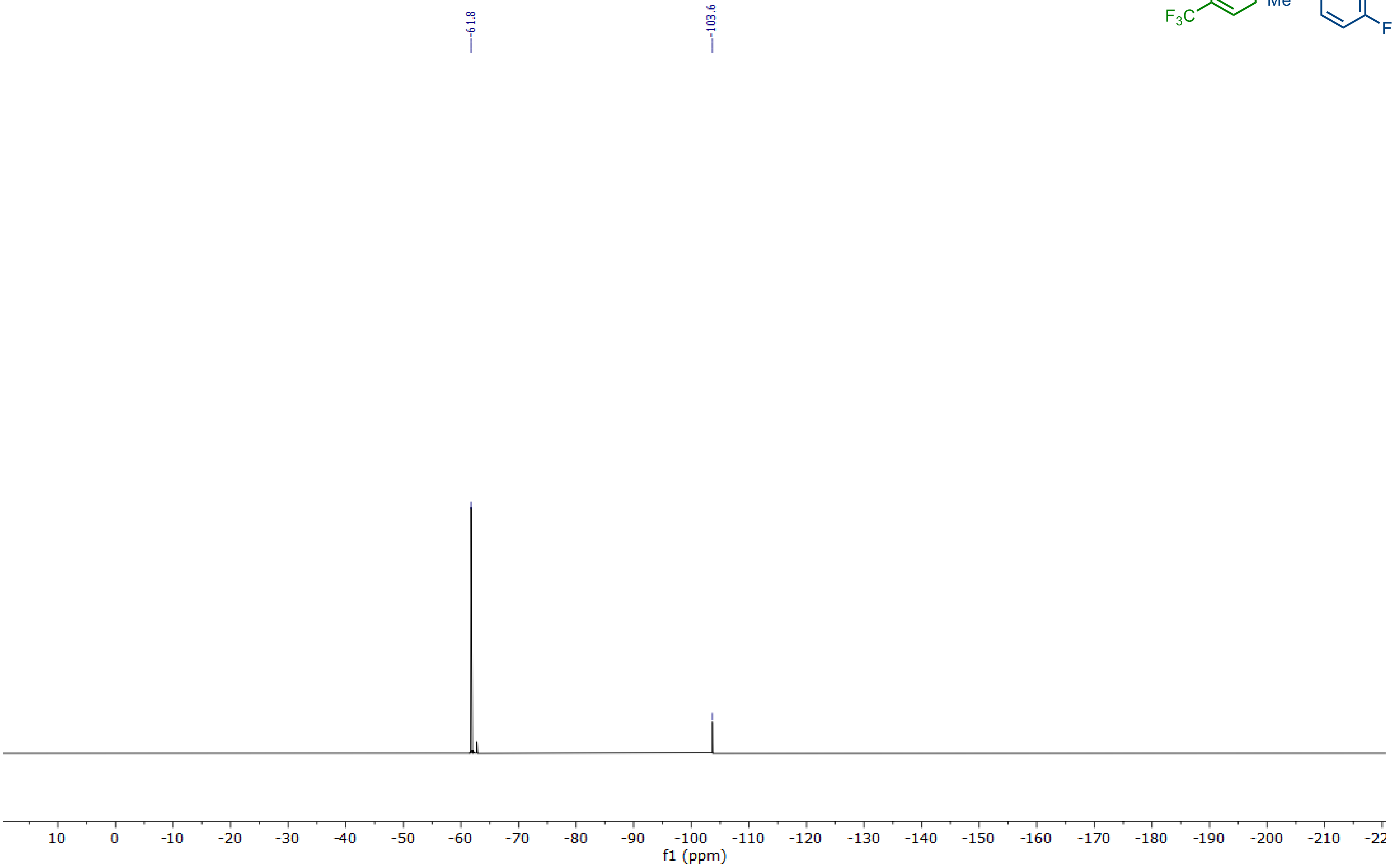

^{1}H NMR of (4-fluorophenyl)(methyl)((2-(trifluoromethyl)phenyl)imino)-λ[6]-sulfanone (21-C3)

500 MHz, $CDCl_3$, 298 K

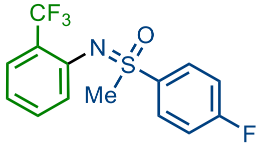

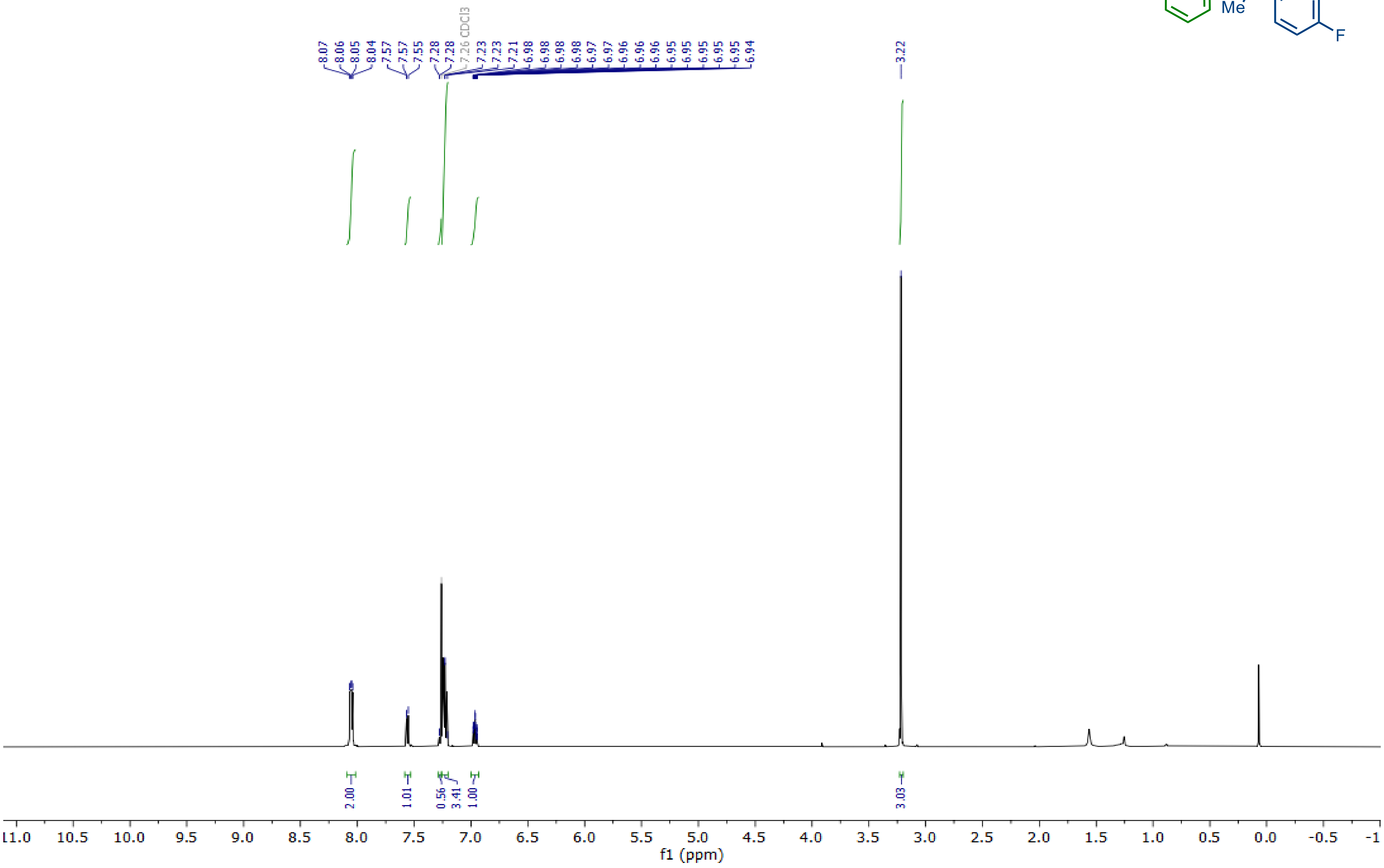

^{13}C NMR of (4-fluorophenyl)(methyl)((2-(trifluoromethyl)phenyl)imino)-λ^6-sulfanone (21-C3)

151 MHz, $CDCl_3$, 298 K

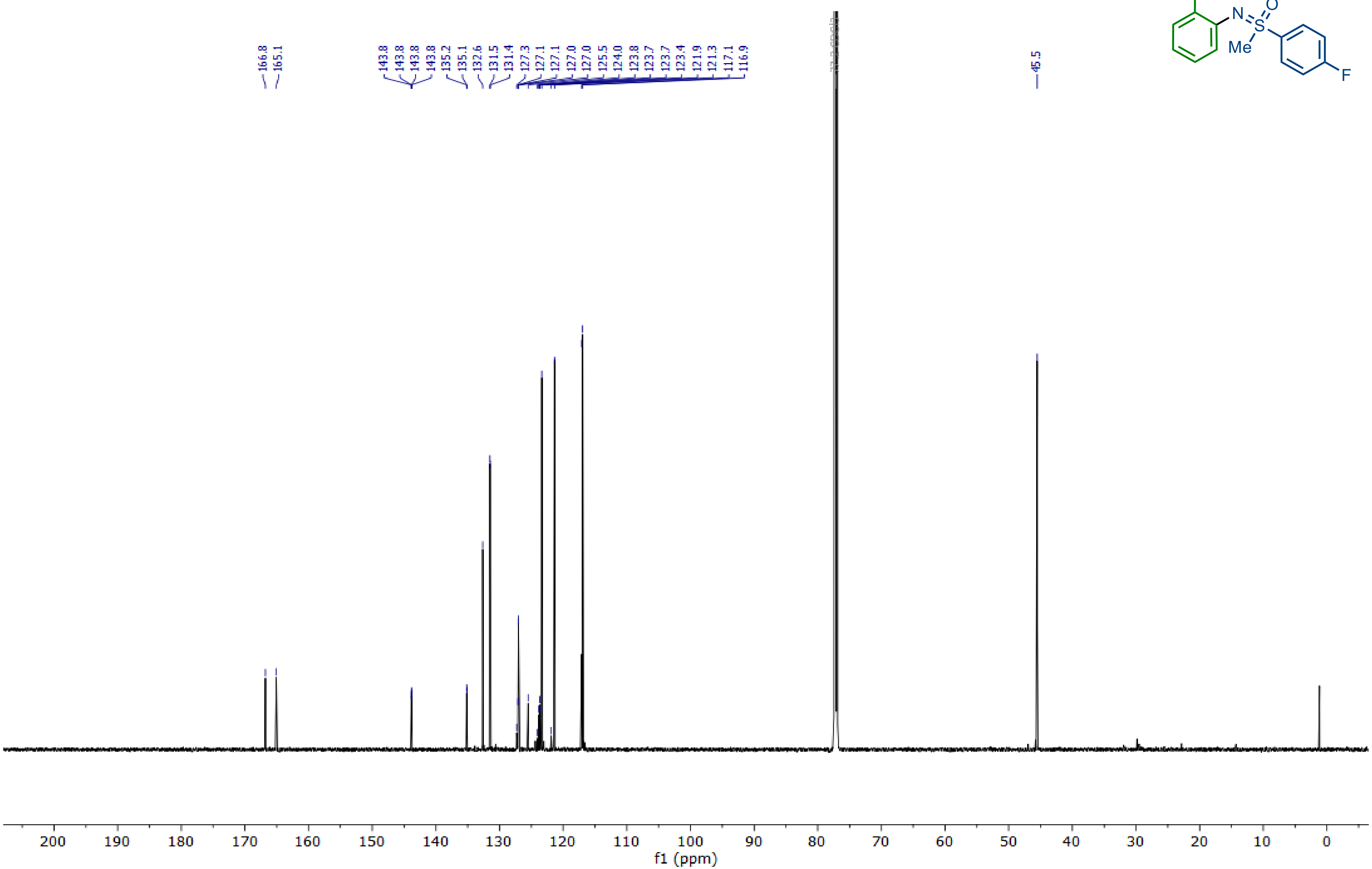

^{19}F NMR of (4-fluorophenyl)(methyl)((2-(trifluoromethyl)phenyl)imino)-λ^6-sulfanone (21-C3)

565 MHz, $CDCl_3$, 298 K

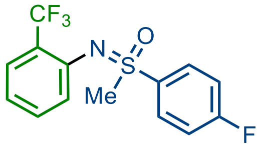

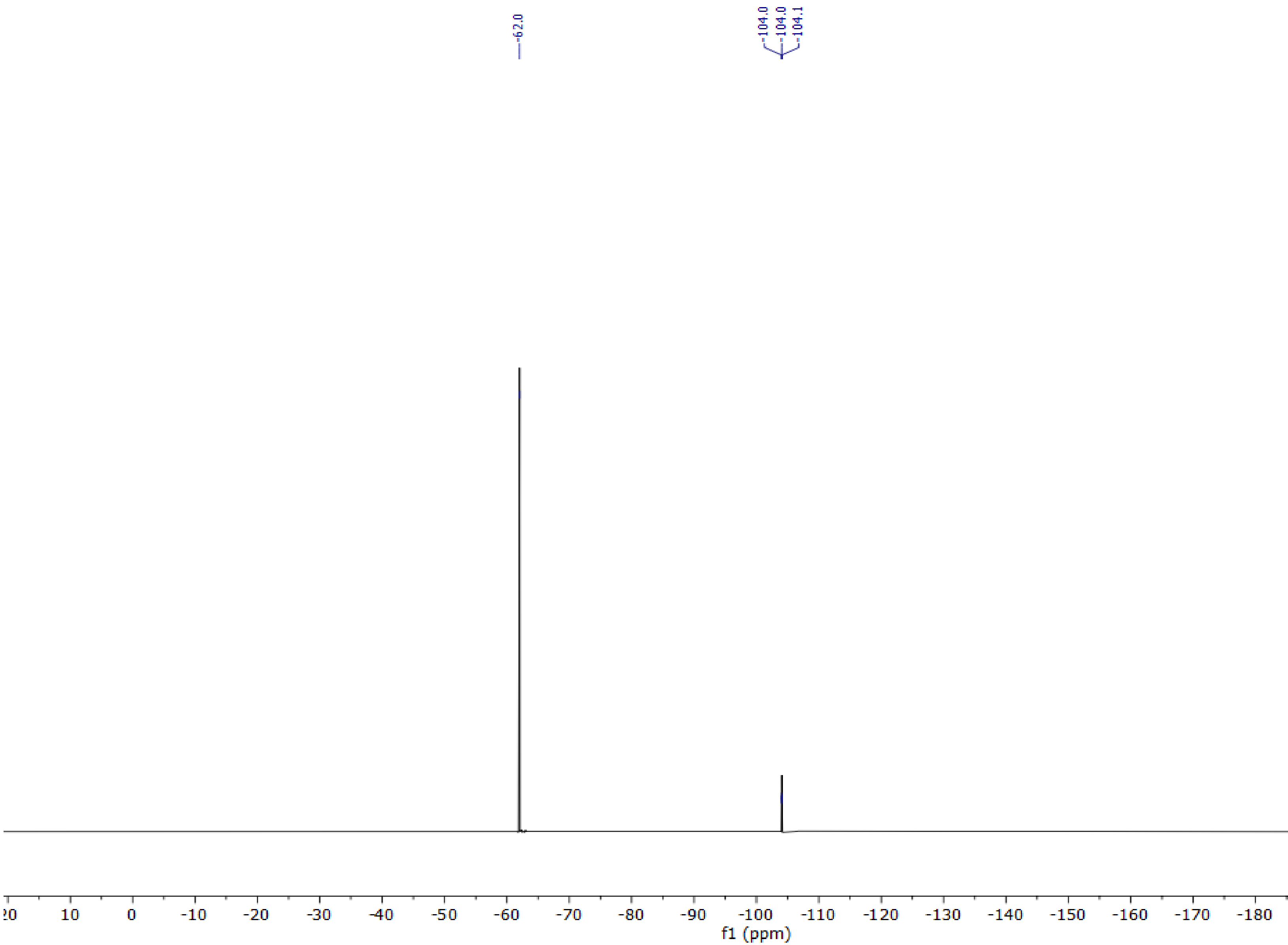

^{1}H NMR of ((2,5-difluorophenyl)imino)(4-fluorophenyl)(methyl)-λ^6-sulfanone (22)

500 MHz, $CDCl_3$, 298 K

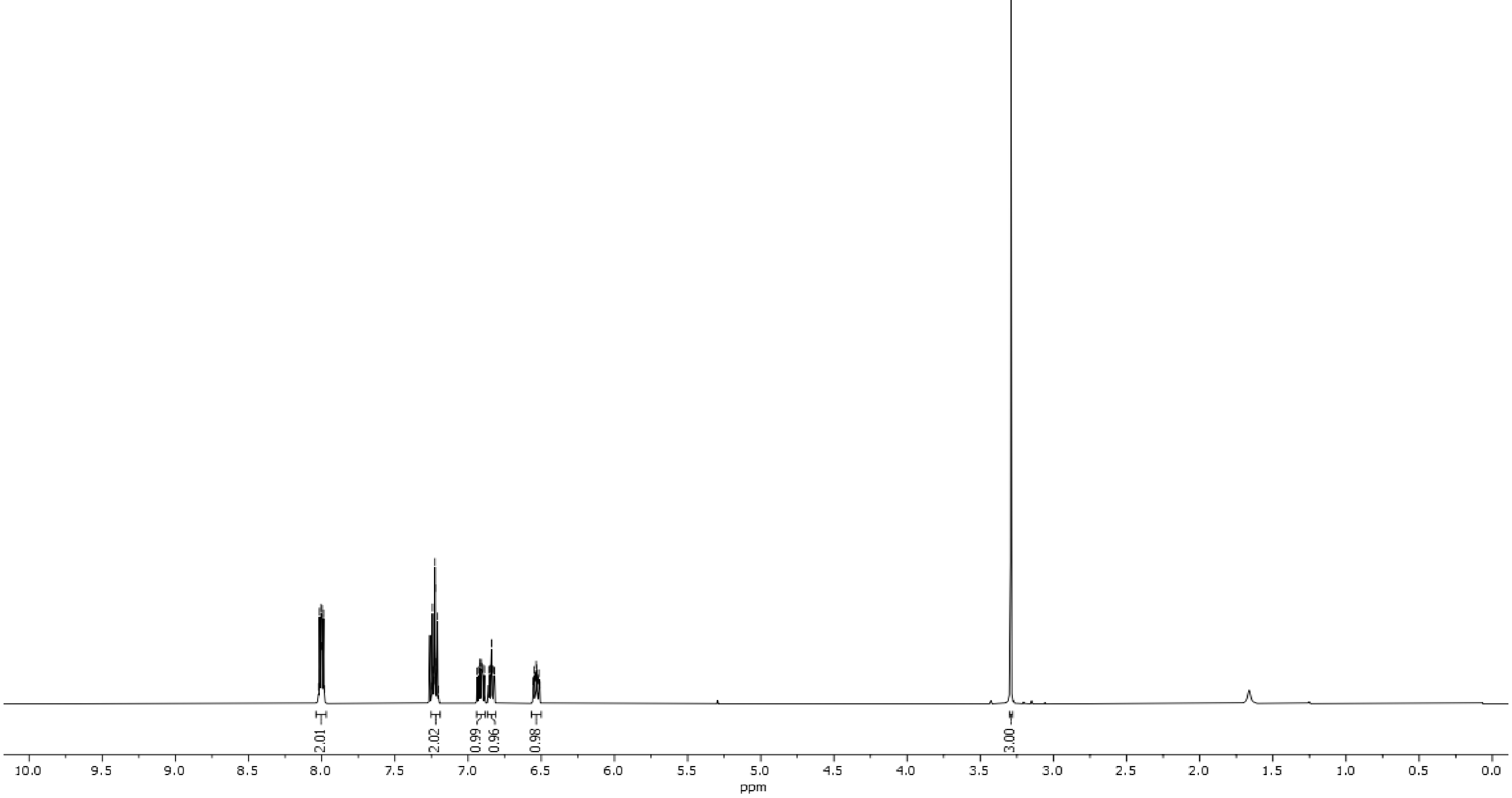

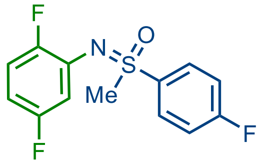

^{13}C NMR of ((2,5-difluorophenyl)imino)(4-fluorophenyl)(methyl)-λ^6-sulfanone (22)

126 MHz, $CDCl_3$, 298 K

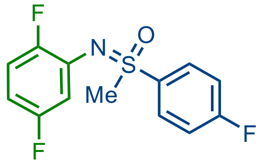

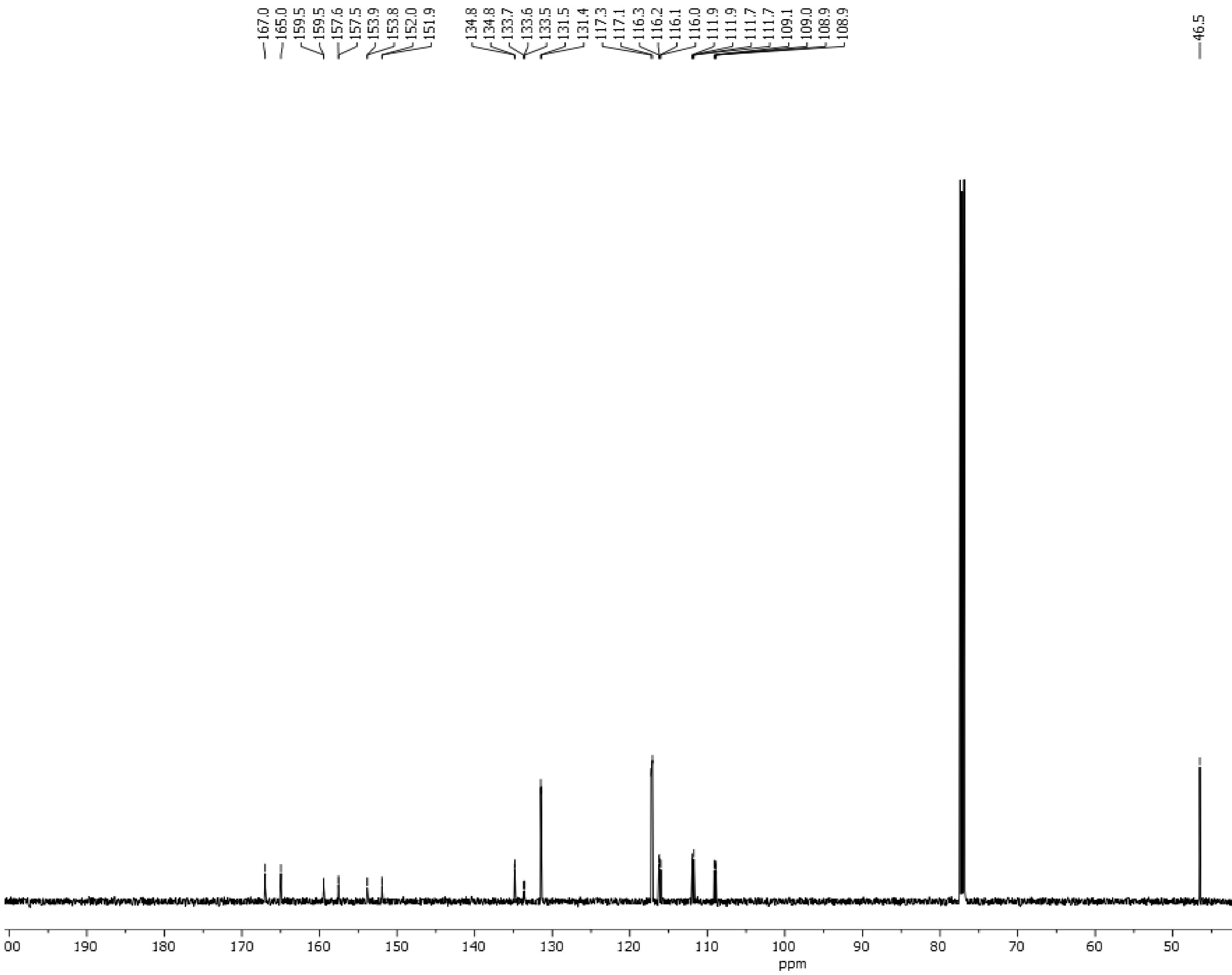

^{19}F NMR of ((2,5-difluorophenyl)imino)(4-fluorophenyl)(methyl)-λ[6]-sulfanone (22)

471 MHz, $CDCl_3$, 298 K

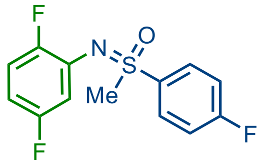

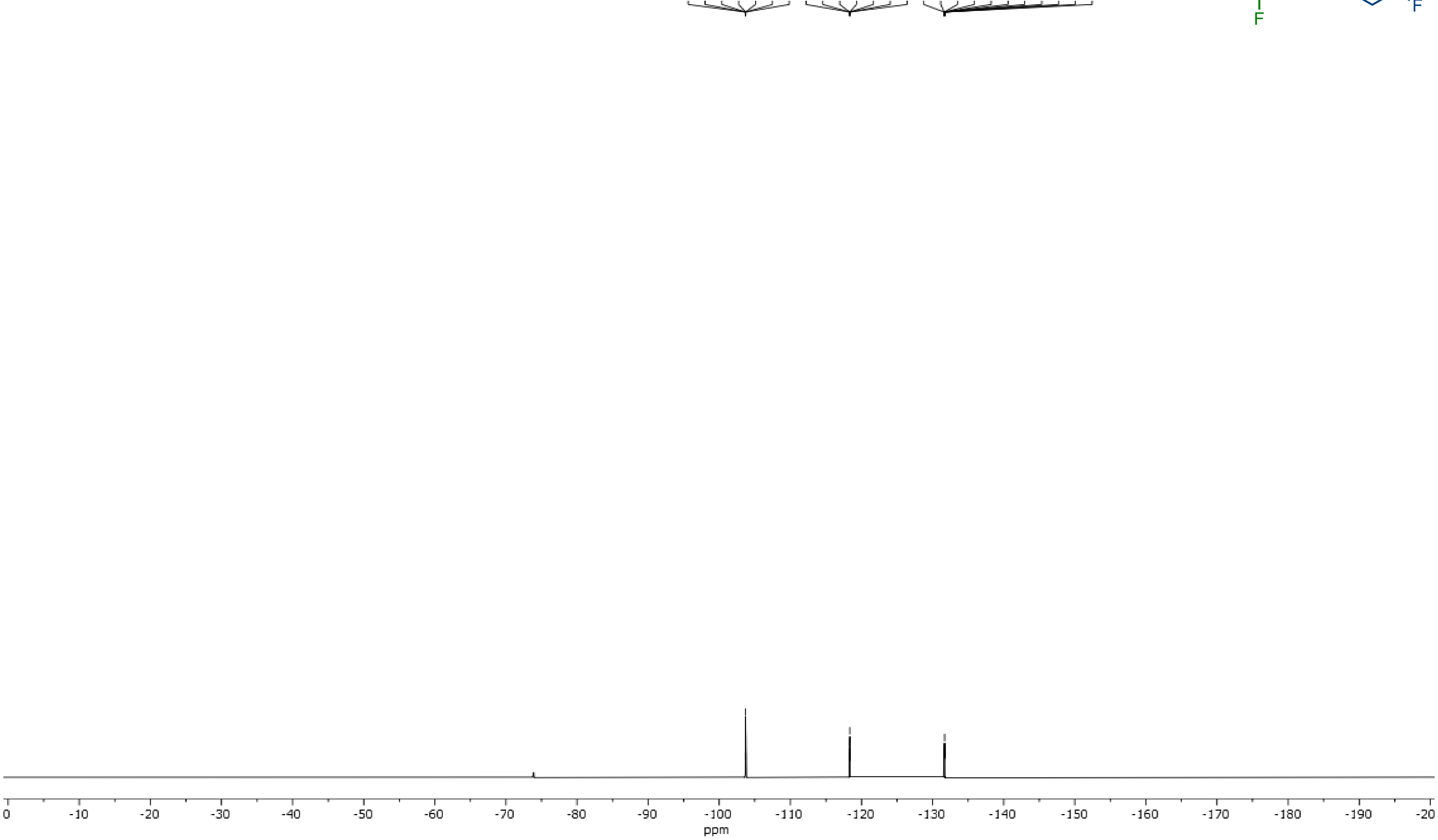

^{1}H NMR of methyl(phenylimino)(thiophen-2-yl)-λ^6-sulfanone (23)

500 MHz, $CDCl_3$, 298 K

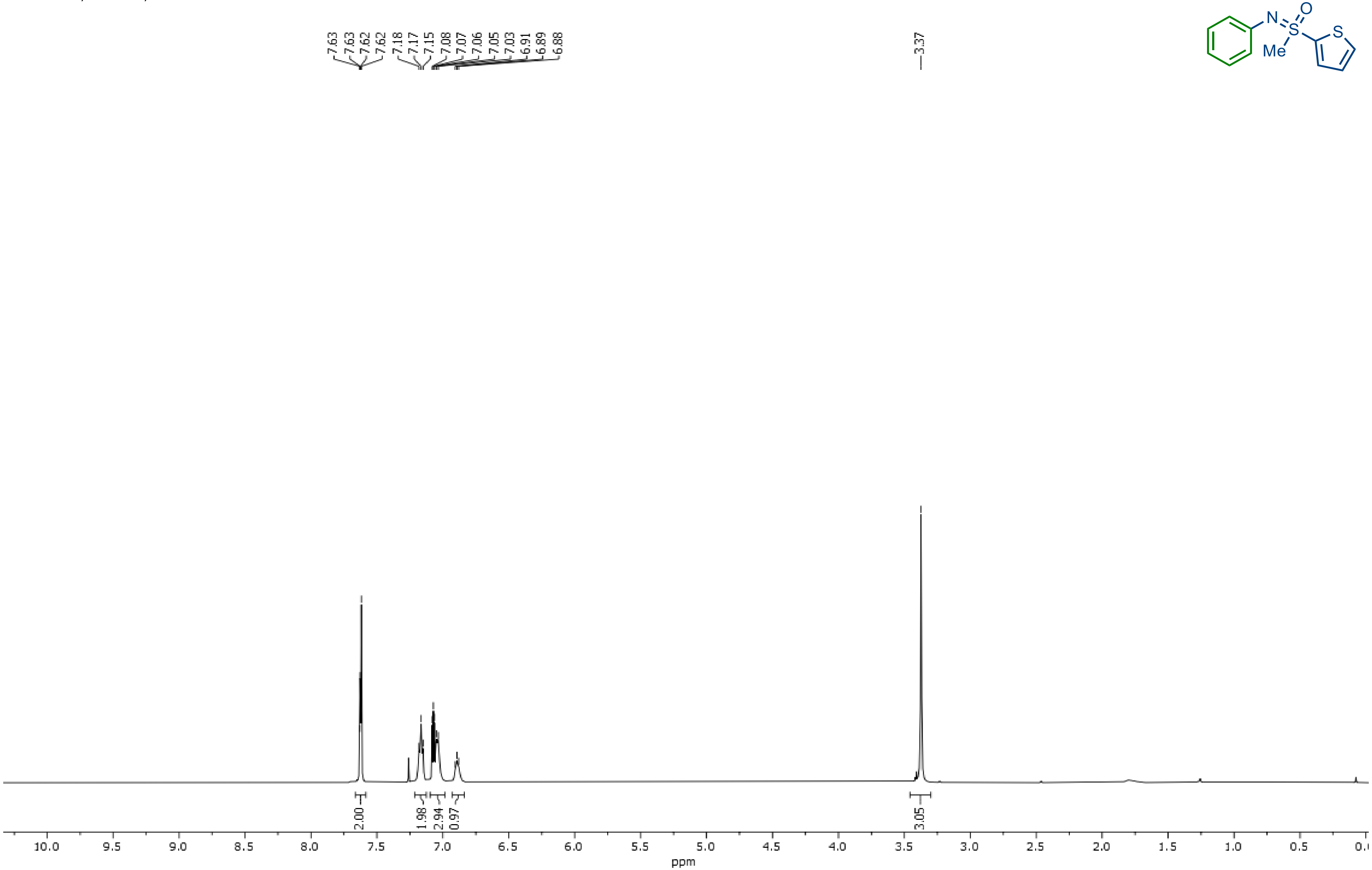

^{13}C NMR of methyl(phenylimino)(thiophen-2-yl)-λ^6-sulfanone (23)

126 MHz, $CDCl_3$, 298 K

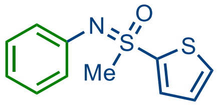

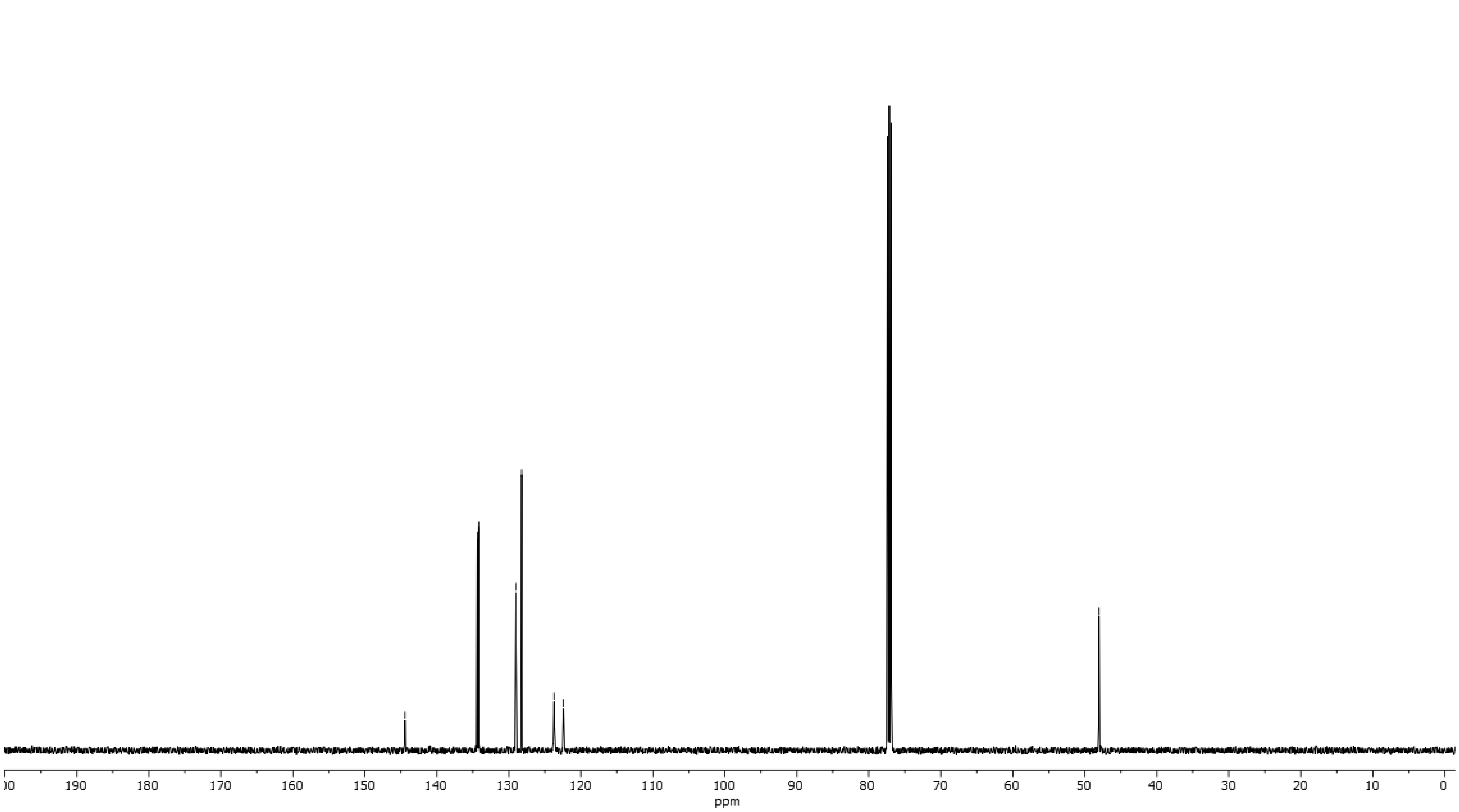

^{1}H NMR of ((2,5-dichlorophenyl)imino)diphenyl-λ[6]-sulfanone (24)

500 MHz, $CDCl_3$, 298 K

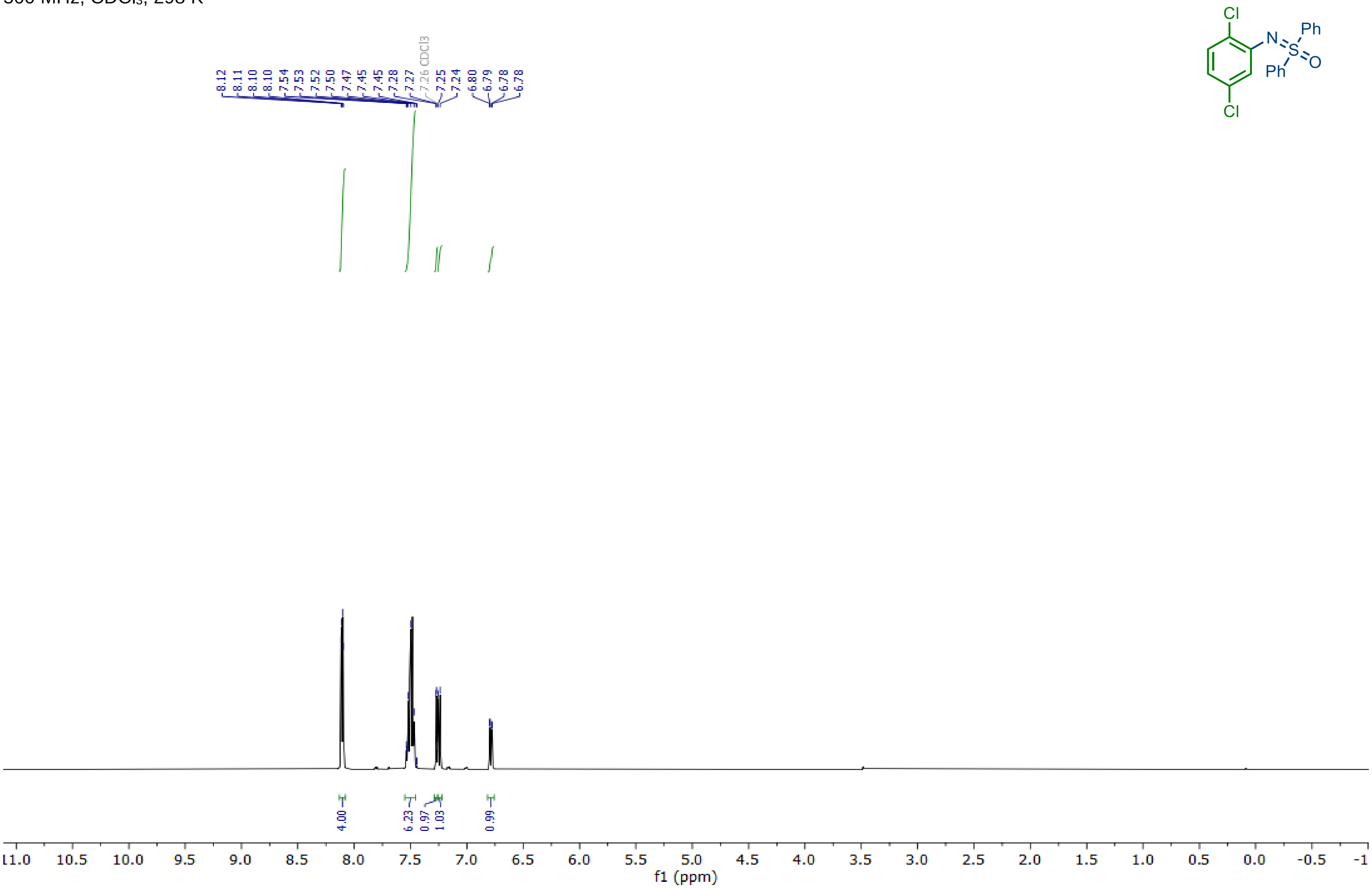

^{13}C NMR of ((2,5-dichlorophenyl)imino)diphenyl-λ^6-sulfanone (24)

126 MHz, $CDCl_3$, 298 K

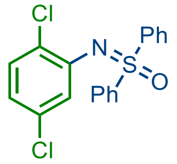

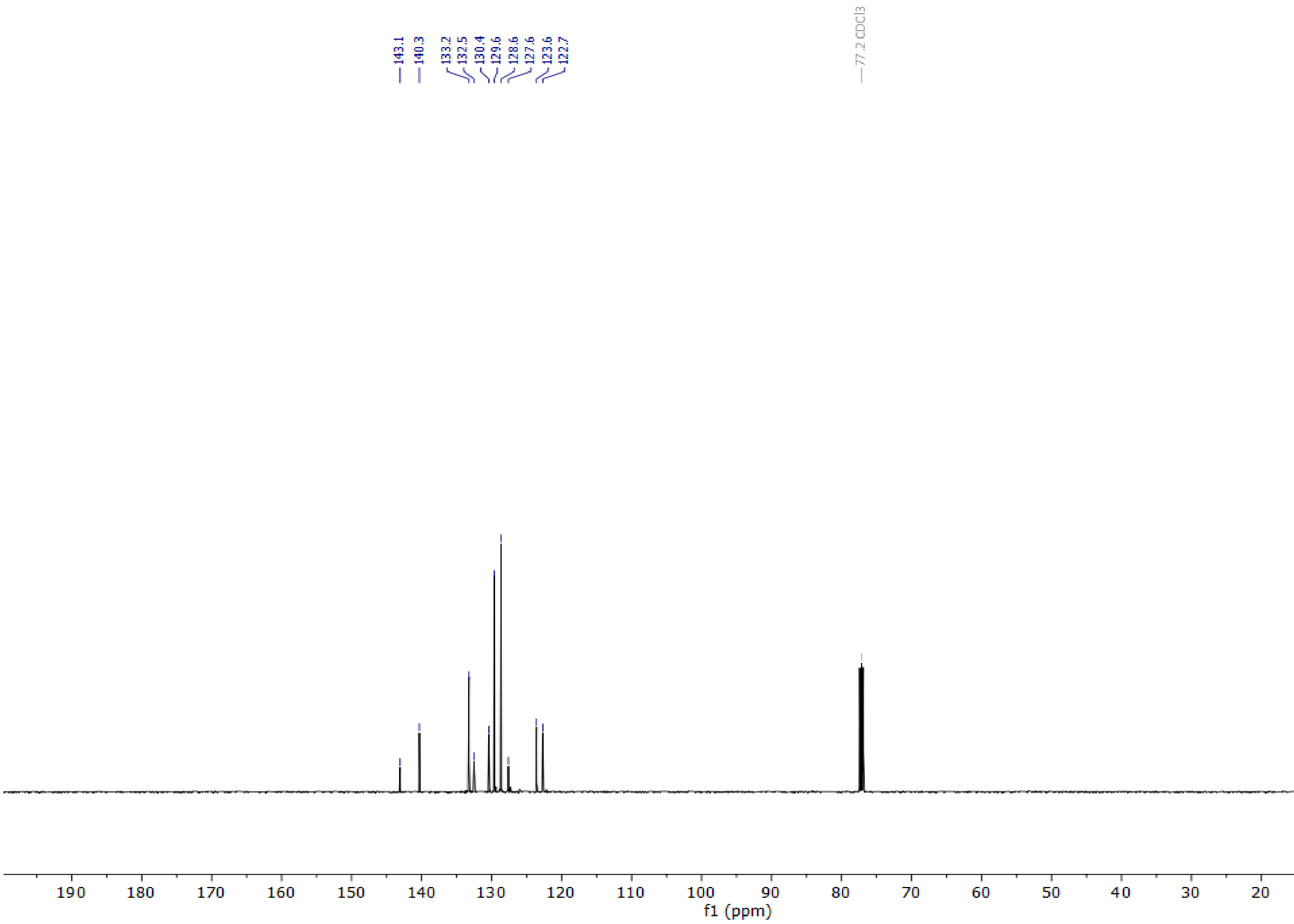

^{1}H NMR of ((2,5-dichlorophenyl)imino)(4-iodophenyl)(methyl)-λ[6]-sulfanone (25)

500 MHz, $CDCl_3$, 298 K

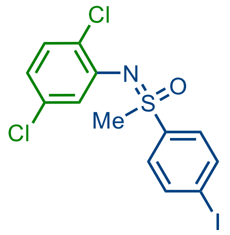

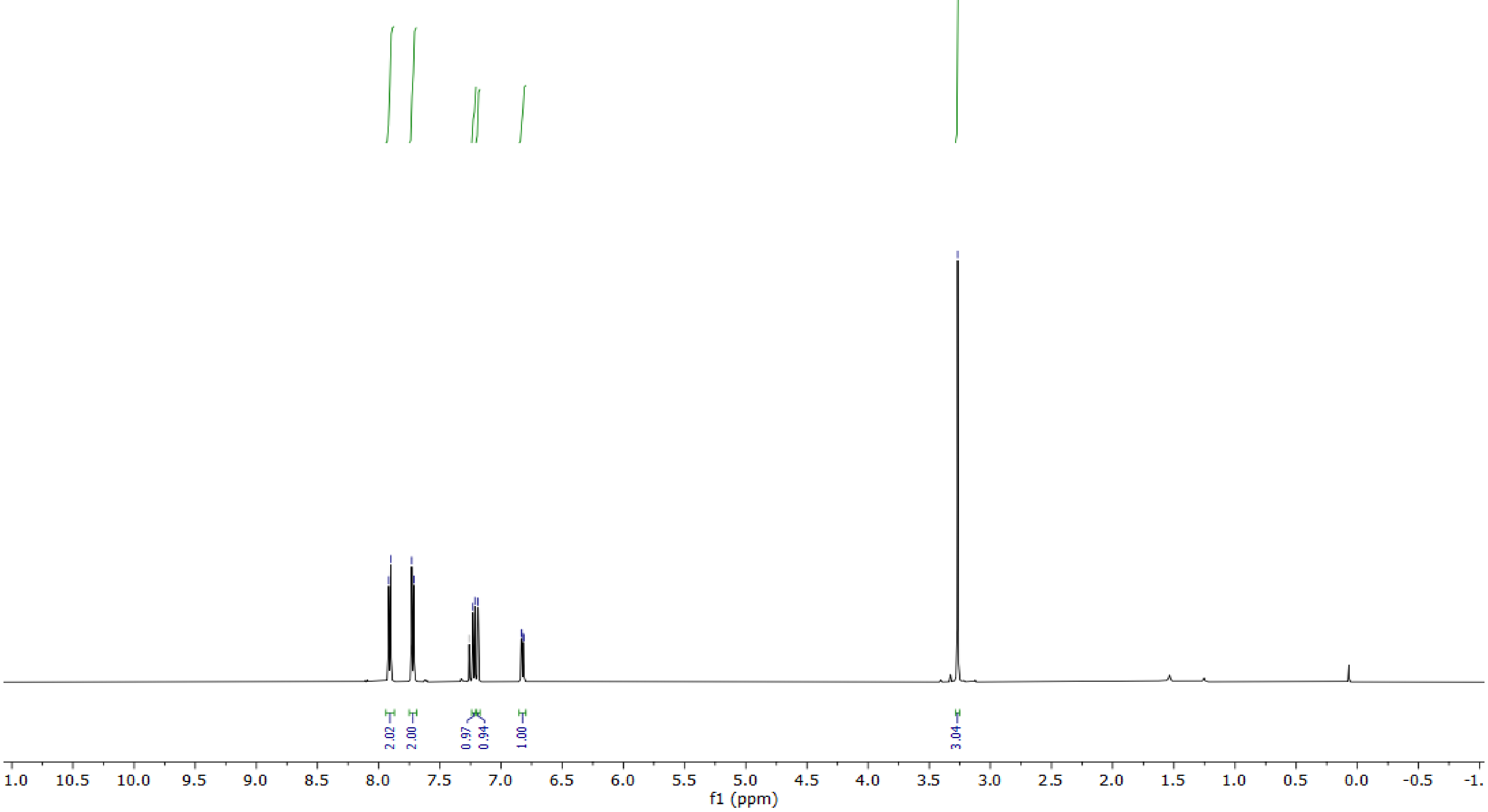

^{13}C NMR of ((2,5-dichlorophenyl)imino)(4-iodophenyl)(methyl)-λ^6-sulfanone (25)

126 MHz, $CDCl_3$, 298 K

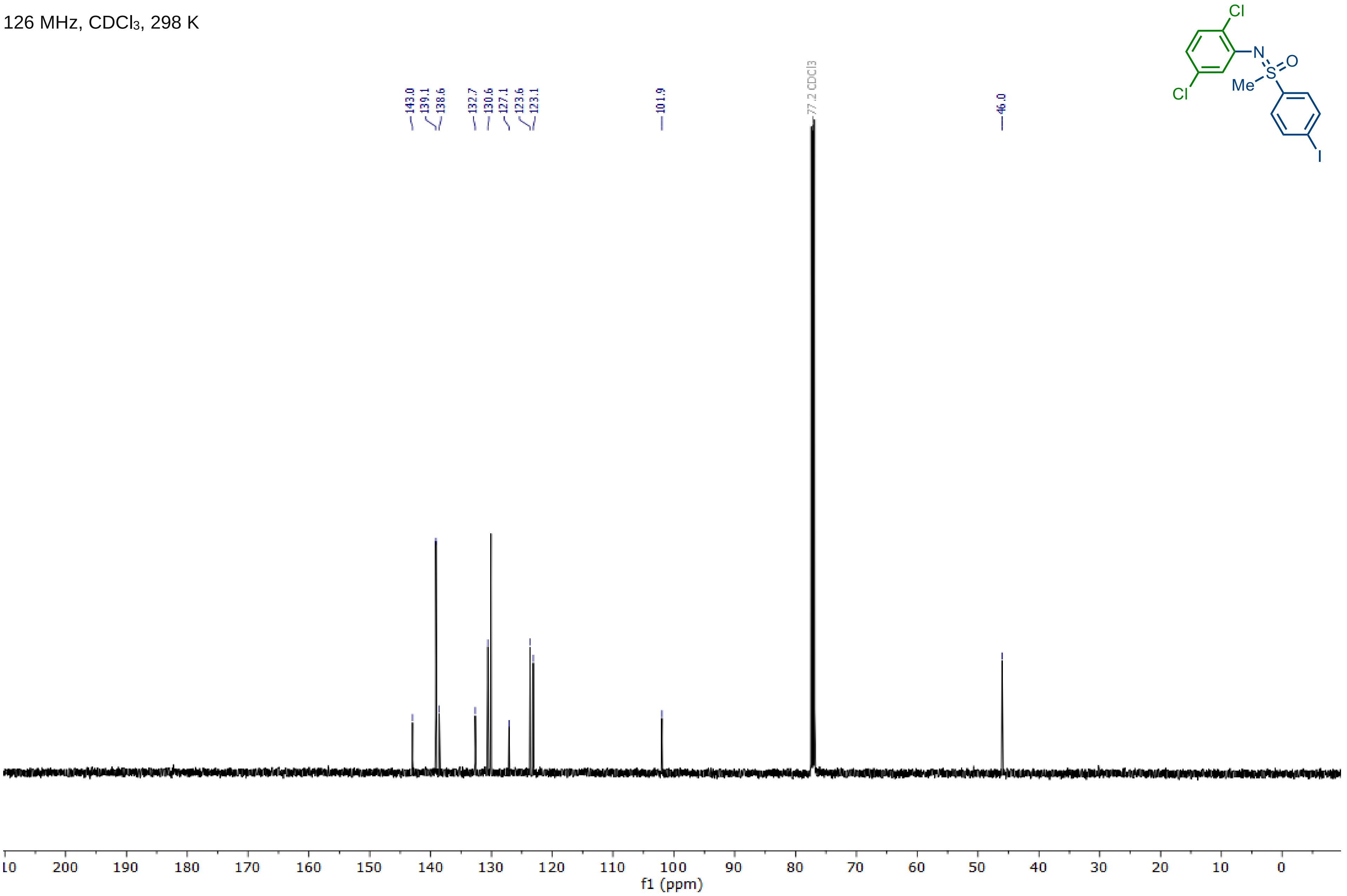

^{1}H NMR of ((2,5-dichlorophenyl)imino)(methyl)(4-(trifluoromethoxy)phenyl)-λ[6]-sulfanone (26)

500 MHz, $CDCl_3$, 298 K

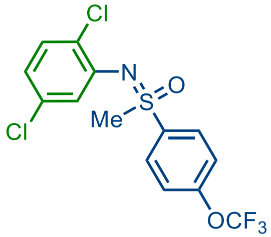

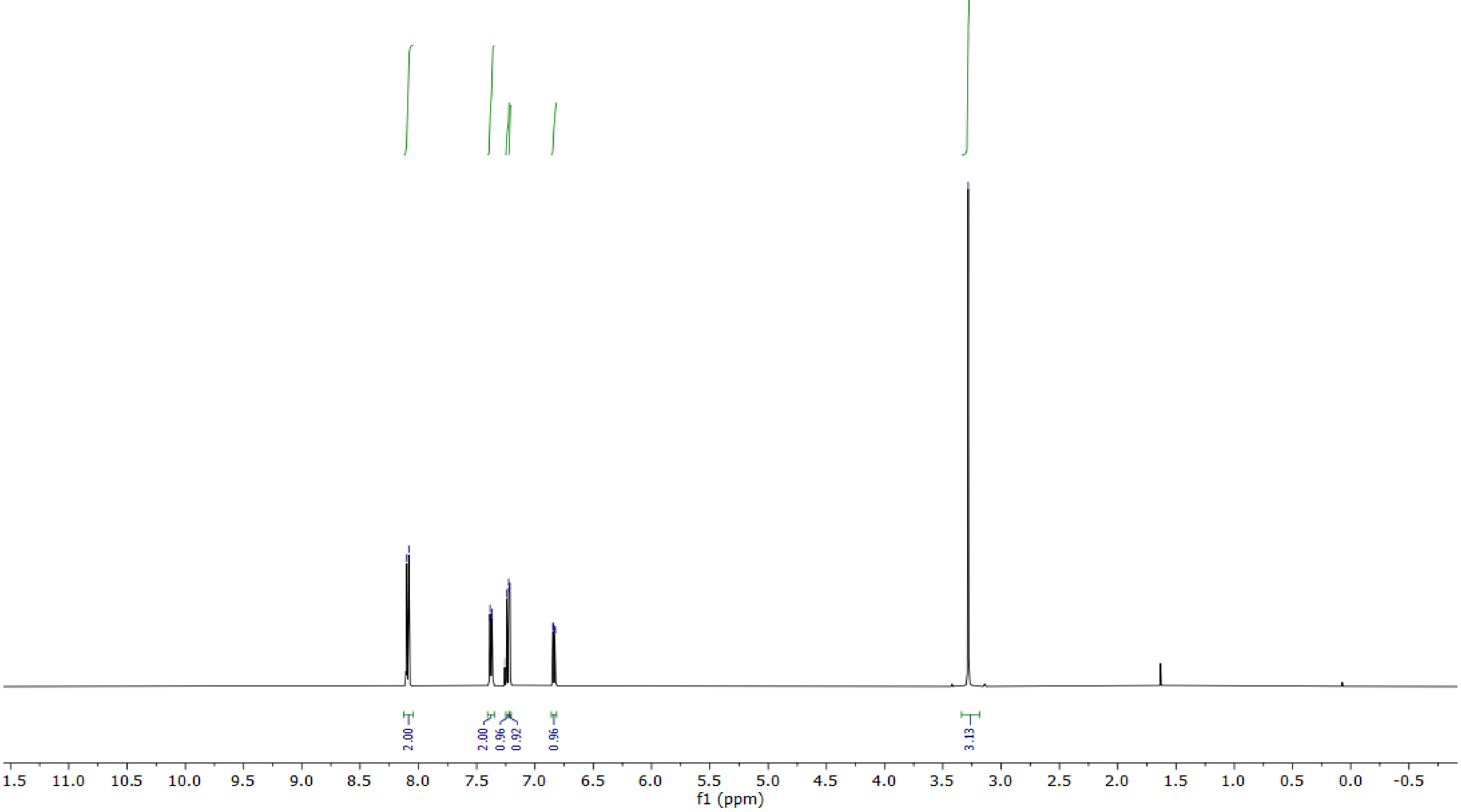

^{13}C NMR of ((2,5-dichlorophenyl)imino)(methyl)(4-(trifluoromethoxy)phenyl)-λ^6-sulfanone (26)

126 MHz, $CDCl_3$, 298 K

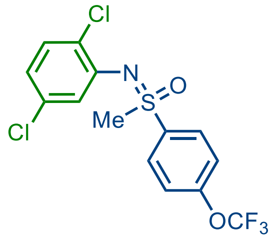

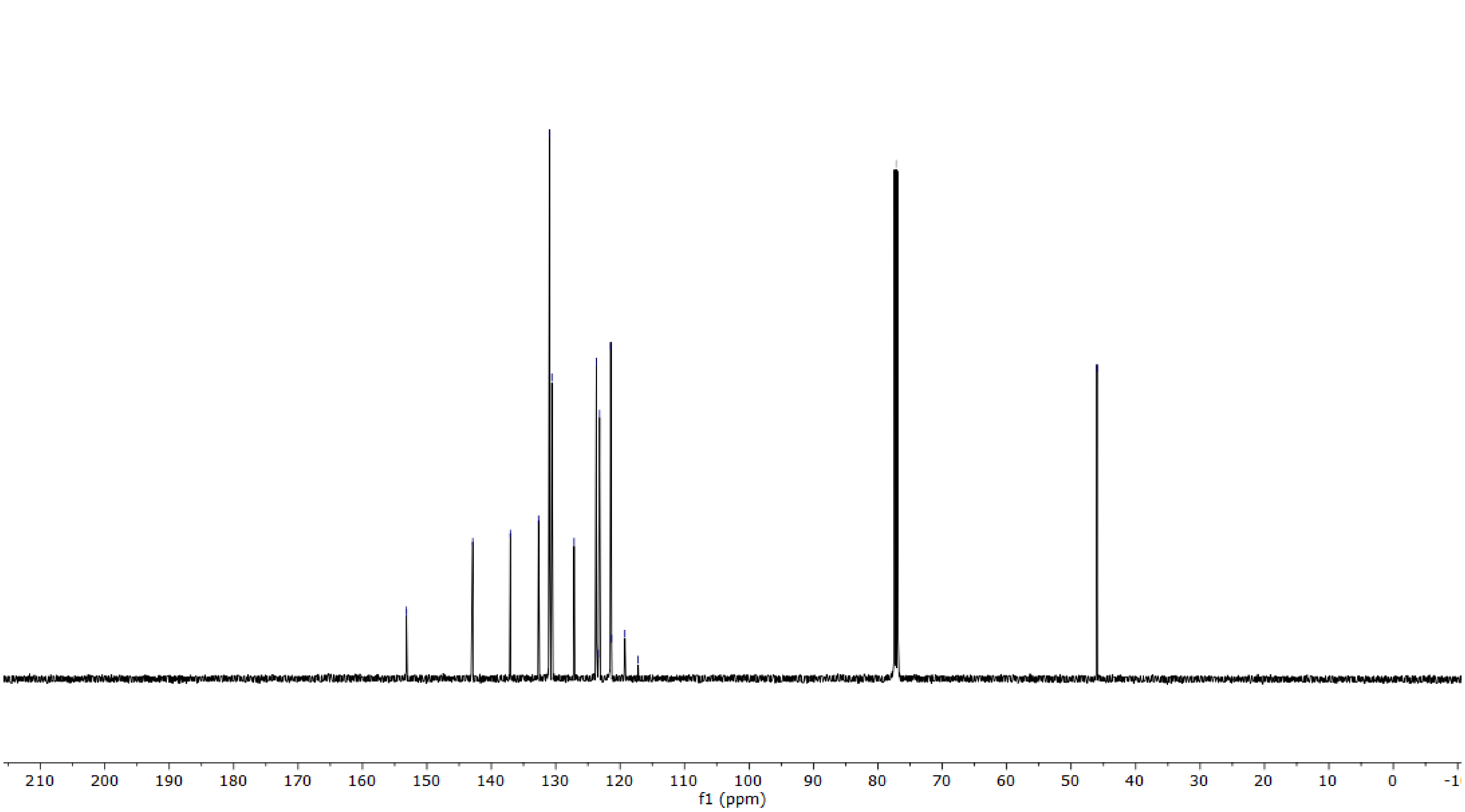

^{19}F NMR of ((2,5-dichlorophenyl)imino)(methyl)(4-(trifluoromethoxy)phenyl)-λ^6-sulfanone (26)

471 MHz, $CDCl_3$, 298 K

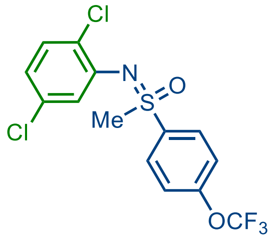

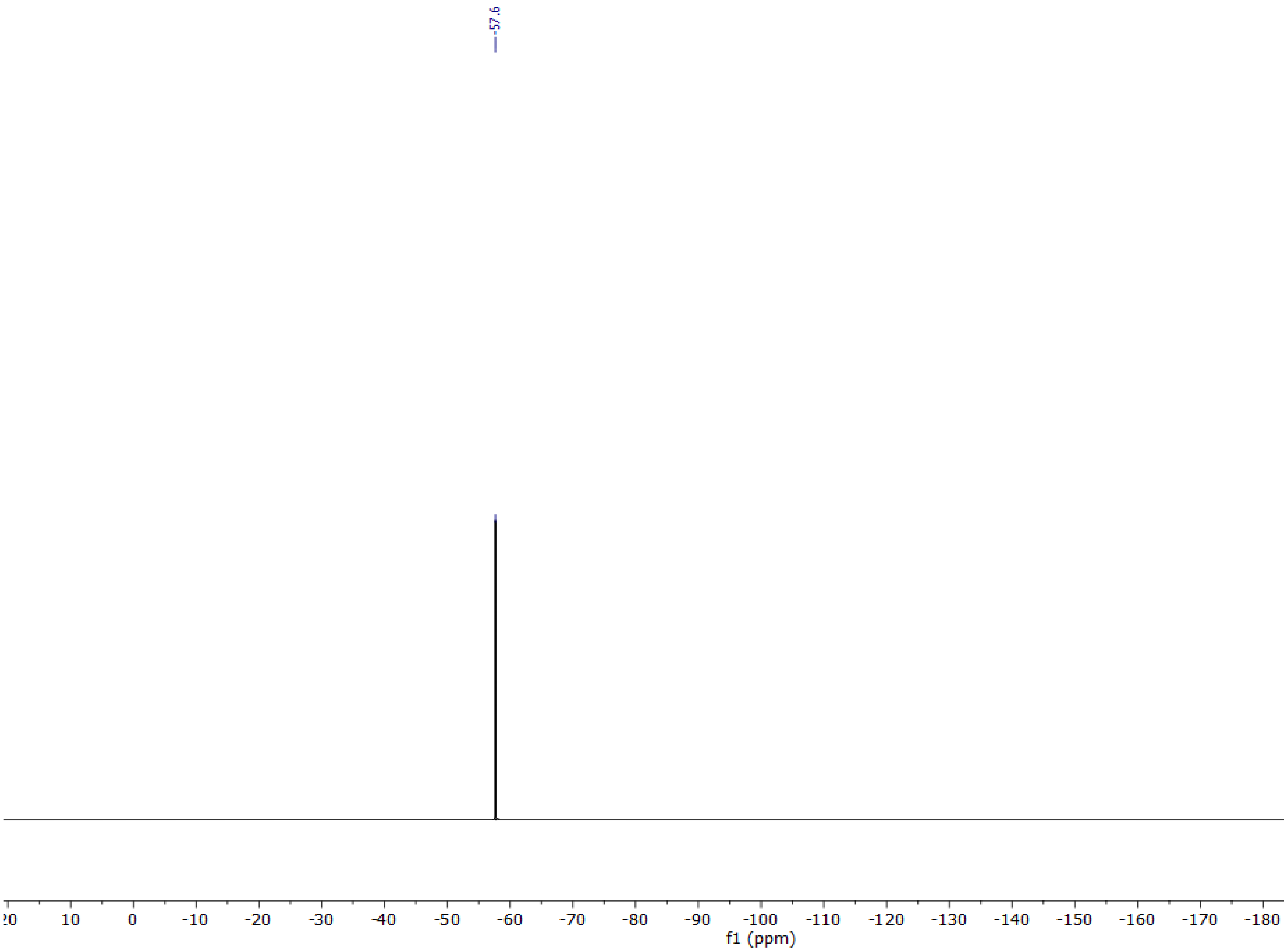

^{1}H NMR of 10-((2,5-dichlorophenyl)imino)-10*H*-10-λ^{4}--phenoxathiine 10-oxide (27)

500 MHz, $CDCl_3$, 298 K

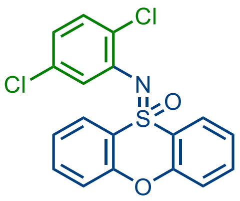

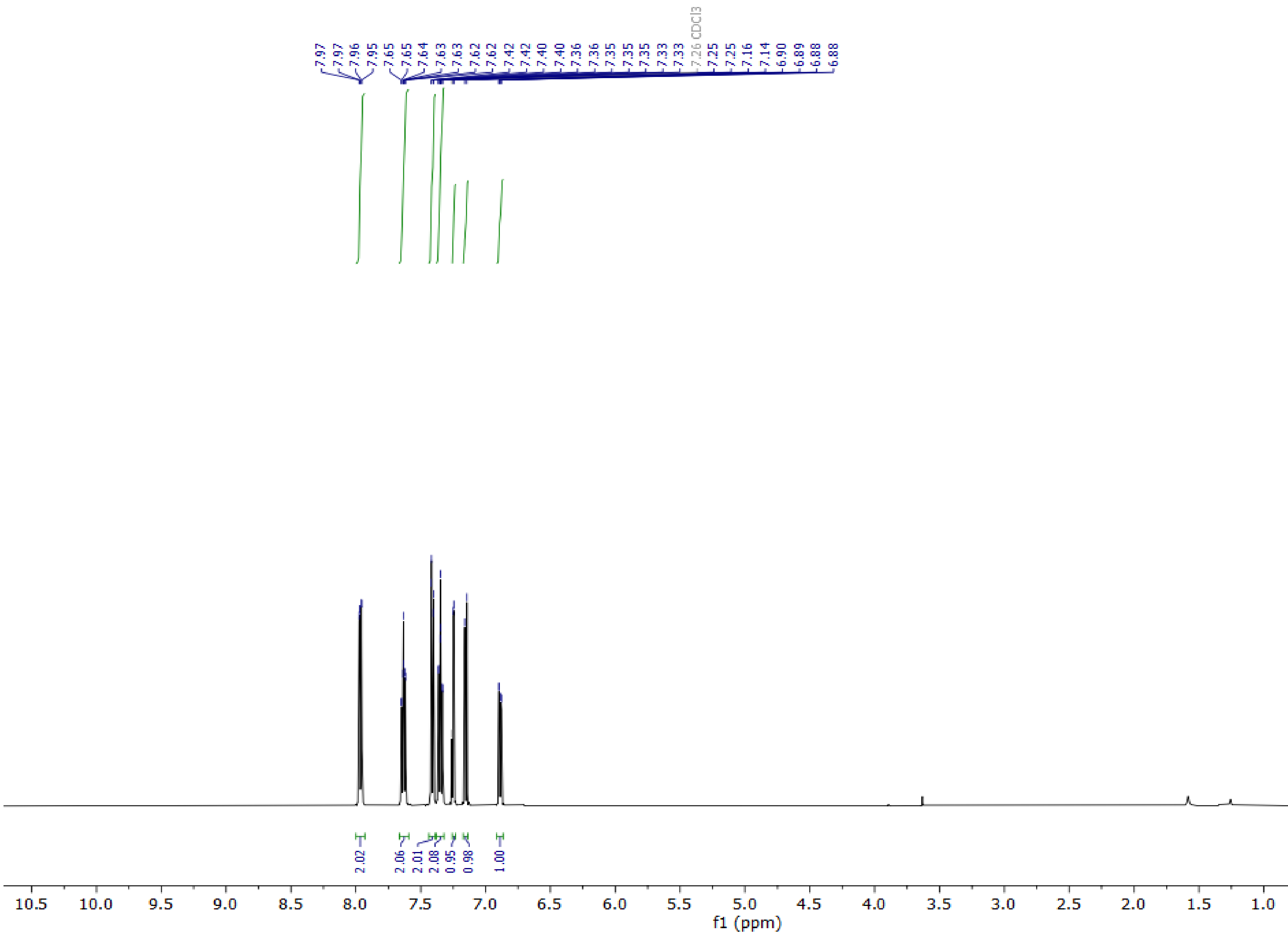

^{13}C NMR of 10-((2,5-dichlorophenyl)imino)-10*H*-10-λ^4--phenoxathiine 10-oxide (27)

126 MHz, $CDCl_3$, 298 K

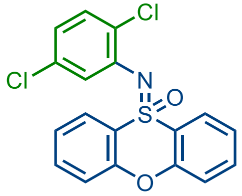

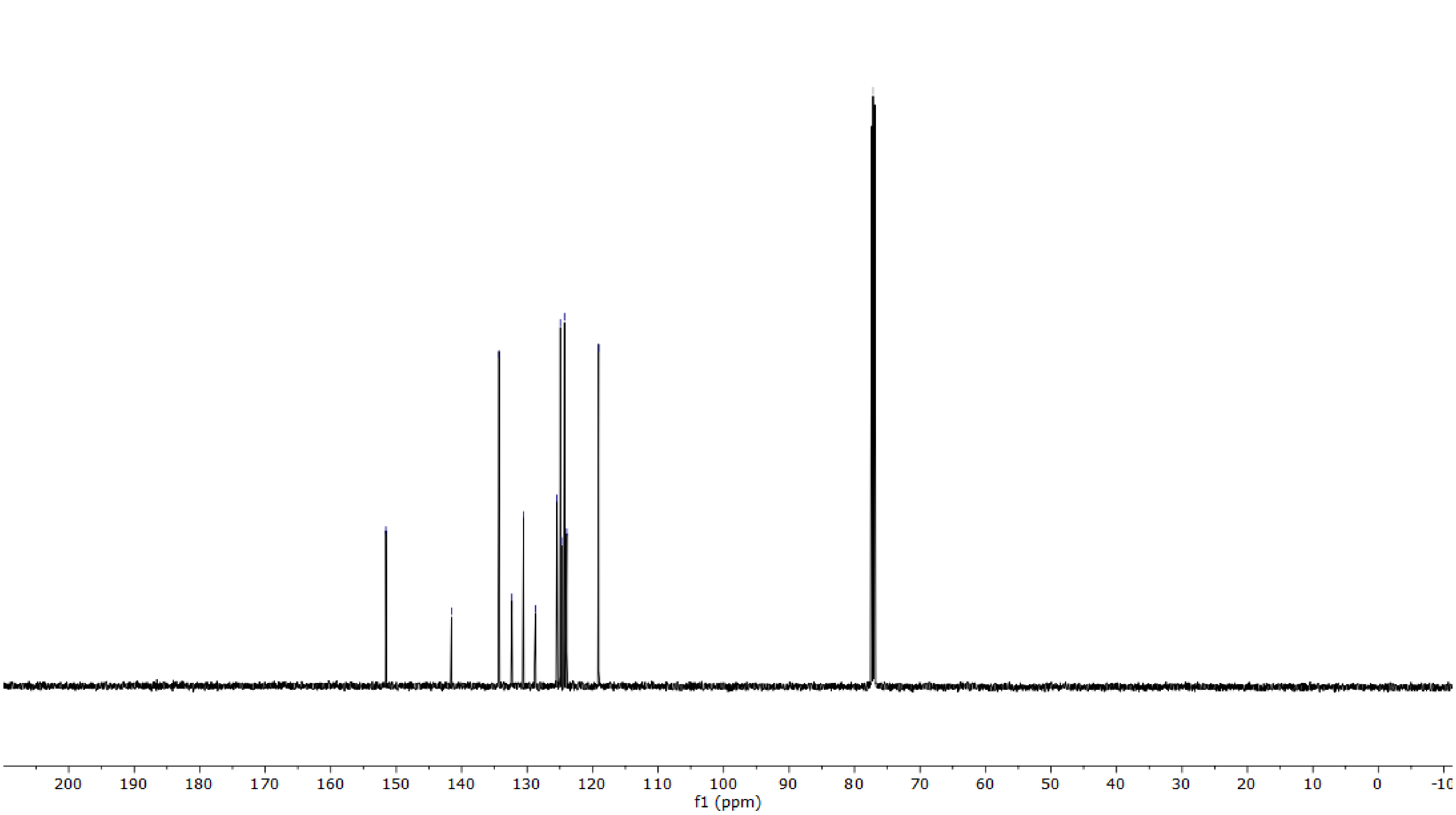

^{1}H NMR of ((2,5-dichlorophenyl)imino)(ethyl)(phenyl)-λ⁶-sulfanone (28)

500 MHz, $CDCl_3$, 298 K

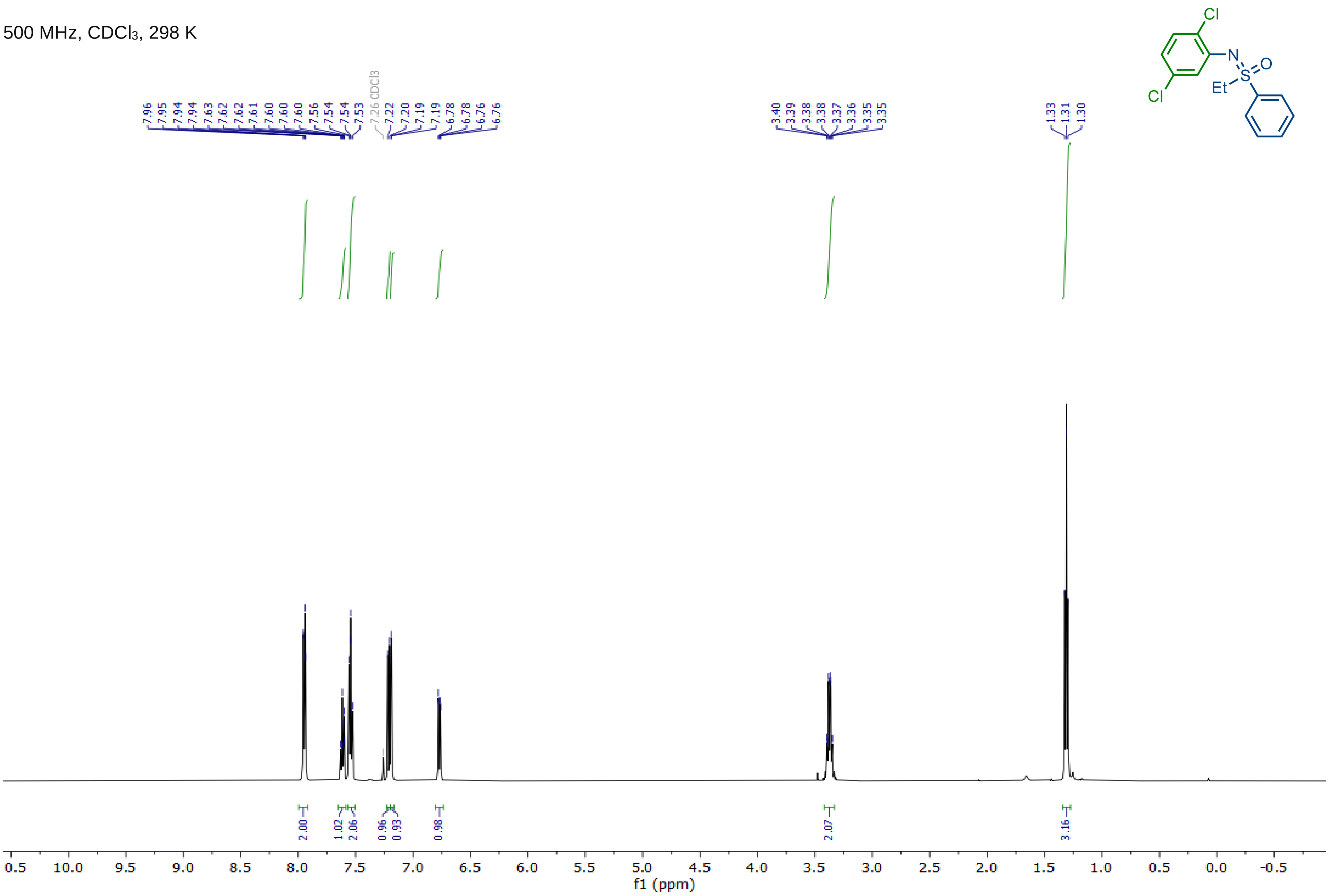

^{13}C NMR of ((2,5-dichlorophenyl)imino)(ethyl)(phenyl)-λ^6-sulfanone (28)

126 MHz, $CDCl_3$, 298 K

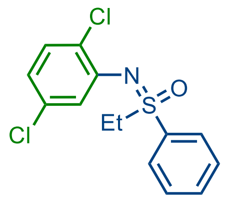

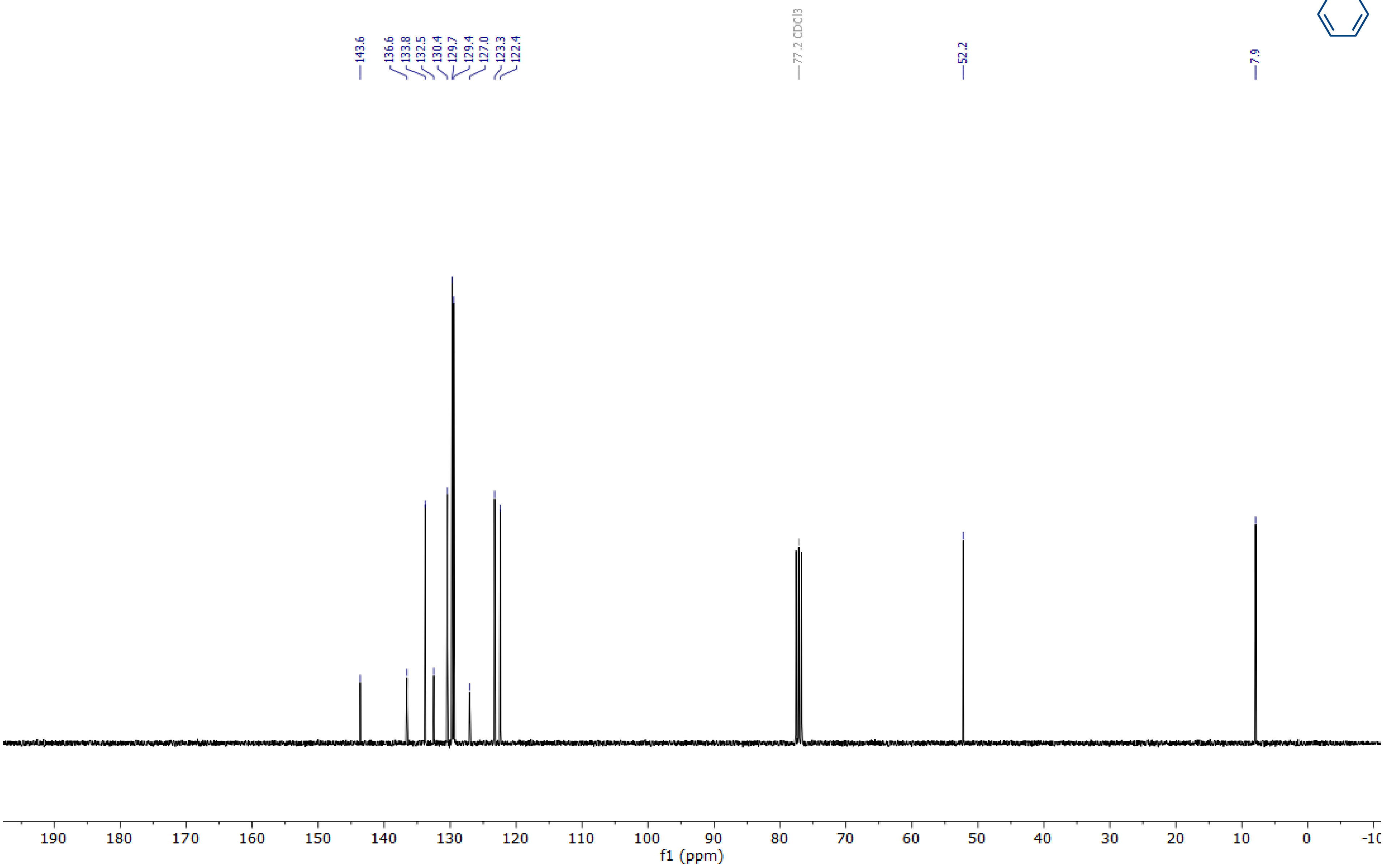

^{1}H NMR of ((2,5-dichlorophenyl)imino)(methyl)(m-tolyl)-λ^{6}-sulfanone (29)

500 MHz, $CDCl_3$, 298 K

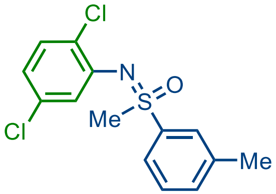

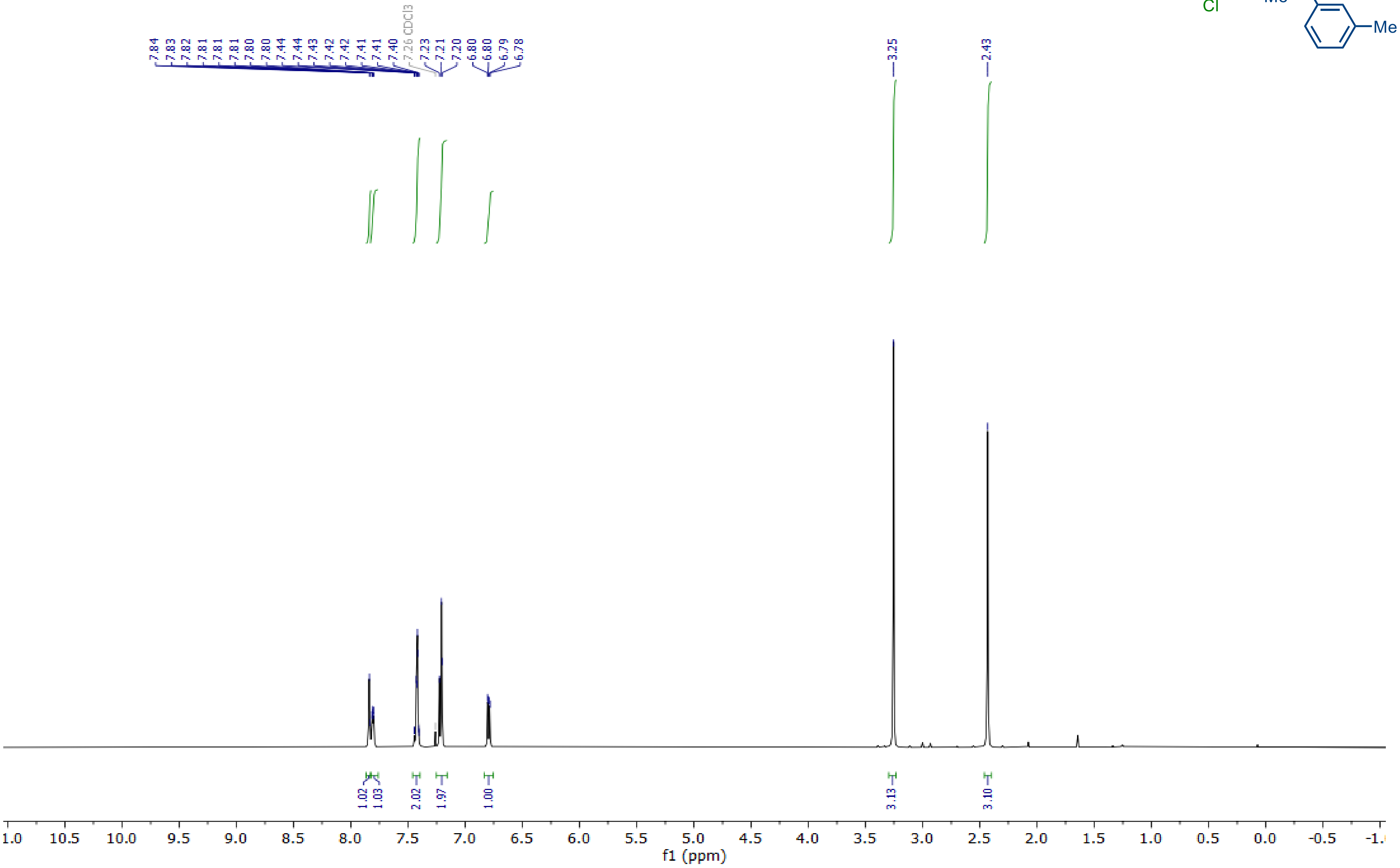

^{13}C NMR of ((2,5-dichlorophenyl)imino)(methyl)(m-tolyl)-λ[6]-sulfanone (29)

126 MHz, $CDCl_3$, 298 K

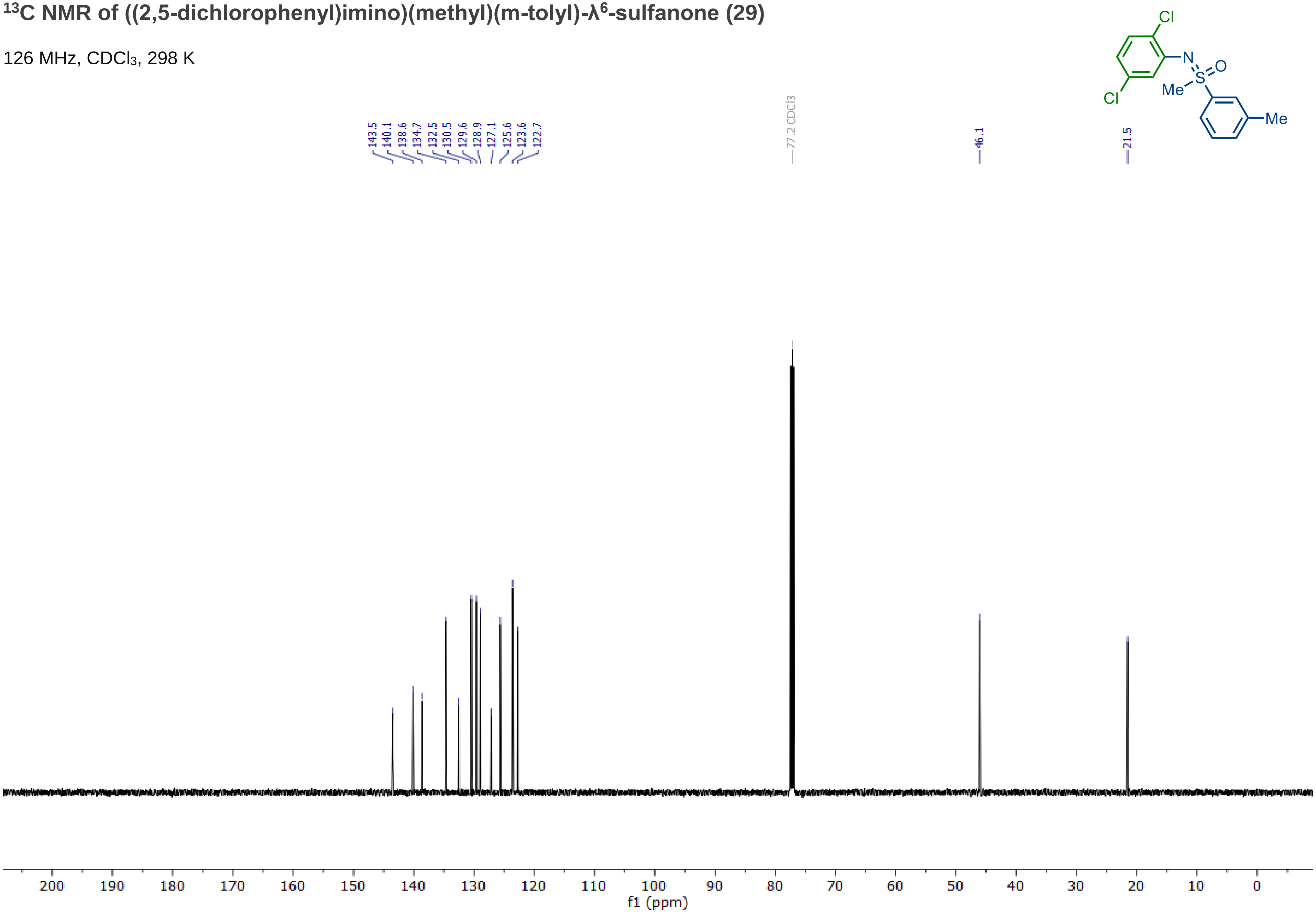

^{1}H NMR of (4-Bromophenyl)((2,5-dichlorophenyl)imino)(methyl)-λ[6]-sulfanone (30)

500 MHz, $CDCl_3$, 298 K

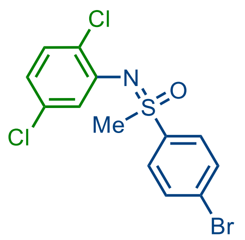

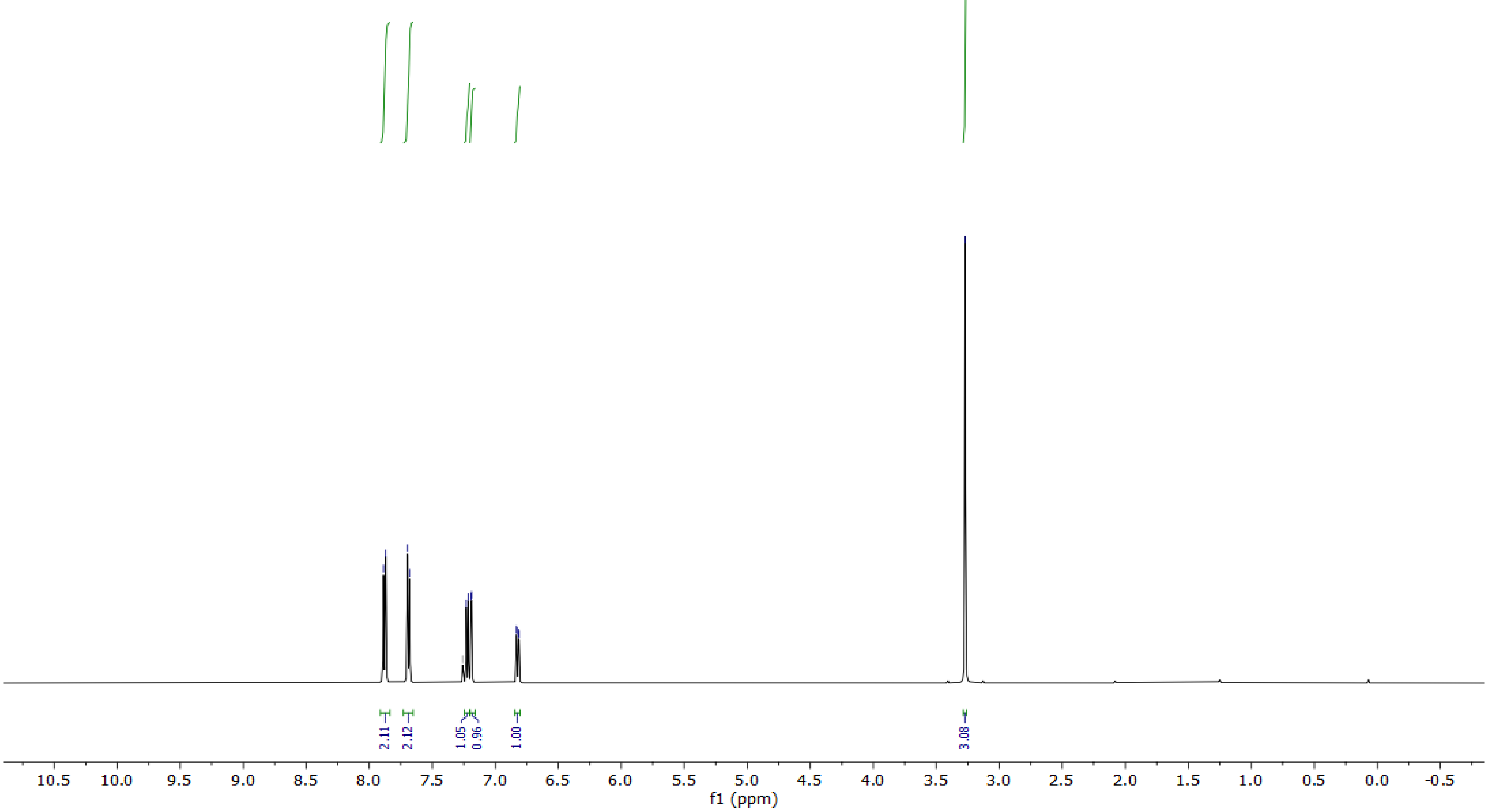

^{13}C NMR of (4-Bromophenyl)((2,5-dichlorophenyl)imino)(methyl)-λ^6-sulfanone (30)

126 MHz, $CDCl_3$, 298 K

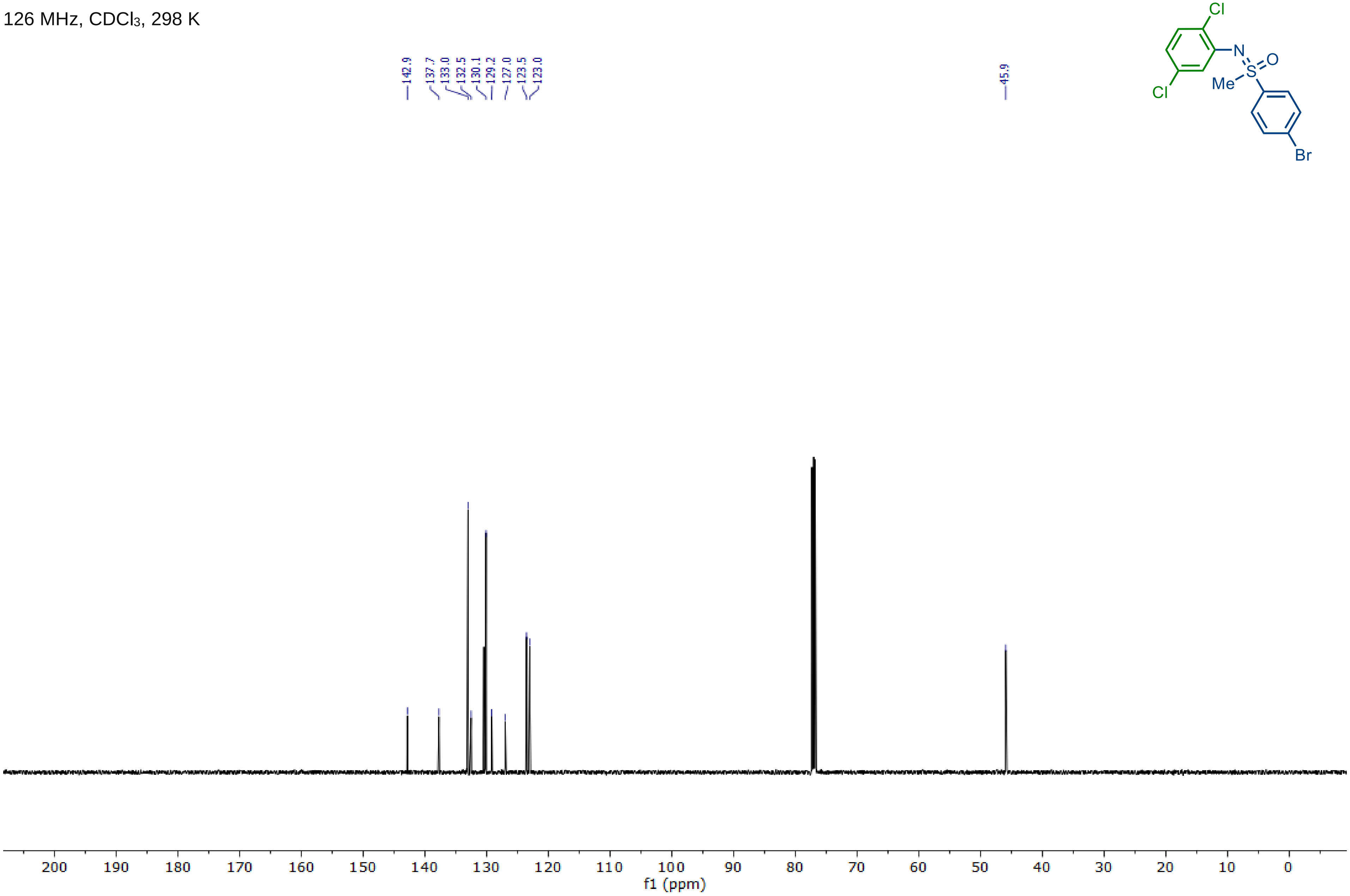

REFERENCES

1. Fulmer, G. R.; Miller, A. J. M.; Sherden, N. H.; Gottlieb, H. E.; Nudelman, A.; Stoltz, B. M.; Bercaw, J. E.; Goldberg, K. I. NMR Chemical Shifts of Trace Impurities: Common Laboratory Solvents, Organics, and Gases in Deuterated Solvents Relevant to the Organometallic Chemist. *Organometallics* **2010**, *29*, 2176–2179.
2. [a]Wang, B.; Han, X.; Li, J.; Li, C.; Liu, H. Sulfoximines-Assisted Rh(III)-Catalyzed C–H Activation and Intramolecular Annulation for the Synthesis of Fused Isochromeno-1,2-Benzothiazines Scaffolds under Room Temperature *Molecules* **2020***, 25,* 2515. [b]Tota, A.; Zenzola, M.; Chawner, S. J.; John-Campbell, S. S.; Carlucci, C.; Romanazzi, G.; Degennaro, L.; Bull, J. A.; Luisi, R., Synthesis of NH-sulfoximines from sulfides by chemoselective one-pot N- and O-transfers. *Chem. Commun.* **2017,** *53*, 348-351.[c]Lohier, J.-F.; Glachet, T.; Marzag, H.; Gaumont, A.-C.; Reboul, V., Mechanistic investigation of the NH-sulfoximination of sulfide. Evidence for λ^6-sulfanenitrile intermediates. *Chem. Commun.* **2017**, 53, 2064-2067.
3. Bohnen, C.; Bolm, C., N-Trifluoromethylthiolated Sulfoximines. *Org. Lett.* **2015**, 17, 3011-3013.
4. Akasaka, T.; Furukawa, N.; Oae, S., Sulfoximidoyl radical. Homolytic addition of N-halosulfoximides to olefins. *Tetrahedron Lett.* **1979**, 20, 2035-2038.
5. Tu, Y.; Shi, P.; Bolm, C., Visible-Light-Mediated α-Ketoacylations of NH-Sulfoximines with gem-Difluoroalkenes. *Org. Lett.* **2022**, 24, 907-911.
6. https://kessil.com/products/science_PR160L.php
7. Lockhart, T. P., Mechanistic investigation of the copper-catalyzed reactions of diphenyliodonium salts. J. *Am. Chem. Soc.* **1983**, 105, 1940-1946.
8. Tang, X.-J.; Dolbier Jr, W. R., Efficient Cu-catalyzed Atom Transfer Radical Addition Reactions of Fluoroalkylsulfonyl Chlorides with Electron-deficient Alkenes Induced by Visible Light. *Angew. Chem. Int. Ed.* **2015**, *54*, 4246-4249.
9. Wang, C.; Tu, Y; Ma, D.; Bolm, C, Photocatalytic Fluoro Sulfoximidations of Styrenes. *Angew. Chem. Int. Ed.* **2020**, *59*, 14134-14137
10. Neeze, F. The ORCA Program System. *WIREs Comput. Mol. Sci.* **2012**, *2*, 73–78.
11. Becke, A. D. Density-Functional Thermochemistry. III. The Role of Exact Exchange. *J. Chem. Phys.* **1993**, *98*, 5648–5652.
12. Lee, C.; Yang, W.; Parr, R. G. Development oft he Colle-Salvetti Correlation-Energy Formula into a Functional of the Electron Density. *Phys. Rev. B.* **1988**, *37*, 785–789.
13. Grimme, S.; Antony, J.; Ehrlich, S.; Krieg, H. A Consistent and Accurate ab initio Parametrization of Density Functional Dispersion Correction (DFT-D) for the 94 Elements H–Pu, *J. Chem. Phys.*, **2010**, *132*, 154104.
14. Grimme, S.; Ehrlich, S.; Goerigk, L. Effect of the Damping Function in Dispersion Corrected Density Functional Theory. *J. Comput. Chem.*, **2011**, *32*, 1456–1465.
15. Weigend, F. Accurate Coulomb-Fitting Basis Sets for H to Rn. *Phys. Chem. Chem. Phys.*, **2006**, *8*, 1057–1065.
16. Weigend, F.; Ahlrichs, R. Balanced Basis Sets of Split Valence, Triple Zeta Valence and Quadruple Zeta Valence Quality for H to Rn: Design and Assessment of Accuracy. *Phys. Chem. Chem. Phys.,* **2005**, *7*, 3297–3305.
17. Valeev, E. F. Libint: A Library for the Evaluation of Molecular Integrals of Many-Body Operators over Gaussian Functions. *http://libint.valeyev.net/*.
18. Hanwell, M. D.; Curtis, D. E.; Lonie, D. C.; Vandermeersch, T,; Zurek, E.; Hutchison, G. R. Avogadro: an Advanced Semantic Chemical Editor, Visualization, and Analysis Platform. *J. Cheminformatics*, **2012**, *4*, 17.
